# *GmSYP24*, a putative syntaxin gene, confers osmotic/drought, salt stress tolerances and ABA signal pathway

**DOI:** 10.1038/s41598-019-42332-5

**Published:** 2019-04-12

**Authors:** Li-Miao Chen, Yi-Sheng Fang, Chan-Juan Zhang, Qing-Nan Hao, Dong Cao, Song-Li Yuan, Hai-Feng Chen, Zhong-Lu Yang, Shui-Lian Chen, Zhi-Hui Shan, Bao-Hong Liu, Jing-Wang, Yong Zhan, Xiao-Juan Zhang, De-Zhen Qiu, Wen-Bin Li, Xin-An Zhou

**Affiliations:** 1 Key Laboratory of Oil Crop Biology, Ministry of Agriculture, Wuhan, 430062 China; 2 Oil Crops Research Institute of Chinese Academy of Agriculture Sciences, Wuhan, 430062 China; 3 Key Laboratory of Soybean Biology in the Chinese Ministry of Education, Northeast Agricultural University, Harbin, 150030 China; 4 Division of Soybean Breeding and Seed, Soybean Research & Development Center, CARS (Key Laboratory of Biology and Genetics & Breeding for Soybean in Northeast China, Ministry of Agriculture), Harbin, 150030 China; 5 Crop Research Institute, Xinjiang Academy of Agricultural and Reclamation Science, Key Lab of Cereal Quality Research and Genetic Improvement, Xinjiang Production and Construction Crops, 832000 Shihezi, China

## Abstract

As major environment factors, drought or high salinity affect crop growth, development and yield. Transgenic approach is an effective way to improve abiotic stress tolerance of crops. In this study, we comparatively analyzed gene structures, genome location, and the evolution of syntaxin proteins containing late embryogenesis abundant (LEA2) domain. *GmSYP24* was identified as a dehydration-responsive gene. Our study showed that the GmSYP24 protein was located on the cell membrane. The overexpression of GmSYP24 (*GmSYP24*ox) in soybean and heteroexpression of GmSYP24 (*GmSYP24*hx) in Arabidopsis exhibited insensitivity to osmotic/drought and high salinity. However, wild type soybean, Arabidopsis, and the mutant of *GmSYP24* homologous gene of Arabidopsis were sensitive to the stresses. Under the abiotic stresses, transgenic soybean plants had greater water content and higher activities of POD, SOD compared with non-transgenic controls. And the leaf stomatal density and opening were reduced in transgenic Arabidopsis. The sensitivity to ABA was decreased during seed germination of *GmSYP24ox* and *GmSYP24*hx. *GmSYP24*hx induced up-regulation of ABA-responsive genes. *GmSYP24*ox alters the expression of some aquaporins under osmotic/drought, salt, or ABA treatment. These results demonstrated that *GmSYP24* played an important role in osmotic/drought or salt tolerance in ABA signal pathway.

## Introduction

As an important legume crop, soybean can be processed into a variety of beans and oil, or used to feed livestock. The global demand for soybean is increasing for various uses. However, the quality and yield of soybean are affected by drought or high salinity stress. To adapt to adverse environments, plants have developed series of defense response requiring lots of genes regulated by abiotic stresses. Therefore, it is necessary to explore the key drought/salt-tolerant genes in order to develop high drought-tolerant cultivars of soybean. In the present study, we established a stable soybean transformation system according to previous study^1^, and our findings suggested that transgenic approach could be used to effectively improve abiotic stresses tolerance of soybean.

In plants, the syntaxin proteins are a set of crucial membrane proteins involved in vesicle trafficking^2,3^. SYP61 plays a role in the exocytic trafficking and the transport of cell wall components to the plasma membrane. Some studies have demonstrated that two syntaxin proteins (AtSYP61/ AtSYP121) interact and coordinate the trafficking of plasma membrane aquaporin PIP2–7 to modulate the water permeability of cell membrane^4,5^. Syntaxin proteins are not limited to vesicle trafficking, and they may be involved in abiotic stresses. It has been reported that the expression of *NtSYP121* integrates with ion channel regulation^6^. The mutant plants of AtSYP61 are more prone to wilting under limited soil moisture than wild type^7^. Overexpression of *OsSYP21* can increase tolerance to oxidative stress and resistance to rice blast^8^. However, the syntaxin proteins (containing LEA2 domain, PF03168) are special in LEA2 protein subfamily. It remains unclear about the structure and evolution of syntaxin proteins. There is no direct evidence showing that the soybean syntaxin proteins participate in ABA response, drought and salt tolerance, and seed germination.

In the present study, we performed the analysis of syntaxin proteins containing LEA2 domain in soybean. A syntaxin gene harboring LEA2 domain, *GmSYP24*, was identified. The expression of *GmSYP24* was greatly and rapidly induced by drought or salt stress in drought-tolerant cultivar. Moreover, we generated the GmSYP24 transgenic soybean and Arabidopsis plants. Our results showed that *GmSYP24*ox and *GmSYP24*hx decreased the sensitivity to osmotic/drought or salt stress of plants. The seeds of *GmSYP24*ox and *GmSYP24*hx transgenic plants were less sensitive to ABA than wild type.

## Results

### The search of the proteins containing LEA2 domain in soybean

The key word searching results showed that there were 206 candidate sequences harboring LEA2 domains in the soybean genome databases. A total of 120 genes in different soybean loci were identified by removing redundant sequences and incomplete open reading frame (ORF) sequences. These putative protein sequences contained the conserved LEA2 domains except for seven genes. Finally, 113 soybean genes were named GmLEA2-1 to GmLEA2-113 according to their chromosomal positions^9,10^. Table S1 lists the detailed information of these genes. Table S2 lists the nucleotide and amino acid sequences of the 113 soybean LEA2 proteins. The length of nucleotide sequences of the 113 LEA2 genes ranged from 369 to 9,377 bp, and most of them contained a single LEA2 domain. However, GmLEA2–9, GmLEA2–79 and GmLEA2–101 included two LEA2 domains.

### Phylogenetic analysis and genome location of the syntaxin genes containing LEA2 domain

**Figure 1:**
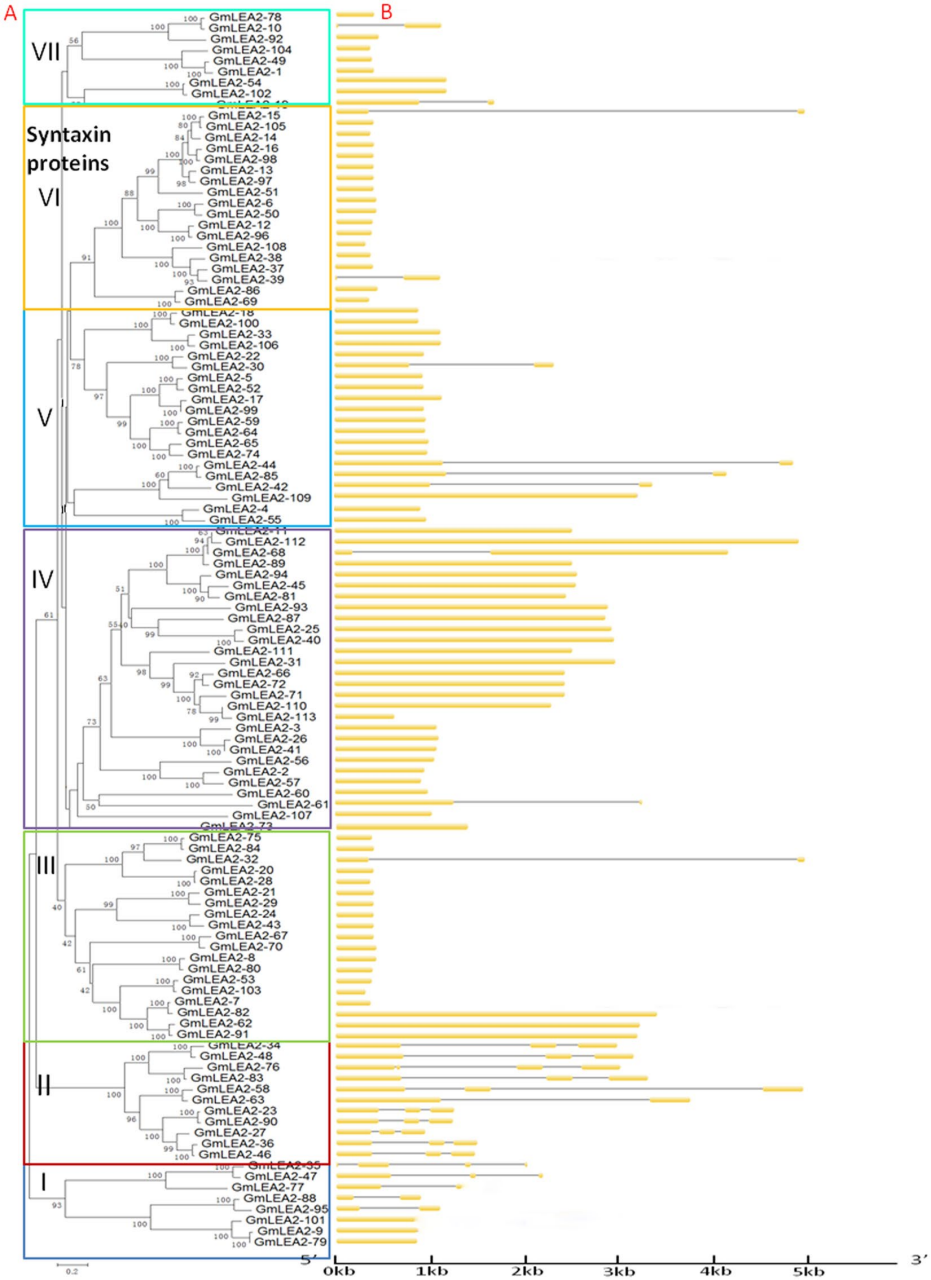
Phylogenetic analysis and gene structure of soybean genes harboring LEA2 domain. (**A**) Phylogenetic tree or (**B**) Exon/intron structures of soybean LEA2 proteins Yellow boxes represent exons, and black lines indicate introns.

The alignments of the 113 full-length LEA2 protein sequences were performed. Figure 1 shows that these proteins could be divided into seven groups according to the phylogenetic tree. The members within each subgroup showed similar exon/intron structures. Notably, subfamily VI contained 18 members, most of which were syntaxin proteins with a single exon. Phylogenetic analysis revealed that these syntaxin proteins possibly evolved from primary LEA2 proteins of subfamily I. Seven pairs of syntaxin proteins had a high degree of homology of each subfamily, suggesting that they were putative paralogous pairs with sequence identity ranging from 86% to 98% (Table S3).

**Figure 2:**
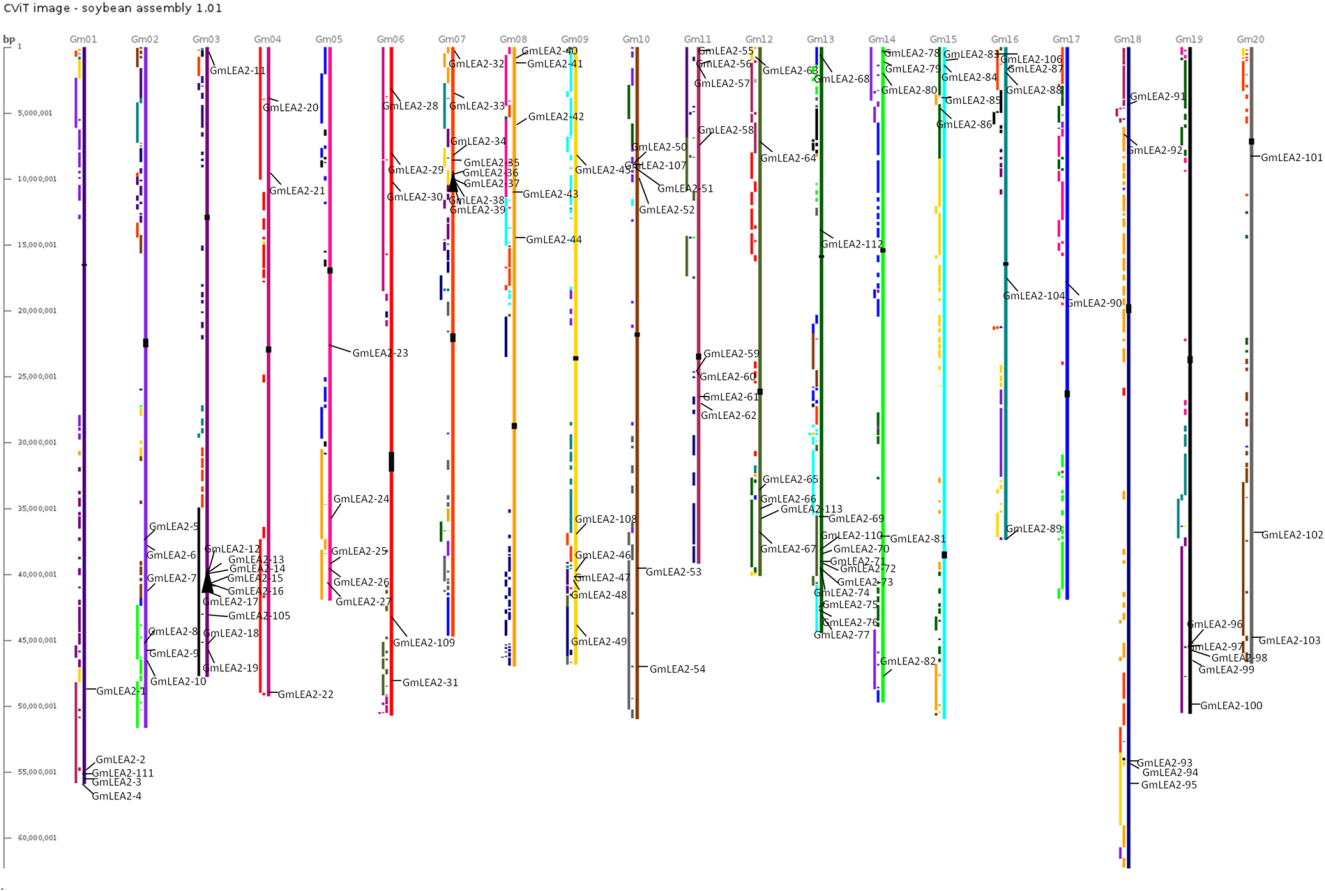
Chromosomal location and region duplication of soybean LEA2 genes. The black triangle Icon were marked as tandem duplication of syntaxin proteins on chromosome 3 and 7. The scale on the left represents the length of the chromosome.

Figure 2 shows the distribution of 113 genes containing LEA2 domain on soybean chromosomes. Gene duplications are the main events of gene-family expansion. It mainly includes three categories, such as segmental duplication, tandem duplication and transposition events^11^. In this research, the syntaxin genes were mapped onto the duplicated blocks in order to identify duplicate patterns during genome evolution. Four out of seven putative paralogous pairs were located in segmental duplication blocks, and two were located in tandem duplication blocks, which were marked using black triangle Icon (Fig. 2, Table S3). Among them, Gm LEA2-12/Gm LEA2-13/Gm LEA2-15/Gm LEA2-16 and Gm LEA2-37/Gm LEA2-39 existed between 20-kb to 25-kb segments on chromosome 3 and 7, respectively, while other three genes Gm LEA2-96/Gm LEA2-97/Gm LEA2-98 were located in 8.1-kb segments on chromosome 19 (Fig. 2). Some syntaxin proteins might be generated from segment and tandem duplications of primary LEA2 proteins.

### Evolutionary relationships among syntaxin genes containing LEA2 domain in soybean and Arabidopsis

**Figure 3:**
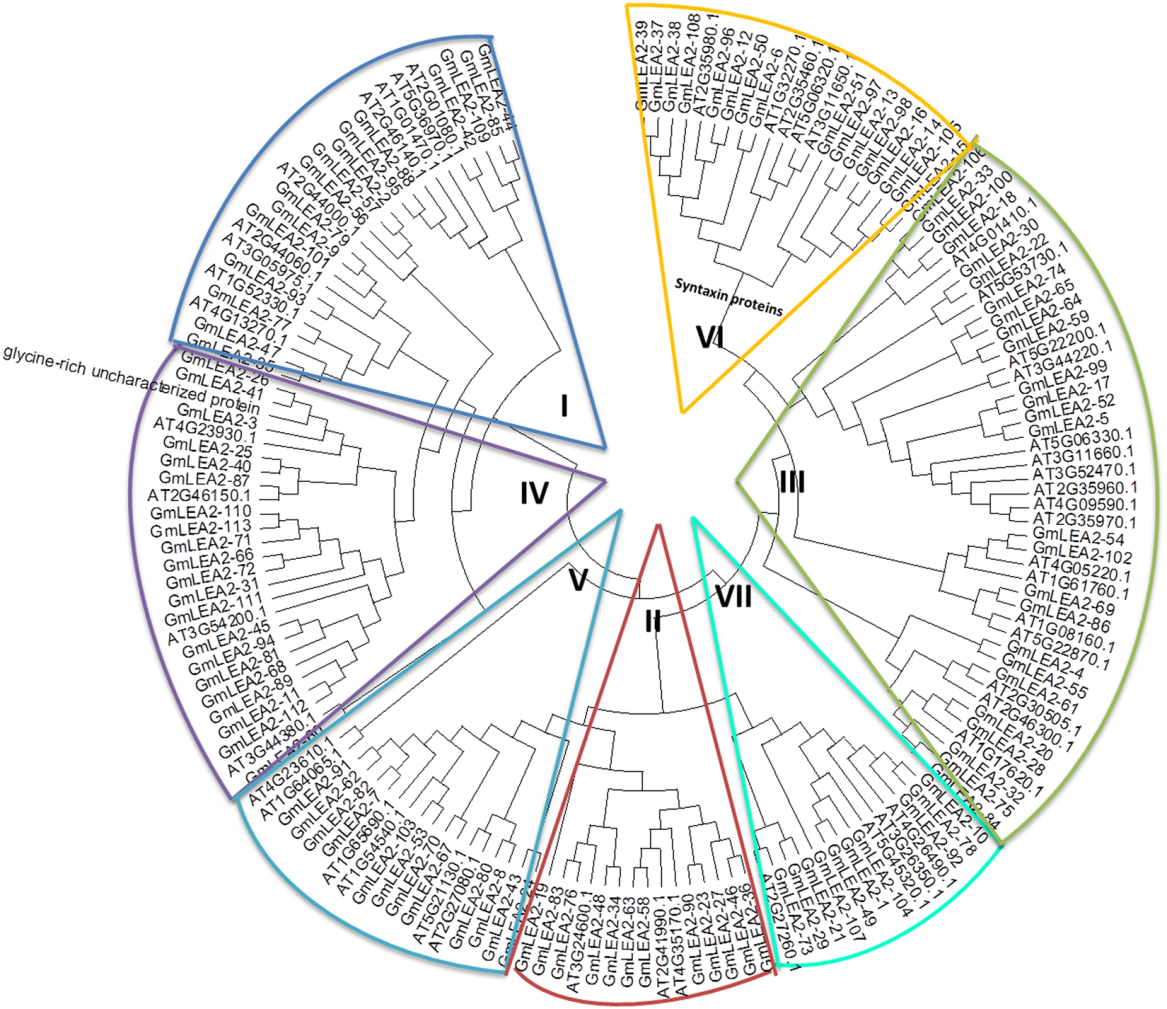
Phylogenetic tree of the amino acid sequences of LEA2 domains from soybean and Arabidopsis. The tree was conducted based on the LEA2 amino acid sequences using MEGA 6.0 by the neighbor-joining method with 1000 bootstrap replicates.

To understand the phylogenetic relationships of syntaxin proteins containing LEA2 domain in soybean and Arabidopsis, we aligned 113 proteins and 49 Arabidopsis proteins containing GmLEA2 domain. Table S4 lists the amino acid sequences of Arabidopsis proteins containing LEA2 domain. These proteins and soybean LEA2 proteins could be divided into seven subgroups (Fig. 3). The end of the phylogenetic tree (subgroup VI) was syntaxin proteins. In general, the LEA2 proteins from two higher plants were interspersedly distributed in all subgroups, indicating the expansion of LEA2 proteins before the divergence of soybean and Arabidopsis.

The LEA2 domain-containing syntaxin proteins consisted of 16 soybean members and five Arabidopsis members. Eight soybean LEA2 proteins (GmLEA2-12, GmLEA2-96, GmLEA2-6, GmLEA2-50, GmLEA2-108, GmLEA2-38, GmLEA2-37 and GmLEA2-39) were clustered with the Arabidopsis ones (AT2G35460, AT2G35980, AT1G32270, AT3G11650 and AT5G06320). Other eight genes (GmLEA2-13, GmLEA2-14, GmLEA2-15, GmLEA2-16, GmLEA2-51, GmLEA2-97, GmLEA2-98 and GmLEA2-105) were clustered in one branch. The seven Arabidopsis genes were searched in the Information Resource (Tair, http://www.arabidopsis.org/). *AT1G32270* was annotated to be involved in intra-Golgi vesicle-mediated transport by GO biological process. *AT2G35980*, *AT3G11650* and *AT5G06320* were annotated to be involved in defense response to virus by GO biological process. The Arabidopsis protein (AT2G35980, also called NHL10) had been reported to involving in cucumber mosaic virus response and leave senescence^12^. It prompted us to better understand biological functions of soybean syntaxin proteins harboring LEA2 domain by identifying evolutionary relationships of LEA2 proteins from soybean and Arabidopsis.

### The expression characterization of *GmSYP24* and subcellular localization

**Figure 4:**
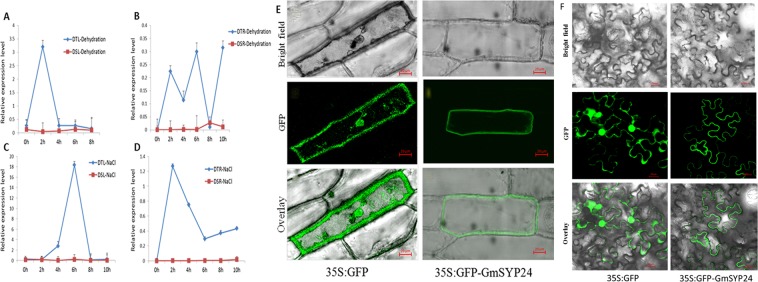
The expression pattern of *GmSYP24* and sub-cellular location. Bars indicate SE of the mean. (**A**–**D**), The expression of *GmSYP24* in the leaf or root of drought-tolerant and -sensitive under dehydration or salt. (**E**) The sub-cellular location of GmSYP24 protein in onion epidermal cells. (**F**) The sub-cellular location of GmSYP24 protein in tobacco epidermal cells. Scale bars = 20 μm.

*GmSYP24* (GmLEA2-96, annotated a syntaxin gene) cDNA was isolated from a dehydration-stressed soybean DGE sequencing. The conserved LEA2 domain was located in 93-195 amino acids. There were no differences in the amplified encoding and promoter sequences of *GmSYP24* from drought-tolerant and drought-sensitive varieties. However, there were significant expression differences of *GmSYP24* between the two varieties under dehydration or salt stress. The results showed that the expression of *GmSYP24* was highly induced in leaves of drought-tolerant variety, and the highest expression was up to 3.5 or 20 times, respectively. There was almost no expression of *GmSYP24* in the drought-sensitive variety. Its expression in leaves or roots under salt stress was rapidly increased, which peaked at 6 h and 2 h, respectively, and then the expression was gradually decreased. Under drought, the peak time of its expression in leaves was at 2 h, while there were fluctuations of expression level in roots (Fig. 4A–D).

To determine the sub-cellular location of GmSYP24, a GmSYP24-GFP fusion protein, together with the control plasmid 35S-GFP was transformed into onion or tobacco epidermal cell, and visualized under a confocal laser scanning microscopy. The results indicated that the GmSYP24-GFP fusion protein was specifically localized in the cell membrane, whereas GFP alone showed ubiquitous distribution in the whole cell (Fig. 4E,F).

### *GmSYP24hx* plants/the mutant of homologous genes changes tolerance to the stresses in Arabidopsis

**Figure 5:**
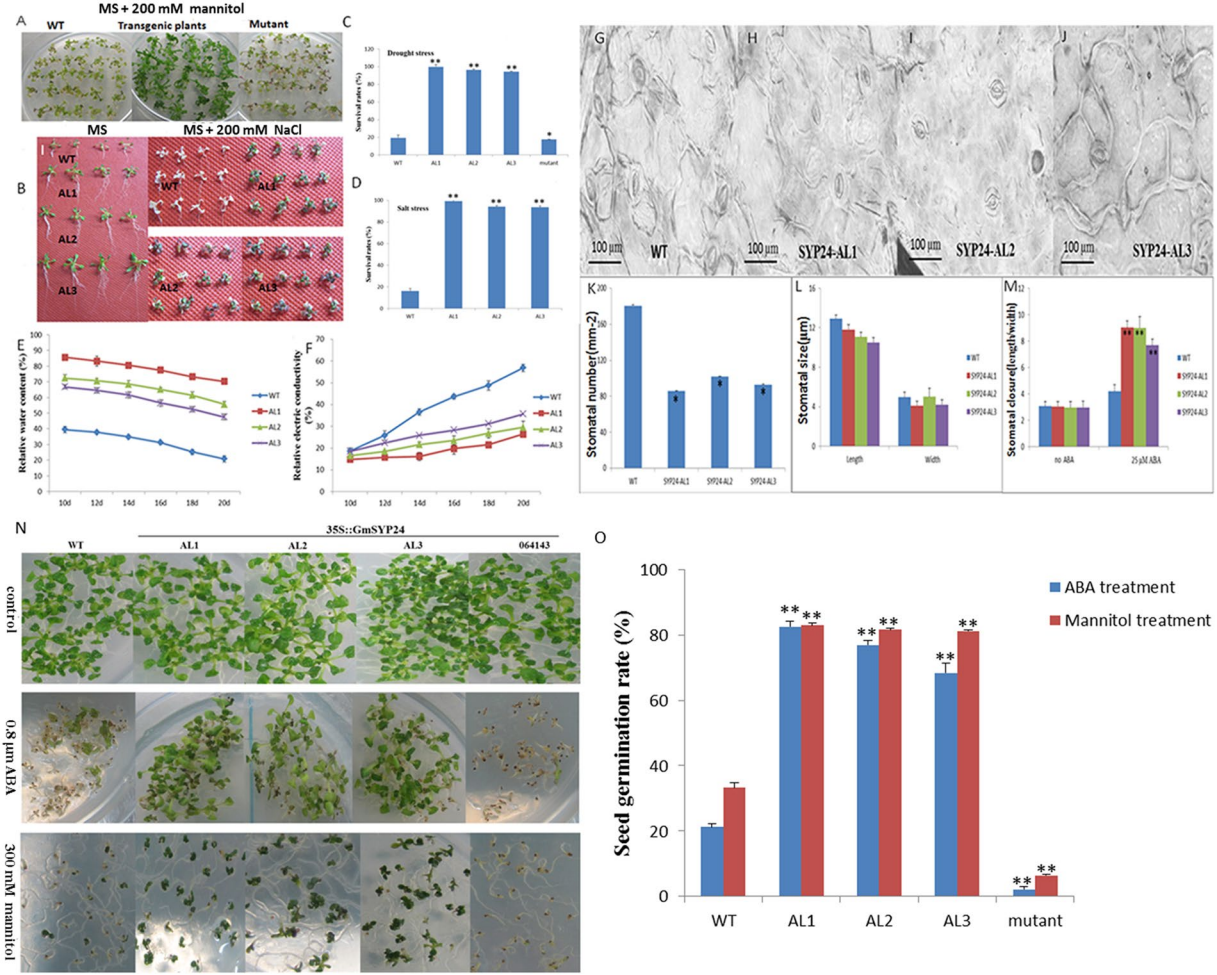
The selection test of drought or salt of transgenic Arabidopsis, wild type and mutant. (**A**,**B**) The phenotype of transgenic and wild type Arabidopsis with 200 mM mannitol/drought or NaCl. Bar = 10 mm. (**C**,**D**) The Survival rates and SE values (error bars) were calculated from the results of three biological replicates. Asterisks indicate significantly higher survival rates than the wild type as determined by Student’s t test (*P < 0. 05; **P < 0.01). (**E**,**F**) The relative water content or electric conductivity of wild type and transgenic lines. (**G**–**M**) The stomatal movement, number, size, and stomatal closure of wild type or *GmSYP24hx* transgenic plants. (**N**) The germination of *GmSYP24*hx, wild type Arabidopsis, and mutant seeds under ABA or mannitol treatment. (**O**) The seed germination rates and SE values (error bars) were calculated from the results of three biological replicates. Asterisks indicate significantly higher survival rates than the wild type as determined by Student’s t test (*P < 0. 05; **P < 0.01).

The leaves of transgenic lines of *GmSYP24*hx were greener after 6-day osmotic/drought or high salinity treatment (Fig. 5A,B). The survival rates of transgenic Arabidopsis reached above 90%, while those of wild type were less than 20% (Fig. 5C,D). In the further study, transgenic plants maintained relatively higher water content in leaf and lower electric conductivity under drought condition (Fig. 5E,F), inferring an association with their intact membrane structure. The stomata were further investigated, which had a close relationship with water content. The length and width of stomata were determined, and the length/width ratio was used as a measurement of stomatal closure^13^. The research found that wild type plants and transgenic lines showed the same length/width ratio of stomata without ABA treatment. But *GmSYP24*hx lines showed a higher length/width ratio of stomata compared with wild type plants after ABA treatment (Fig. 5M). Most of stomata were fully closed, some stomata were semi-opened, and a few were full-opened in transgenic lines. However, the stomata of wild type plants exhibited the contrary morphology. In summary, the stomatal opening in *GmSYP24*hx lines was much less than that in wild type plants after ABA treatment. In addition, we compared the stomatal size by a fluorescence microscope (Fig. 5K,L). There was no significant difference in stomatal size between the transgenic lines and wild type plants. The stomatal density (stomatal number) was distinctly decreased in the *GmSYP24*hx lines (Fig. 5G–J). Taken together, less stomatal density and aperture of transgenic plants under exogenous ABA treatment could reduce water loss, and possibly contribute to drought or high salinity tolerance. It indicated that GmSYP24 might function in stomatal development and closure in ABA mediated abiotic stress response pathway.

### *GmSYP24ox* reduces sensitivity to osmotic/drought or salt stress in soybean seedlings

**Figure 6:**
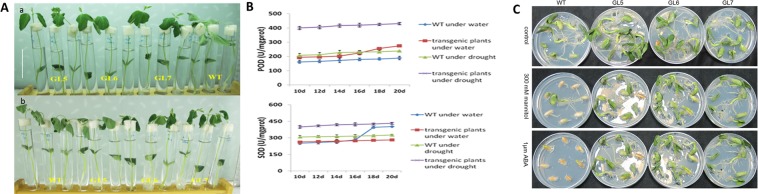
(**A**) The phenotype of *GmSYP24ox* and wild type soybean seedlings into the solution including 300 mM NaCl for 36 h or 25% PEG for 12 h. Bar = 5 cm. (**B**) Measurement of POD and SOD content in transgenic and wild type plants. (**C**) The germination of *GmSYP24*ox and wild type soybean seeds under ABA or mannitol treatment.

Under high salinity stress, the stems of T_2_ transgenic lines were kept erect and their leaves were kept unfold. However, the ones of wild type seedlings showed severe bending (Fig. 6A,a). After PEG treatment, the transgenic plants and wild type had visible wilting, however, GL6 and GL7 still keep erect, and wild type showed bending completely (Fig. 6A,b). In the present study, we measured several stress-responsive physiological indexes to further clarify the underlying mechanism of stress tolerance in the *GmSYP24*ox plants. The results showed that the *GmSYP24ox* plants accumulated greater amounts of POD or SOD content compared with wild type plants under drought condition (Fig. 6B). Nevertheless, there was little difference of POD or SOD between wild type and *GmSYP24*ox plants under non-stress condition.

### *GmSYP24* and its homologous gene in Arabidopsis improves the tolerance to ABA/mannitol treatments during soybean/Arabidopsis seed germination

In the absence of ABA/mannitol, *GmSYP24*hx, wild type and mutant seeds showed identical germination behavior. However, seeds from the *GmSYP24*hx lines germinated much faster than wild type seeds, and the germination of mutant seeds was the slowest in the presence of ABA/mannitol. Furthermore, the transgenic lines showed more open and green leaves than wild type and mutant plants (Fig. 5N). Soybean transgenic lines (SYP24-GL5, -GL6 and -GL7) were also used to study the seed germination sensitivity to ABA**/**mannitol. The results showed that the germination rate of GmSYP24ox seeds was increased with 1 μM ABA treatment and quicker than that of wild type seeds. On the other hand, the *GmSYP24*ox soybean seeds were able to develop healthy cotyledons subsequent to seed coat breakage and radicle emergence (Fig. 6C).

### *GmSYP24* positively regulates the expression of some ABA/stomata-responsive genes in Arabidopsis

**Figure 7:**
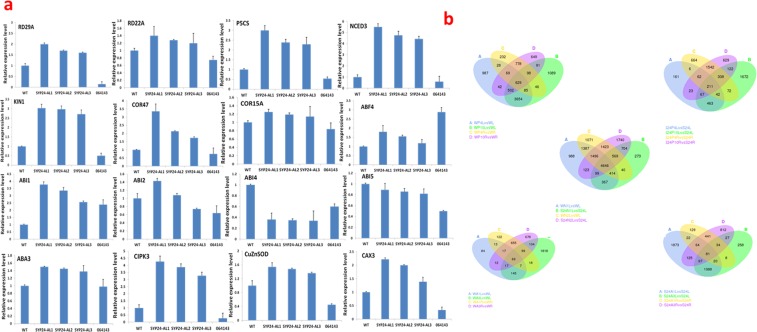
(**a**) The relative expression level of ABA or stomatal-relative genes in *GmSYP24hx* Arabidopsis plants. (**b**) Transcriptome analysis of transgenic and wild type soybean plants. Venn diagrams comparing the differentially expression genes under drought, salt or ABA stress.

In the presence of ABA, all of 13 tested ABA-responsive genes were up-regulated in the transgenic lines, except for *ABI4* and *ABI5*. The expression of most of these genes in mutant plants were decreased compared with wild type plants. Moreover, the expression of the stomatal-responsive genes (CAX3, CIPK3 and Cu/Zn SOD) was also up-regulated in the transgenic lines and decreased in the mutant plants compared with wild type (Fig. 7a). These results indicated that GmSYP24 was an essential protein in ABA signal pathways to increase drought and salt tolerance.

### The up-regulated genes in *GmSYP24-*transgenic soybean show stress-inducible expression

We analyzed expression profiles of transgenic soybean plants under drought, ABA or salt stress by Illumina Hiseq. 4000. The plants without stress treatment were used as controls (Fig. 7b). Due to poor quality of root samples, only the data of leave samples were supplied under high salinity. Compared with the control, 63, 38 and 558 genes were up-regulated under drought, ABA or salt stress, respectively (log2. fold change > 1, and p value < 0.05; Tables S6–S8). Among these genes obtained under drought stress, seven genes encoded TFs and 24 genes encoded enzymes. The seven genes encoding TFs included two genes (Glyma.01G185800 and Glyma.09G190600) encoding heat shock TFs, three genes (Glyma.04G050300, Glyma.07G126800 and Glyma.06G059600) encoding zinc finger TFs, one gene (Glyma.12G117000) encoding ethylene-responsive TF, and one gene (Glyma.17G096700) encoding homeobox-leucine zipper protein. In these genes encoding enzymes, four could catalyze the synthesis of chalcone, asparagine and stachyose with a relatively higher expression level (Table S6). Seven genes involved in regulating transport were identified in the transgenic soybean lines of *GmSYP24ox*. They included two genes encoding WD proteins, two encoding calmodulin-like proteins, one encoding chloride channel protein, one encoding translocator protein and one encoding polyol transporter. Moreover, two genes encoding DIR proteins (dirigent protein 19 and dirigent protein 3) and one encoding proline (PRO) factor were identified, which played an important role in lignin synthesis, biotic and abiotic stresses^14–16^. In addition, eight genes encoding unknown proteins were obtained under drought stress.

Many genes encoding protein kinases were identified in GmSYP24ox transgenic line compared with wild type under salt stress. (Table S7). Two genes encoding WRKY TFs (Glyma.03G109100 and Glyma.13G370100), and four genes encoding MYB proteins (Glyma.05G098200, Glyma.09G169300, Glyma.17G245200 and Glyma.20G117000) were obtained. F-box family genes as key elements in response to salt possibly are helpful in salt tolerance^17^. In this study, we detected four F-box genes (Glyma.06G120600, Glyma.08G216300, Glyma.19G081700 and Glyma.16G197800). Besides, two genes encoding Delta-1-pyrroline-5-carboxylate synthase (Glyma.03G069400 and Glyma.01G099800), four genes encoding Beta-glucosidase Glyma.06G263100, Glyma.15G142400, Glyma.16G039400, Glyma.12G054300) and three LURP genes (Glyma.03G040900, Glyma.09G070200, Glyma.16G013400) were identified.

Under ABA stress, we detected some photosynthesis-related genes, such as genes encoding chlorophyll binding proteins (Glyma.01G115900 and Glyma.16G205200), plastocyanin (Glyma.04G020300) and ferredoxin (Glyma.05G168400). Four genes encoding snakin proteins (Glyma.17G237100, Glyma.14G087200, Glyma.06G185300, and Glyma.04G179500) had been identified, and they were annotated as gibberellin-regulated genes according to pfam description in the soybean database (https://phytozome.jgi.doe.gov/). There were some genes encoding two WRKYs (Glyma.09G005700 and Glyma.15G110300.Wm82) and single bHLH130 involved of *GmSYP24ox* lines under ABA. Besides, two genes encoding structural proteins (Glyma.04G118700 and Glyma.08G200100) and five genes encoding unknown proteins were also obtained (Table S8).

### *GmSYP24*ox alters the expression of *aquaporins* under osmotic/drought, salt or ABA condition

We found that the expression level of some *aquaporins*, such as *PIPs*, *TIPs*, *NIPs*, and *SIPs*, were altered in the *GmSYP24*ox plants rather than wild type under any one of three stresses (Table S9). Under salt stress, seven *PIPs*, 12 *TIPs* and single *NIP* protein were obtained. Most of these *aquaporins*’ expression was decreased. We detected 9 *PIPs*, six *TIPs*, and one *NIP* in the leave of *GmSYP24*ox plants under osmotic stress. The expression of the *aquaporins* was up-regulated after the treatment of 4 h, and some negatively expressed genes appeared after 10 h. However, the expression of some *aquaporins* identified in root mainly was suppressed. Under ABA treatment, 14 *PIPs*, six *TIPs* and single *SIP* were identified in the leave, and the expression level of all the *aquaporins* increased. There were 13 *aquaporins* detected in the root, and most were down-regulated proteins of GmSYP24.

## Discussion

The proteins containing LEA domains belong to a large protein family. Some studies have indicated that these proteins can be divided into several subfamilies according to sequence similarity^18–20^. Most of LEA-containing proteins are hydrophilic proteins. However, LEA2 subgroup has been reported as hydrophobic protein or atypical protein^21,22^. It remains unclear about the structure and function of LEA2-containing proteins. In the present study, we analyzed the gene structure, chromosomal distribution and evolutionary relationship of the proteins containing LEA2 domain (Figs 1–3). Syntaxin proteins harboring LEA2 domain were located at the end branch of evolutionary tree, speculating that these syntaxin proteins appeared through a long evolutionary history from this large family of LEA2-containing proteins by several gene duplications, such as segmental and tandem duplication.

The cell wall is the first protection barrier of plants against environmental stresses. Lignin, surrounding cellulose cells and sclerenchymatous tissue, is a polymer which is formed by random polymerization of phenyl propane units. It is filled with the cellulose and can enhance mechanical strength of plants to water transport and resist diseases and abiotic stresses^23^. Here, DIR proteins were identified in transgenic lines under drought stress, which have been reported to involve in lignin synthesis^24,25^. They can be induced by pathogen defense and abiotic stresses^15,16,26^. In this research, GmSYP24 is a membrane protein by subcellular localization. Therefore, we speculated that the protein may respond to abiotic stress by participating in membrane system activity. The proteins involved in regulating transport, such as aquaporins, ion channel proteins and calmodulin-binding proteins were identified under drought, salt or ABA condition in the *GmSYP24*ox transgenic plants (Table S6–S9). These related proteins located on the membrane possibly form signal transduction network to adapt for adverse environment, when plants subject to drought, salt or other stresses^27,28^.

The stomata on the leaf surface are important multicellular complex. The stomatal movement affects many crucial physiological processes, such as photosynthesis and response to environmental stresses. The stoma is composed of series of guard cells containing chloroplasts^29^. Some studies have indicated that chloroplasts in the guard cells may be involved in regulating stomatal movement by converting starch or carbon degradation into osmolytes or providing ATP on the plasma membrane H^+^-ATPase to actuate K^+^ influx^30^. The stomatal opening of an Arabidopsis mutant without chloroplasts in guard cells is reduced compared with wild type. A recent study has shown that the interaction between HCF106 and THF1 proteins in the chloroplast and the mutants can drive stoma closure^31^. In our study, the syntaxin protein GmSYP24 was located on the cell membrane, and *GmSYP24hx* in Arabidopsis changed stomatal movement under drought. In addition, we found that *GmSYP24ox* in soybean transgenic lines promoted the expressions of some chloroplast proteins under ABA stress (Table S8). These results showed that *GmSYP24* was the upstream gene of some chloroplast genes to regulate stomatal aperture by ABA signal transduction pathway under drought stress.

Transcription factors are crucial regulatory proteins in response to abiotic stresses. Early research has shown that homeobox-leucine zipper proteins are developmental regulators and they confer drought or salt tolerance to plants^32,33^. In soybean, the heat shock transcription factor (HSF) family has been studied by genome-wide analysis, and the overexpression of *Gmhsf34* gene can improve the tolerance to drought and heat stresses in Arabidopsis^34^. Ethylene-responsive transcription factors (ERFs) are also involved in biotic or abiotic stresses. The ERF1 from wheat mediates host responses to both pathogen and freezing stresses, and LchERF-overexpressing plants show higher chlorophyll and PRO contents as well as lower H_2_O_2_ content under salt stress^35,36^. Recent studies have indicated that CCCH zinc finger proteins are associated with senescence delaying effect, and they can interact with ABA and drought response regulators^37,38^. WRKY TFs are also important factors in response to ABA stress^39^. The *WRKY20* gene of *Glycine soja* has been identified to improve drought or high salinity tolerance in transgenic alfalfa^40^^.^ In Arabidopsis, bHLH122 has been identified to function against drought or osmotic stress and in repression of ABA catabolism^41^. We detected HSF, ERF, HD-ZIP and three zinc finger proteins under drought stress, and one bHLH protein and two WRKYs under ABA treatment in the *GmSYP24ox* plants. Some proteins in these TFs families have been reported to be involved in drought, salt or ABA stress. We speculate that these TFs identified in our experiment may be important proteins in response to drought or ABA stress.

In conclusion, our study found that *GmSYP24ox* in soybean and GmSYP24hx in Arabidopsis made transgenic plants insensitivity to drought or salt stress, and improved transgenic seeds tolerance to ABA. It indicated that soybean syntaxin protein GmSYP24 might regulate plant several abiotic stresses tolerance and seed germination through ABA signaling pathway.

## Methods

### Identification of genes harboring LEA2 domain in soybean

Key words “pfam 03168 and (or) cl12118” of LEA2 domain were used to search soybean and Arabidopsis LEA2 subfamily genes in the National Center for Biological Information (NCBI; http://blast.ncbi.nlm.nih.gov/ Blast), Phytozome (v10.2) database (http://www.phytozome.net) and the Arabidopsis Information Resource (Tair, http://www.arabidopsis.org/). These searched putative LEA2 superfamily genes were confirmed by using InterPro (https://www.ebi.ac.uk/ interpro/) database.

### Gene structure analysis and chromosomal distribution

Genomic DNA and cDNA sequences of predicted soybean syntaxin genes were downloaded from Phytozome10.2 database. Their exon/intron structures were analyzed by the gene structure display server program^42^. The chromosomal location was generated by Chromosome Visualization Tool (CViT) at the Legume Information System^43^. The presence of the syntaxin genes in segmental and tandem duplication blocks was investigated using CViT and web-based Synteny Viewer as previously described^10^.

### Phylogenetic tree construction of syntaxin proteins containing LEA2 domain in soybean and Arabidopsis

Phylogenetic tree was constructed using MEGA 6.0 software^44^. Clustal W was used to process multiple alignments of amino acid sequences containing the conserved LEA2 domain of soybean and Arabidopsis. A phylogenetic tree was constructed by the neighbor-joining method with the Poisson correction, pairwise deletion and bootstrap analysis with 1,000 replicates.

### Screening and cloning analysis of *GmSYP24* gene from soybean

To identify dehydration/drought stress responsive genes, we have previously performed a DGE (digital gene expression profile) analysis between two soybean materials and discovered some DEGs (differentially expressed genes) between these varieties^45^. Among them, we searched 18 syntaxin containing LEA2 domain and finally focused on a syntaxin gene (named *GmSYP24*). The full-length CDS of *GmSYP24* was obtained according to the database (http://phytozome.jgi.doe.gov/pz/portal.html). The sequence of *GmSYP24* gene including complete ORF was respectively amplified from two soybean materials. The PCR products were cloned into pMD-18T vector, transformed into *Ecoli* DH5α and then sequenced (Invitrogen, Shanghai, China). The cDNA sequence containing the full-length coding sequence of *GmSYP24* was amplified by PCR using the primers SYP24-F1 and SYP24-R1 (Table S10).

### Plant materials and transformation methods

Soybean drought-tolerant cultivar *Jindou21* and drought-sensitive cultivar *Zhongdou33* were selected for gene amplification and expression analysis. *Tianlong1* (a soybean genotype suitable for soybean transformation, more sensitive to drought stress than *Jindou21* (Fig. S, S1)) and Arabidopsis ecotype Columbia (Arabidopsis thaliana) were used in genetic transformation. Soybean transgenic plants were obtained by *Agrobacterium tumefacien*-mediated cotyledonary node transformation with several modifications^1^. Arabidopsis transgenic plants were obtained by floral dip method^46^.

### Acquirement of *GmSYP24* transgenic plants and the Arabidopsis mutant selection of *GmSYP24* homologous genes

The PCR products were cloned into pB2GW7 expression vector and pCXSN expression vector for soybean and Arabidopsis transformation, respectively (Fig. S, S2-A,B). The resulting constructs were confirmed by sequencing and then transferred into the agrobacterium EHA105 and GV3101 respectively. The three lines of transgenic soybean (GL5, GL6 and GL7) and those of Arabidopsis (AL1, AL2 and AL3) were from T_0_ multiple independent alleles respectively (Fig. S, S3). The expression level of *GmSYP24* in the transgenic lines was detected by SYP24-F_2_ and SYP24-R_2_ (Table S10). Positive transgenic plants were tested by PCR and bar protein detection (Fig. S, S2-C, and -D). Mutants of four homologous genes (AT2G35980, AT2G35460, AT3G11650 and AT5G06320) of *GmSYP24* were obtained from TAIR database (https://www.arabidopsis.org/). The primers for identification of mutants were designed using http://signal.salk.edu/tdnaprimers.2.html”.

### Plant growth conditions and stress treatments

Soybean drought-tolerant and drought-sensitive cultivars were grown until the first trifoliolate leaf was unfolded. Soybean transgenic seedlings were grown indoors at 28 °C under 16-h-light/8-h-dark cycle at a photon flux density of 120 µmol m^–2^ s^–1^. Arabidopsis thaliana seeds were grown on MS^16^ plates (0.8% agar, 3% sucrose, pH 5.8) for 10 days (22 °C, 16-h-light/8-h-dark cycle, 80 mmol m^–2^ s^–1^ photon flux density) before stress treatments.

### Drought or salt treatment of transgenic and wild type soybean

The osmotic stress was a course of rapid water loss to simulate drought. Transgenic and wild type soybean seeds were germinated in water-soaked paper until the first trifoliolate leaf was unfolded. They were transferred into the solution with 25% PEG for 12 h. The phenotypes of transgenic lines and wild type were investigated and photographed. Soybean seedlings for salt stress were grown in 300 mM NaCl solution for 36 h under the same conditions of osmotic stress.

### Drought or salt treatment of transgenic and wild type Arabidopsis

Transgenic and wild type Arabidopsis seeds were grown on 0.5 × MS agar plates for 10 days. When the sixth rosette leaf was generated, one part was transferred into 0.5 × MS agar plates in the absence or presence of 200 mM mannitol until visual symptoms were observed. For the salt stress, the seedlings were cultured on 0.5 × MS agar plates in the absence or presence of 200 mM NaCl for indicated time periods as previously described until visual symptoms were observed^47^.

### Gene expression analysis

Total RNA was extracted from soybean drought-tolerant and drought-sensitive cultivars and transgenic plants of soybean or Arabidopsis using the Trizol reagent (Invitrogen, USA). The DNase-treated RNA was reversely transcribed into cDNA using Prime Script RT reagent kit with gDNA Eraser (Takara, China) according to the manufacturer’s instructions. Quantitative real-time PCR (qRT-PCR) was performed in 96-well plates on a Bio-Rad iQ5 Real-Time PCR Detection system using SuperReal PreMix (SYBR Green) reagents (Tiangen, China). Reactions were carried out referring to^48^. The actin gene of soybean and Arabidopsis was selected as the housekeeping gene. Cycle threshold (ct) values were standardized for each template based on reference gene, and the 2^–ΔΔCT^ method was used to analyze relative gene expressions^49^. Three replicate reactions per sample were used to ensure statistical credibility. Table S10 lists the gene-specific qRT-PCR primers used in this study.

### Microarray experiment

Transgenic line GL7 and wild type soybean seeds were germinated in water-soaked paper until the first trifoliolate leaf was unfolded. Illumina HiSeq was used for genome-wide expression analysis using transgenic lines and wild type soybean under drought (25% PEG for 4 h and 10 h), salt (300 mM NaCl for 1d and 2d), or ABA stress (for 1 h and 3 h). Three biological replicates were used to ensure statistical credibility. High-throughput sequencing was performed by Novogene, Beijing^50–52^. WL or WR represents the gene expression in the leaf or root of wild type under normal condition; WA1L(WA3L) and WA1R (WA3R) represents the gene expression in the leaf or root of wild type after 1 h or 3 h of ABA treatment, respectively; S24L or S24R represents the gene expression in the leaf or root of *GmSYP24*ox plants under normal condition; S24A1L(S24A1R) and S24A3L(S24A3R) represents the gene expression in the leaf or root of GmSYP24ox plants after 1 h or 3 h of ABA treatment, respectively; WP4L(WP4R) and WP10L(WP10R) represents the gene expression in the leaf or root of wild type after 4 h or 10 h of PEG6000 treatment, respectively; S24P4L(S24P4R) and S24P10L(S24P10R) represent the gene expression in the leaf or root of *GmSYP24*ox plants after 4 h or 10 h of PEG6000 treatment, respectively. WN1L and WN2L represent the gene expression in the leaf of wild type after 1 d or 2 d of 300 mM NaCl treatment, respectively. S24N1L and S24N2L represent the gene expression in the leaf of *GmSYP24*ox plants after 1 d or 2 d of 300 mM NaCl treatment, respectively.

### Sub-cellular localization of GmSYP24-GFP fusion proteins

The whole coding sequence of *GmSYP24* was amplified with two primers SYP24-F3 and SYP24-R3 (Table S10). The PCR product was sub-cloned into PJG053 vector under the control of CaMV 35 S promoter. The recombinant construct was confirmed by sequencing and used for transient transformation of onion (Allium cepa) and tobacco epidermis via a gene gun (Bio-Rad, USA) and the infection by Agrobacterium tumefaciens respectively. Transient expressions of 35 S:: GmSYP24-GFP and 35 S::GFP as a control in onion epidermal cells were performed according to a previously described method^53^. Transformed onion or tobacco cells were observed under a confocal microscope (Nikon, Japan).

### Germination assay and growth inhibition assay

#### ABA and mannitol treatments of transgenic soybean seeds

The seeds of wild type and T_3_ transgenic soybean lines were surfaced-sterilized and then sown on B_5_ agar medium with 1 μM ABA or 300 mM mannitol. The germination and growth of seeds were investigated accordingly.

#### ABA and mannitol treatments of transgenic Arabidopsis seeds

For the germination, the seeds from the wild type, mutant and T_3_ generation transgenic homozygous Arabidopsis were surfaced-sterilized and sown on 0.5 × MS agar medium with 0.8 μM ABA or 300 mM mannitol^54^.

### Measurement of stress-responsive physiological indexes in transgenic and wild type soybean plants

RWC during long-term drought stress was determined using a previously described method^55^. Relative electrical conductivity (REC) analysis was conducted using a previously described method with minor modifications^56^. Activities of superoxide dismutase (SOD) and peroxidase (POD) were determined by colorimetry.

### Accession numbers of ABA/stomatal-responsive genes

*RD29A* (AT5G52310), *RD22* (AT5G25610), *NCED3* (At3g14440), *COR47* (At1g20440), *ABI1* (AT4G26080), *ABI2* (AT5G57050), *ABI4* (AT2G40220), *ABI5* (AT2G36270), *COR15A* (AT2G42540), *KIN1* (AT5G15960), *P5CS* (AT2G39800), *ABF4* (AT3G19290), *Cu/Zn SOD* (AT1G08830), *CIPK3* (AT2G26980), *ABA3* (AT1G16540), *CAX3* (AT3G51860)

## Supplementary information

Supporting Information

Table S7
