# Supplementary material for: GmSYP24, a putative syntaxin gene, confers osmotic/drought, salt stress tolerances and ABA signal pathway
Source: Sci Rep. 2019 Apr 12;9:5990. doi: 10.1038/s41598-019-42332-5 (PMC6461667; doi:10.1038/s41598-019-42332-5)
Supplement: Supplementary file 1 — Supporting Information [file 41598_2019_42332_MOESM1_ESM.pdf]

GmSYP24, a putative syntaxin gene, confers osmotic/drought, salt stress tolerances and ABA signal pathway

Li-Miao Chen[1,2], Yi-Sheng Fang[1,2], Chan-Juan Zhang[1,2], Qing-Nan Hao[1,2], Dong Cao[1,2], Song-Li Yuan[1,2], Hai-Feng Chen[1,2], Zhong-Lu Yang[1,2], Shui-Lian Chen[1,2], Zhi-HuiShan[1,2], Bao-Hong Liu[1,2], Jing-Wang[1,2], Zhan Yong[5], Xiao-Juan Zhang[1,2], De-Zhen Qiu[1,2], Wen-Bin Li[3,4]*, Xin-An Zhou[1,2]*

[1]Key Laboratory of Oil Crop Biology, Ministry of Agriculture, Wuhan, China 430062

[2]Oil Crops Research Institute of Chinese Academy of Agriculture Sciences, China 430062

[3]Key Laboratory of Soybean Biology in the Chinese Ministry of Education, Northeast Agricultural University, Harbin, China 150030

[4]Division of Soybean Breeding and Seed, Soybean Research & Development Center, CARS (Key Laboratory of Biology and Genetics & Breeding for Soybean in Northeast China, Ministry of Agriculture), China 150030

[5]Crop Research Institute, Xinjiang Academy of Agricultural and Reclamation Science，Key Lab of Cereal Quality Research and Genetic Improvement, Xinjiang Production and Construction Crops，832000, China

***Corresponding authors:**

Xin-An Zhou; Wen-Bin Li

Xin-An Zhou

Oil Crops Research Institute, Chinese Academy of Agriculture Sciences, Xudong 2nd Road, Wuhan, China 430062

Phone/FAX: 86-027-86711563

Email: zhouocri@sina.com

Wen-Bin Li

Northeast Agricultural University, Key Laboratory of Soybean Biology in the Chinese Ministry of Education, 600 Changjiang Road, Harbin, China 150030

Phone/ FAX: 86-0451-55190778

Email: wenbinli@yahoo.com

Supporting Information

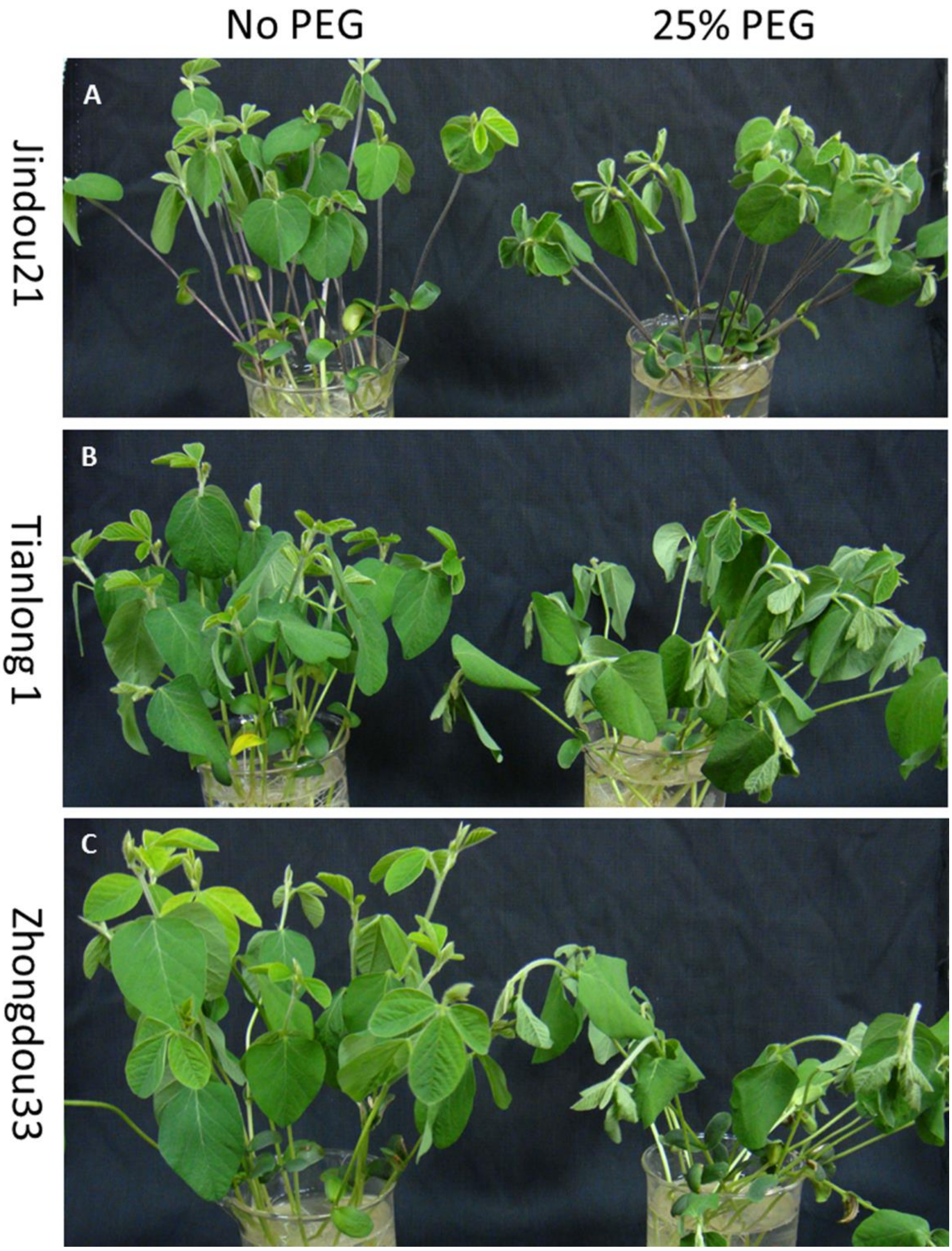

Figure S1. The phenotype of Jindou21, Tianlong1 and Zhongdou33 under 25% PEG or not. (A) Jindou21, (B) Tianlong1, (C) Zhongdou33.

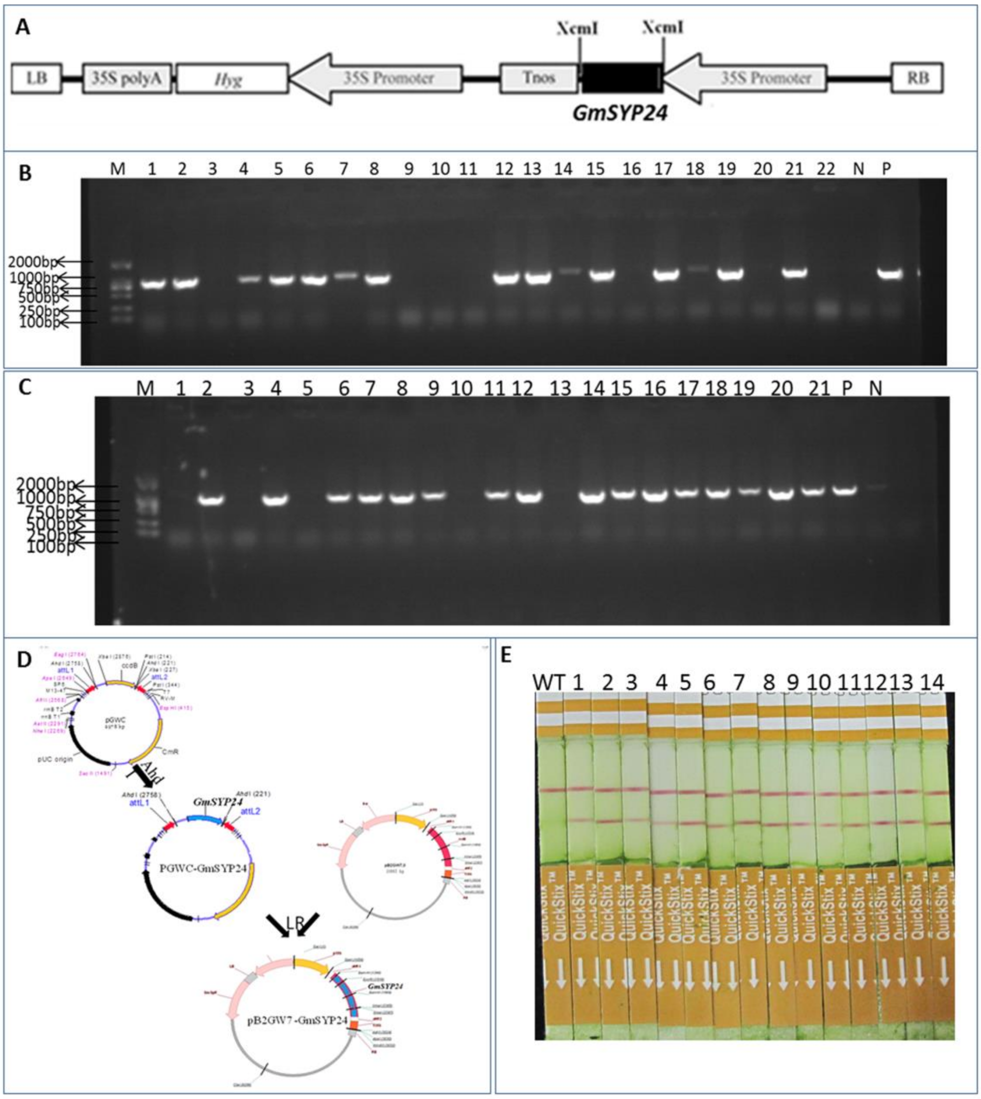

Figure S2. The construction of plant expression vector and molecular detection of transgenic plants. (A) The construct of *GmSYP24* into the pCXSN vector to transform Arabidopsis. (B) The amplification of *GmSYP24* in transgenic Arabidopsis plants. M: 2000 maker; 1-22: transgenic plants; N: negative control; P: positive control. The length of amplified fragment is 775bp (including CDS full-length of *GmSYP24*). (C) The amplification of *GmSYP24* in transgenic soybean plants. M: 2000 maker; 1-21: transgenic plants; P: positive control; N: negative control. The length of amplified fragment is 775bp (including CDS full-length of *GmSYP24*). (D) The construct of *GmSYP24* into the pB2GW7 vector to transform soybean. (E) The Bar protein detection in transgenic soybean of *GmSYP24ox*. WT: wild type plants (negative control); 1-14: transgenic positive plants.

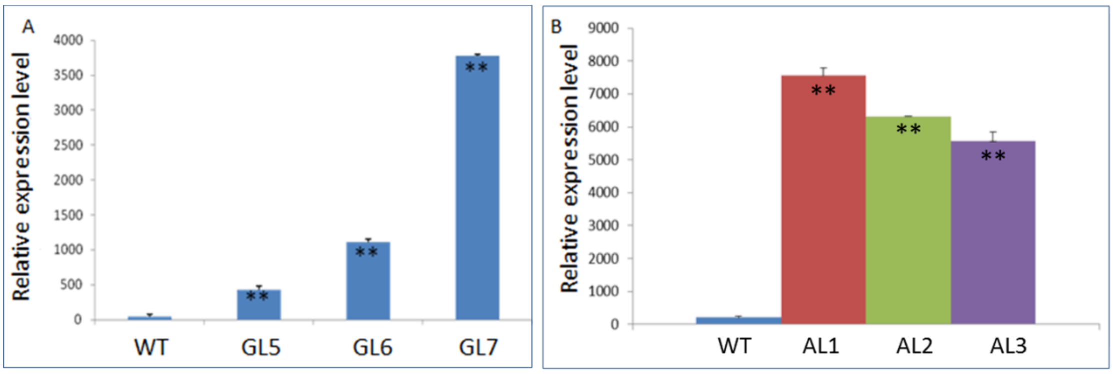

Figure S3. The expression analysis of *GmSYP24* in wild type and three transgenic lines of soybean (A) and Arabidopsis (B). Total RNA was isolated from the leave of greenhouse grown plants. The keephouse gene actin was employed as an internal control. The expression level of these genes in the leave of WT was set to 1. Error bars represent the SDs from three biological replicates. ** indicate significant differences in comparison to WT at $P < 0.01$.

Table S1. LEA2 family genes of soybean were list.

Gene name	Locus name (version Glyma2.0)	the number of LEA2 domain	GenBank Accession No.	Genome Location	The annotation of Arabidopsis homologous genes	Peptide Length	genome sequence	pI	Mw (KD)
GmLEA2-1	Glyma.01G152600	1	XM_003516456	Chr01:48960693-48961382	AT5G45320.1(molecular_function unknown)	229	690	8.95	25.82
GmLEA2-2	Glyma.01G222800	1	XM_006573731	Chr01:55174239-55175109	AT1G64065.1(Late embryogenesis abundant (LEA) hydroxyproline-rich glycoprotein family)	184	871	10.24	20.34
GmLEA2-3	Glyma.01G226100	1		Chr01:55470092-55471377	AT1G64450.1(Glycine-rich protein family)	209	1286	9.51	23.09
GmLEA2-4	Glyma.01G240600	1		Chr01:56444294-56444830	AT4G05220.1(Late embryogenesis abundant (LEA) hydroxyproline-rich glycoprotein family)	178	537	5.2	19.90
GmLEA2-5	Glyma.02G197600	1	XM_003519073	Chr02:37639021-37640558	AT3G11660.1(NDR1/HIN1-like 1)	207	1538	9.79	23.92
GmLEA2-6	Glyma.02G197800	1	XM_003519074	Chr02:37686736-37687986	AT3G11650.1(NDR1/HIN1-like 2, SYNTAXIN)	244	1251	9.26	27.63
GmLEA2-7	Glyma.02G239100	1	NM_001252948	Chr02:42754798-42755899	AT1G65690.1(Late embryogenesis abundant (LEA) hydroxyproline-rich glycoprotein family)	255	1102	10.12	28.77
GmLEA2-8	Glyma.02G274400	1	XM_003519436	Chr02:45751520-45753085	AT2G27080.1(Late embryogenesis abundant (LEA) hydroxyproline-rich glycoprotein family)	273	1566	9.81	30.50
GmLEA2-9	Glyma.02G277300	2	XM_006574374	Chr02:46010712-46013329	AT2G44060.1(Late embryogenesis abundant protein, group 2)	321	2618	4.85	35.82

GmLEA2-10	Glyma.02G303100	1		Chr02:47852657-47854573	AT4G26490.1(Late embryogenesis abundant (LEA) hydroxyproline-rich glycoprotein family)	236	1917	9.96	26.70
GmLEA2-11	Glyma.03G000800	1	XM_003521561	Chr03:113864-115495	AT3G54200.1（Late embryogenesis abundant (LEA) hydroxyproline-rich glycoprotein family）	192	1632	9.17	20.47
GmLEA2-12	Glyma.03G201000	1	XM_003521442	Chr03:40968934-40970257	AT2G35980.1（Late embryogenesis abundant (LEA) hydroxyproline-rich glycoprotein family，syntaxin）	221	1324	9.58	24.94
GmLEA2-13	Glyma.03G201100	1	NM_001253965	Chr03:40972049-40972976	AT2G35980.1(Late embryogenesis abundant (LEA) hydroxyproline-rich glycoprotein family,SYNTAXIN)	227	928	9.31	26.34
GmLEA2-14	Glyma.03G201200	1	XM_003520665	Chr03:40977183-40977806	AT2G35980.1(Late embryogenesis abundant (LEA) hydroxyproline-rich glycoprotein family,SYNTAXIN)	207	624	9.46	24.14
GmLEA2-15	Glyma.03G201300	1		Chr03:40980745-40990121	AT2G35980.1(Late embryogenesis abundant (LEA) hydroxyproline-rich glycoprotein family,SYNTAXIN)	243	9377	9.22	26.77
GmLEA2-16	Glyma.03G201500	1	XM_003521445	Chr03:40986155-40987233	AT2G35980.1(Late embryogenesis abundant (LEA) hydroxyproline-rich glycoprotein family,SYNTAXIN)	229	1079	9.92	28.67
GmLEA2-17	Glyma.03G201600	1	XM_006577032	Chr03:40993013-40994536	AT3G11660.1（NDR1/HIN1-like 1)	252	1524	9.83	21.56
GmLEA2-18	Glyma.03G261400	1	XM_003521778	Chr03:45488450-45489586	AT4G01410.1(Late embryogenesis abundant (LEA) hydroxyproline-rich glycoprotein family)	197	1137	9.66	27.86
GmLEA2-19	Glyma.03G263800	1		Chr03:45666942-45667919	AT2G01080.1 (Late embryogenesis	185	978	8.88	21.09

					abundant (LEA) hydroxyproline-rich glycoprotein family)				
GmLEA2-20	Glyma.04G051600	1	XM_003522201	Chr04:4179782-4181288	AT5G11890.1(molecular_function unknown)	256	1507	9.87	28.23
GmLEA2-21	Glyma.04G103700	1	XM_003523778	Chr04:9701112-9702592	AT2G27080.1(Late embryogenesis abundant (LEA) hydroxyproline-rich glycoprotein family)	255	1481	9.51	28.41
GmLEA2-22	Glyma.04G228200	1		Chr04:49753281-49754534	AT5G53730.1(Late embryogenesis abundant (LEA) hydroxyproline-rich glycoprotein family)	210	1254	9.69	23.26
GmLEA2-23	Glyma.05G094300	1	XM_003525763	Chr05:23964664-23966589	AT1G45688.1(molecular_function unknown)	324	1926	10.06	35.72
GmLEA2-24	Glyma.05G179200	1	XM_006580216	Chr05:36725007-36725747	AT2G27080.1(Late embryogenesis abundant (LEA) hydroxyproline-rich glycoprotein family)	246	741	8.82	26.90
GmLEA2-25	Glyma.05G212600	1	XM_003525320	Chr05:39412222-39413348	AT2G46150.1(Late embryogenesis abundant (LEA) hydroxyproline-rich glycoprotein family)	224	1127	9.28	24.80
GmLEA2-26	Glyma.05G217500	1	XM_003525294	Chr05:39757555-39758761	AT1G64450.1(Glycine-rich protein family)	213	1207	9.54	22.97
GmLEA2-27	Glyma.05G242600	1	XM_003525161	Chr05:41753113- 41754953	AT1G45688.1(molecular_function unknown)	294	1841	9.98	32.78
GmLEA2-28	Glyma.06G052400	1	XM_003525960	Chr06:3947109-3948444	AT5G11890.1(molecular_function unknown)	256	1336	9.87	28.11
GmLEA2-29	Glyma.06G104900	1	XM_003527865	Chr06:8383654-8384433	AT2G27080.1（Late embryogenesis abundant (LEA) hydroxyproline-rich	259	780	9.04	28.59

					glycoprotein family ）				
GmLEA2-30	Glyma.06G136700	1	XM_006580863	Chr06:11191691-11193555	AT5G53730.1（ Late embryogenesis abundant（LEA）hydroxyproline-rich glycoprotein family ）	223	1865	9.1	25.41
GmLEA2-31	Glyma.06G303300	1		Chr06:49275774-49276891	AT3G54200.1（ Late embryogenesis abundant（LEA）hydroxyproline-rich glycoprotein family ）	227	1118	9.65	25.10
GmLEA2-32	Glyma.07G009700	1	XM_006582950	Chr07:728460-730026	AT1G17620.1（ Late embryogenesis abundant（LEA）hydroxyproline-rich glycoprotein family ）	254	1567	9.68	28.02
GmLEA2-33	Glyma.07G049300	1		Chr07:4196836-4197585	AT4G01410.1（ Late embryogenesis abundant（LEA）hydroxyproline-rich glycoprotein family ）	249	750	9.56	27.80
GmLEA2-34	Glyma.07G084600	1	XM_003528883	Chr07:7793897-7796474	AT1G45688.1(molecular_function unknown)	308	2578	9.51	33.71
GmLEA2-35	Glyma.07G087000	1		Chr07:8053652-8055911	AT4G13270.1（ Late embryogenesis abundant（LEA）hydroxyproline-rich glycoprotein family ）	163	2260	9.07	18.36
GmLEA2-36	Glyma.07G099500	1	XM_003528914	Chr07:9419717-9422235	AT1G45688.1(molecular_function unknown)	297	2519	9.93	32.80
GmLEA2-37	Glyma.07G103700	1	XM_003528941	Chr07:9969632-9970849	AT2G35980.1（Late embryogenesis abundant（LEA）hydroxyproline-rich glycoprotein family，syntaxin）	205	1218	9.67	23.52
GmLEA2-38	Glyma.07G103800	1	XM_006583406	Chr07:9985288-9986135	AT2G35980.1(Late embryogenesis abundant（LEA）hydroxyproline-rich glycoprotein family,SYNTAXIN)	209	848	9.43	24.11

GmLEA2-39	Glyma.07G103900	1	XM_003530019	Chr07:9990878-9991757	AT2G35980.1(Late embryogenesis abundant (LEA) hydroxyproline-rich glycoprotein family,SYNTAXIN)	207	880	9.8	24.12
GmLEA2-40	Glyma.08G019000	1	NM_001249306	Chr08:1549130-1550331	AT2G46150.1(Late embryogenesis abundant (LEA) hydroxyproline-rich glycoprotein family)	226	1202	8.98	24.72
GmLEA2-41	Glyma.08G023500	1	XM_003532388	Chr08:1873670-1874780	AT1G64450.1(Glycine-rich protein family)	209	1111	9.54	22.50
GmLEA2-42	Glyma.08G077900	1	XM_003532583	Chr08:5920205-5925896	AT2G01080.1(Late embryogenesis abundant (LEA) hydroxyproline-rich glycoprotein family)	226	5692	9.38	24.70
GmLEA2-43	Glyma.08G136900	1	XM_006586319	Chr08:10465850-10466602	AT2G27080.1(Late embryogenesis abundant (LEA) hydroxyproline-rich glycoprotein family)	250	753	9.07	27.08
GmLEA2-44	Glyma.08G184000	1	XM_003532927	Chr08:14749219-14752814	AT2G01080.1(Late embryogenesis abundant (LEA) hydroxyproline-rich glycoprotein family)	253	3596	9.66	27.33
GmLEA2-45	Glyma.09G071900	1	XM_003534908	Chr09:7418618-7419205	AT3G54200.1(Late embryogenesis abundant (LEA) hydroxyproline-rich glycoprotein family)	195	588	9.21	21.27
GmLEA2-46	Glyma.09G179400	1	XM_006587421	Chr09:40412865-40415233	AT1G45688.1(molecular_function unknown)	297	2369	10.06	32.82
GmLEA2-47	Glyma.09G189900	1	XM_003534146	Chr09:41452904-41455957	AT4G13270.1(Late embryogenesis abundant (LEA) hydroxyproline-rich glycoprotein family)	257	3054	6.73	28.81
GmLEA2-48	Glyma.09G192300	1	XM_003534159	Chr09:41692753-41695528	AT1G45688.1(molecular_function	314	2776	9.82	34.58

					unknown)				
GmLEA2-49	Glyma.09G217800	1	XM_003533428	Chr09:44083696-44084349	AT5G45320.1(molecular_function unknown)	217	654	9.97	24.37
GmLEA2-50	Glyma.10G079400	1	XM_003537055	Chr10:9086412-9087146	AT3G11650.1(NDR1/HIN1-like 2 ,syntaxin)	244	735	9.09	27.57
GmLEA2-51	Glyma.10G079900	1	XM_003535760	Chr10:9245085-9246282	AT2G35980.1(Late embryogenesis abundant (LEA) hydroxyproline-rich glycoprotein family,SYNTAXIN)	227	1198	9.64	26.25
GmLEA2-52	Glyma.10G080000	1	XM_003535761	Chr10:9258476-9260697	AT3G11660.1(NDR1/HIN1-like 1)	209	2222	9.64	26.25
GmLEA2-53	Glyma.10G174100	1	XM_003536133	Chr10:40785837-40786595	AT5G36970.1(NDR1/HIN1-like 25)	252	759	10.02	24.00
GmLEA2-54	Glyma.10G252500	1	XM_003536512	Chr10:48034926-48035710	AT4G05220.1(Late embryogenesis abundant (LEA) hydroxyproline-rich glycoprotein family)	227	785	9.56	26.04
GmLEA2-55	Glyma.11G003000	1		Chr11:188906-189478	AT3G20600.1(Late embryogenesis abundant (LEA) hydroxyproline-rich glycoprotein family)	190	573	9.71	21.64
GmLEA2-56	Glyma.11G020500	1		Chr11:1450017-1450631	AT1G64065.1(Late embryogenesis abundant (LEA) hydroxyproline-rich glycoprotein family)	204	615	9.74	22.98
GmLEA2-57	Glyma.11G020600	1	XM_006591461	Chr11:1453020-1453553	AT1G64065.1(Late embryogenesis abundant (LEA) hydroxyproline-rich glycoprotein family)	177	534	9.87	19.66
GmLEA2-58	Glyma.11G097100	1	XM_003537704	Chr11:7411891-7415860	AT2G41990.1(molecular_function unknown)	321	3970	9.63	36.32
GmLEA2-59	Glyma.11G179900	1	XM_003538046	Chr11:24548259-24549398	AT3G44220.1(Late embryogenesis abundant (LEA) hydroxyproline-rich	214	1140	9.96	23.59

					glycoprotein family)				
GmLEA2-60	Glyma.11G182400	1	XM_003538062	Chr11:24988921-24993379	AT3G44380.1(Late embryogenesis abundant (LEA) hydroxyproline-rich glycoprotein family)	191	4459	6.17	20.94
GmLEA2-61	Glyma.11G199600	1	XM_006591204	Chr11:27769806-27772081	AT2G30505.1(Late embryogenesis abundant (LEA) hydroxyproline-rich glycoprotein family)	249	2276	8.88	27.63
GmLEA2-62	Glyma.11G203000	1	XM_003538305	Chr11:28357079-28358465	AT1G65690.1(Late embryogenesis abundant (LEA) hydroxyproline-rich glycoprotein family)	246	1387	10.03	28.18
GmLEA2-63	Glyma.12G023200	1		Chr12:1701710-1704543	AT2G41990.1(molecular_function unknown)	343	2766	9.8	38.70
GmLEA2-64	Glyma.12G093600	1	XM_003539797	Chr12:7654387-7659033	AT3G44220.1(Late embryogenesis abundant (LEA) hydroxyproline-rich glycoprotein family)	213	4647	9.96	23.56
GmLEA2-65	Glyma.12G187600	1	XM_006592709	Chr12:34865817-34869986	AT3G44220.1(Late embryogenesis abundant (LEA) hydroxyproline-rich glycoprotein family)	221	4170	9.34	24.63
GmLEA2-66	Glyma.12G194700	1	XM_003539456	Chr12:35653530-35654090	AT2G46150.1(Late embryogenesis abundant (LEA) hydroxyproline-rich glycoprotein family)	186	561	8.42	20.54
GmLEA2-67	Glyma.12G202900	1		Chr12:36333664-36334359	AT1G65690.1(Late embryogenesis abundant (LEA) hydroxyproline-rich glycoprotein family)	166	696	8.88	18.60
GmLEA2-68	Glyma.13G000500	1		Chr13:154446-155404	AT3G54200.1(Late embryogenesis abundant (LEA) hydroxyproline-rich	207	959	9.23	22.14

					glycoprotein family)				
GmLEA2-69	Glyma.13G248600	1	XM_003541708	Chr13:35666896-35668024	AT5G22870.1(Late embryogenesis abundant (LEA) hydroxyproline-rich glycoprotein family)	203	1129	9.71	23.25
GmLEA2-70	Glyma.13G298800	1	XM_006595387	Chr13:39723376-39724074	AT1G65690.1(Late embryogenesis abundant (LEA) hydroxyproline-rich glycoprotein family)	232	699	7.93	26.32
GmLEA2-71	Glyma.13G307700	1	XM_003541896	Chr13:40333363-40333923	AT2G46150.1(Late embryogenesis abundant (LEA) hydroxyproline-rich glycoprotein family)	186	561	7.93	26.32
GmLEA2-72	Glyma.13G307900	1		Chr13:40339771-40340331	AT2G46150.1(Late embryogenesis abundant (LEA) hydroxyproline-rich glycoprotein family)	186	561	9.03	20.37
GmLEA2-73	Glyma.13G313600	1	XM_003543371	Chr13:40891153-40892294	AT2G27260.1(Late embryogenesis abundant (LEA) hydroxyproline-rich glycoprotein family)	271	1142	10.01	30.00
GmLEA2-74	Glyma.13G313700	1	XM_003543372	Chr13:40902956-40904226	AT3G44220.1(Late embryogenesis abundant (LEA) hydroxyproline-rich glycoprotein family)	218	1271	9.36	24.01
GmLEA2-75	Glyma.13G349900	1	XM_003543546	Chr13:43954196-43955906	AT1G17620.1(Late embryogenesis abundant (LEA) hydroxyproline-rich glycoprotein family)	254	1711	10.24	27.84
GmLEA2-76	Glyma.13G357600	1		Chr13:44542513-44544526	AT1G45688.1(molecular_function unknown)	302	2014	9.62	33.33
GmLEA2-77	Glyma.13G359200	1	XM_006595073	Chr13:44651121-44653243	AT1G52330.1(Late embryogenesis abundant (LEA) hydroxyproline-rich	201	2123	10.06	22.71

					glycoprotein family)				
GmLEA2-78	Glyma.14G010800	1	XM_003545035	Chr14:824392-825474	AT4G26490.1(Late embryogenesis abundant (LEA) hydroxyproline-rich glycoprotein family)	230	1083	9.85	26.19
GmLEA2-79	Glyma.14G037300	2	NM_001254648	Chr14:2808746-2811889	AT2G44060.1(Late embryogenesis abundant protein, group 2)	320	3144	4.8	35.65
GmLEA2-80	Glyma.14G041700	1	XM_003545437	Chr14:3150091-3152649	AT2G27080.1(Late embryogenesis abundant (LEA) hydroxyproline-rich glycoprotein family)	273	2559	9.65	30.38
GmLEA2-81	Glyma.14G160000	1		Chr14:36711633-36712196	AT3G54200.1(Late embryogenesis abundant (LEA) hydroxyproline-rich glycoprotein family)	187	564	9.39	20.81
GmLEA2-82	Glyma.14G208000	1	XM_003544903	Chr14:47379161-47380263	AT1G65690.1(Late embryogenesis abundant (LEA) hydroxyproline-rich glycoprotein family,SYNTAXIN)	260	1103	10.15	29.48
GmLEA2-83	Glyma.15G015800	1	NM_001254412	Chr15:1250483-1253447	AT1G45688.1(molecular_function unknown)	309	2965	9.95	34.24
GmLEA2-84	Glyma.15G024400	1	XM_006597127	Chr15:1962152-1963820	AT1G17620.1(Late embryogenesis abundant (LEA) hydroxyproline-rich glycoprotein family)	254	1669	10.28	27.92
GmLEA2-85	Glyma.15G048800	1	XM_003547015	Chr15:3852038-3855379	AT2G01080.1(Late embryogenesis abundant (LEA) hydroxyproline-rich glycoprotein family)	260	3342	9.66	27.89
GmLEA2-86	Glyma.15G065400	1	XM_003545751	Chr15:4971607-4972756	AT5G22870.1(Late embryogenesis abundant (LEA) hydroxyproline-rich glycoprotein family)	203	1150	9.62	23.24

GmLEA2-87	Glyma.16G031000	1	XM_003548573	Chr16:2934876-2936089	AT2G46150.1(Late embryogenesis abundant (LEA) hydroxyproline-rich glycoprotein family)	219	1214	10.2	24.19
GmLEA2-88	Glyma.16G031300	1	NM_001251006	Chr16:2949165-2950467	AT1G01470.1(Late embryogenesis abundant protein)	152	1303	5.16	16.69
GmLEA2-89	Glyma.16G221900	1	XM_003548290	Chr16:37848866-37850458	AT3G54200.1(Late embryogenesis abundant (LEA) hydroxyproline-rich glycoprotein family)	192	1593	9.3	20.54
GmLEA2-90	Glyma.17G172900	1	XM_003550013	Chr17:17752933-17754945	AT1G45688.1(molecular_function unknown)	323	2013	10.01	35.52
GmLEA2-91	Glyma.18G047800	1	XM_003553105	Chr18:4153739-4154925	AT1G65690.1(Late embryogenesis abundant (LEA) hydroxyproline-rich glycoprotein family)	244	1187	9.97	27.86
GmLEA2-92	Glyma.18G073200	1	XM_003552938	Chr18:6901495-6902707	AT4G26490.1(Late embryogenesis abundant (LEA) hydroxyproline-rich glycoprotein family)	258	1213	10.05	29.48
GmLEA2-93	Glyma.18G218700	1		Chr18:50553919-50555110	AT3G05975.1(Late embryogenesis abundant (LEA) hydroxyproline-rich glycoprotein family)	221	1192	9.83	24.28
GmLEA2-94	Glyma.18G218800	1	XM_003551579	Chr18:50556074-50559120	AT3G54200.1(Late embryogenesis abundant (LEA) hydroxyproline-rich glycoprotein family)	196	3047	8.6	21.38
GmLEA2-95	Glyma.18G238700	1		Chr18:52756503-52759147	AT1G01470.1(Late embryogenesis abundant protein)	176	1617	5.83	18.93
GmLEA2-96	Glyma.19g198600	1		Chr19:45575608-45576562	AT2G35980.1(Late embryogenesis abundant (LEA) hydroxyproline-rich	216	955	9.63	24.48

					glycoprotein family,SYNTAXIN)				
GmLEA2-97	Glyma.19G198700	1	NM_001252717	Chr19:45578902-45579788	AT2G35980.1(Late embryogenesis abundant (LEA) hydroxyproline-rich glycoprotein family,SYNTAXIN)	227	887	9.34	26.41
GmLEA2-98	Glyma.19G198800	1	XM_003554413	Chr19:45582865-45583763	AT2G35980.1（Late embryogenesis abundant (LEA) hydroxyproline-rich glycoprotein family，syntaxin）	227	899	9.34	26.43
GmLEA2-99	Glyma.19G198900	1		Chr19:45588629-45590084	AT3G11660.1（NDR1/HIN1-like 1)	210	1456	9.81	23.91
GmLEA2-100	Glyma.19G260500	1	XM_003554739	Chr19:50373544-50374912	AT4G01410.1（Late embryogenesis abundant (LEA) hydroxyproline-rich glycoprotein family）	197	1369	9.89	21.54
GmLEA2-101	Glyma.20G044800	2	NM_001254150	Chr20:8268501-8275551	AT2G44060.1(Late embryogenesis abundant protein, group 2)	312	7051	4.72	34.36
GmLEA2-102	Glyma.20G140900	1	XM_003555974	Chr20:37967537-37968547	AT4G05220.1(Late embryogenesis abundant (LEA) hydroxyproline-rich glycoprotein family)	227	1011	9.36	26.06
GmLEA2-103	Glyma.20G216300	1	XM_003556371	Chr20:45204176-45205311	AT5G36970.1(NDR1/HIN1-like 25)	250	1136	9.83	27.94
GmLEA2-104	uncharacterized protein LOC100797168	1	XM_006599136	Chr16:18333921-18335433	AT5G45320(Late embryogenesis abundant protein, group 2)	207	1513	9.23	22.77
GmLEA2-105	protein YLS9-like	1	XM_006577031	Chr3:42994014-42994917	AT2G35980(NDR1/HIN1-like 10)	227	904	9.5	26.22
GmLEA2-106	uncharacterized protein LOC100784646	1	XM_003548348	Chr16:1579950-1580875	AT4G01410(late embryogenesis abundant hydroxyproline-rich glycoprotein)	250	926	9.51	27.90
GmLEA2-107	uncharacterized protein	1	XM_006589805	Chr10:9182898-9183497	AT2G27260(late embryogenesis abundant hydroxyproline-rich	199	600	8.84	22.42

	LOC102663298				glycoprotein)				
GmLEA2-108	protein YLS9-like	1	XM_003533271	Chr9:37252919-37253898	AT2G35980(NDR1/HIN1-like 10)	178	537	9.44	21.08
GmLEA2-109	uncharacterized protein LOC100777620	1	XM_003526081	Chr06:43350865-43360367	AT2G01080.1(Late embryogenesis abundant (LEA) hydroxyproline-rich glycoprotein family)	624	1875	8.12	68.95
GmLEA2-110	uncharacterized protein LOC100807939	1	XM_003543336	Chr13:39157431-39157958	AT3G54200(late embryogenesis abundant hydroxyproline-rich glycoprotein)	175	528	8.57	19.28
GmLEA2-111	uncharacterized protein LOC102659599	1	XM_006574253	Chr01:55422524-55423102	AT3G54200(late embryogenesis abundant hydroxyproline-rich glycoprotein)	192	579	8.13	21.16
GmLEA2-112	uncharacterized protein LOC100804569	1	XM_003543939	Chr13:11874210-11876570	AT3G54200(late embryogenesis abundant hydroxyproline-rich glycoprotein)	376	1333	9.57	40.96
GmLEA2-113	uncharacterized protein LOC100800449	1	XM_006593218	Chr12:35682617-35682985	AT3G54200(late embryogenesis abundant hydroxyproline-rich glycoprotein)	122	369	7.75	13.47

Table S2. The coding sequences and protein sequences of LEA2 gene family.
The coding sequences
>GmLEA2-1
ATGCCGCCCCACCGCGATCGCGACCATGACCACGACGACGATCACGTGGCTGAGCACC
ACCACCACCACCACCACAGACCCATCAGAAGAAAATTGGTGAACACTCACCACAGCG
GCAAAACACACCCTCTTATCTGGCTAGCGGCCATTCTCTGCACCATCATAGCCATAGGC
GTTGTAATAGCCGGCATAGTTGTTTTCGTGGGTTACATGGTGATCCACCCTAGAATCCCT
GTGATCAGTATCACCAATGCCCACCTCGATCTTCTCAGCAACGACTACACGGGCCTCCT
CCAGACGCAGCTCACCATCATCGTCGTGGCGCAGAACGGCAACGCCAAGGCCCATGC
AACGTTCTCCGACATAAGGTTCAACCTAAGCTACCAGGGACAAGGAATTGCTGTTATG
CTCGCTCCGCCATTCGATGTGGCCAAGAACAGCTCCAAGCCACTCAACTACGTTGTTC
GGTCTGCTTCCATTCCGTTAACTCCTGAGCAGATGGAAGAAGTGGATGAGTCGTGGAA
GCGTGACGTTATAGGGTTTGACTTGAAGGGAAGCGCGAGGACACGGTGGAGGGTTGG
GCCGTTAGGTTCTGTTAAGTTCTGGTGCAATTTGGAGTGTCAGCTCAGGTTTCGTCCTT
CTAATGGGAGTTACATTCATCACTCACGCTGCACCTCTGAGTCTAAATAA
>GmLEA2-2
ATGGCCAAGTCCATACAGCAAGAGCGAAGAAGCGGCAAATGTTTCGTATATTTGCTGG
CAGCCTTTGTCATCTTATGCGCACTTGTGTTGGTTTTTGCGTCCCTATTGCGCGTCAAGA
ATCCATATCTCAAATTAAGATCAGCTACTTCCAACAAAATCAGTTACAGCACTTCACCTT
CCTTCAACGCCACCCTCATCATCTTTCTCGGCATCAAGAACCCAAATTTCGGCGCCTTC
AGTTACAATAATAACAGGGTGAGTGTGCTCTATGCGGGTGTAAAGATCGCTGATAGGCA
AATCAATGGTGGCAGGGTGCGTTTCAGAGAAACCAAAGAAATTAATGTTACTGTGAAA
TTGATGTCCGCTAAGGCACCCATTAGTGAGAATCTTTCTATTGACATTAGTTCGGGGTC
GTTGAATCTCACGAGTAATGTCAAATTTAGTGGCACAGTTCATATGCTCAAGATTATCA
ACATCAGAAAGACCATAGAAATGGCTTGTGCTATGAAGCTCAACTTGACCTCGCACAC
AATTCAGGGTATTCAATGCCAATAG
>GmLEA2-3
ATGTGCCCGTGGGCGTGGGTTATGTTTCACTTTGCCACATCAATCCATCCATCCTCACAC
TCCAACACCGAGCGAGCCATGGCGAAGCCCGAGAACCGCACTAACTTAGCTTCGTGTT
TCGTGGCCACCGTTTTCTTGATCTTCCTTCTCATTGTGGTCTTCATCGTTTACTTCACTG
TGTTCAAGCCGCAGTACCCCAAAATCGCCGTGAGCGCCATCCAGATCCCTTCCTTCTCC
GCCACTAACGGCACCGTCAACTTCACCTTCTCCCAGTACGCCTCCGTTAGGAACCCTA
ACCGAGGCACCTTCTCCCACTACGACAGCTCGCTCCAGCTTCTCTACTACGGCAGACA
GGTCGGCTTCATGTTCGTTCCGGCCGGAAAGATCGCCGCCGGCAGGACTCAGTACATG
GCCGCCACCTTCACCGTTCAGTCCTTTCCCTTGGGCCTGGGGCCCACCTCCGTGGATG
GGCCTAGTAGTGTGGGGCCCACGATGGAGATGGAGTCGAGGATAGAGATGGCGGGGC
GCGTGAGAGTGTTACACCTTTTCAGTCATCACGTGGAGGCCAAAGCTCAATGCAGGGT
CGCCATCGCCATCAACGATGGATCGGTGTTAGGTTTTCGGTGCTAA
>GmLEA2-4
ATGCCTGCAATCTCTCCCGCGTACTATGCGCCTAACTACAATAACTTGCTGCACCATTCA
CCACTTTATAATGAAATTAATGCGCTCTCTCAAATATCAAACATTTTTGTCAAGAGTCTC
TGCATATGGCTCCTGCAAGTTATAGGCCTCTTAAGCCTTATTGTTCTGTGCCTATGGCTG
GCCTTACGACCCAAGAGCCCCTCCTACTCCATTGTGTTCATCTCCATAGGACAGCCCTC
GAACTCAAATGAAAACAACACCATCTTTTACAACCTTGATATTGAAAACCCAAACAAA

GACTCGAGCATTTACTATGACGACACAATATTAAGTTTCTTATATGGGGAGCAAGAGGA
TGATGTGGGGGAGACAACTATAGGCTCATTCCACCAAGGAACTGGCAACACCCGGGAT
GTATCCGATACAGTTAATGCCAAACCTCGACCATTCAAACCTCTCTTAAATGCCATCTCA
AATGCAACCAGAGTTAAAAGTTGCTTTGATCACAAGATATCAATACAAGACATGGGGA
ATTAA
>GmLEA2-5
ATGTCGGAGTGCGAGCACCACAAGGGGAAGAAGAGGAAGATCTTCCGGCAAGTGTTC
TGGTGCATAGTGGTGTTCCTCTTCCTCGTGCTGCTCACAATTCTTTTGATATGGGCAATC
CTTAGACCCACCAAACCCACCTTCACCCTCCAAGACGTCACGGTCTACGCCTTCAACG
CCACCATACCGAACTTCCTCACTTCGAATTTTCAGGTCACGCTCATCTCGCGCAACCCG
AACGACAACATCGGTGTTTACTACGACCGCCTCGAAATCTACGTAATCTACCGGAGCCA
GCAGATCACGTACCGAACCGCCATCCCTCCGACCTACCAGGGCCACAATGAGATCAAT
GTTTGGTCTCCGTTTGTTTATGGTACCAACATCCCCGTCGCGCCGTTCAATTTCCTCCGC
CTCAGCCAGGACCAGAGTGACGGTAATGTCCTCGTCACCATCCGAGCCGACGGAAGG
GTTCGTTGGAAGGTCGGCGCCTTCATCTCCGGTCGCTACCACTTCTACGTCCGCTGCCC
TGCCTTCATCTCCTTTGGCCCCCGCAGCAATGGAATCGTCGTCGGAGAAAACGCCATC
AAGTTCCAGATAATCCAGCGGTGCTCCGTTAGTGTCTAA
>GmLEA2-6
ATGTCCCATCTAAACGGCGCGTACTACGGCCCCTCCGTTCCGCCGCCGAAATCCTACCA
CCGCCCCTCCCGCGGCGGCCGCGACGGCTGCTGCTGCGGCTGCCTGAGCTGCTTCTGC
GGCTGCATCTTCGACTGCATCCTGGGCCTCATCTGCAAGATCCTCACCACAATCCTCAT
CATCGTAGCAATACTCGGCTTTCTCTTCTGGTTCATCGTGCGCCCCAACGTGCTCAAAT
TCCACGTTACCGACGCGTCCCTCACGCGCTTCGACTACACCACCAACAACACTCTCCA
CTACGACCTCGCCCTCAACGTCTCGATCCGCAACCCGAACCGCAGGGTCGGCGTCTAC
TACGACCACATCGAAGCGCACGCGCTGTACCAGGATGTTTTGTTCGGGAACCAGACAT
TAGGGCCGTTCTTCCAGCACCACAAGAACACCACCTTCGTGAATCCCCTTTTCAAGGG
CCAGCGCGTGACGCCACTCGCCGGGAACCAGGTCGAGGTGTTTGACAAGGAGAAGG
GTTCGGGTGTTTACACCATCGATTTGAAGCTCTTTATGGTGGTTCGGTTCAAGTTCTTG
CTGTTCAAGAGTGCGAGCGTGAAGCCCAAGATTCGGTGTGCGTTGCACGTGCCGCTG
AAATCGCGTAACGCAACAACAACTATCTCCCCCGATGCTGCGTTTCAACCCACAGAGT
GCGGTTGGGATTACGGGAAAAAATGGTGGATCCATTAG
>GmLEA2-7
ATGGCAGACCCTCAGAAAATTCACCCTGTGCACCATGATGTTGAGGCACAAAACCACC
CTTCAGCACCATTGGTGCCAAGGAGCATGTCCAAATCTGATGCAGGTGATCCACAACG
AGTAGTAGTACAACAACAACAACAAAACATTCCTCTGAAGCAAACCAAGCCACCACC
GAAGAAAAGAAGAAGCTGCTGCTGCAGGTTCTTCTGTTGGCTAATAAGCATATTGCTG
ATTCTGATAGTGGCAATTGGCATCACTGTTGGAATACTCTACCTTGTCTTCAGGCCAAA
GCTTCCAAAGTACTCGGTGGACGAACTTAGAGTCACACACTTTGATCTTGCAGACAAC
AACAGCTTATCAGTGACATTCAACTTGACAATAACCGCCAGAAACCCCAACAAAAAAA
TTGGAATAGACTATAGGGGTGGCAGCCACATAAGTGCTTGGTACATGGACACAAAATT
GTGTGAAGGGTCTTTGCCAAAATTCTACCAAGGTCACCGCAACACCACGGTGCTTAGC
ATCCCTCTTACTGGGAAAACGCAGGATGCTACCGGCTTGCAAAGTACCCTTCAGAATC
AGCTGCAAGAAAGTGGGAATGTGCCTCTCAATTTGAAGGTGAAGCAGCCTGTGAGGA
TCAAGCTTGGGAAGTTGAAGCTGTTCAAAATCAAGTTCAGGGTTAGGTGCAAGATTGT

GGTGGATAGCCTTAGTGCTAATAGTTCTATCAGAATTCAGAGTAGTAGTTGTAAATTCAG
GTTTAGGCTGTGA
>GmLEA2-8
ATGGCCGACCGAGTTCACCCCAGCCACTCGCCTTCCGTTTCCGCCGACTCACAACCGG
CGTCGCCTCAAGACTCCTCCGTCGTTCCAAAACCGCCGTTGCCACCGTCGTCGGAGAA
GCCAGTGCCTCCGCCTGGAACCTACGTCATCAAGATCCCTAAGGACCAAGTCTATCGC
GTCCCTCCGCCGGAGAACGCTCGCCGCTACGACCAATACACTCGCCGTAAACACCGCC
GGAGCCGATGCTGCTGCTGCTTCTGCTGGCTCATCGGAATCCTCTTCATTCTCGTCGTT
TTCCTCGCCATCGCCGCCGGCGTCCTCTACCTCGTTTTCCGTCCCGAGGAGCCGAAATA
CTCCATCGAAAACATCGCCGTCAGAGGAATTAACCTCACCTCGCCGTCATCCACGGCG
GCGATGTCGCCGGTGTTCAACGTCACGGTCAAGGCCGATAACCCTAACGATAAGATCG
GAATCCGTTACTTGAAGGATAGCTCCGCCGAAGTGTTTTATAAGGACGCGAGGCTGTGT
AACGGCGCTCTGCCGGCGTTTTACCAGCCGTCAAACAACGTGACGGTGTTTGGGACG
GCGTTAAGGGGTGACGGAATCGAACTGAGGAGCGAGGTCCGAAGAGCGTTGCTGGAG
GCGCAGACCAAACGGAGGGTGCCGTTGACCGTTAGGATTAGGGCGCCGGTGAAAATA
AAAGTGGGGTCCGTCAAGACGTGGAAGATAACCGTTAAGGTGAACTGTCATATGACGG
TGAATGAGTTAACGGCGCGGGCCAAGATTGTTTCTAAACGTTGTAATTATGACGTGGAT
CTTTGGTGA
>GmLEA2-9
ATGTCGACATCTGATAAGCCAGAAGAGGTGGAAAGGGGTAGCAAGGATGAAAAGCAC
AAGGAAGATGATCAAGAGGAGGGGAAGGGTGGATTCATTGAGAAGGTGAAGGATTTC
ATTCATGACATTGGTGAGAAGATTGAGGAAGCTATTGGGTTTGGGAAGCCGAGTGCCG
ATGTTACTGCGATACACATTCCATCAATTAATCTTCACAAGGCAGATCTTGTTGTTGATG
TGCTCATCAAGAACCCGAATCCGGTGCCGATCCCTCTGATTGACATAGATTACTTGGTT
GACAGTGATGGAAGGAAGCTAGTTTCCGGATTGATACCTGATGCGGGTACCATCCATGC
ACATGGAGAGCAAACTGTCAAAATTCCTGTCACTTTGATTTATGATGACATCAAGCAAA
CATATGCTGATATTAAACCTGGAAGCATCATTCCTTATAGGGTGAAGGTTAGTCTCATTT
TTGATGTTCCCATCTTGGGAAGGCTAACTCTACCTTTGGAGAAAACTGGAGAAATTCCC
ATACCATACAAGCCTGATATTGATCTTGAGAAGATTCATTTTGAAAGGTTCTCTTTTGAA
GAGACAATTGCAACTCTTCACTTGAAGTTGGAAAACAAGAATGATTTTGACCTCGGCC
TCAACGCGCTCGATTATGAGGTCTGGCTTGGTGATGTTAGCATTGGTGGTGCAGAACTC
ACCAAGTCTGCTAAAATTGAAAAAAGTGGAATTAGTTATATTGATATTCCAATTACCTTC
AGGCCTAAGGATTTTGGCTCTGCACTATGGGACATGATTAGAGGAAGAGGAACTGCTT
ACACCATAAAAGGACATATTGATGTTGACACTCCCTTTGGAGCAATGAAGTTGCCCATC
AGCAAAGAAGGTGGTACTACCCGTCTTAAGAAAAAGAAGGAAGATCGTGATTATGATG
ATGATGACGATGATGAGCAGGAATGA
>GmLEA2-10
ATGCCAACCGAATCTGGTCCAAATGTGGTGGCTAATGAAGAACTTGGCACACCAAAAC
AACCAAGGAATCAACATTCTCAGCAATCAGGAGCACCAAATTCCCTTGCCAACACCAG
AGCCATTTTCGGGCAACCTCGACTTCAAAGGACAAAACCAATTATATGGTGTGCTGCA
ATAATGTGCTTCATATTCAGCCTAGTGCTTATCTTCTTTGGAATTGCAACACTGATTCTCT
ACCTTTCCATGAAGCCGCGAAACCCCACGTTTGACATTCCCAATGCAAGCCTCAATGT
GGTGTACTTTGACTCACCACAGTACCTCAATGGTGAATTCACTCTCCTTGCAAATTTTT
CCAATCCTAACAGGAGAATTGGGGTGAGGTTTGAGTCCTTGAACATTGAGCTTTTCTAC

TCAGACAGGCTCGTTTCTTCACAAACAATAAAGCCTTTTACTCAAAGGCCAAGGGAAA
ACAGGTTGCAATCAGTGAACTTGATATCAAGCTTGGTGTTTTTGCCTCAAGATGTTGGT
GTAAAACTACAAAGGCAAGTGGAGAATAACAGGGTCAATTACAATGCAAGGGGAACA
TTTAAGGTGAGGTTCAATGTTGGCCTTATCCATTTATCTTACTCTCTGTATAGCACATGCC
AGATAGAGATGACTAGTCCTCCAGCGGGTATTCTAGTAGCCAGACAATGCATAACAAAC
CGATGA
>GmLEA2-11
ATGAAGGTAGGATCTGGTAAAGGGAGAAAAGTGTGCCTGACGGTGACAGGTGTTGTG
ATTGCAATTGTATTGCTAATTGTGATACTAGCGTTGACAGTGTTCAAAGCCAAGCATCCT
GTTACCACAGTGGACTCAACGAAGCTAGAGGACTTTCACGTGAGCTTGGATCCAGTAA
AACTAAGGGTAGATTTGAATGTGACCCTGGGAGTGGATGTCTCAGTGAAGAACCCGAA
CAAGGTGGGATTCCAGTATTCAGACAGCACTGCCCACCTCAATTACAGAGGGCAGCTG
ATAGGTGAAGTCCCGATCTCTGCCGGAGAGATTTCATCCGGTGAGACCAAAGGATTCA
ATCTCACCCTCACCATTATGGCCGACCGTTTGCTCTCCAATTCTCAGCTTTTATCTGATG
TCACATCTGGTACATTGCCCCTAAACACTTTCGTGAGGATGTCTGGGAAAGTCAGCATC
TTAGGCTTTATCAAAGTCCATGTGGTTTCCTCCACTTCTTGTGATGTTGCAATTAATCTT
TCTAATGGAACTGTTGGGAACCAAGAGTGCCAGTACAAGACAAAACTTTGA
>GmLEA2-12
ATGTCCCAGTTGAACGGAGCCTACTACGGCCCCTCCATCCCGCCGCCGAAAACTTCCT
ACCACCGTCCTGGGCGCGGCGGAGGCCTCGGCTGCTGCTGCGGGTGCCTCTTCAGCCT
CATCTTCAAGCTCATCCTAACCGTGATCATCATCATTGGCATCGCCGTGTTCCTTTTCTG
GCTCATAGTCCGTCCCAACGTGGTGAAATTCCACGTCACCGAGGCCACCCTGACGCAG
TTCAACTACACCCCCAACAACACGCTCCACTACGACCTCGCCCTCAACATCACGGTCC
GAAACCCCAACAAGAGGCTCGGAATCTACTACGACCGCATCGAGGCGCGTGCAATGTT
CCACGACGCGAGGTTTGATTCCCAGTTCCCGGAACCATTCTACCAGGGCCACAAGAGC
ACCAACGTGCTGAACCCGGTGTTTAAGGGTCAGCAATTGGTGCCACTCAACGCTGACC
AATCCGCGGAACTGAAGAAGGAGAACGCCACTGGGGTGTACGAGATCGATGTGAAGA
TGTACCTTAGGGTCAGGTTCAAGTTGGGTGTCTTCAAGACCAAGACGCTTAAGCCCAA
AGTATCATGCGACTTACGTGTTCCTTTGAAAGGAAGCGCCGGTGCTGGTGTCTTTCAG
ACCACCAAGTGCGACTGGGATCGCTGA
>GmLEA2-13
ATGGCCGATAAGCAACCCCACTTGAATGGTGCTTATTACGGTCCCGCCATTCCCCCGGC
GGAGCAACCACACTACCGCCCTAGCCGCGAAAGAAGCTGCTGTTGCTGCCTCTTCGGA
ATCTTGTGGAAGATTCTGGTTGCACTCATTGTCCTCGTTGGCCTCGCGGTCCTCATCTTC
TGGCTGGTGGTTCAACCCCGTTCCTTCAAGTTCCACGTCACGAAAGCCAACCTAACAC
AATTTGATTACTATACCAACAACAACACCCTTCACTACAACATGGTCCTCAACTTCACT
GCACGCAACCCCAACAAAAAACTCAGCATATACTACGACAAAGTAGAGGCATTAGCAT
TCTACGAGGATGTCAGGTTCGCCAATTACAGTGTGATAACGCACATGAACTCCTTCCGC
CAGTACAAGAAGACCACCAGCCACATGAGCGCCGTTTTCTCGGGGCAGCAAGTGTTG
CCGCTCGACAACGACCTAGTCTCAGAGTTGAACCAAGACAAGAGTGGTGGGGTGTAC
GAGATCGATGTGAAGCTCTACTTCAGGATTAGGTTCAGGCTCGGGGATGTCAAAACCC
GTCGCTTCAAGCCCGAGGTCAAATGTGATATCAGGGTTCCCTTGAGGACCAATGGCAG
CGTAACTTTGTTTCAGACCACCAAGTGTGATGTCGATTACTAG
>GmLEA2-14

ATGGCAGATTATAAGCAACACCACGGAAGAAGCTGCGGCTGCTGCTGCCTCTTTGGAA
TCTTGTGGAAGATTGTGGTTGCACTCATTGTCCTCGTTGGTCTTGTATTCCTCATATTTT
GGCTGGTTGTTCAACCCCGCTACTTCAAGTTCCACGTCACGGAAGCGGACCTAACTCA
ATTTGAGTACTATCCAAACAACAACACCCTTCACTACAACATGGTCCTCAATTTCACTG
CACGCAACCCCAACAAGAAGCTCAGCATATACTACGACAAAGTTGAGGCATTAGCATT
CTACGAGGATGCCAGGTTCGCCAATTACGATGTCATAACTCACATGAACTCCTTCCGCC
AGTACAAGAAGAGCACCAGTCCCATGAGCGCCGTTTTCTCGGGGCAGAAGGTGTTGAT
GCTCAACAACGAACAAGTCTCTCAGTTCAACCAAGACAAGAGTGTTGGAGCTTATGAT
ATCTATGTGAAGCTCAACTTCAGGATTCGGTTCAGGCTCGGGGACTCGATTTCCCGTCA
CTTGAAGCCCAAGGTCAAATGTGATCTCAAGGTTCCCTTGAACAAAAGTGGCACCTTT
ACTCTGTTTCAGACCACCAAGTGCCATGTCAATGTCTAG
>GmLEA2-15
ATGGCCGATAAGCACGGAAGAAGCTGCTGTTGCTGCCTCTTTGGAATCTTGTGGAAGA
TTCTGGTTGCACTCATCGTCCTCGTTGGCCTCGTATTCCTCATATTCTGGCTGGTGGTTC
AACCCCGCTCCTTCAAGTTCCAGGTCACGGAAGCGGACCTAACCCAATTTGATTACTA
CACCAACAATCACACTCTCCACTACAACATGGTGCTCAACTTCACTGCACGCAACCCC
AACAAGAAGCTCAGCATATACTACGACAAAGTTGAGGCATTAGCATTCTACGAGGATG
CCAGGTTCGCCAATTACGATGTCATAACTCACATGAACTCCTTCCGCCAGTACAAGAAG
AGCACCAGCCCCATGAGCGCCGTTTTCTCGGGGAAGAAGGTGTTGATGCTCAACAGC
GAACAAGTCTCTAAGTTGAACCAAGACAAGAGTGATGGGGTTTATGACATCTACGTGA
AGCTCAACTTCAGGATTCGGTTCAGGCTCGGGGACTCCATATCCGGTAACTTGAAGCC
CAAGGTCAAATGTCACCTCAAGGTTCCCTTCAGTAAAAGTGGCACCTTTACTCTCTTTG
AGACCACCAAATCTGCTGTTGCTGCCTCTTTGGAATCTTGTGGAAGATTCTGGTTGCAC
TCATCGTCCTCGTTGGCCTCGTATTCCTCATATTCTGGCTGGTGGTTCAACCCCGCTCCT
TCAAGTTCCAGGTCACGGAAGCGGACCTAA
>GmLEA2-16
ATGGCCGATAAGCAACCCCACTTGAACGGCGCTTATTACGGTCCCGCCATTCCTCCGGC
GGAGCAACCACGCTACCGCCCTCACAGTCACCGCGGAAGAAGCTGCTGTTGCTGCCT
CTTCGGAATCTTGTGGAAGATTCTGGTTGCACTCATTGTCCTCGTTGGCCTCGCGGTCC
TCATCTTCTGGCTGGTGGTTCAACCCCGTTCCTTCAAGTTCCACGTCACGGAAGCCGA
CCTAACACAATTTGATTACTATACCAACAACAACACCCTTCACTACAACATGGTCCTCA
ACTTCACTGCACGCAACCCCAACAAGAAGCTCAACATATACTACGACAAAGTTGAGGC
ATTAGCATTCTACGAGGATGTCAGGTTCGCAAGTTACGATGTCATAACACACATGAACT
CCTTCCGCCAGTACAAGAAGAGCTCCAGCCCCATGAGCGCCGTTTTCACAGGGCAACA
ATTGCTGATGCTGAACAATGATCAAGTCTCAGAGTTCAACCAAGACAGGAATACTGGT
GTTTATGACATCTATGTGAAGCTCTACTTCAGGATGAGGTTCAGGCTCGGGGACTTTATA
TCCAATGACTACAAGCCCAAGGTCAAATGTCACCTCAAGGTTCCTTTCAGTAAAAATG
GCACCTTTACTCTGTTTCAGACCACCAAATGTGATGTCGATTTCTAG
>GmLEA2-17
ATGATCTTACCATATCATTTCAAGACCTTAAAGTACCAAACTTCCAACTCTTCTCTCTTC
TCTCTTCACTCTTCACTCTTGTCGTCATTTCCTCACAAAACCAGTGTCCGTAACATCTCC
ACCTTAACGATGTCGGTAAAGGAGTGTCACCACCACAAGGGAAAGAAGCACAAACTC
TGGAGACGCATCTTCTGGGGCATAGTGATCTTCGCCTTCATCGTGTTGCTAACAGTTCTT
ATAATCTGGGCAATCCTAAAGCCTTCAAAGCCAACCTTCATCCTCCAAGACGTGACCGT

CTACGGCTTCAACGCCACCATCCCCAACTTCCTCACCTCAAGCTTTCAGGTCACGCTTT
CCTCGCGCAACCCCAACGACAAAATCGGAGTCTACTACGACCGTCTCGACACCTACGT
CACTTACCGGAACCAGCAGGTCACGTACCGAACCTCAATCCCACCCTCATACCAAGGT
CACAAGGAAGAGGATGTGTGGTCCCCATTCGTCTTCGGCACTAACGTCCCCGTCGCAC
CCTTCAACTTCGTTGGTCTCAGTCAAGACCAGACCAACGGCAACGTCCTCGTCCTCGT
TAAGATTGACGGTAAGGTACGATGGAAAGTTGGTACCTTCGTCTCTGGTCACTACAACC
TCTATGTAAGGTGCCCTGCTTTCATAACTTTTGGCCCCCAAAGCACCGGGATTGCCCTT
GGCAAGAACGCCGTTAAGTATCAATTGGTTCAACGCTGCACCGTCGGGGTTTGA
>GmLEA2-18
ATGTCAAAGGACAAAGTTTCCGGTGATCCAAGACGCGCAGTGTGCACAGGCATCACCA
TCTTCCTCCTCTTAGCCGGCGTCACTCTCCTAGTCCTCTGGCTGGTCTACCGTCCCCAC
AAGCCGCGCTTCACAGTAATCGGCGCCGCCGTCTACGACCTAAACACCACCACCCCAC
CGCTGATGTCGACCACCGTGCAGTTCTCCGTCCTCATAAAGAACCCGAACAGGCGTGT
CTCCATTTACTACGACAGGTTCTCCGCCTTTGTCTCGTACAGGAACCAGGCCATAACGC
CGCAGGTTCTGCTGCCGCCTCTGCACCAGGAGAAGCGAAGCTCGGTGTCGGTGTCGC
CGGTGATGGGAGGCACGGCGCTTCCGGTGTCGGTGGAGGTGTCTGACGGGCTGGCGG
TGGACGAGGCTTATGGGTTGGTGGGTCTGAGGCTGATATTTGAGGGCAGAGTGAGGTG
GAAGGCCGGGGCCATCAAAACTGCGCACTATGGACTGTACGTTAAGTGCGATGTTTTG
ATGGGTTTGAAGAAAGGGTTGGTGGGTCAAGTTCCTCTCCTGGGAGTTACACCCTGCC
ATGTCCATCTATGA
>GmLEA2-19
ATGGATGTTTCCACACAGAATCCCGTGTTTTCTTCCAAGAAATGCTGTTTCTTCCTTTTG
ATCATATGCATGGTGGTTGGATTAGCCACTTTTTTTATGATCCTCATCATTAGACCCCACA
AGCCTGTTTTCTCTGTTCGGGAGGTTAAAATAAACTTTTACAAGATTGATAATGATAGTT
CTAATCTAACTCTGCTGGTTTCATCTGTAATCTACTTAACCCTCAATGCAGAAAATCATA
ATAAGTTTGGCATAGGTTTTAGTTCATCAAGGTTTCTTGTTTATCATGAAGGTTTGCATAT
AGGAACTATTAGAATTCCTTGGTTTTTTCAACCTCCTCACAGTGAAAACGTGAGTGTAC
CATCTCGTGTCTTATTGCATAGTGCCAATCTCACCAAAATCATCGCCAACACATCGTTAC
AAGAAATTTCAAAGCAAAACATGGCACAGATGAGGATCATAGGGGATTCTAGAGCCCA
CGTGTGGGTACTTCATATAAAACTTTTTGAAATTATGATTTTTGCCTTTTCATGTGACTGT
GAGTCACATTTAAGTTGA
>GmLEA2-20
ATGTCCGACCCAGCTTCCAAACCCAACGGAAACGCCGCGGCCGCCACTAACGGCAAT
CCCGGACCCGTTAAGTCTCAACTCTACAACCCCAACCGTCAAGTTTACCGGCCGCAAT
CGCACTACCACCGTCGCGGCCAACGCTCCCACCGCAACTTCTGCTGCTGCTGCTGCTT
CTGGACCATCCTCACCCTCCTCGCGGTGGCTCTCCTCGCCGCCATTGTCGGCGCCGCAC
TTTACGTACTCTACCGCCCTCACCGTCCCGAATTCTCCGTCACAAACCTCCGCATTGCG
AAGATGAACCTCACCACTTCCGCCGACTCCCCTTCCCACCTCACCACGCTCTTCAACC
TCACTCTCATCGCGAAGAACCCTAACAACCACCTCGTCTTCTTCTACGACCCGTTCTCC
ATGACGGTGTTCTCGAACTCCGTCCCCGTCGGGAACGGTTCCGTTACGGCGTTTACTTC
GGACAAGAACAACCAGACGAGCCTCCGCGCGGTGCTGTCGGGGTCGCAAGATCTGGA
CACGGACTCGTTGACTAGCCTGAGATCGGGGCTGAAGATGAAGAGAGGTTTCCCCGTG
GAGATTCAGATGGACACCAAGGTGAAGATGAAGATGGATTGGCTCAAGAGCAAGAAG
GTCGGAATTCGAGTCACCTGCGACGGAATCAGAGGAACCGTTCCCTCCGGCAAGTCTC

CGGCGGTGGCCTCCGTCGTCGACTCCGAGTGTAAGGTGGATCTTCGAATCAAGATCTG
GAAGTTCTCCTTTTAA
>GmLEA2-21
ATGGAGGAGCGGGTGTTGTTGCCATCACCGCCACCGCCACCGCCACCTCTCGAAAAG
AAACATAGTACCAACAAACTCGAGTTACCAGACTTCAACCCTGGCACCTACGTTGTCC
AGGTCCCCAAGGACCAAGTCTACCGTGTTCCGCCGCCGGAAAACGCCCGAATCGCCG
AGAGCCATAAGAAAGCCCCTCCCAAGGCGGCGAAAACGTCACGCTGCTGCTTGTTTTG
CGTATTGTTCTTCATTATATTCTTCGTCCTTCTCATTCTTCTAGGAGCCGTTCTAGGTGGT
CTTTTTTCCATGTTACTCACACCAAAGGACCCTCAGTTTTCCATCACACGCTTTAAGGT
GGTGGAGACCAAACCCCACCCCAAATACGACGTCACTTTGGAAGTTCACAATCTAAAT
TCCGACGTGGGAGTTTCGTATAAAAACAAAGGCCATGTCTCCCTCTCTCTTAGGCGAC
AAGAGGTTGCTTCAGGAGCGTACCCTAGTTTCAATCAAGACGCTCACGATAGGACAAC
GTTTGGCGTAACTTTAACATCCTCAAAAGTCGGTCTCCCAAAGGAGGTGGAGGAAAGC
GTCACGAACGATAAGAAGAAAGTGAATGTGACATTCTCTTTGGCCATTCACGCCCTAG
CACGCATGAAAATGGGCTTGTTGCGTAGTGGGACAATGAAATTCGATGTTACGTGCAAT
GTGAAGTTGGATACATTGGCCAAAACCACTCAAGTACTCTCTCAACAATGCGAAACTA
AACGACACTAG
>GmLEA2-22
ATGTCTAAAATCACAATAACCTCTCCAAAACACTGTGCTGGCAAAGAAGGACTTAGAA
TCAAGAAGAACTACAAGAAGATCTACTTCACTTTCTCAGCATTTTTCACCACAATCCTA
CTACTAATACTTCTTATATGGCTCATCCTCCACCCTGCAAAGCCTCAATTCTCCCTCAAA
GAAGTTGACATCTACCAACTAAACCTCTCAGGCCCAAACCTCAACTCCTCCATCCAAC
TCACCCTCCTCTCCAAAAACCCAAACCAGAAAGTTGGCATTTACTACGACGAGATTCA
ATTCTATGCAACCTACAAGGGCCAACAAATTACCGGTGATACCCCTGTGCCACCCTTCT
ATCAAGGCCAAGAAGAGAGTAATCTCATAACAGCATCTTTGGTCGGAAATGCTTTACCC
GTGGCTCCATCTCTTGGTTATGAACTTGGTCGTGATCAAATTGTTGGAAGACTAGTTTT
GAATCTCAAAGCCAATGGAAAGCTTCGTTGGAAAGTGGGAACGTGGGTCTCTGGACG
TTACAGATTCAATGTTAATTGTGTTGCCATCAATGCCTTTGGACCCTCTATTCCTGCAGG
TCCTCTCACTTCAAAGCAAGGGGCTCAGTGTTCTACCACACTTTAA
>GmLEA2-23
ATGAAGCCCAACGACAACATGACAATGCTGGCCAAAACCGACTCCGAGGTTAGCAGC
CTCACGCAGTCTTCGCCGGCGCGGTCCCCGCGACGGGACGTGTACTACGTGCAAAGTC
CGTCGCGGGACTCGTCCCACGACGGCGAGAAGACTACGAACTCGTTCCACTCGAGTC
CGCTGCAGAGCCCCTTAGGCTCCCCGCCCCACTCCCACTCCAACTCCTCCCTGGGCCG
CCACTCCCGCGAGTCCGCCTCCACGCGGTTCTCGGGCTCACGCAAGAGCAGCTCCTCC
GGCAACAACCGCAAGGGTCCGTGGAGGCCCTGGAAGGACCAGTTCCACGCCATCGAG
GAAGAGGGCCTCCTCGACCCCAACGACAATGCCCACCATGGCTTCCCTCGTCGCTGCT
ATTTCCCCGCGTTTGTCGTCGGCTTCGTCGTCCTCTTCTCCGCGTTCTCCCTCATTCTCT
GGGGTGCTAGTCGCCCTCAAAAGCCCGCCATTTCTCTCAAGAGTATAACGTTTGACCAA
TTCGTGATACAAGCGGGAGCGGATATGTCAGGAGTTGCCACAAGTTTGGTGTCCATGA
ATTCATCTGTGAAAATGACGTTTCGTAACACCGCTACATTTTTCGGGGTCCATGTGACC
TCAACTCCCGTGGATCTCAATTATTATCAACTTACTGTAGCCACTGGAACTATGCCAAA
GTTTTATCAATCAAGGAAGAGCCAAAGATCTGTTCGGGTAATGGTAATTGGGAGTCATA
TTCCACTGTACGGAGGTGGAGCCAACCTGAACAGTGTGAACGGTAAACCGGTTGAAC

CGGTGCCGTTGACGTTGAGCGTGATGGTGCGGTCGAGGGCTTATGTTTTAGGCAAATT
GGTGAAGCCAAAGTTCTACAAGAAAATAGAGTGTTCCATCGTTATGGATCCGAAGAAG
ATGGGCAAGGCAATTTCACTCGTCAAGAAATGCACTTACCAGTAA
>GmLEA2-24
ATGCCAATGGAGGGTCACGCCGACGTCTCCGCCACATTTCCGCCGCCTCCTGGCCACC
ACGGCGCCGACACGTATATCGTGCAGCTCCCGAAGGACCAAGTGTACCGCGTGCCGCC
ACGCGAGAACGCGTTGATCGTGGAACAGTACCGCAACCCAGCCACCGCGAAAAAAAG
ACGCGGCGGTTGTTGTTGCTGCTGCAGCCGTCGTGTCTTCCTAACCGTCGCTCTTATCG
TTGTGGCAATCGTTGCCGTCGTTGGCATCACGCTGGCCACGTTGTACTTTATTTTCAGC
CCCTCGGGTCCCAAGTTTACTGTCTCCCATGTGGCCGTTAATAGGAACAAGAGCTCGC
GGGCGGCGCCGCAGTACGAGGTTTCGTTGAGGGCGAGGAATCCGAACGAGAAGTTGG
CGATTGAGTACCAGGATGGGGACGTGTCGCTGTTGTTCGATGACACTAAGGTTGCCGA
GGGGAAGTTTCCGACGTTGGAGCAAGGGCGAGGTGAGGCGAGTGAGGTTACGGTTGA
GCTGACCGGATCCAGTGGAGCGTTGCCCAGGCGAATGAATGGTGGGGACGCAGCCGT
GGATTTGAAATTGGAGATGAAACTTGCGATAAGGATAAGAACCGCGGGACTCGAAACG
TGGGCCATGAGGTCCGATGTGGCGTGTGAGTTTAAGGTCAGCGCTCTCGGGAACGACA
CGCGAGTCTTGTCACAGCAATGCGATACCAAGTTCAAGCAATATTAA
>GmLEA2-25
ATGGCTCAGAGCCCTGATCAGCAGATGAAGCCCTTAGCTCCATTCATATCATCAACCCA
GTTCAGCAGACAAGAAGATCAGTTTCAAGAGAGAACATCCGAACAGAAAATCATTCG
CATAAGAAAGTTCGTGCTGTGCTGCGGTTGTTTCACTGCCCTTGTTGTGATACTCGTGG
TCATACTGATAGTCTTGAGCTTCACCGTTTACAATGTCAAAGAACCCGAGGTGAGGATG
AATTCGGTTACCCTTCTCAGTGGAACTTTTGCAAACGGTGGTGCCACTAACAACGTTAC
ACTTGTTGCTGATATCTTTGTCAAGAACACAAACGCTTTTACCCTGAGGTTTGGAAGCA
CCAGCACCATTGTTTACTATGATGGTGTGAGAATAGGTGAGGGTACTTCTCCACCGGGT
AAGGCCAAGGCAAGAAGGACCATAAGGGTAAACTCGACCTTGGAGATTATGTCAAAG
AAGCTTTTGGAAATCCCAACTTTGAATATTGACCTCAGGGATCAGTTTTTGAATATCAG
CAGCTATACAAGGATAGATGGTAAGGTGAAAATACTCAACATATTTCCGAGGAAGGTTG
TGGTTGAGATGAATTGTACCATTGGATACAACATCACCACTGGGTCGGTCACAAATGGT
GATAATTGTCTTGGAGCCGTTGATATTTAG
>GmLEA2-26
ATGGCGGCCGGAGTCTCTGCTCCGCCCCCCGCCCCGCAGGCGCCGAGGCCAAAGCGG
CCTCGGCCTCCGTCGGGGCGCACCAACCTAGCCTCCTGCGTGGTGGCCACCATCTTCC
TAATCTTCATCATCATCGTTATCCTCATTGTATACTACACCGTCTTCAAACCCCAGGACC
CCAAAATCGCCGTCAACGCCGTCCAGCTCCCCTCCTTCTCCGTCGCTAACGGCACCGT
CAACTTCACTTTCTCCCAGTACGCCTCCGTTAGAAACCCTAACCGTGCCGCGTTCACCC
ACTACGACAGCTCCCTCCAGCTCATCTACTCCGGCAGCCAGGTCGGCTTCATGTTCATC
CCCGCCGGCGAGATCGACGCTGGCAGGACGCAGTACATGGCAGCCACCTTCTCCGTCC
AGTCCTTCCCCCTCTCGGTGCCGCCCAGGATGGGCCCCACATTGGCCAACGGCGACGG
AGTGGGCTTCAACTATGGGCTTAGGGTTGAGCCCACTATGGAAATCGAGTCCAAGTTG
GAGATGGCGGGGCGTGTCAAAGTGTTGCACTTCTTCACCCATCACGTTTATGCCAAAG
CCGGTTGCAGGGTTGCCATTGCCGTAACTGATGGATCTGTCTTAGGTTTTCACTGCTAA
>GmLEA2-27
ATGCATGCCAAGACAGACTCGGAGGTAACAAGCCTGGATGCGTCGTCCAGCACAAGG

TCTCCTCGGCGAGCAGTGTACTACGTTCAGAGCCCTTCCCACGATGGGGAGAAAACGA
CGACGTCGTTGCACTCCACCCCTGTTCTCAGCCCCATGGGTTCCCCTCCTCACTCTCAC
TCCTCCTCCAGCCGCTTCTCCGCTTCACGCCACCGCAATAACCATAATAATAAATCTTGG
AAGGGCATCGACGTCATCGAAGAAGAGGGTCTTCTCCAATCCGAATTAGATCGCCAAC
ATTCTCTCTCTCGTCGATACTATTTCCTCGCCTTCCTCCTCGGCTTCTTCCTTCTCTTCTC
TCTCTTCTCTCTCATCCTCTGGTGTGCCAGCCGACCCATGAAGCCCAAAATCCTCATCA
AGAGTATAAAATTTGACCATCTCAGAGTTCAAGCTGGTTCCGATTCCAGCGGAGTAGCC
ACCGACATGATCACCATGAATTCCACCGTCAAATTCACTTATCGCAACACTGGAACATT
CTTTGGGGTCCATGTCACATCCACACCTTTCGATCTCTCCTATTCAGATATTGTAATTGCT
ACGGGAAATTTGAAGAAGTTCTATCAGTCAAGGAAGAGTCAGAGATTAGTGAGTGTG
GCGGTGATGGGGAACAAGATCCCGTTGTATGGAGGAGGTGCTAGCTTGAGTAGCTCAA
CCGGTGTGCCCACGCTGCCTGTTCCTTTGAATTTGACCTTTGTCATTCGATCCAGAGCT
TACGTGCTTGGGAGATTGGTGAAGCCCAAGTACTACAAAAGGGTTCAATGTTCCATCA
ATTTGGATCCCAAGAAGATTAATGTTCCAATTTCCCTCAAGCATTCTTGCACCTATGATT
GA
>GmLEA2-28
ATGTCTGACCCAGCCTCCAAACCCAGCGGAAACGGCGCGGCCGCCACTAACGGCAAT
CCCGGACCCGTCAAGTCTCAACTCTACAACCCCAACCGTCAAGTTTACCGCCCGCAAT
CACACTACCACCGTCGCGGCCAACGCTCCCACCGTAACCTCTGCTGCTGCTGCTGCTT
CTGGACCATCCTCACCCTCCTCGCGGTGGCCCTCCTCGCTGCCATTGTCGGCGCCGCAC
TCTACGTACTCTACCGCCCTCACCGTCCCGAATTCTCCGTCACAAACCTCCGCATTGCA
AAGATGAACCTCACAACCTCCGCGGACTCACCTTCACACCTCACTACGCTCTTCAACC
TCACTCTCATCGCCAAGAACCCTAACAACCACCTCGTCTTCTTCTACGACCCGTTCTCC
GTGACGGTGCTCTCGAACTCTGTCCCCGTTGGGAACGGTTCCGTTACGGCGTTTACCT
CCGACAAGAACAACCAAACGAGCCTCCGCGCGGTGCTGTCGGGGTCGCAAGATCTGG
ACACGGACTCGTTGACTAGCCTGAGATCGGGGCTGAAGATGAAGAGAGGGTTCCCCG
TTGAGATTCAGATGGACACCAAGGTGAAGATGAAGATGGATTGGCTCAAGAGCAAGA
AAGTCGGAATTAGAGTCACCTGCGACGGAATCAGAGGAACCGTTCCCTCCGGCAAAA
CTCCGGCGGTGGCCTCCGTCGTCGACTCCGAGTGTAAGGTGGATCTTCGAATCAAGAT
CTGGAAGTTCTCCTTTTAA
>GmLEA2-29
ATGGAGGAGCGGGTGTCTTCGTCATCGCTACCGTCGCCATCACCCCCACCTCTTGAAA
AGAACCATAATACCGACAAACTCAAGTTACCAGACTTAGACCCTGGCACCTACGTTGT
CCAGGTCCCCAAGGACCAAGTCTACCGTGTTCCGCCGCCGGAAAACGCCCAAATCGC
CGAGAGCCATAAGAAATCTCCTCCCAACAAGGAAGCAAAAACGTCACGCTGCTGCTT
GTGTTGCGTGTTGTTCTTCATTATATTCTTGGTCCTTGCAATTCTTCTCGGAGCCGTTCTA
GGTGGTCTTTTTTCCATGTTACTCACACCAGAGGACCCTAAGTTTTCCATCACAAGCTT
TAAGGTCGTGGAGACCAAGCCACACCCCAAATACGACGTCACTTTGGAAGTCCACAAT
CCAAATTCCGACGTGGGAATTTTGTATAATGGCAAAGGCCATGTCTCCCTCTCTCTAAG
GCGACAAGAGAATATTGCTTCAGGAGCATACCCTACTTTCCGTCAAGACTCTCACGATA
CGACAACGTTTGGGCTAACTTTAACATCATCCTCAAAAGCCGGTCTCCCTAAGGAGGT
GGAGGAAAGCGTCAGGAACGATAAGAAGAAAGTGAGTGTGACATTCTCGTTGGCCAT
TCACGCCCTAGCACACATGAAAATGGGCTTGTTGCGTAGTGGGACAATGAAATTCGAT
GTTACGTGCAAAGTGAAGTTGGATACGTTGGCCAAAACCACTCACGTACTCTCTCAAC

AATGCGAAACTAAACGACACTAG
>GmLEA2-30
ATGTCTAAAATAACAATAACCTCTCCAAAACACTGTGCTGACAAAGAAGGACTAAAAA
TCAAGAACTACAAGAAGATCTACTTCACTTTCTCAGCATTTTTCATCACAATCCTACTAC
TAATACTTGTTATATGGCTCATCCTCCACCCTGCAAAGCCTCAATTCTCCCTCAAAGAAG
TCGACATCTTCCAACTTAACCTTTCAGGCCCAAACCTCAACTCCTCCATCCAACTCACC
CTCCTCTCCAAAAACCCAAACCAGAAAGTTGGCATTTACTACGACGAGATTCAACTCT
ATGCAACCTACAAGGGCCAACAAATTACTGGTGATACCCCTGTGCCACCCTTTTATCAA
GGCCAAGAAGAGAGTAATCTCATAACAGCTTCTTTGGTGGGAAATGCTTTACCCGTGG
CTCCGTCTCTTGGCTATGAACTTGGTCGTGATCAAATTGTTGGAAGACTAGTCTTGAAT
CTCAAAGCCAATGGAAAGCTTCGTTGGAAAGTGGGAACATGGGTCTCTGGACGTTACA
GCATTTTGTCACGCATTGATTACCAAGAGCTACTCTTTCATCTATGCGTCTATGCCAGCA
TTTTCGGAATTCATTGTTACATGCATATTCTGAAGCTGCCAGTTGAATATTTTGTGCTTA
ACCATTTGTCCCATGAATACTGA
>GmLEA2-31
ATGAGTCGACACAATATTAATCCAATAGGATCACAATTTTTCTTGCGTACACCCGCATTG
TTTTTTCCCCACAAGCAAGTTTGCAAAGGCAATAATCTCTATTACCCTCATTATCCAAAA
TCAGAAAAAATGGCCACTAGAGGCCTCAAAATTTGCTTGGCCGTGTCCTCTCTTTTCTT
GATCATTCTTGCCATCGTTATTGTGACCTTAATTTTGACCATCTTTAAACCCAAGAACCC
AGATATATTTCTCCACCCAGTTGACCTAGAAAACTTTCAATTGCTTTCACCTAATACAAC
TAGTGCACCCTTAGGCATAGTGATCACAATTGTGAACCCTAATTATGGAAACTTCAAGT
ACGTGAATTCCAGTGGCTATCTTAAATATCGTGACACCATTATAGCCGAAGTTCCATTGG
GGATAAGATCATTCCCTGCTCGTAGCACTACCAATGTGAGCACTACTGTGGGTATTATG
ACCGATAAATTGATACAAGATCCAAAGTTTTTGTCAGATATTGAAGGTGGGGTGTTCAA
TTTGACAGCAGAGGCCACACTTCCTGGGAAAGTGACCATGATCAAGATTTTAAGGCTT
AAGGCCAAGATTTATATCTCTTGTGGCGTCTCTTTCAACATAATTGCTGTGGATGCTAGT
TCCAGCTGCATGTCCAAAATCAAATTGTGA
>GmLEA2-32
ATGACTGATAGGGTTCACCCTTCGGCCAAAACCACCGCCAACGCCGGCCCCAAGCCGA
CATTCCCCGCTACGAAATCCCAGCTTTCCGGCGCCAACCGCCCCACCTACCGCCCCCA
ACCGCAGCACCACCGCCGCCGCCGTAGTCGCGGATGTGCCTCCACCCTCTGCTGCTGG
CTCCTCCTGATCCTCCTCTTCCTCCTCCTCCTCGTCGGTGCCGCCGGCACCGTCCTCTAC
TTTCTCTACCGTCCCCAACGACCCACATTCTCCGTCACCTCCCTAAAACTCTCTTCCTTC
AACCTCACCACTCCCTCCACCATCAACGCCAAGTTTGACCTCACTCTCTCAACAACTA
ACCCTAACGACAAAATCATCTTCTCCTACGACCCTACCTCCGTATCCCTTCTCTACGGCG
ACACCGCCGTCGCCAGCACCACCATCCCCTCCTTCCTCCACCGCCAAAGGAACACCAC
CGTGCTCCAGGCTTATGTTACTAGCACTGAGGAAGTGGTGGATAGTGACGCCGCGATG
GAGCTGAAGAGGAGCATGAAGAGGAAGAGTCAGCTGGTGGCGCTGAAGGTGGAGCT
GGAGACCAAGGTGGAGGCCCAGATGGGCGTGTTCCAGACGCCTCGAGTCGGGATCAA
GGTTCTGTGCGACGGCGTCGCCGTATCTCTCCCCGACGATGAGAAACCGGCGACGGCG
TCGGCTGAGAATACGGCGTGCCAGGTGGATGTGAGGTTTAAGGTCTGGAAATGGACCG
TTGGATGA
>GmLEA2-33
ATGCAGATACAACATCAAGAAGACCATAACCATAACCCTAATCACATCCTTCGCCCTAA

CCCTAACCCTAACCCTAACCCTACACAATACACACATTACCGCTTCCCTGGCAAAGTTC
CCCACCACCAACACCAAGTCCACAACATGGACAAAGGCGCAGCTTCACGCTTCAAAC
CCAATGCCCCCAAACGTGAACACTGCATTTGCATCACCGTTTTCCTCCTCCTACTCGGC
ATCATACTCCTCGTTCTCTGGCTGGCCTACCACCCTACCAAGCCGCGCTTCACGGTGGC
CAGCGCCGCCGTCTACGGCCTCAACGCCACCTCGCCGCCACTCATGTCAATCGCCATG
CAGTTCAACATGGTCATAAGAAACCCTAACAGGCGCGTCTCAATTTCCTTTGACAGGC
TCTCCGCCTACGTGTCCTACCGCAACCAGCCCGTTACGCCGCATGTCATGCTTCCGCCG
CTCTTCATAGAGAAGCACAGCGCCGTGTCGCTGTCCCCGGAGATCGGAGGCGTGCCGG
TGCCGGTGTCGGAGGACGTCAGCAATGGACTGGCCATGGACGAGAATTATGGGGTGGT
GGGCGTGAAACTTGTGTTGTTTGGAAGGTTGAGGTGGAGAGCTGGTGACATAAACTCT
GCACATTATGGATTGTATGTCAAGTGTGATGTTTTGATGGGTTTGAGGAAAGGTTTTGT
GGGTCAGGTTCCTCTTCTTGGTGCTCCAGTTTGTGATGTTAATACATGA
>GmLEA2-34
ATGGCGCACGCGAAGACCGATTCCGACGTCACGAGCATGGACACGTCATCGTCGCCAA
AACGCGCCGTTTACTACGTGCAGAGCCCCTCGCGCGACTCTCACGACGGCGACAAGTC
GTCGACGGCGACGCACGCGACGCCGGCGTGCAACAGCCCCGTGGACTCGCCCTCGCA
CCATTCCTACGGGCACCACTCTAGGGCTTCTTCTTCGAGCAGGGTCTCCGGCGGGTCCT
ACAACATCGCCTCTTGGGGGAGGAAAGTGACCCGCAAAAACAAATTAGGGTGGCCTC
ATGAGTGCAAGGTCATTGAGGAAGAAGAGGAAGGGTATTATGGGGAAAGACAAGGCT
TTTCTAGGAAGACTCAGATCTTCGTTGGGGTCCTTGGCTTTGCCTTCATTTTCACCCTCT
TTTGTTTCATCATTGCTGCGGCTGCCAGGCCTTATAATGTTCGGATCAGTGTTAAGAGTT
TCACAGTACACAACTTCTTATTCGGAGAAGGGTCGGATATGACAGGAGTTCCAACCAA
GATGCTGACGGTAAATTGTTCGGCGAGAATGACAGTGCATAACCCTGCAACCTTCTTC
GGAATTCATGTCAGCTCTAAAGCAGTGAATCTTATGTATTCTGAGATGACAGTTGCAAC
TGGTGAGCTGAAGAAGCATTATCTATCGAGAAAGAGTACCCGTACAGTGTCTGTGAAC
CTGCAAGGAAGCAAGGTTTCTCTATATGGTGCTGATGCAAGCTTAACTGGCTTGGTGG
ACAATGGAAAAATACCAATGACTCTTGTCTTTGAAGTTGGATCGCTAGGGAATATTGTT
GGGAGGTTGGTGAGGTCCAAACATCGAAGGCGTGTCTCTTGCTCAGTGGCTATTGATT
CCCACAACATCGAACCCATCAAACTTAAAGAGAATGCATGCACATACAATTGA
>GmLEA2-35
ATGACCGGGAAGGACGAGTTCATCCGCCTTACCCGAATCCGACTCAACCGCATCGGGA
TCCGAACCAACCCGAGGCCCATTCTCGACCTCTCCTTCTCCATCACCGTTAAAGTCCAC
AATAGAGACTTCTTCTCCCTCACCTATGACACCCTCGCCGTCTCCGTCGGCTACCGCGG
CCGCCAGCTCGGCTTCGTCACCGGCGGCGGCGGCGGCCGCATCAGGGCGCGTGGCTC
CTCCTACGTCGACGCCACGCTCACCATTGACGGATTTGAGGTTATCTACGACGCGTTCT
ACCTGCTCGAAGATATCGCTAAGGGCGTCATTCCATTCGATACTGACACCCGAGTTGAA
GGGAAATTAGGGCTTTTCTTCTTCACTGTTCCTTTGAAGGCAACAGTGTCGTGCGAAG
TGTATGTGAATATAAACCAGCAGACAATTGTACGTCAAGACTGCTATCCTAAGATCTTTT
GTAGCTTAACTATTATGCAATAG
>GmLEA2-36
ATGCACGCAAAGACAGACTCAGAGGTAACAAGCCTCGCCGCGTCTTCCCCCACGCGC
TCCCCTCCACGCCGTCCTCTCTACTATGTTCAGTCTCCCTCACGAGACTCTCACGACGG
CGAGAAAACCGCAACGACGTCGTTTCACTCAACGCCCGTTCTCAGCCCCTCTGCCTCT
CCTCCTCACTCGCGCCACTCCTCCTCCACGCGCTTCTCCAAGAAAGACCATAGCCACA

GCCTCAAGCCGTGGAAGCAAATCGATGTTATTGAAGAAGAAGGGCTTCTTCAAGGGGA
TGATCACCATAACGGGCTTCCACGTCGGTGCTACTTTCTCGCGTTCGTTGTTGGGTTTT
TGGTTCTCTTCTCCTTCTTCTCGCTTATCCTTTGGGGGGCTAGTAGGCCCATGAAGCCCA
AGATCAACATCAAGAGCATAAAGTTTGATCATGTTAGGGTCCAAGCAGGTTCGGATGC
AACAGGGGTAGCCACCGATATGATTACATTGAATTCCACTTTGAAATTCGCATACCGCA
ACACAGGCACATTTTTTGGGGTCCATGTCACATCCACACCCGTGGAACTGTCCTATTCA
GACATTGTAATTGCTTCGGGAAATATGAAGAAGTTTTATCAATCTAGGAGGAGCCAAAG
GTTGGTGAGTGTAGCAGTGATGGGTAACAAGATCCCTCTATATGGAAGTGGTGCTAGCC
TAAGTAGCACAACGGGTGTGCCCACTGTGCCTGTGCTATTGAACCTCAACTTTGTGCTA
CGATCTAGGGCTTATGTGCTTGGGAAATTGGTGAAGCCTAAATACTACAAAACCATTCA
ATGTTCCATAACTTTGGATCCAAAAAAGCTCAATGCTGCGATTTCCCTCAAGAAATCTT
GCACATATGATTGA
>GmLEA2-37
ATGTGTTGCAGAAACCGCTTCATCGCCTGCACCTGCTGCATAATCATCACCCTCTTCTTC
ATGTTCATAATTTCCATCATCGTCTGCATCTCACCCTCAAGTGTCAAGTTCCATGTAACC
GATGCCTCCCTAACACAATTCAACCTCACAAGCAACAACACTTTGTATTACAACTTGAA
GGTCAACGTCACAGTGAGAAACCCCAACAAGCACACCATAGTGTACTATAGGAGGATC
ACAGTAATTTCTTGGTACAAAGATAATGCTTTTGGTTGGGTGAGCTTAACACCCTTTGA
CCAAGGCCACAAGAACACAACCTTCCTTCAAGCAGTGTTTGAAGGGCAGAGGGTGAT
TAAGCTCAAATCTAAACAACTTGGTGAGTATAAAGATGAGACAAGTGTTGGGATTTAC
AAGGACCTTGCTGTGGATTTTGATCTTAGAATCAGAGCCAAGTATGGAAAGTTCAAGA
GTAGTCGTTTCAATACACCCATTGTTCAGTGTCGCCGATTGAGGGTTCCTTTGATTTCTA
ATGGTAAATCAGTACCTCCTTTTAGTGTCACCAGATGCAAAAGTGCTTCTTTCTTCTCA
GATCGTGATGCGCCTGATGTTGATGGATAG
>GmLEA2-38
ATGTGTTGCAGAAACCGCTTCATCGCCTGCACCTGCTGCATAATCATCACCCTCTTCTTC
ATGTTCATAATTTCCATCATCCTCTTTTGGATAATCATATCCCCATCAAATGTCAAGTTCC
ATGTAACCGATGCCTCCCTAACAGAATTCAACCTCACAAGCAACAATACCTTGTATTAC
AACCTAAAGGTCAACGTCACAGTGAGAAACCCCAACAACAAAATCGTAGTGTACTATA
GGAGGATCAAAGCAATAGCTTGGTACAAAGATAATGATTTTGGTTGGGTGAGCTTAAC
ACCCTTTGACCAAGGCCACAAGAATACCACTTTCCTTCAAGCAGTGTTTGAAGGGCAA
AGAGTGATTAAGCTCAAACCTAAACAACTTGGTGAGTACAAAGATGAGACAAGTGTTG
GGATTTACGAGGACTTGGCTGTGGATTTTGATCTTAGAATCAAAGCAAACTTTGGAAG
GTTCAAGAGTAGTCGTTTCGATCCACCCGTTGTTCAGTGTCGCCGTTTGAAGGTTCCTT
TGATTTCTAATGGTAAAACTGCACCTCCTTTTAGTGTCACCAAATGTAAAATTGATGGTT
CTTTCTTTGCAGATCGTGATTCTCAAGCAGAAGCTAGATAG
>GmLEA2-39
ATGTGTTTCAGATCGCGTTGCAGCTTCTGCTATATCTTCTTCACAATCTACACCCTTATG
CTCATGTTCATTCTTTCCATCATCCTCTTCTGGATAATCATCTCACCCTCAAGTGTCAAG
TTCCATGTAACCGATGCCTCACTAACACAATTCAACCTCACAAGCAACAACACATTGTA
CTACAACTTCAAAGTCAACGTCACAATGAGAAACCCCAACAACAACATCATAGTGTAC
TACAGGAGGATCACAGCAATTTCTTGGTACAAAGATAATGCTTTTGGTTGGGTGAGCTT
AACACCCTTTGACCAAGGCCACAAGAACACAACCTTCCTTCAAGCAGTGTTTGAAGG
GCAGAGGGTGATTAAGCTCAAATCTAAACAACTTGGTGAGTATAAAGATGAGACAAGT

GTTGGGATTTACAAGGACCTTGCTGTGGATTTTGATCTTAGAATCAGAGCCAAGTATGC
AAAGTTCAAGAGTAGTCGTTTCAATACACCCATTGTTCAGTGTCGCCGATTGAGGGTTC
CTTTGATTTCTAATGGTAAATCAGTACCTCCTTTTAGTGTCACCAGATGCAAAAGTGCTT
ATTTCTTCTCAGATCGTGATGCTGCTGATGGATAG
>GmLEA2-40
ATGGCTCAGAGCCCTGATCAGCAGATGAAGCCCTTAGCTCCATTCGTATCATCAACAAG
CCACTTCAGCAGACAAGAAGATCATGATCAGTTTCAAGACACATCCGAACAGAAAATC
ATTCGCATAAGAAAGTTCGTGTTGTGCTGCGGTTGCTTCACTGCCCTTGTCGTGATACT
TGTGGTCGTACTCATAGTCTTGGGCTTCACCGTTTACAATGTCAAAGAACCCGAGGTGA
GTATGAATTCGGTTACTCTTGTAAGTGGAACTTTTGCAAACAGTGGTGCCACAAACAA
CGTTACACTTCTTGCTGATATCTCTGTAAAGAACACAAACGCTTTTACCCTCAGGTTTG
GAAAAACTACCACCATTGTTTACTATGGTGGTATGGGAATAGGTGAGGGTACTTCTCCA
CCGGGTAAGGCCAAGGCAAGAAGGACCATAAGGGTAAACTCGACCTTGGAGATTATG
GCAAAGAAGCTTTTAGATATCCCAACTTTGAATATTGACCTCAGGGATCAATCTTTGAAT
ATCAGCAGCTATACCAGGATAGATGGTAAGGTGAAAATACTCAACATGTTTCCGAGGAA
GGTTGTGGTTGAGCTTAATTGCACCATTGGATACAACATCACCACTGGGTTGGTCACAA
ATGGTGACAATTGTCTTGGAACCGTTGATATTTAG
>GmLEA2-41
ATGGCCGCCGGAGCCGCGCCGGTACCGGCGCCGAGGCCGAAGCGGCCTCGGCCTCCG
TCGGGGCGCACCAACCTCGCCTCCTGCGTGGTGGCCACCATCTTCCTAATCTTCATCGT
CATCGTCATCCTCATTGTCTACTACACCATCTTCAAACCCCAGGACCCCAAAATTGCCG
TTAACGCCGTCCAGCTCCCCTCCTTCTCCGTCGCTAACGGCACCGTCAACTTCACCTTC
TCCCAGTACGCCTCCGTTAGAAACCCTAACCGCGCCGCATTCTCGCACTACGACAGCT
CCCTCCAGCTCATCTACTCCGGCAGCCAGGTCGGCTTCATGTTCATCCCCGCCGGCGAG
ATCGACGCGGGCCGGACGCAGTACATGGCCGCCACCTTCTCCGTCCAGTCCTTCCCCC
TCTCGGCGCCGCCTCGAATGGGCCCCACATTGGCCAACGGCGACGGAGTGGGCTTCA
ACTATGGGCTTAGGGTTGAGCCCACTTTGGAAATCGAGTCTAAGTTGGAGATGGCGGG
GCGCGTCAAAGTGTTGCACTTTTTCACCCATCACGTTTACGCCAAAGCCGGTTGCAGG
GTTGCCATTGCCGTAACTGATGGATCTGTCTTAGGTTTTCACTGCTAA
>GmLEA2-42
ATGAACACAAGAGCGGGAGCTAACTCAGAATCCCAGCCACAGCCATCAAGACGACAA
CAGCACCTCCACTACGCCAGCTCCCCAACGACGTCGTCTCATTCTTCATGCTGCTGCTG
CCTCTTCCTCCTCTTCTCCTTCTTCGCTCTCTTAGTCCTCGCCGCGCTTCTCGTAATCGT
GCTCGCCATCAAGCCCAAAAGCCCGCACCTGGACCTCCGCCAAGTGGGCCTCCAGTAC
ATGGCCCTCGTGCCCAACCCAACTCGCCCAGGCCCATCGACCGCGAAGCTCTACCTCG
TGATCCGGTTGGTGCTGGCGGTGGTGAACCCGAACGAGGTGGGGATCAGGTGCGGCG
AGTCCAGAGTCACGGTCGTGTACCGGGACACACCCTTGGGCAGGACCTCCCTGCCCG
CGTTCTGCCAGCGCGCTCACACCGTGAAGGAGGTGGTGGCCACCATGGCAGTGGATG
ACGTTAACTTGTCCAACGCCGATGGTGCTGACTTCGCCAGAGACGCGTTGCTCAATGA
CAGGGTCGAGCTGAGGGTGTTGGCCCATGTCGCCACCAAGATTCGCCTCTTCAACCTA
CCATCTCCTCCACTTCAGGTCTCAGTGAATTGTGTAATAGTGATAAGTCCAAGAAAGCA
ATCTCTAACTTACAAGCAGTGTGGATTCGAGGGATTGAATTAA
>GmLEA2-43
ATGGAGGGGCACGCCGACCACAACTCCGCCGTGTTGCCGCCACCTCCTGGCCGCCAC

GCCCCAGCCGCAGGCACGTACATCGTGCAGTTCCCGAAGGACCAAGTGTACCGCGTTC
CACCACGCGAGAACGCGTTGATCGTGGAACAGTACCGCAACCCGGCCACCGCGAAAA
AAAGACGCGGCGGTTGTTGTTGCTGTTGCAACCGTCGTGTCTTGATAACCTTCGCTCTC
GTGGTTACCACAATCGTTGCCGTGGTTGGCATCACGTTGGCCACGTTGTACTTTATTTTC
AGCCCCGCGGGTCCCAAGTTTACTGTCTCCCATGTGGCGGTGAATAGGAACAACAAGA
ACTCGCAGGGGGCGGCGGCGCAGTATGAGGTTTCGTTGAGAGCGAGGAATCCGAACG
AGAAGTTGGCGATTCAGTACCAGGAAGGGGACGTGTCGCTGTTGTTGTTCGATGAGAG
TAAGGTTGCCGAGGGGAAGTTTCCAACGTTGGAGCAAGGGGGAGGTGAGGCGAGTG
AGGTTAAGCTTGAGCTGACCGGATCCAGTGGAGCGTTTCCCAGGGGAATGCATGGTGG
GGACGCAGCCGTGGATTTGAAATTGGAGATCAAACTTGCGATAAGGATTAGGACCGCG
GGTCTCGAAACGTGGGGTATGAGCTCCAATGTGGCGTGTCAGTTTAAGGTCAGTGGTC
TCGGTAACGACACGAGAATCTTGTCACAACAATGTGATACCAAATTCAAGCAATATTGA
>GmLEA2-44
ATGACCACCCCAAGAGGAAACCACCACCCACAGCCTAACGGTGAGCGAAGAAGACAA
TGGCCACCGCCACAGCCACCAGCAGCGGCACCGGAAGCGGCAGCGGCACCGACAGC
AGTAGCCGCAAATTACAATGGATACCGACAGTACCACCCGACAACGCCAGCTCGTTCG
TCGTCGTCGGCGTCGTTCAAAGGATGCTGTTGCTGTCTGTTCCTTCTATTCTCGTTCCTG
GCGCTGTTGGTGCTAGCGGTGGTGCTGGTAATAATCCTAGCGGTGAAGCCGAAGAAGC
CGCAGTTCGATCTGGAGCAAGTCGGGGTGCAGTACATGGGCATCACACCCAATTCCCC
CTCCACCGCTTCCCTGTCTCTCACCATTCGCCTCCTCTTCGCCGCCACCAACCCCAACA
AGGTCGGGATCAGGTACGGCCAGTCCAGCTTCACCGTCATGTACCGCGGCATCCCCCT
CGGAAAAGCCACCGTCCCCGGCTTCTTCCAGCAACCTCACAGCACCCGACAGGTGAT
CGCCACCATCGCCGTCGATCGCGTCAATTTGCTTCAGGCGGACGCCGCCGATTTGATCC
GAGACGCCTCCCTCAACGACCGAGTCGACCTGAGAGTCTTGGGAGACGTTGCTGCCA
AGATCCGCGTCATCAACTTCGATTCTCCTGCTGTTCAGGTTTCGGTGGATTGTGCTATAG
TGATCAGTCCAAGAAAGCAATCTCTGACTTACAAGCAGTGTGGGTTCGATGGATTGAC
TGTTTGA
>GmLEA2-45
ATGAAAAAGGGATTTGGCTGCAAAGGAAGCATTATGTGCCTAGTGGTAGCATTATGTGT
AGCGATTGCACTTGTATTGTTAGGGGTGATCTTAGCATTGACGGTGTTCAAACCAAGGC
ACCCCATTACCAATGTGGATTCAGTAAGGCTTCAGAACATGAGCTTGGCCATGGACATA
TTTAGCATGAGTGTAAATGTGAACTTGACATTAGAAGTGGATGTTTCAGTCAACAACCC
TAATAAGCTTGGATTCAATTACTACAATAGCTTTGCCCAACTCAACTATAAAGGGCAGTT
GATTGGGGAAGCTCCTATCCCTAATGGAGACATATTAGCTGAGGAGATTAAGGGACTCA
ACTTGACACTCATTGTTATGGCTGATCGTTTGGTCTCCAATTCTAATGTCACCAAGGATG
TTGCATTGGGTTCATTGCCCCTCAACACCTTAGTGAGAATTTTTTGCCAAGTCAACATC
TTAGGGTTTATGAAATTCTATGTGGCTTCCACCTCATCACCAAAAATTCCCCATTTCCTC
GACTTCAAGAATTTGTACAAAGGAGCAAAGTTGTCCCCATTCACTTCAATTTAG
>GmLEA2-46
ATGCACGCAAAGACAGACTCAGAGGTAACAAGCCTCGCCGCGTCTTCCCCCACGCGC
TCCCCTCCACGCCGTCCTCTTTACTACGTTCAGTCTCCCTCGCGAGATTCTCACGACGG
CGAGAAAACCGCAACGACGTCGTTTCACTCCACGCCCGTTCTCAGCCCCTCTGCTTCC
CCTCCTCACTCGCGCCACTCCTCTTCCACGCGCTTCTCGAAGAAGGACCATAGCCATAG
TCTTAAGCCGTGGAAGCAAATCGATGTTATTGAAGAAGAAGGGCTTCTTCAAGGGGAT

GATCGCCGCAACGGGCTTCCACGTCGGTGCTACTTTCTTGCTTTCGTTGTTGGGTTTTT
GGTTCTCTTCTCGTTCTTCTCGCTTATCCTTTGGGGGGCTAGTAGACCCATGAAGCCCA
AGATCACCATCAGGAGCATAACGTTTGATCATGTAAGGGTCCAAGCTGGTTCGGATGC
AACAGGGGTAGCCACCGATATGATCACATTGAATTCCACTTTGAAATTCACATACCGCA
ACACAGGCACATTTTTTGGGGTCCATGTCACATCCACACCCGTGGAACTTTCTTATTCA
GACATTGTAATTGCTGCGGGAAATTTGAAGAAGTTTTATCAATCTAGGAGGAGCCAAA
GATTGCTGAGTGTATCAGTGATGGGTAACAAGATCCCTCTATATGGAAGTGGTGCTAGC
CTAAGTAGCACAACGGGTGTGCCAACACTGCCTGTGCCATTGAACCTCAGCTTTGTGC
TACGATCTAGGGCTTATGTGCTTGGGAAATTGGTGAAGCCTAAGTACTACAAAACCATT
AAATGTTCCATCACATTGGATCCCAAAAAGCTCAATGCTGCTATTTCCCTCAAGAAATC
TTGCACATATGATTAA
>GmLEA2-47
ATGATCGGGAAGGACGAGTTCGTCTCTTACTCGCCCCTTCCCTCCAACCCTAACCCTAA
CCCTAACCCTAACCCTAACCCCAACCCCAACCCTTATCCCAATCCTAATCCTTATTACGA
CCCTAACCCTCATCCTTACCCCCAAAACGTCGTCGTTTTGCTCCCCTCCTACCGTCCCCA
TTTCCAGCGTCGTCGCCGCCGCTGCATCATCTACTCCGCGGCACTCTTCCTCTTCCTCCT
CGTCGCTGGCGCCGCCTTCCTCCTCTACCCCTCCGACCCGGAGATTCGTCTCGCTCGGA
TCCGACTCGACCGCATCGGGATCCGAACCAACCCGAGGCCCATTCTCGACCTCTCATT
CTCCCTCACCGTTAAAGTTCGCAACAGAGACTTTTTCTCCCTTTCCTATGACTCCCTCA
CCGTCTCCGTCGGCTACCGCGGCCGCCAGCTCGGCTTCGTAACCGCCGGCGGCGGCAG
CATCAGGGCGCGCGGCTCCTCCTACGTTGACGCCACGCTCACCATCGACGGATTTGAG
GTTATCTACGATGCGTTCTACTTGCTCGAAGATATCGCTAAGGGTGTGATTCCGTTTGAC
ACTGACACGCGAGTTGAAGGGAAATTAGGCCTTTTCTTCTTCACTGTTCCTTTGAAGG
CAACAGTGTCGTGCGAAGTGGATGTGAATATAAACCAGCAGACAATTGTACGACAAGA
CTGCTATCCTAAGTCACTAGGTGATCCGCTGGATGAGAGCGCATATATTGAAGCTGGAG
ATACATGA
>GmLEA2-48
ATGGCGCACGCCAAGACCGATTCCGACGTCACCAGCATGGACACGTCATCGTCGCCAA
AACGCGCCGTTTACTACGTGCAGAGTCCCTCGCGCGACTCTCACGACGGCGACAAGTC
ATCGACGGCGACGCACGCCACGCCGGCGTGCAACAGCCCCGTGGACTCGCCGTCGCA
CCACTCCTACGTCCACCACTCTAGGGCTTCCTCTTCGAGCAGGGTCTCCGCCGGCTCCT
ACAACAATATCGCCTCTTACTGGGGGAGGAATAATAAAGGGACCCGCAAAAACAAATT
AGGGTCATGGACTCATGATCAGTGCAAGGTCATTCAGGAAGAGGAAGGGTATTATGGG
GAAAGAGAAGGCTTTTCTAGGAGGAAGACTCAGATCTTCGTTGGGATCCTAGCCTTTG
CCTTCATTTTCACCCTCTTTTGTTTCATCATTGCTGGGGTTGCCAGGCCACACAAAGTT
CGGATCAGTGTTAAGAGTTTCACAGTACACAACTTCTTATTCGGAGAAGGGTTGGATTT
GACAGGAGTTCCAACCAAGATGCTGACAGTAAATTGTTCAGTAAGAATGACAGTGCAT
AACCCTGCAACCTTCTTCGGAATTCATGTCAGCTCTAAAGCAGTGAATCTTATGTATTCT
GAGATGACAGTTGCAACTGGTGAGCTGAATAAGCATTATCTACCAAGAAAGAGTACCC
GGATAGTGTCGTTGAATCTGCAAGGAAGCAAGGTTTCTCTATATGGTGCTGGTGCTAGC
TTAATTGGCTTGGTGGACAATGGAAAAATACCAATGACTCTTGTCTTTGATGTTAGATC
GCGAGGGAATATTGTTGGGAAGTTGGTGATGTCCAAACATCGGAGGCGTGTCTCTTGC
TCAGTGGCTATTGATTCCCACAACATCAAACCCATCAAACTTAAGGAGAATGCATGCAC
ATACAACTGA

>GmLEA2-49
ATGCCGTCCCACGATCACGTGGCCGAACGCCAGCACCATAGACCAATCAGAAGAAGAT
TGGTGAACACTCACCACAGCGGCAAAACACACCCTCTAATCTGGCTAGCAGCCATTCT
CTGCACCATCATAGCCATAGGAGTGGTGATAGCCGGCATAGTAGTGTTCGTGGGTTACA
TGGTGATCCACCCTAGAATCCCAGTTATGAGCATCACCAACGCCCACCTCGATCTTCTC
AGCAACGACTACGCAGGGCTCCTCCAGACGCAGCTCACCATCATCGTCGTGGCTCAGA
ACGGCAACGCCAAGGCCCATGCAACCTTCTCCGACATTAGGTTTAACCTCAGCTACCA
GGGACAGAGAATTGCGCTCATGCGCGCTCCGCCGTTCGATGTCCCCAAGAACAGCTCC
AAATCACTCAACTACGTTGTTCGGGCGGCTTCCATTCCCTTAACTCCCCAACAGATGGA
GGAGGTGGACGAGTCGTGGAAGCGTGACGTCATTGGCTTTGACTTGAAGGGCAGCGC
CAGGACTCGGTGGAGGGTTGGCCCTTTAGGTTCTGTTAAGTTCTGGTGCAATTTGGAG
TGTCAGCTCAAGTTTCATCCTTCTAATGGCTCTTACATTCATCACTCTCGCTGCACCTCT
AAGTCTAAATGA
>GmLEA2-50
ATGTCCCATCTAAACGGCGCCTACTACGGCCCCGCCGTTCCCCCGCCGAAATCCTACCA
CCGTCCCTCCCACGGCGGCGGCGGCCGCGACGGCTGCTGCTGCGGCTGCCTGAGCTG
CTTCTGTGGCTGCATCTTCGACTGCATCCTCGGCCTAATCTGCAAGATCCTGACCACAA
TCATCATAATCCTGGCAGTTCTCGCTTTTCTGTTCTGGTTTATCGTGCGCCCCAACGTGA
TCAAATTCCACGTCACAGACGCCTCCCTCACGCGCTTCGACTACACCACCAACAACAC
TCTCCACTATGACCTCACTCTCAACGTGTCGATCCGCAACCCGAACCGCAGGGTCGGC
GTCTACTACGATCAAATCGAAGCGCTCGCGCTGTACGAGGATGTTTTGTTCGGGAACCT
GACATTGGGTTCTTTCTACCAGCACCACAAGAACACCACCTTCGTGAGTCCCATTTTCA
AGGGCCAGCGCGTGACTCCACTCGCCAAGGTCCAGGTTTCGGAGTTTGACAAGGAAA
AGGGTTCTGGGGTTTACACCATAGATTTGAAGCTCTTCATGACGGTTCGGTTCAAGTTC
TTGCTGTTCAAGAGTGGGAGTTTGAAGCCCAAGATTCGGTGTGCGTTGCACGTGCCAC
TGAAATCGCGTAACGCAACAACCTCTCCGGACGCTGCGTTTCAACCCACTGAGTGCGA
TTGGGATTACGGGAAAAAGTGGTGGATCCATTAG
>GmLEA2-51
ATGGCTGATAAGCAATCCCAACTAAACGGTGCGTATTACGGCCCCGCAATCCCTCCATC
GGAGCTGCCACGTCACCACCACCGTGGCCGACGCTGCTGCTGTTGCCTCTTCAGCTTC
TTCTGGAAGCTTCTCCTTGCCATCGTCGTCTTCCTCGTTGTCGTATTCCTCGTCTTCTGG
GCTGTGGTCCAGCCACGCACCTTCAAGCTCCATGTCACCGACGCCAAACTCACCCAAT
TCAACCTCACCACCAACAACAATAATAACATTAACATGCTCCGCTACAATCTCGTTCTC
AATCTCACTGCACGAAACCCTAACAAAAAACTCAACATCTACTACGACCAAGTGGAGG
GCCACGTGTCCTACGAGGGGATGAGGTTCGCCTCAGCGGACCTGATCACATGGCGAAA
CTCCTTCCGCCAATACACAAAGAGCACGAACAACATGAGCGGGGTTTTCACGGGGCA
GCACGGGTTGGTGTTCGATGGCGATCACGTGAAGGATTTCGAAAAAGATGAGAGAGG
TGGGGTTTTTGATATTGATGTGAAGCTTTACTTTAACATTAGGTTTAGGCTTGGTGACTT
CATAGGAAGTAGCTCAAGGGTTAGGGCGAAGTGCGAGCTTCAGGTTCCTTTGGTTTCG
AGGGGAGTGTTTCTTCCCACCAGGTGCCATGTCAAATTCTGA
>GmLEA2-52
ATGTCGGTGAAGGAGTGCGAGCACCACAAGGGGAAGAAGAGGAAGATCTTCCGGCA
AGTGTTCTGGTGCTTAGTGGTGTTCCTTTTCATCGTGCTGGTCACAATTCTTTTGATATG
GGCAATCCTTAGACCCACCAAACCCACCTTCACGCTCCAAGACGTCACCGTCTACGCC

TTCAACGCCACCGTGGCGAACTTCCTCACTTCGAATTTTCAGGTCACGCTCATCTCGCG
TAACCCGAACGACCGCATCGGTGTTTACTATGATCGCCTCGAAACCTTCGTGACCTACC
GGAGCCAGCAGGTCACGTACCGAACCGCCATCCCTCCGACATATCAAGGCCATAAAGA
GATCAATGTTTGGTCTCCGTTTGTTTATGGCACCAACATCCCCGTCGCGCCGTTCAATTT
CCTCGGCCTCAGCCAGGACCAGAGTAACGGTAACGTCCTCGTCACCATCCGAGCCGAG
GGTAGGGTTCGTTGGAAGGTCGGCACCTTCATCTCCGGCCGCTACCACCTCTACGTCC
GCTGCCCGGCGTTCATCAGCTTTGGACCCCGCAGCAATGGAATCGTCGTCGGAGAGAA
CGCCATCAAGTTCCAGATTATCCAACGGTGCTCCGTTAGTGTCTAA
>GmLEA2-53
ATGGCAGATCATCAGAGACTGAGGATCCACCCTATGGAGGGGGAAGCACCACCGCCCC
CAACAACTCCATTGGTCCCTCCAGGCTCATCAAGATCAGAAAAGGGTGTTCCTTTGCA
CCATCCTCCACCTCTACCACGTGCTATGCCAGCAGCATACCCTACACCACACAAAAGA
AGCTCAAGCTGTTGCTGCAAGTGCATATGCTGGACAATAATCTTGGTTGTTCTCCTCCT
CATTATCTTTGCAGCAAGTGTTGGAATCCTTTATCTAGTCTTCCAACCAAAGCTTCCTGA
TTACTCAGTTGACACTCTCAGGATAAGTGATCTGAGGCTTAACTTTGACATGAGCCTCT
ATGCAAAGTTTGATGTGAAGATCACAGCAACCAACCCAAACAAGAAGATTGGCATCTA
CTATGAAAAGGGTGGAAAGTTGAGTGTGTGGTACACAAACACAAGGCTCTGTGAAGG
GTCACTGCCACAATTCTACCAAGGTCATGAGAACAAAACAGTGCTCAATGTGTCCTTG
AGTGGTCAAGTGCAGTCTGGAAGCACCCTAATGAATGCACTGCAGCAGCAACAGCAG
ACAGGGCGCATTCCATTGGATCTCAAGGTGCATGCACCAATAGCCATCAAACTTGGGA
GGTTGAAGCTGATGAAGGTGAGAGTGTTGGGAGAATGCCTTTTGGTGGTGGATAGCTT
GTCATCTAATAATCTCATAAGCATCAAGGCTAGCAACTGCAAGTTTAGATTGAAACTTT
GA
>GmLEA2-54
ATGCACAACAACGACCACATACCCGTTCACCACGTGCAGGGTTCCAACCCGAAGCCCG
TGAAACTGAACCGGCACCACACGATGCGGTACTACGTCCACCGGGTCCACGAAAGCC
TCACAACCCGCGTCTCCAAGATGATCTGCGCTACCTTCTTAGGCCTTCTTTTTATTGTGG
GCCTCATCACCTTCATCCTCTGGCTCAGCCTCCGGCCCCACAGGCCCAGGTTCCACATC
CAGGAGTTCAACTTGCCGGGCCTGACCCAAAACTCCGGGTTCGAAAACGCCGTCATA
ACGTTCAAAGTATCCGCGCGAAACTCCAACCAGAACATCGGGGTTTACTACGAGTCCA
TGGACGGCGCCGTTTATTACCGGGACCAGAAAATCGGGTCCAAGCCGTTACTTTACCC
GTTTTATCAGCAGCCCAAGAACACGACGGAGGTGGACGGCGATCTTAGTGGGGCGAC
GTTGACCGTTAGTAGTCAGCGCTGGTCGGAGTTCCAGAGCGATAGGGCTGACGGTAGC
GTGGTGTTCCGCTTGGAATTGACATCTGTGATCAGATTCAAGATATCCACGTGGGACAG
CAAGCGCCACACGATGCACGCCAACTGTAATGTGGGCGTGGGACCCGATGGTTCCCTC
TTGACCCTTTACAAGGACAAGAGGTGCCCCGTTTATTTCTCTTGA
>GmLEA2-55
ATGTGTGAAGGCAAGAGTCTCTACATATGGCCCCTGAAAGTTATAGGCCTCTTAGGCCT
TATTGTTCTGTGCCTGTGGCTGGCCTTACGTCCCAAGAACCCCTCCTACTCCATTATGTT
CATCTCCATACAACATCCCTCCAACTCAAGTGAAAACTGCACCATCTTTTACAGCCTTC
AAATTGAAAACCCAAACAAGGACTCGAGCATTTACTATGACAAAACGATTTTAAGTTT
CTTATATGGGGAGCCTGAGGACGAGGTGGGGGAGACAACTATAGTCCCATTCCATCAA
GGAACTGGCAACACCCGGGATGTATCTGATACTGTTAATGCCAAACCTCGACCATTCAA
ACCTCTCTTCAGTGCCATCTCAAATGCAACCACAGAGTTAAAAGTTGCTTTGATCACAA

GATACCGATACAAGACATGGGGAATTAAGAGCAAGTTCCACGGGTTACAACTCAAAGG
TATTCTACCAATTGATTCCGATGGGAAGCTCTCACGTAAGAAGAAAAAGTACCCACTTA
GCCGTAATTCCAACAAACTAGGAAGGTTCAAAATAAGGCACTGA
>GmLEA2-56
ATGACAACAGAGGAAGTGATCACCATGTTCCCATCACGCAAAGAAGAAAAACAAAGC
AGCAGCAAGTGTTTGGTGTATGCTTTGGTTGTCCTTGTAGCCATATTGTTCATTTGGCTT
GTTTTTGCATCTATCGTGTTGCGCGTGGTTGATCCCCAGATTCAGTTGAAATCAGCTAGA
TTGATGCACAACACTAAAAATCATAGTTTTTCATCAACTTCTTCCTTGAACGTGACTATG
ATTGCTCGTGTGTCCCTCACGAATCCAAACTTGTTCGGTCGCTTCTATTACGGGAACAG
TAGAGTGAGTGTGCTATATGGGGCTTCTATAGTTGGTGCTTGGGAATTGGAAGGTGCGA
GATTGGAGGGTAGAGAAACCAAAGAAATCGATTTCATGGTTCACATGAGGTTCAGCAC
TAAGCTATTGGTAATAATGAGAAATCTAACCAATGACACTGCCCACTCTGACTCTGCAG
GCATGCTTAAACTCAGAAGCTACGCAAAATTAAGTGGTACAGTGCACGTGCTGAACAT
GGTAAATAAGAAAAAGACCTTAGGAATGGCTTGTATTATGAATCTCAACCTGACCTCAT
ATTCCACACAACATTTCCAATGCTAA
>GmLEA2-57
ATGGCCAAGTCCATAGAGCAAGAAGGAAGAAGCGGCAAGTGTTTCGTATATTTTCTGG
GCGCCTTTGTCATCTTATGCGCACTTGTATTGGTTTTTGCCTCCATATTGCGCCTCAAGA
ATCCATATCTCAAATTAAGATCAGCTACTTTCAACCAAATCCGTTACAGCGCTTCACCAT
CACCTTCCTTCAACGCCACCCTCATCATCTTTCTTGCCCTCAACAACCCAACTTTCGGT
GCCTTCACTTACGAGAATAACAGCCTCAGTGTGCTCTATGCGGGTCTAAAGATCGCTCA
TAGCCAAATCAACGGTGGCAGGGTGAGTTTCAGACAAACCAAAGAAATTCATGTTACT
GTGAAATTCATGTCCGCTATTGACATTACTTCGGGGTCGTTGAATCTCACCACTAATGTC
TTTTTTAGTGGCAAAGTTCATCTGTTTAAGATTATTAACATCAGAAAGACCATTGAAATG
CCTTGCTCTATGAACCTCAACTTTACCTCTCACGCAACTCAGGCTATTCAATGCCAATA
G
>GmLEA2-58
ATGGGAGGAAGAATGCACACGAAATCAGACTCGGAGGTTACGAGCAACAGCATGGAG
CAGTCGTCACCGTCGCGTTCCCCTCCGCGGCGGCCACTCTACTACGTGCAGAGCCCTT
CGAACCACGACGTGGAGAAAATGTCGTACGGTTCAAGCCCCATGGGTTCCCCTCACCA
CCATTTCCACTACTACCTCTCTTCCCCCATCCACCACTCCCGCGAATCCTCCACCTCTCG
CTTCTCCGCTTCCCTCAAAAACCCTCGCAGCAACTTCTCCTCCTCCTCCTCCTGGAAGA
AGCTCCACCCACACCCCAACCCCGACGCCGGCCTCGACGACGACGACGACGACGACG
ACGGCGACGACGACCTCAACCACTTCCCCCGCAACCTCCGCCTCTACTTCTGCTTCTTC
CTCCTCTTTCTTCTCTTTTTCACCCTCTTCTCTTTGATCCTATGGGGCACCAGCAAGAGC
TACAAGCCTCGAATCATTGTCAAGAGTATAGTGTTCGAGAACTTGAATGTTCAGTCAGG
AAACGATGGAACTGGGGTACCCACTGATATGTTGTCGTTGAACTCAACGGTCAGAATT
TTGTATAGAAACCCAGCCACTTTCTTCGGCGTTCATGTCACTTCTACCCCTCTTCACCTT
AGCTATTACCAGCTTGCAATTGCCTCTGGCCAGATGCAGAAGTTCTATCAATCGAGGAA
GAGTCAGCGGAAGTTGGCGGTAGTGGTGCTAGGACACCAGATTCCTCTCTATGGAGGA
GTGTCAGTTCTTGGAAATACTAAAGAGCACTTGGAGAACGTTGCATTGCCACTAAAAC
TGACATTTGTAGTGAGATCAAGGGCTTTCATCTTGGGAAGATTAGTTAAGTCTAAATTCT
ATCGAAGAATCACATGTTCTGTCACTTTGCATGGCAACAAACTTGGAAAACATCTTAAT
TTGACGGATTCATGTGTCTACAAGTGA

>GmLEA2-59
ATGTCCGTCAAAGAGTGCGGCCACCACTCCTCCCAACGCCGCAACCTCCTCCGCCTCA
TCCTCGGCGCCACCGCCGCCTTCGTCCTCCTCATCCTCCTCACCATCTTCCTCATCTGG
GTCATCCTCCGCCCCACAAAGCCCCGCTTCACTCTCCAAGATGCCACCCTCTACGCCTT
CAACCTCTCCACTCCCACACCCAACACCCTCACCCTCACCATGCAGGTCACCCTCTCC
TCCCACAACCCCAACGCGCGCGTCGGCGTCTACTACCACGCGCTCCACGTCTACGCCT
CCTACCGGAGCCAGCAGATCTCCCTCGCCACCGCGCTCCCCGACACCTACCAGGGCCA
CCGCGACTTCGCCGTCTGGTCGCCGTTTCTATTCGGTAACGTCGTCCCCGTCTCGCCGT
TTGTGCTCAGCTCACTGCAGCAGGACCAGAGCGCTGCCGGCGCCGTTTTGGTCAACGT
TAAGGTCAACGGGAGGGTCAAGTGGAAGGTCGGGTCTTGGGTCTCTGGGAGGTACCA
TATCTATGTGAACTGTCCCGCGTATATTAGCTTCGCCGGAGATCGGAGCATTGCCGCCG
GAGGACTCGTGGCTTCGCCGGTGAAGTTTCGCCTCTTGCAGAGTTGCAGTGTTGATGT
GTAG
>GmLEA2-60
ATGGACAACAAAAAGTCAAAATGGAGTTGGAGTTCAGCAATAATCGGAGCAGCATCC
GCAGTGGCAGCATCAGCCCTAATATCCGCGAAACCCAAGGACCCAACATTTCACCTCA
TTTCCATAAACTTCACCTCTTTAAAACTCAATTTCCCTCTCTTAGACGCAGAGGTTCTCC
TCACGGTCCACGTCACCAACCCTAATATCGCCCCCATCCACTACTCCTCCACTTCCATGT
CCATCTTCTACCAAGGTTCTCTTCTCGGCTCGGCTCAGGTTCAAGCCGGCTCCCAGCCG
CCCCGCTCCTGCCAGCTCCTCCGCCTCCCCGCACGCCTCCACGCGCTCGAGCTCGCTC
ACCACGCCACGCGCTTCCTCCACGACGTCGCGCGCCGCGAGATGTTCCTCGACGCCGC
GGTTGATATCGCCGGCACCGCCAGGGTCATGTGGTGGGACCATAACTTCAAGGTCCAC
GTGGACAGCCACGTCACCGTCGATCCCGTGTTTCTCGACGTCATTGATCAGGAAAACA
CCTCCGAGCTTGAAGTATTCACCGCAGCGGCGTTGGGGAGTGAGCAATGA
>GmLEA2-61
ATGGAGGAGGGAAAGAGCAAGTCTACATTGGCGAAGAATCCGAGCCGCAGCCGGAAT
CCGAGCCGGCGTGTGGCCTTGGACGTGCCCTCCCCTCGCCACATGGGCTCCACCCTCA
GTGGCATCCATTCCTCCATCGACCACCTTGACAAGGGACGTTACCACCCATGTTGCTTT
GCATGTTGTGCATGGTCATGCCTTATTGTGTTCATCTTAATCATAGCCATTCTCTTTCTTG
GTATTACCTACTTGGCTTTTCTCAAGTCAGGGATGCCAAAAATTAACGTGAGAGCCTTC
AACATAACAAAGTTTCAAGTTGATGATGGGTCACAAAAGATGAATTCGGTTATAGGCTT
AGGATTAATATTCTCTAACAAGAATGATAAGCTTAAGCTTTTGTATGGGCCTCTTGATGT
TGATGTCACTAGTGAGGATGTGCTATTGGGGAAGAAAAAACAAGGTGGCTTCTCTCAA
AAGCCCTTGAATGTCACCAATTTGGACATGACCATGACATTGGAAAATGCCGATGTTGA
CAAGTATGCCGCTGAAGAGTTGAAATCAGACATCAAAGCCTATGAGATGGTGTTTGATT
TGTATGTGGGTGGACACATTGGTTTTCAGGTTGGGAAATTGCAGATGAATAATGTACCC
TTTCTAGCATCTTGTAATCAAATCAAGAGGGAGGATGTGGATTTTGGAAGAAAGCCTG
AATGTGAAGTTAAGCTGTTTGCTGCCAGGCCTTCAACAAACTGA
>GmLEA2-62
ATGATGGCTGCAGAACATCAAAGAATTCATCCTGTTCATGATGTGGAGGCACAACACA
GACCATTGGTGCCTCAAAACATTGCAAAATCTGATGATAAGGGTATTCCTCATCGCACT
TTTCCTGTGATGCATTCAAAGCCACCTAAGAGAAGAAGAAGCTGTTGCTGCAGGTTCA
TGTGTTGGACACTTAGCATATTACTGATTTTGATAATTGCTATTGCCATCACAATAGGGAT
CCTATACCTTGTTTTTAGGCCAAAGCTCCCCAAATACTCAGTGGACCAACTGAGGATAA

GCCAGTTCAATGTTTCAGACAACAACACCCTCTATGCCACCTTCAATGTTGCAATCACT
GCAAGAAACCCTAACAAGAAGATTGGAATATACTATGAGGGTGGAAGCCACATAAGTG
CTTGGTACATGGAAACACAATTGTGTGAAGGGTCTTTGCCAAAATTCTACCAGGGTCA
CAGGAACACCACAGTGCTTGATTTGCCTCTAACTGGTCAAGCACATGATGCAAATGGC
TTGGTGAATAGAATTCAGGAGCAGCTGCAACAGACCAACAATGTTCCTCTCAATCTTA
AGGTGAATCAGCCTGTGAGGGTTAAGCTTGGTAAGTTGAAGCTCTTCAAAGTCAAGTT
CAGGGTTAGGTGCAAGCTTGAGGTGGATAATATTCTTGGGGCTAGTAATGATATTAGAA
TTTCTAGAAGTAGTTGTAAGTTCAGGCTTAGGCTGTGA
>GmLEA2-63
ATGGGAGGAAGAATGCACACGAAATCAGACTCGGAGGTGACGAGCAACAGCATGGAG
CAGTCGTCGCCGGCGCGTTCTCCTCCGCGGCGGCCACTCTACTACGTCCAGAGTCCTT
CAAACCACGACGTGGAGAAAATGTCCTACGGCTCAAGCCCCATGGGTTCCCCACACCA
CCATTTCCACTACTACCTCTCTTCCCCCATCCATCACTCTCGCGAATCCTCCACCTCTCG
CTTCTCCGCTTCCCTCAAAAACCCTCGCAACTTCTCCTCCTCCTCCTCCTGGAAGAAGC
TCCACCCTCACCCCAACCGCGACGTTGACCCCGACGACCATGACGACGCCGACCACC
ACCTCAAGCAGTCCTCCCGCAACCTCCGCTTGTACTTGTGGTTCTTCCTCCTCTTTCTT
CTCCTTTTGACCCTCTTCTCTTTCATCCTCTGGGCCGCCAGCAAGACCTACAAGCCTCG
AATCATTGTCAAGGTAACTTGTCCAAATGGTATTGTTATTTACTGTAGTCTTTGTGATTT
GATATTTGGTTTTTGTTATGGGGGTGGACAGAGTATAGTGTTGGAGAACTTGTATGTTCA
GTCAGGAAACGATGGAACTGGGGTACCAACTGATATGTTGTCGTTGAACTCAACGGTC
AGAATATTGTACAGAAATCCTGCCACTTTCTTCGGCGTTCATGTCACTTCTACCCCTCTT
CTCATTAGCTATTACCAGCTTGCAATTGCCTCTGGCCAGATGCAGAAGTTCTATCAATCG
AGGAAGAGTCGGCGGAGTTTGGCTGTAGTGGTTTCAGGACACCAGATTCCTCTCTATG
GTGGCGTGTCAGTTTTAGGGAATACTAAAGAGCATTTGGAGAGTGTTGCATTGCCACT
GAACCTGACATTTGTAGTGAGATCAAGGGCCTTCATCTTGGGAAGATTAGTTAAGTCTA
AATTCTTTCGAAGAATCAGATGTTCTGTCACTTTGCATGGCAACAAACTTGGAAAACAT
CTTAATTTAACGGATTCCTGTGTCTACAAGTGA
>GmLEA2-64
ATGTCCGTCAAAGAGTGCGGCCACCACTCATCCCAACGCCGCAACCTCCTCCGCCTCA
TCCTCGGCGCCATCGCGGCCTTCGTCGTCCTCATCCTCCTCGTCATCTTCCTCATCTGGG
TCATCCTCCGCCCCACAAAGCCCCACTTCACCCTCCAAGACGCCACCCTCTACGCCTTC
AACCTCTCCACCCCCACACCCAACACCCTCACCCTCACCATGCAGGTCACCCTCTCCT
CCCACAACCCCAACGCGCGCATCGGCGTCTACTACCACGCGCTCCGCGTCTACGCCTC
CTACCGGAGCCAGCAGATCTCCCTCGCCACCGCGCTCCCCGACACCTACCAGGGCCAC
CGCGACTTCGCCGTCTGGTCGCCGTTTCTCTTCGGCAATGTCGTCCCCGTCTCGCCGTT
TGTGCTGACCTCGCTGCAGCAGGACCAGAGCGCCGCCGGCGCCGTAGTGGTCAACGT
TAAGGTCAACGGGAGGGTCAAGTGGAAGGTCGGGTCTTGGGTCTCTGGGAGGTACCA
TATCTACGTGAACTGTCCCGCGTATATTAGCTTCGCCGGAGATCGGAGCAATGTCGCCG
GAGTCGTGGCTTCTCCGGTGAAGTTTCGCCTCTTGCAGAGTTGCAGTGTTGATGTGTA
G
>GmLEA2-65
ATGAGCACCAAGGAGTGCTGCCACCACGACGAGGAGCGGCGTCAGCTCCCCCGCCGC
GTATTCGCCGCGATCCTCGGCTTCATCCTCCTCATCCTCCTGGTGATCTTCCTCATCTGG
ATCATCCTCCGCCCCACCAAACCCCGCTTCATCCTCCAAGACGCCACCGTCTACGCCTT

CAACCTCTCCTCCACCGGCGACACCCCTTCCCCATTACCCCAACCCCCAACACCCTC
ACCCTCACCATGCAGGTCACCCTCGCCGCCTTCAACCCCAACCACCGCATCGGAGTTT
ACTACACCAAACTCGACGCCTACGCCGCCTACCGCGGCCAGCAGGTCTCCGTCGCCAC
CTCCCTCCCCGCCACCTACCAGGGCCACCGCGACACCTCCGTCTGGTCCCCCTACCTCT
ACGCCACCGCCGTCCCCGTCTCCCCCTTCACGCTCCAGATCCTCCAGCAGGACAAAAC
CTCCGGAGGAATTCTGGTCAACGTAAAAGTCAACGGAAGAGTCAAGTGGAAGGTCGG
AACTTGGGTTTCCGGAATTTACCATATTAATGTCAACTGTCCGGCGTACCTCAGGCTCG
CCGGCGACCGGGACGACGCCATCGGATTTGCCGGTCCGGCCGTGAAGTTTCAGCTCTT
CCAAACATGCATTGTTGATGTCTAG
>GmLEA2-66
ATGGCTAATAGAGGCCTCAAAATTTGCTTGGCTGTGTCTCTACTCTTCTTGGTCATCGTT
ACAATTATGATTGTGACTTTATTTATGACCATCTTTAAGCCCAAAAATCCTGAAATCACT
GTCCACCCTGTTGGTCTAGAAGACTTTCAATTTTCCCTTTCACCCAATTTGACCATAAAT
GTGACTCTAGGCATGATAATTACTATAAGGAATCCAAACTATGGAAGCTTCGAGTACAA
AAACTCCACTGGTTATGTGAATTTTCATGATACCGTGGTAGCCGAAGTTCCAATAGAAG
CAGAGTTAGTTCCAGCACGTGGCCAGATTAATGTGAACACTTCAGCAGATTTTATGGTA
GAAAAGTTAATCAATGATCCTAATTTTTTGTCAGATGTTCTAGGTGGAACTTTGAATTTC
ACATCAACAACTGCACTTCCTGGGAAAGCCCGTATGTTCAACATTATCAAATTGAAGGC
CACGTCTTATAGCTCATGTGACATCTCTGTTAATATAAGCTCTAGGAAAGTTGATACCAA
TTGCAATTACAAAATCAAGCTTTAA
>GmLEA2-67
ATGACATCTGAAGCTTATGTACCTAAAAGATATGAGTCGCTAGAGAACCCTTTTGCTCAT
AAACCGCGTAAGCGTTCCCACCAAGGCTCTGGTTTATGCGGGTGCCTTAGAAAGGAAA
ATAAGGTATACTCTGATGTTGTCATTGTCGTGAAAGCGGATAATCAGAATGAAGACATT
GGATTGGACTATTTAGATAATGAAGTTGGGATAATGTACTTAGGATCTCAACTTAGCTCG
GGGCAGATCCCTCCTTTCTTGCAGCCAGGGAAGAACACAACAAAGGTGAATGTGGAA
TTGAAAGGGGAAAATGAATTTGGTGAGAAAGGGAAAATTCCTTTGTTGATAACAGTGA
AGCTTCCCATAAGGCTTGTGATTAAAGATTTGATTCATCTCAGGAAAGTTGTGGTTAAT
GTAAATTGTTCCGTAGTTATTGACAAGTTGGAAGCCAACAAAAGTCCTAAGATTTTGGA
CAAAGTTTTCACCTATGGCATAGATTTCTGA
>GmLEA2-68
ATGCGACCAACACCCACCCCTGATAACCTCAATTGCATTCCCAGAATAAAGGTAGGATC
TGGTAAAGGGAGAAAAGTGTGCCTGACGGTGACAGGTGTTGTGATTGCAATTGTATTG
CTAATTGTGATACTAGCGTTGACAGTGTTCAAAGCCAAGCATCCTGTTACCATAGTGGA
CTCAACGAAGCTAGAGGACTTTCACGTGAGCTTGGATCCAGTAAAACTAAGGGTAGAT
TTGAATGTGACCTTGGGAGTGGATGTCTCAGTGAAGAACCCGAACAAGGTGGGATTCC
AGTATTCAGACAGCGCTGCCCACCTCAATTACAGAGGGCAGCTGATAGGTGAAGTCCC
GATCTCTGCCGGAGAGATTTCATCCGGTGAGACCAAAGGATTCAATCTCACCCACACC
ATTATGGCCGACCGTTTGCTCTCCAATTCTCAGCTTTTATCTGATGTCACATCTGGTACA
TTGCCCCTAAGCACTTTCGTGAGGATGTCTGGGAAAGTCAGCATCTTAGGCTTTATCAA
AGTCCATGTGGTTTCCTCCACTTCTTGTGATGTTGCAATTAATCTTTCTAATGGAACTGT
TGGGAACCAAGAGTGCCAATACAAGACAAAACTTTGA
>GmLEA2-69
ATGGCTCATCCTCCCACACAATCGCAGTCTAGAGCAGCCAATAAGCCAAAACGCTCAA

ACCTTCTTCGTTGCATTGCCATTTTCATACTAGCCTTGATCATCCTTGTGGGCATTGCTG
TGATCATAATTTGGCTAGTTTTGAAGCCCAAAAGGTTGGAATACACTGTGGAGAATGCT
GCAATCCACAACTTCAACTTAACCGATGCAAATCACCTCTATGCCAACTTTGACTTCAC
CATAAGATCCTACAATCCCAACTCTAGAGTCTCCATATACTATGACACTGTGGAGGTGTC
AGTGCGTTATGAGGACCAAACTCTAGCAACCAATGCGGTTCAGCCATTTTTCCAGTCA
CATAAGAATGTGACCAGGCTTCATGTGGGCCTCACGGCCCAAACCGTGGCCCTGTATG
ATTCTGTGCCTAAGGACCTTAGGCTCGAGAGATCCTCTGGGGATATTGAGTTGGATGTG
TGGATGAGGGCAAGGATAAGGTTCAAGGTTGGAGTGTGGAAGTCAAAGCATCGTGTTT
TGAAGATTTTTTGTTCTCCTGTCTTGGTGCACTTTTCTAAAGGAAAGAGTTTCGAAAGG
GCCCCTTGTGATGTAGAACTCTAA
>GmLEA2-70
ATGACATCTGAAGCATATGTGCCTAAAAGATATGAGTCTCTAGAGAACCCTTTTGCTCAT
AAACCGCCGCGTAAGCGTTCTCGTCGTGGTTCTGGTTTTTGCGGGTGCCTGAGGTGTT
GTTGCTGCTGCTTTAGTTTCTGCCGTTGCTGCATTTGCACCATTTTTATCATAATTATACT
CTTGGTGGGAATTGGTTTGGCTTTATTTTACTTTATTAAGCCTAAGGTTCCTTCCTATGA
CATTGAACACATTAATGTCAAGTCATTTGATATCAGAAAGGAAAGTAAGGTATACACTG
ATGTTGTCATTGTCGTGAAAGCGGATAATCCTAATGAAGAAATCGGATTGGACTATTTG
GAGAATGAAGTTGGGATAATGTACGTAGGATCTCAACTTAGCTCGGGGCAGATCCCTCC
TTTCTTGCAGCCAGGGAAAAACACAACAACGGTCACCGTGGAATTGAAGGGGGAAAA
TGAATTTAGTGTTGAAATGCAAAATCACTTTATGGAAGACCAGGAAAAAGGGAATATT
CCTTTGTTGATAACAGTGAAGCTTCCCATAAGGATCGTGATTAATGATTTGATTCATCTC
AGAAAAGTTGTGGTTAATTTAAATTGTTCCGTGGTTATTGACAAGTTGGAAGTCAACAA
AAGTCCTAAGATTTTGGACAAAGTTTTCAGCTATGGCATAGAGTTCTGA
>GmLEA2-71
ATGGCTAATAAAGGCCTCAAATTTTGTTTGATCGTGTCTCTACTCTTCTTGATCATTGTT
ACCATTGTGATTGTGACCTTGTTTTTCACCGTCTTTAAACCCAAGGATCCTAACATCACT
GTCCACCCTATTGGCCTCGAACACTTTGACTTTTCCCTTTTACCCAATATCACCGCGAAT
GTGAGTTTAGGCATGGTGATCACTATAGAGAATCCAAATTATGGAAGCTTCGAGTTCAC
AAACTCGATTGGTTACATCAATTTTCATGATACTGTTGTAGGCGAAGTTCCAATTGGGG
CAGAGTTGGTTCCAGCGCATGGCCAGATTAATGTGAACACTTGGGCAAATTTTATGGTA
GCGAAGTTAATCAGTGTTCCCAAATTTTGGTCAGATGTTCTAAGTGGAACTTTGAATTT
CACATCAACATCTTCACTGCCTGGGATAGCTCGCATGTTCAAGATTTTCAAATTGAAGG
CCACGGCCTATAGCTCATGTAACATCTCTCTTAGAATAGTCCCTAGGAATGTTGATACCA
AATGCATATCCAAAATCAAGCTTTGA
>GmLEA2-72
ATGGCTTCTAGAGGCCTAAAAATTTGCTTGGCTGTGTCTCTACTCTTGTTGATCATTTTT
ACAATTGCAATTGTGACCTTATTTATGACCATCTTTAAGCCCAAAAATCCTGAAATCACT
GTCCACCCTGTTGGTCTAGAAGACTTTCAATCTTCCTTTTCACCCAATTTGACCCTAAAT
GTGACTCTAGGCATGATAATTACTATAAGAAATCCAAACTATGGAAGCTTCCAGTACATA
AACTCCACTAGTTATGTGAAATTTCATGATACCGTGGTAGCTGAAGTTCCAATTGAAGC
AGAGTTAGTCCCAGCACGTAGCCAAATTAATGTGAACACTTCAGCAGATTTTATGGTAG
CAAAGTTAATCAATGATCCTAATTTTTTGTCAGATGTTCTAGGTGGAACTTTGAATTTCA
CATCAACAATTGCACTTCCTGGGAAAGCCCGTATGCTCAATATTATCAAATTGAAGGCC
ACGTCCTATAGCACATGTGACATCTCTGTTAATATAAGTTCTAGGAATGTTGATACCAAT

TGCAAGTCCAAAATCAAGCTTTAA
>GmLEA2-73
ATGATAAATCCCACCAAAGCTATGCCCGTTTCACACTCTTTCCTTTCTCTGGCTGATTCC
ACAGACACACACCGCTTTACTATTATGCAAGACCCTTCTCGCCCCGCCCCTCCTCCCTT
CTACCACCAAAACGGCGGCCAGCCTCCTCCCCCCGCCGCCTCCGCCGCCATCGCCACC
GGCTACCCCTACGCTGCCCAACAACAATACTACAACACCACTTACCCTCCTCCTCGCTC
CTACGCCTCCCGCTCCTTCTTCCGCGCTTTCTTCGCCACCATGATCTGCCTCGCCGTCGT
CTTCGGCGTCGTCCTCATCATCACCTGGCTCGTCCTCCGCCCCTCCCTCCCCCACTTCA
CCCTCCACTCCCTCTCCGTTTCCAACCTCTCCAGCACCTCCCAATCCCTCTCCGCCACG
TGGCACCTCTCCTTCCTCGTCCGCAACGGCAACAAGAAGATGACCGTCTCCTACAACG
CCCTCCGCTCTTCCATCTTCTACCGCCAAAACTACATCTCCGAGTCTCAGCTCGCTCCC
TTCAGGCAGGACACCAGGTCCCAGACCACACTCAATGCCACCCTCACCGCCGCCGGC
ACCTACCTCGAGCCCAAACTGATCGACAATCTCAACGCCGAGAGGAACGCCAGCTCG
GTCCTCTTCGACGTTCAGGTCGTCGCCGCCACCTCTTTCCGCTCCGGCTCGTGGAGGTT
CAGGACGAGGGTTCTCAAGGTTCTGTGCCGCAAAGTACCCGTCGGCGTCTCGTCCAAG
TCCAGCTCCGGTGATTTGGTCGGCGGAGATCGGGAATGTCAGGTGTGGACGTGA
>GmLEA2-74
ATGAGCACCAAGGACTGCGGCAACCACGACGAGAAGAACCGTCAGTTCCTCCGCTGC
CTATTCGCGGCGATCCTCGGCTCCATCCTCCTCCTCATCTTCCTCATCTGGATCATCCTC
CGCCCCACCAAACCCCTCTTCATCCTCCAAGACGCCACCGTCTACGCCTTCAACCTCTC
CTCCTCCGGCCCCACCCCTTCCCCCATCAATCCAACCCCCAACACCCTCACCCTCACCT
TGCAAGTCACCCTCGCTTCCTTCAACCCCAACCACCGCATCGGAGTCATCTACACCAA
ACTTGACACCTACTCCGCCTACCGCGGCCAGCAGCTCTCCATCGCCACCTCCCTCCCCG
CCACCTACCAGGGCCACCGAGAAACCGCCGTCTGGTCCCCCTACCTCTACGCCTCCGC
CGTCCCCGTCTCCTCTTTCACTCTCCAGATCCTCCAGCAGGACAGAACCTCCGGAGGA
ATTCTGGTTAACGTCAAAGTCAGCGGAAGAGTGAAGTGGAAGGTTGGAACTTGGGTT
TCCGGAAATTACCATATTAATGTCAACTGTCCGGCATACCTCAGGGTCGCCAGCGATCG
GGACGACGCCGTTGGATTCGCCGGTCCCGCCATCAAGTTTCAGCTTTCACAAAGTTGC
ATTGTTGATGTCTAG
>GmLEA2-75
ATGACAGACAGGGTGTACCCATCGGCCAAGCCCGCCGCGGTTAACGGCGCCGCCGCC
AACCCATCTTTCCCGGCGACTAAGGCTCAACTTTACGGCGCCACACGCCCCACCTACC
GCCCTCAGCCCCACCACCGGCGCCGGAGCAAGCGCCGGTGCTGCTGCACCTTCTTCTT
CTGGCTGATCCTCACCGTTCTGATCCTCCTCCTCCTGATCGGCGTCGGCGGCACGGTGT
TCTACCTCCTCTACCGCCCCCACCACCCCACCTTCACCGTGACGTCGCTGAAACTCTCT
TACCTGAACCTCACCTCCTCCTCCAACACCCTTAACTCCCGCTTCGACATCACCGTCTC
CGCCACGAACCCCAACAAGAAGATCCTCTTCGCCTACGACCCCACCTCCATCACCATC
CTCTCCGCCGACATCGACCTCGGCGACGGCACCGTCCCCGGCTTCCAGCACCCGAAGA
AGAACACCACGCTGATCAAAGGCTCCATCCTCAGCTCCGGCCAGGCGCTCCAAAGCG
ACGAAGCCTCACGCTTGAAGTCCAGCATGAAGAGCAAGAACGGGTTGCCACTCAAAG
TGAATTTGGAAACCAAAGTGAAGGCCAAGATGGGAAATTTGAAAACCCCAAAAGTCG
GAATCAGAGTCTCCTGCGACGGAATCAGAGTCAGTCTTCCCTCCGGTAAGAAACCGGC
GACGGCGTCCACTTCTAACGCCAAATGTGACGTCGATGTTCGGTTCAAGATCTGGAAG
TGGACCGTTTGA

>GmLEA2-76
ATGACGTTGTCAGCAAAATCTGAATCGGATATCACAAGCTTAGCTCCATCTTCACCTTC
AAGATCACCAAAGCGGCCTGTGTACTATGTGCAGAGTCCTTCAAGGGATTCTCATGATG
GGGACAAGTCATCTTCAATGCAGGCAACTCCAATATCAAACAGTCCTATGGAGTCTCCC
TCACACCCTTCATTCGGGCGTCACTCAAGGAACTCTTCCGCGAGCCGCTTTTCGGGGA
TTTTCAGGTCATCTTCGGGAAGGAAAGGGAGTAGGAAGAGGAATGATAAGGGGTGGC
CTGAGTGTGATGTGATTCTGGAAGAAGGGTCTTATCATGAGTTTCAGGACAAGGGTTT
CATGAAGGCGCTTCCAGGCCTTGATTACCTTTGTGGTTGTCTTCGCTGTGTTTTGTTTGA
TCATTTGGGGCCTTACAAGGCACAAATTGCTGTTAAGAGTTTGACGGTGCATAATTTCT
ATGTTGGAGAGGGTTCAGACTTCACGAGTGTCCCCACAAAAATGCTGACAGTTAATGG
CACTTTACGCATGAGCATCTACAACCCTGCTACATTGTTTGGGATCCATGTGCACTCCA
CACCCATCAATCTTGTTTTCTCAGACATCACTGTTGCTACTGGCGAGCTGAAGAAACAC
TATCAGCCAAGAAAAAGTCACCGCATTATATCAGTGAACCTAGAGGGTACTAAGGTTCC
CTTATATGGGGCTGGATCCACCATAACTGTATCCCAAACTGGCGTTGAAGTTGGACTCA
CATTGAACTTTGAAATCCGTTCGCATGGAAATGTGGTGGGTAAACTGGTCAAGACAAG
GCATCGTAAGGAAATCACTTGCCCCTTGGTCCTCAACTCTTCTCGATCAAAGCCCTATC
AAATTCAAAAGGAATTCATGCACCTATGA
>GmLEA2-77
ATGCGATTCTCAAAAGCTTCCTTCTACACTCTTCTTCTCCGCAAACCTCATAACCATCAC
CATCAAAACTACGTCGTTTTATCGCGGCGTCGCGTAGTGACCTTGGCAGTGGGTGTGTT
GATCTTGCTGAGTGCTCTCTACATTTTCTGGCCATCGGGTCCTGATCTGAAGATCGTGG
GGCTGAAACTGAGGCGCATAAAGGTGCACCCTGTGCCGCCAATCACCATAGACATCTC
CATGCTTCTCACGCTGAGGGTGCACAACGTTGACGTCTACTTCATGGACTTCGGAGCG
GTTAACGTCGCCGTCGCCTACAGGGGGAAGATGCTGGGCCACGTGACGTCCAGGCGC
ATGCACGTGAGGGCCAGGGGGTCTTCCTACGTGGATGCTGACGTGGAATTTGCTGGCA
TCAGCGTGCTACCGGAGCTTGTGTTGCTGCTGGAGGACGTGGCCAGAGGTATCGTACC
CTTTGATACCATTAGCCATGCTAAGGGCCAATTGGGCCTTCTCTTCTTTCACTTCCCCAT
CAAGGCAAAACTATCGTGTGAGGTTGTGGTCAGTATAATAAACCAGACCATTGTTCGTC
AACATTGTCTCCACGAGTGA
>GmLEA2-78
ATGCTAGCCTCTGTCGAAGAAGAACTTGGCACACCAAAACAACCAAGGAATCAACATT
CTCAGCAATCAGAAGCACCAAATTCCCTTGCCAACACTAGAGCAATTTTCGGGCAACC
TCGACTTCAAAGGACAAAACCAATTATATGGTGTGCTGCAATACTGTGCTTCATATTCA
GCCTAGTGCTTATCTTCTTTGGAATTGCAACACTGATTCTCTACCTTTCCATGAAACCAC
GAAACCCCACGTTTGACATTCCCAATGCAAGCCTCAATGTGGTGTACTTTGACTCACCA
CAGTACCTCAATGGTGAATTCACTCTCCTTGCAAATTTTTCCAATCCTAACAGGAGAAT
TGGGCTGAGGTTTGAGTCCTTGAACATTGAGCTTTTCTACTCAGACAGACTCGTGTCTT
CACAAACAATCAAGCCTTTCACTCAAAGGCCAAGGGAAACCAGGCTGCAATCAGTGA
ACTTGATATCAAGCTTGGTGTTTTTACCGCAAGATGTTGGTGTAAAACTTCAAAGGCAA
GTGGAGAATAACAGGGTCAATTACAATGCAAGGGGAACATTTAAGGTGAGGTTCAATA
TTGGCCTTGTCCATTTATCTTACTCTCTGTATAGCACATGCCAGATAGAGATGACTAGTC
CTCCAGCTGGTATTCTAGTAGCAAGACAATGCATAACAAACCGATGA
>GmLEA2-79
ATGTCGACATCTGATAAGCCAGAAGTGGTGGAAAGGGGTAGCAAGGATGAAAAACAC

AAGGAAGATGATCAAGAGGAGGGAAGGGTGGATTCATTGAGAAGGTGAAGGATTTC
ATTCATGACATTGGTGAGAAGATTGAGGAAGCTATTGGGTTTGGGAAGCCAACTGCTG
ACGTTACTGCGATTCACATTCCATCGATTAATCTTCACAAGGCAGATCTTGTTGTTGATG
TGCTCATCAAGAACCCGAATCCGGTGCCAATCCCTCTGATTGACATAGATTACTTGGTT
GACAGTGATGAAAGGAAGCTAGTTTCTGGATTGATACCAGATGCGGGTACCATCGGTG
CACATGGAGAGCAGACTGTCAAAATTCCTGTCACTTTGATTTATGATGACATCAAGCAA
ACATATGCTGATATTAAACCTGGAAGCATCATTCCTTATAGGGTGAAGGTTAGTCTCATT
TTTGATGTTCCCATCTTGGGAAGGCTAACTCTACCTTTGGAGAAAACTGGAGAAATCCC
CATACCATACAAGCCTGATATTGATCTTGAGAAGATTCATTTTGAAAGGTTCTCTTTTGA
AGAGACAATTGCAACGCTTCATTTGAAGTTGGAAAACAAGAATGATTTCGACCTTGGC
CTCAATGCGCTCGATTATGAGGTCTGGCTTGGTGATGTTAGCATTGGTGGTGCAGAACT
CACCAAGTCTGCTAAAATTGAGAAAAGTGGAATTAGTTATATTGATATTCCAATTACCTT
CAGGCCCAAGGATTTTGGCTCTGCACTCTGGGACATGATTAGAGGAAGGGGAACAGGT
TACACCATGAAAGGACATATTGATGTTGACACTCCCTTTGGAGCAATGAAGTTGCCCAT
CAGCAAAGAAGGTGGTACTACCCGTCTTAAGAAAAAGAAGGAAGATCGTGATTATGAT
GACGATGACGATGATGAGGATTGA
>GmLEA2-80
ATGGCTGACCGAGTTCACCCCAGCCACTCGCCTTCCGTTTCCGCGGATTCACAACCGC
CATCGCCTCAAGACTCCTCCGTCGTTCCGAAACCGCCGTCGCCACCGTCACCGGAGAA
GCCAGTGCCTCCGCCTGGAACCTACGTCATCAAGATCCCCAAGGACCAAGTCTACCGC
GTCCCTCCGCCGGAGAACGCTCGCCGCTACGACCAATACGCTCGCCGTAAACACCGCC
GGAGCCGATGCTGCTGCTGCTTCTGCTGGCTCATCGGAATCCTCTTCATTCTCGTCGTG
CTCCTCGCCATCGCCGCCGGCGTCCTCTACCTCGTTTTCCGTCCCGAGGCGCCGAAATA
CTCCATCGAAAACATCACCGTCAGAGGAATTAACCTCACCTCGCCGTCGTCCGTGGCG
GCGATCTCGCCGGAGTTCAACGTCACCGTCAAGGCCGATAACCCTAACGACAAGATCG
GGATCCGTTACTTGAAGGATAGCTCCGCCGAGGTGTTTTATAAGGACGCGAGGCTGTGT
AACGGCGCTCTGCCGGCGTTTTACCAGCCGTCAAACAACGTGACGGTGTTTGGGACG
GCGTTAAGGGGTGACGGAATCGAGCTGAGAAGCGAGGACCGAAGAGCGTTGCTGGA
GGCGCAGACCAAACGGAGAGTGCCGTTGACCGTTAGGATTAGGGCACCGGTGAAAAT
AAAAGTGGGGTCCATTAGGACGTGGAAGATTACCGTTAAGGTGAACTGTGATGTGACG
GTGAATGAGTTAACGGCGCAGGCCAAGATTGTTTCTAAACGTTGTAGTTATGACGTGGA
TCTTTGGTGA
>GmLEA2-81
ATGGTAGCATTATGTGTAGCGATTGCACTTGTATTGTTAAGGGTGATCTTAGCATTGACA
GTGTTCAAACCAAGGCACCCCATTACCAATGTGGATTCAATAAGGCTTCAGAACATGA
GCTTGGGCATGGACATGTTTAGCATGAGTGTAAATGTGAACTTCACATTGGAAGTGGAT
GTTTTAGTCAACAACCCTAATAAGCTTGGATTCAATTACTACAATAGCTCTGCCCAACTC
AATTATAGAACACAATTGATTGGGGAAGCTCCTATCCCCAATGGAGACATATTAGTTGA
GGAGATTAAGGGACTTAACTTGACACTCACTGTTATGGCTGATCGTTTGGTCTCCAATT
CTAAGGTCACCAAGGATGTTGCATTGGGTTCATTGCCCCTCAACACCTTAGTGAGAATT
TTTTGCCAAGTCAACGTCTTAGGGTTTATGAAATTCTATGTGGCTTCCACCTCATACCAA
AAATTCCCCATTTCCTCGAGTTCGAGAATTTGTACAAAGGAGCAGAGTTGTCCCCATTC
ACTTCAATATAGCAAAAGAATCACAAAATAG
>GmLEA2-82

ATGGCAGACCCTCAGAAAATTCACCCTGTGCACCATGATGTTGAGGCACAAAACCACC
CTTCAGCACCATTGGTGCCAAGGAGCATGTCAAAATCTGATGCTGGTGATCCACAAAG
AGTAGTAGTAGTACAACAACAACAACAACAGCAGCAGCAACACATTCCTGTGAAGCA
CACCAAGCCACCAACAAAGAAAAGAAGAAGCTGCTGCTGCAGGTTCTTCTGTTGGCT
AATTAGCATATTGCTGATTCTGATTGTGGCAATTGGCATCACTATTGGAATACTATACCTT
GTCTTCAGACCAAAGCTTCCAAAGTACTCAGTGGATGAACTTAAAGTCACAAACTTTG
ATCTTGCAGACAACAACAGTTTATCAGTGACATTCAACTTGACAATCACTGCCAGAAA
CCCCAACAAGAAGATTGGAATAGACTATAGGGGTGGCAGCCACATAAGTGCTTGGTAC
ATGGACACAAAATTATGTGAAGGGTCTTTGCCAAAATTCTATCAAGGTCACCGCAACA
CAACGATTCTTAGTATCCCTCTTACGGGGAAAACGCAGGATGCTACCGGCTTGCAAAAT
ACCCTTCAGAATCAGCTGCAAGAGACTGGGAATGTGCCTCTTAATTTGAGGGTGAAGC
AGCCTGTGAGGATCAAGCTTGGGAAGTTGAAGCTGTTCAAAATCAAGTTCAGGGTTAG
GTGCAGGATTGTGGTGGATAGCCTTAGTGCTAATAGTTCTATTAGAATTCAGAGTAGTA
GTTGTAAATTCAGGTTTAGGCTGTGA
>GmLEA2-83
ATGATGTTGTCAGCAAAATCTGAATCGGATATCACAAGCTTAGCTCCATCTTCACCTTC
AAGGTCACCAAAGCGCCCTGTGTACTATGTGCAAAGTCCTTCAAGGGATTCTCATGAT
GGGGACAAGTCATCTTCAATGCAGGCAACTCCAATATCAAACAGTCCTATGGAGTCTC
CCTCACATCCTTCGTTTGGGCGTCACTCGAGGAACTCTTCTGCGAGCCGCTTTTCGGG
GATTTTCCGGTCATCTTCTGGAAGGAAAGGGAGTAGGAAGAGGAATGATAAGGGGTG
GCCTGAGTGTGATGTAATTCTGGAAGAAGGGTCTTATCATGAGTTTCAGGACAAGGGT
TTCACAAGGCGCTTCCAGGCTTTGATTGCTGTCCTTACCTTTGTGGTTGTCTTCACCGT
GTTTTGCTTGATCATTTGGGGTGCTAGCAGGCCTTACAAGGCAGAAATTGCTGTTAAGA
GTTTGACGGTGCATAATTTCTATGTTGGAGAGGGTTCAGACTTCACGGGTGTCCTCACA
AAAATGCTGACAGTTAATGGCACTTTACGCATGAGCATCTACAACCCTGCTACATTTTT
TGGCATCCATGTGCACTCCACACCCATCAATCTAGTTTTCTCAGAAATCACTGTTGCTA
CTGGCGAGCTGAAGAAACACTATCAGCCAAGAAAAAGTCACCGCATTGTATCAGTGA
ACCTAGAGGGTACTAAGGTTCCCTTATATGGGGCTGGATCCACCATAACTGTATCCCAA
ACTGGTGTTGAAGTTCCACTCACATTGAACTTTGAAATCCGTTCGCGTGGAAATGTAGT
GGGTAAACTGGTCAAGACAAGGCATCGTAAGGAAATCACTTGCCCCTTGGTCCTCAAC
TCTTCTCGATCAAAGCCTATCAAATTCAAAAAGAATTCATGCACCTATGATTGA
>GmLEA2-84
ATGACAGACAGGGTGTACCCATCCGCCAAGCCCGCCGTGGTTAACGGCGGCGCCGCC
AACCCATCTTTCCCGGCGACGAAGGCTCAACTTTACGGCGCCACCCGCCCCACCTACC
GCCCTCAACCCCACCACCGGCGCCGGAGCCGGCGACGGTGCTGCTGCACCTTCTTCTT
CTGGCTGATCCTCACCGTGCTGATCCTCCTCCTCCTGATCGGCGTCGCCGGCACGGTGT
TCTACCTCCTCTACCGCCCGCACCACCCCACCTTCACCGTCACGTCGCTGAAACTCTCC
TACCTGAACCTCACCTCCTCCTCCAACACCCTGAACTCCCGCTTTGACATCACCGTCTC
CGCCACGAACCCCAACAAGAAGATCCTCTTCGCCTACGACCCCACCTCCATCACCATC
CTCTCCGGCGACATCGACGTCGGCGACGGAACCGTCCCCGGCTTCCAGCACCCGAAG
AAGAACACCACGCTCATCAAAGCCTCCATCCTCAGCTCCGGCCACGCGCTCCAAAGCG
ACGAAGCTTCACGTTTGAAGTCCAGCATGAAGAGCAAGAACGGGTTGCCACTCAAAG
TGAATTTGGAAACCAAAGTGAAGGCCAAGATGGGAAATTTGAAAACCCCAAAAGTGG
GAATCAGAGTCTCCTGCGACGGAATCAGAGTCACTCTTCCCTCCGGCAAGAAACCGGC

AACGGCGTCCACTTCCAACGCTAAGTGCGACGTCGATGTTCGGTTCAAGATCTGGAAG
TGGACCATTTGA
>GmLEA2-85
ATGACCACCCCAAGAGGAAACCACCACCCGCAGCCTAACGGTGAGCGAAGAAGACAA
TGGCCACCGGCACATCCAGCAGCGGCAGCGGCAGCGGCTCCGGAAGCGGCAGAGAC
AGCGACAGCAACAGCAGCAGGCGGACATTCTTACAATGGGTACCGACAGTACCACCC
AAGAACGCCGGGTCGTTCGTCGTCGTCGTCGGCATCGTTCAAAGGGTGCTGTTGCTGC
CTCTTCCTTCTATTCTCGTTCCTGGCGCTGCTGGTGCTAGCGGTGGTGCTGGTAATAATC
CTAGCGGTGAAGCCGAAGAAGCCACAGTTCGATCTGGAGCAAGTGGGGGTGCAGTAC
ATGGGCATCACACCCAATCCCCCCTCCACCGCTTCCCTCTCCCTCACTATTCGCCTCCTC
TTCGCCGCCACCAACCCCAACAAGGTCGGGATCAGGTACGGCCAGTCCAGCTTCACCG
TCATGTACCGCGGCATCCCATTGGGAAAAGCCACCGTCCCCGGCTTCTTCCAACAGCC
TCACAGCACCCGACAGGTCATCGCCACCATCGCCGTCGATCGCGTCAATTTGCTTCAG
GCCGACGCCGCCGATTTGATCCGAGACGCCTCCCTCAGCGACCGAGTCGACCTCAGAG
TCTTGGGCGACGTTGCTGCCAAGATCCGCGTCATCAACTTCGATTCTCCAGGTGTTCAG
GTCTCGGTGGATTGTGCTATTGTGATCAGTCCAAGAAAGCAATCTCTGACTTACAAGCA
GTGTGGGTTCGATGGATTGACTGTTTGA
>GmLEA2-86
ATGGCTCATCCTCCCTCACAATCGAACTCTACAGCAGCCAATAAGCCAAAACGTTCCA
ACCTTCTCCATTACATTGCCATGTTTATAGTAGCCTTGATCATCCTTGTAGGCATAGCTGT
GATAATAATTTGGCTAGTTTTGAAGCCAAAAAGGTTGGAGTACAGTGTGGAGAATGCT
GCAATCCACAACTTCAACTTAACGGATGCAAATCACCTCTATGCCAACTTTGATTTCAC
CATAAGATCCTACAATCCCAACTCTAGAATCTCCATATACTATGACACTGTGGAGGTGTC
AGTGCGTTATGAGGACCAAACTCTAGCCACCAATGCTGTTCAGCCATTCTTCCAGTCAC
ATAAGAATGTGACCAGGCTGCACGTGGCCCTCACGGCGCAATCCGTGGCCCTGTATGA
GTCTGTGCCTAAGGACCTTAGGCTTGAGAGATCCTCTGGGGATATTGAGTTGGATGTGT
GGGTGAGGGCAAGGATAAGGTTCAAGGTTGGTGCGTGGAAGTCAAGGCATCGTGTTT
TGAGGATTTTTTGTTCTCCTGTGTTGGTGCACTTTTCTAAAGGAAAGAGTTTCGAAAGG
GCACCTTGTGAAGTAGAACTCTAA
>GmLEA2-87
ATGCTAGAGACGGAGCAAGCAAGACCATTAGCCCCATCAATAGAGCGCCAAAGCAGC
GATGAGGATAACACCACTCCACACCCTCAAACACAAGGCCACAAAAAACTCATCAAA
CGTTGTGCTTGCCCATTAATCTCTTTACTTCTCATAGCAATAGTGATCATAGTTTTAATCT
TCACAGTATTTCGTGTCAAGGACCCCGTGATCACAATGAACAGCATCAAGATCACAAA
GCTCCAACTTGTCAACACCATGTCACAACAACCCGGGGCCAACATGTCCTTAGTCGCT
GACGTGTCGGTGAAGAACCCTAACGTTGCATCCTTTAGGTATAGCAACACCACCACGA
GTTTGTACTACCATGGTGTCATTGTGGGGGAGGCTAGAGGACCACCTGGGAGGGCCAA
GGCCAGAAGAACATTGAGGATGAATGTCACAATTGATGTCATCACCGCTCGCGTCATTT
CTAGCCCGGATTTTGTGACGGATTTAGGTTCCGGGTTGTTGACTATGAGCAGCTTCTCT
AGAGTTCCTGGGCAGGTCAAGATCTTGAACTTGATTAAGAGACATGTTGTTGTGAAAA
TGAATTGCACCACCACTTTTAATATTTCCACACAGGCGATTAAGGAGCAAAGTTGTAAG
CGGAAGGTTAAACTTTAG
>GmLEA2-88
ATGTCGCAGTTGCTGGATAAAGCCAAGAACTTCGTGTCGGAGAAGGTAAATGATATGG

CCAAGCCGGAGGCGAGTGTCACTGACGTGGACTTCAAGCGCGTGAGCAAAGATAATG
TCGAGTACTTGGCCAAGGTCTCTGTTCGTAACCCTTATTCAACTTCCATACCCATTTGTG
AGATCAACTACTCTTTCAAAAGCGCATCCAGGGAGATAGCATCAGGGAAAATTCCAGA
CCCAGGATCGTTGAAGGCAAAGGACACAACAATGGTGGATGTGCCAGTTAAGGTTCCA
TACAGCATATTGATGAGCTTGGCAAAGGACATTGGTGCTGACTGGGACATAGACTATCA
ATTGGATCTTGGTCTGGTTATTGATGTTCCTGTCATTGGCATCTTCACCATTCCTCTCTCT
CAGAAAGGAGAGATCAAGCTACCAACCCTCTCTACCATGTTCGCCTAA
>GmLEA2-89
ATGAAGGTAGGATCTGGTAAAGGGAGAAAAGTGTGCCTGTCGCTGACAGGTGTTGTGA
TTGCAATTGTATTGCTAATTGTAATACTAGCGTTGACAGTGTTCAAAGCCAAGCATCCTG
TTACCACAGTGGACTCAACGAAGCTAGAGGACTTTCACATGGGCTTGGATACACCAAA
ACTAAGGGTCGATTTGAATGTGACCCTGCACGTGGATGTCTCAGTGAAGAACCCGAAC
AAGGTGGGATTCAAGTATTCAGATAGCACCGCCCACCTCAATTACAGAGGGCAGTTGA
TAGGTGAAGTCCCGATCCCTGCCGGAGAGATTTCATCCGGTGAGACCAAAGGATTCAA
CCTCACCCTCACCATTATGGCGGACCGTCTTCTCTCCAATTCCCAGCTTCTCTCTGATGT
AGCGTCTGGTACATTGCCCCTAAACACTTTTGTGATGATGTCAGGGAAAGTCAGCATCT
TAGGCTTTATCAAAGTCCATGTGGTTTCCTCCACTTCTTGTAACGTTCCAATTAATCTTT
CTAATGGAACAGTTGGCAACCAAGAGTGCCAATACAAGACAAAACTTTGA
>GmLEA2-90
ATGAAGCCCAACGACAACATGGCAATGCTGGCCAAGACCGACTCCGAGGTTAGCAGC
CTCACGCAGTCCTCGCCGACGCGATCCCCGCGACGGGCAGTGTACTATGTGCAGAGCC
CGTCGCGAGACTCATCGCACGACGGGGAGAAGACTACGAACTCGTTTCACTCGAGTC
CGCTGCAGAGCCCATTGGGCTCCCCGCCCCACTCCCACTCCAACTCCTCCTTGGGCCA
TCACTCGCGCGAGTCCGCCTCCACGCGCTTCTCTGGCTCGCGCAAGAGCAGCTCCTCG
GGCAACAACCGCAAGGGCCCGTGGCGGCCCTGGAAGGACCAGTTCCATGCCATCGAG
GAGGAGGGCCTCATCGATGCTCACGACAATGCCCGTGGCTTCCCTCGTTGCTGCTATTT
CCCAGCATTTGTCATCGGCTTCGTCCTCCTCTTCTCCGCATTCTCCCTCATTCTCTGGGG
TGCTAGTCGCCCCCAAAAGCCCGCCATTTCTCTCAAGAGTATAACCTTTGATCAATTCG
TGATACAAGCCGGAGCGGACATGTCAGGAGTTGCCACAAGTTTGGTGTCCATGAATTC
ATCTGTGAAAATGACGTTTCGTAACACCGCTACATTTTTCGGGGTCCATGTGACCTCAA
CTCCCGTGGATCTCAATTATTATCAACTTACTCTAGCCACTGGAACTATGCCAAAGTTTT
ATCAATCTAGGAAGAGCCAAAGATCTGTTAGAGTGATGGTGATTGGGAGTCATATACCA
CTGTACGGAGGAGGAGCAAACCTGAACAGTGTGAACGGTAAACCGGTTGAACCGGTG
CCACTGACGTTGAGCGTGATGGTGCGGTCTAGGGCTTATGTTTTGGGCAAATTGGTGA
AGCCAAAGTTCTACAAGAAAATAGAGTGTTCCATCGTTATGGATCCGAAGAAGATGGG
CAAGGCCATTTCACTGGTCAAGAAATGCACTTACCAGTAA
>GmLEA2-91
ATGATGGCTGCAGATCAGCAAAGAATTCATCCTGTTCATGATGTTGAGGCACCACACA
GACCTTTGGTGCCTGAAAACTATGCAAAATCTGATAAGGGTATTCCTCAGCGCACTTTT
CCTGTGATGCATTCAAAGCCACCTAAGAGAAGAAGAAGCTGTTGCTGTAGGTTCATGT
GTTGGACACTAAGCATATTGCTGATTTTGATCATTGCTATTGCCATCACAATAGGGATCC
TATACCTTGTGTTCAGACCAAAGCTCCCCAAATACTCAGTGGACCAACTGAGGATAAG
CCAGTTCAATGTTTCAGACAACAACACCCTCTATGCCACCTTCAATGTGGCAATCACTG
CAAGAAACCCTAACAAGAAGATTGGAATATACTATGAGGGTGGAAGCCACATAAGTGC

TTGGTACATGGACACAAAATTGTGTGAAGGGTCCTTGCCAAAGTTCTACCAGGGTCAC
AGGAACACCACAGTGCTTGATCTGCCTCTAACTGGTCAAGCACCTGATGCAAGTGGTT
TGGTGAATAGAATTCAGGAGCAGCTGCAACAGACCAACAATGTTCCTCTCAATCTTAA
GGTGAATCAGCCTGTGAGGGTTAAGTTTGGTAAGCTGAAGCTCTTCAAAGTCAAGTTC
AGGGTTAGGTGCAGGCTTGAGGTGGATAATTTTGGTGCTAGTAATGATATTAGAATTTCT
AGCAGTAGTTGTAAGTTCAAGCTCAGGCTGTGA
>GmLEA2-92
ATGCTTACCCTTCCACCCCCACCTCCTCCCCGACTCCTAACCACAAGGCAAACCAAAC
AAGTCTCTCCTGACCAAATTGTCATATCAAAACTACCAATCAAACAACATTCTCAAGAA
TCAGATGCACCAAATTATGTTACAACAAAGTCAATTAGGCCGCCGCCACCGCCACCAC
CACCACCACCAATACTCAGGCAACCTCCATTTCAAAGGACTAATCCAATCATATGGTTT
GCTGCAGTGTTGTGCCTCATATTCAGCCTTCTACTCATCTTCTTTGGAGTTGTAACTTTG
ATCATCTTCCTAGGAATCAAGCCAAGAAACCCCTACTTTGACATCCCCAATGCAAACCT
CAATGCAGTGTACTTTGACTCACCAGAATACTTCAATGGTGACTTCACCCTCGTTGCAA
ACATCACCAACCCCAACAAGAAGATTGATGTGAGGTTTGAATCTTTTGATGTCGAGCTT
TTCTTCTCAGACAGGATCATATCAACACAGTCCATTGAGCCCTTTACACAGAGGAGAA
GGGAGAGCAGGTTGGAGTCCTTGCACTTCATATCCAGCTTGGTGTTTTTGCCTAAGGAT
CTTGGTGTGAACCTTAAAGGACAGGTGCAAGGGAACAGGGTTAAGTACAATGTAAGA
GGAACCTTTAAAGTGAGAGTAAGCATGGGGTTTTTCCATTTGTCTTACTGGTTGCATAG
CAGATGCCAGATAGAGATGACTGGTCCACCAACTGGTGTCTTGGTAGCTAGGAAGTGT
ATCACAAAGAGATGA
>GmLEA2-93
ATGTCTCAATTAGCACCAAAACAATTCATGACTGAATCACAAAGTAACCAGGACCACG
AGCAAGTGGTGGTGATCTCACAGAAAAAGCTAAAGAGGAGAAGAGTGTGTGTGATGG
TCACAGGAGCAGTGCTGTTACTGCTCATTGTGCTAGTCATAGTGGCAATTATCTTGGCC
TTTACCTTGTTCAAGACCAAAGAACCAAGAACTCAGCTTGTGTCTGCCACTTTAGAAG
GTATAGCACCTCGTCTCACACTTCCTGCCATTGACCTACAAATCAATGTCACACTTGAC
CTCAAGGTTCGGGTTGAAAACAGAAATCGTGCTAGTTTGAAGCATGAAGGGGGAAAG
AGTGTGTTGCTGTATAAAGGAAAAGAGGTTGGAGATGCTTATATAAACCCTGGTCTTAT
TCCTTCAAGGGGTTCTACTATTCTTCCATGCAGACTCACCCTTCAGGTGGAAAAGCTAG
CTTCCAACTTGACCAGTTTAGTTGGTGATTTGATGGGGGGAGAGATCTCTATGGACACA
GTTACTAGAATTCCTGGGAAAGTTACCTTCCTTGGATTCATCAAGAAACATATTGTTGC
AGAGTCCAATTGCCAATTCACTATTAGTGTTTCTGAGTTGAAGATCACAAACCAAACTT
GCAAGAGTAAGGCCAAGTTATGA
>GmLEA2-94
ATGAAGAAGGGATCTGGCTGCAAAGGAAGCATTGTGTGCCTGATGGTAACATTATGTGT
AGTGATTGCACTTGTATTGCTAGGAGTGATCTTAGCAATGACAGTGTTCAAACCTAGGC
ACCCTATTACCAATGTGGATTCCGTAAGGCTTCAAAACATGAGCTTGGACATGGACATA
TTTAGCATGAGTGTAAATGTGAACTTGACATTGGAAGTGGATGTTTCTGTTAACAACCC
TAATAAGCTTGGATTCAATTACTACAATAGCTATGCTCAACTCAATTATAGAGGACAGTT
GATTGGGGAAGCCCCTATACCCAATGGACACATATTAGCTGAGGAGATTAAGGGACTCA
ACTCGACACTAACTGTTATGGCTGACCGTTTGGTCTCAAATTCTGAGGTGACCAAGGAT
GTTGCATTGGGTTTATTGCCCCTCAACAGCTTGGTGAGAATTTTTGGCCAAGTCAACGT
TTTAGGGTTTATCAAATTCTATGTGGCTTCCACCTCATCTTGTGATTTCACCCTCAATCTT

TCCAATAGAACAATAGTGGACAATAAGTGCCAAGAGAAGACAAAGATTTCGGGTTGA
>GmLEA2-95
ATGCGTGTGGGTTTGGGTGTTAAGGTATCGTTGGGTGTGATCGGTGTGAGTCTAATATT
TGCAGTGCTGAAAATGTCGCAGTTGTTGGACAAAGCCAAAAACTATGTTGCGGAGAA
AGTCACGAACATGCCCAAGCCCGAGGCGAGTGTCACCGACGTGGATTTCAAGCGCGT
GAGCCGCGACAGCGTCGAGTACTTGGCTAAGGTCTCTGTTTCCAACCCTTATTCCACTC
CCATTCCCATTTGTGAGATCAAGTACTCCCTCAAAAGTGCTGGCAAGGAGATAGCATCA
GGGACAATACCTGACCCAGGGTCACTGAAGGCAAGTGACACAACAATGCTGGATGTG
CCAGTGAAGGTGCCTCATAGCATATTACTGAGCTTGGCAAAGGACATTGGTGCAGATTG
GGACATTGATTACCAATTGGATCTTGGTCTAGTTATTGACCTTCCTGTCATTGGCAACTT
CACCATTCCTCTTTCTCAGAAGGGAGAGATAAAGCTCCCAACCCTCTCTGACATGTTTG
CCTGA
>GmLEA2-96
ATGTCCCAGTTGAACGGAGCCTACTATGGCCCCTCCATCCCGCCGCCGAAAACCTCCTA
CCACCGCCCAGGGCGCGGCGGAGGCTGCTGCTGCGGCTGCCTCTTCAGCCTCATCTTC
AAGCTCATCTTAACCGTGATAATCATCGTTGGCATCGCCGGGTTCGTGTTCTGGCTCATA
GTCCGTCCGAACGTGGTGAAATTCCACGTGACCGACGCCACCCTGACGCAGTTCAACT
ACACCGCCAACAACACTCTCCACTACGACCTCGCCCTAAACATCACGGTCCGAAACCC
CAACAAGAGGCTCGGAATCTACTACGACCGCATCGAGGCGCGTGCAATGTTCCACGAC
GCAAGGTTCGATTCACAGTTCCCGGAGCCGTTCTACCAGGGCCACAAGAGCACGAAG
GTGCTGAACCCAGTCTTCAAGGGTCAGCAAGTGGTGCCACTCAACGCTGAGCAAAGC
GCGGAACTGAAGAAGGAGAACGCCACTGGGGTGTACGAGATCGACGTGAAGATGTAC
CTTAGGGTGAGGTTCAAGCTGGGGGTGTTGAAGACCAAGACGCTCAAGCCCAAAGTA
TCATGCGACTTGCGTGTTCCTTTCAAAGGAAGCGCCGCCTTTGAGACCACCAAGTGCC
ACTGGGATCGCTGA
>GmLEA2-97
ATGGCCGATAAGCAACCCCACCTGAACGGTGCTTATTACGGTCCCGCCATTCCCCCGGC
GGAGCAACCACGCTACCGCCCTCACCGCGAAAGAAGCTGCTGCTGCTGCCTCTTCGG
AATCTTGTGGAAGATTCTGGTTGCACTCATTGTCCTCGTTGGCCTCGCAGTACTCATATT
CTGGCTGGTGGTTCAACCCCGCTACTTCAAGTTCTACGTCACGGAAGCCGACCTAACA
CAATTTGATTACTATAGCAACAACAACACCCTCCACTACAACATGGTCCTCAACTTCAC
TGCACGCAACCCCAACAAGAAGCTCAGCATATACTACGACAAGGTTGAGGCATTAGCA
TTCTACGAGGATGTCAGGTTCGCCAATTACAGTGTGATAACGCCCATGAACTCCTTCCG
GCAGTACAAGAAGAGCAGCAGCACCATGAGCGCTGTTTTATCGGGGCAGCAAGTGTT
GCCGCTCGACAACGACCTAGTCTCAGAGTTGAACCAAGACAAGATTGGTGGGGTGTA
CGAGATCTATGTGAAGCTCTACTTCAGGATTAGGTTCAGGCTCGGGGATGTCAAAACCC
GTCGCTTCAAGCCCAAGGTCAAATGTGATGCGAAGGTTCCCTTGAGGACCATGGGCAA
CGTAACTCTGTTTCAGACCACCAAGTGTGATGTCGATTACTAG
>GmLEA2-98
ATGGCCGATAAGCAACCCCACTTGAACGGTGCTTATTACGGTCCCGCCATTCCCCCGGC
GGAGCAACCACGCTACCGCCCTCACCGCGGCAGAAGCTGCTGTTGCTGTCTCTTCGGA
ATCTTGTGGAAGATTCTGGTTGCACTTATTGTCCTCGTTGGCCTTGCAATCCTCATTTTC
TGGTTGGTGGTTCAACCCCGCTACTTCAAGTTCCATGTCACGAAAGCCGACCTAACAC
AATTTGATTACTATAGCAACAACAACACCCTCCACTACAACATGGTCCTCAACTTCACT

GCACGCAACCCCAACAAGAAGCTCAGCATATACTACGACAAGGTTGAGGCATTAGCAT
TCTACGAGGATGTCAGGTTCGCCAATTACGATGTGATCACTCACATGAACTCCTTCCGG
CAGTACAAGAAGAGCAGCAGCCCCATGAGCGCTGTTTTCACGGGGCAGCAAGTGCTG
ATGCTCAACAATGAACAAGTCTCAGAGTTGAACCAAGACAAGAATGCTGGGGTTTATG
ACATCTATGTGAAGCTCTACTTCAGGATTAGGTTCAGGCTTGGGGACGTCATATCCAAT
GACTACAAGCCCAAGGTCAAATGTCACCTCAAGGTCCCTTTTAGTAAAAATGGCACCT
TTACTCTGTTTCCGACCACCAAGTGTGATGTCGATTTCTAA
>GmLEA2-99
ATGTCGGTTAAGGAGTGCCACCACCACCACAAGGGAAAGAAGCACAAGCTCTGGAGA
CGCATCTTCTGGGGAATAGTGATCTTCGCCTTCATCGTGTTACTGACAGTTCTTATAATC
TGGGCAATCCTAAAGCCTTCAAAACCAACCTTCATCCTCCAAGACGTCACCGTTTACG
GCTTCAATGCCACCATCCCCAACTTCCTCACCTCAAGTTTTCAGGTCACGCTTTCCTCG
CGCAACCCCAACGACAAAATCGGAATCTACTACGACCGTCTCAACACCTACGTAACTT
ACCGGAACCAGCAGGTCACGTACCGAACCTCAATCCCACCCTCGTACCAAGGTCACA
AGGAAGAGGATGTGTGGTCCCCGTTTGTCTACGGCACTAATGTCCCCGTCGCACCCTA
CAACTTCGTTGGCCTCAGTCAAGACCAGACCAACGGCAACGTCCTCGTCCTCGTTAAG
ATTGACGGTAAGGTACGATGGAAAGTTGGTTCCTTCGTCTCTGCTCACTACAACCTCAA
TGTAAGGTGCCCTGCTTTCATAACCTTTGGCCCCCAAAGCAACGGGATTGCCCTTGGC
AATAACGCCGTTAAGTATCAATTGGTTCAACGCTGCACCGTCGGGGTTTGA
>GmLEA2-100
ATGTCAAAGGACAAAGTTTCCGGTGATCCAAGACGTGCAGTGTGCACAGGTATCACCA
TCTTCCTCCTCTTAGCCGGCGTCACTCTCCTCGTCCTCTGGCTGGTCTACCGTCCCCAC
AAGCCGCGCTTCACGGTCATCGGCGCCGCCATCTACGGCCTCAACACCAGCACGCCGC
CGCTCATGTCAACCACCATGCAGTTCTCCGTCCTCATAAAGAACCCGAACAGGCGTGT
CTCCATTTACTACGACAGGTTCTCCGCCTTTGTCTCGTACAGGAACCAGGCCATAACGC
CGCAGGTTCTGCTGCCACCCCTGTACCAGGAGAAGCGCAGCTCGGTTTCGGTGTCCCC
GGTGATCGGAGGCACGCCGCTCCCGGTGTCGGTGGAGGTTTCGAACGGGTTGGCGAT
GGACGAGGCTTATGGGGTGGTGGGTCTGAGGCTGATATTTCAGGGCAGAGTGCGGTGG
AAGGCTGGGGCCATCAAAACTGCGCACTATGGGCTCTACGTTAAGTGCGATGTTTTGAT
GGGTTTGAAGAAAGGGTTGGTGGGTCAAGTTCCTCTCCTCGGAGTTACACCCTGCGAT
GTCGATCTATGA
>GmLEA2-101
ATGTCTACATCTGATAAGTCAGAAGTGGTGGAAAGGGGTGCTAAGGATGATGAAAAGC
CAAAGGAAGAACATGAGAAGGGTGGATTTCTTGATACAGTGAAGGATTTCATTCAAGA
CATTGGTGAGAAGATTGAGGGAGCAGTTGGGTTTGGGAAGCCAACTGCAGATGTCAC
TGGGATACACATTCCATCAATTAATCTTGAGAAGGCAGAGCTAGTTGTTGATGTTCTTAT
AAAGAACCCGAATCCGGTGCCAATTCCCCTAATTGACATAAACTACTTGGTCGAGAGT
GATGGGAGGAAACTAGTTTCGGGATTGATACCAGATTCGGGCACAATCCATGCACATG
GAGAGGAGACAGTCAAGATTCCTCTTACTTTGATTTTTGATGACATAAGGAATACCTAT
GATGATATTAAACCAGGAAGCATCATTCCTTATAGGGTGAAGGTTGATCTCATTGTGGAT
GTTCCTGTCTTTGGAAGGCTCACTCTACCTTTGGAGAAAACTGGAGAAATCCCCATTCC
ATACAAGCCTGATATTGATCTCGATAAAATTCAGTTTGAGAGGTTTTCTTTTGAAGAGA
CAGTTGCTATTCTTCATTTGAAGTTGGACAACAAGAATGACTTTGACCTTGGCCTCAAT
GCTCTTGATTATGAGGTCTGGCTTGGTGATGTTAGCATTGGAGGAGCAGAACTTGCCAA

ATCTGCAAAGCTCGTGAAAAGTGGGATTAGTGACATTGATGTTCCAATTACCTTCAGGC
CTAAGGATTTTGGCTCTGCTCTTTGGGACATGATTAGAGGAAGGGGGACAGGCTACAC
TTTTAAAGGACATATTGATGTTGACACTCCCTTTGGAGCAATGAAACTGCCCATCACGA
AAGAAGGTGGTACTACCCGTCTCAAGAAGAATGAAGACAGTGGAGATGACGAGGAGT
GA
>GmLEA2-102
ATGCACAACAACGACCACATACCCGTTCACCACGTCCAGGGTCCGAACCCGAAGCCC
GTTAAACTGAACCGGCACCACACGATGCGGTACTATGCCCACCGTGTCCACGAAAGCC
TCACAACCCGCGTCTCCAAGATGATCTGTGCCACCTTCTTGGGCCTTCTTTTCATTGTG
GGCCTCATCACCTTCATCCTCTGGCTCAGCCTCCGGCCCCACAGGCCCAGGTTCCACAT
CCACGAGTTCAACATACCGGGCCTGACCCAAGATTCCGGGTTCGAAAACGCCGTCATA
ACGTTCAAAGTATCCGCGCGAAACTCCAACCAGAACATCGGGGTTTACTATGAGTCCA
TGGACGGCGCCGTTTATTACCGCGACACGAAAATTGGGTACACGCCGTTACTTTACCCG
TTTTATCAGCAGCCCAAGAACACGACGGAAGTGGACGGCGATCTTAGTGGGGCGACG
TTGACCGTTAGTAGTCAGCGCTGGTCGGAGTTCCAGAGCGATAGGGCTGACGGTAGCG
TGGTGTTCCGCTTGGAATTGACGTCTGTGATCAGATTCAAGATATCCACGTGGGACAGC
AAGCGCCACACGATGCACGCCAACTGTAATGTGGGCGTGGGACCCGATGGTTCCCTCC
TGACCATTTATAAGGACAAGAGGTGCCCCGTTTATTTCTCTTGA
>GmLEA2-103
ATGGCAGATCATCAGAGACTGAGGATCCACCCTATGGAGGGGGAAGCACCACCACCAC
CCCCAACAACTCCATTGGTACCTCCAGGCTCGTCAAAATCAGAAAAGCGTATCCCTTTA
CACCATCCTCCACAACTACGTGCTATGCCAGCAGCATACCCTACAGCACACAAAAGAA
GCTGCTGCTGCAAGTGCATATGTTGGATAATAACCTTGCTTGTTCTCCTCCTCATTATCT
TGGCTGCAAGTGTTGGAATCCTCTACCTAGTCTTCAAACCAAAGCTTCCTGATTACTCA
GTTGACACCCTCAGGATAAGTGATCTGAGGCTTAACTTTGACATGAGCCTCTATGCAAG
GTTTGATGTGAAGATCACAGCAACCAACCCAAACAAGAAGATTGGCATATACTATAAA
AAGGGTGGAAGGTTGAGTGTGTGGTACACAAACACAAGGCTTTGTGAAGGTTCACTG
CCACAGTTCTACCAAGGTCATGAGAACAAAACAATGCTCAATGTGTCCTTGAGTGGTC
AAGTGCAGTCTGGAAGCACCTTAATGAATGCACTGCAGCAGCAACAGCAGACAGGGC
GCATTCCATTGGATCTCAAGGTGCATGCACCAATAGCCATCAAACTTGGGAGGTTGAA
GCTTATGAAGGTGAGAGTCTTGGGAGAATGCCTTTTGGTGGTGGATAGCTTGTCATCTA
ATAATCTCATAAGCATCAAGGCTAGCAACTGCAAGTTCAGATTGAAACTTTGA
>GmLEA2-104
ATGGCCACCACAATGCCGAAATTCAAGCGAAAACTTGTGATGGGTGCAAATGGTCGCA
CCAACCCCTTGGTGTGGCTAGTGGCGATTATCTGCACCATCATAGCAGTAGCAGTGGTA
GTTGTAGGCATTGTAGTGTTCATTGGTTACATAGTGATACACCCTAGAGTGCCTGTGATC
AGTGTCACCAATGCACATTTAGATCTTCTCAGAAATGACTATGCTGGTTTGCTCCAAAC
CCAACTCACCATCGTCGTGACTGCGAAGAATGGGAATGCCAAGGCTCATGCATCCTTC
TCCGCGATCACCTTCAACATCAGCTACCAGGGCCAAGACATTGCTGTGCTTGTTGCTGA
CCCTTTTGAGGTGCCAAAGAACAGCTCAAAGGACCTCAGCTATGTTGTCCAATCTTCTT
CTATACCCTTGACTCCTGATCAAATGGAGGAAGTGAATGATGCTTGGAAGAGGAATGA
AATTGAGTTTGACTTCAAAGGGGCTGCAAGGACACAGTGGAGGGTAGGGCCTTTAGG
GTCTGTGAAGTTCTTGTGCCATCTTGATTGTGACCTCAAGTTTCGCCCCGTGAATGGGA
CTTACATTCCCTCGCGGTGCACCTCCAAATCACACTGA

>GmLEA2-105
ATGGCCGATAAGCAGCCACACTTGAACGGTACCTATTACGGTCCCGCCATTCCCCCGGC
GGAACAACCACGCAATCGCCATCACCACGGAAGAAGCTGCTGTTGCTGCCTCTTTGGA
ATCTTGTGGAAGATTCTGGTTGCACTCATCGTCCTCGTTGGCCTCGTATTCCTCATATTC
TGGCTGGTGGTTCAACCCCGCTCCTTCAAGTTCCAGGTCACGGAAGCGGACCTAACCC
AATTTGATTACTACACCAACAATCTCACTCTCCACTACAACATGGTGCTCAACTTCACT
GCACGCAACCCCAACAAGAAGCTCAGCATATACTACGACAAAGTTGAGGCATTAGCAT
TCTACGAGGATGCCAGGTTCGCCAATTACGATGTCATAACTCACATGAACTCCTTCCGC
CAGTACAAGAAGAGCACCAGCCCCATGAGCGCCGTTTTCTCGGGGAAGAAGGTGTTG
ATGCTCAACAGCGAACAAGTCTCTAAGTTGAACCAAGACAAGAGTGATGGGGTTTATG
ACATCTACGTGAAGCTCAACTTCAGGATTCGGTTCAGGCTCGGGGACTCCATATCCGGT
AACTTGAAGCCCAAGGTCAAATGTCACCTCAAGGTTCCCTTCAGTAAAAGTGGCACCT
TTACTCTCTTTGAGACCACCAAATGTTCTGTCCATTTCTAA
>GmLEA2-106
ATGGCAGAAATGCAGATACAACATCAAGAAGACCATAACTATAACCCTAATCACATCCC
TCGCCCTAGCCCTAGCCCTAACCCTACCCAATACACACAATACCGTTTCCCTGGCAAAG
TTCCCCACCACCAACACCAAGTCCACAACATGGACAAAGCCACACCTTCTCGCTTCAA
ACCTAATGCCCCCAAACGTCAACACTGCATTTGCATAACCGTTTTCCTCCTCTTACTCG
GCATCATACTCCTCGTTCTCTGGCTGGCCTACCACCCTAACAAGCCGCGCTTCACGGTG
GCCAGCGCCTCCGTCTACAGCCTCAACGCCACGTCGCCGCCACTAATGTCCATCGCCAT
GCAGTTCAATGTGGTCATAAAAAACCCTAACAGGCGCGTCTCCATTTCCTTTGACAGG
CTCTCGGCCTACGTGTCCTACCGCAACCAGCCCGTGACGCCGCACGTCATGCTTCCGC
CGCTCTTCATAGAGAAGAACAGCGCCGTGTCGCTGTCCCCGGAGATCGGAGGAGTGG
CGGTGCCGGTGTCGGAGGACTTGACCAATGGAATGGCCATGGACGAGAATTATGGGGT
GGTGGGCGTGAAACTTGTGTTGTCCGGAAGGTTGAGGTGGAGAGCTGGTGACATCAA
CTCTGCACATTATGGTTTCTATGTCAAGTGTGATGTTTTGATGGGTTTGAGAAAAGGTTT
TGTGGGTCAGGTTCCTCTTCTTGGAGCTCCAGTTTGTGATGTCAATACATGA
>GmLEA2-107
ATGCCTAGGGAAAACCCTGATTACGAGCTCTTCTTTTGCCGCATGGCTGCCACATGCAT
TGCCTTCCTAATCATTATCCTAATCTTTGTTCTGTGTATTATCTTTTCAACTTACACACCC
TCCTTCACAGTCACGTCGATCACCGTCTCCAAATTCAACGTCACCACTCACGCAGAGG
AGTTAACGGCGACTTTCAACGTTGAGGGCATTCTTGAAAACCCTAACCTGGCGTACTC
CATAAGGTACCAGAGCCTGGACCTCGCGCTCTGGTTCGACAACTTCACCATCGCCTCC
ACTACAATCAAACAACCACCATTCTCCACCCAAGGCCAGACCGACACCCCAGTTCGGG
CCCAGTTTGCCATGGCCCGCAGGTGGTTACCTAGCGGCTTGGCCAGTGAAATTGTGGC
GCAACGAAATTATCATGGTTCAGTGGATTTTGGAGCCACATTGGTTGCTCGGTTCAGGT
ACAAATTTGGCGTGTTGCACTCCAAGAAGGTTCATCATTTCAAGCTTTATTGTCATCCG
TTGCATGTTGCACTCCATTCTAACAGTACTACGGGAAAGTTGGTTGCTCTTGTTGATTGT
AAATCAGTTTAA
>GmLEA2-108
ATGCCTTGCAAACCCCGCTGCATCTACTGCACAATCTACATATTTATATTCATTTTCATGC
TTTGCTTCTTCCTCTTCTTGATAATAATCAACCCTTCAAGTGTCAAGTTCTACGTAACTG
AGGCCACCCTCACACAATTCAACCTCACAAGCAACAACACCTTGTATTACAACTTCAA
GGTCAACATCACAGTGACAAACCCCAACAAGCACATGATAGTGTACTATAGGACGATC

AAAGCAATAGCTTGGTACAAAGATAATGAGTTTGATAGGGTGAGCTTAACACCCTTTGA
CCAAGGCTACAAGAATACCACCTTTCTTCGAGCAGTGTTTGTAGGGCAAAGTGTGATTA
TGCTCAAACCTAAACAACTTGGTGAGTACAAAGATGAGACAAGTATTGGGATTTACAA
TGACTTGGCTGTGGATTTTGATCTTAGAATTAGAGCCAAGTTTGGAAGAATTAAGAGCA
GGCGATTCAAATTTGATTTCCTAAAGTACTTGTGCGGAGAGAACTGTAGGAGTTTTGGC
AATTAA
>GmLEA2-109
ATGGTACATAATTGCTCCTCCACCGCCTCCCTGATTGGCCATAACTTGTGCCACGTTATT
CAAGGCCTCGGTAAGAAGTCATTCTCGCTCATTTCTCGCAGTCATTACTCTCTATGTCAC
TCGCTCAAGCGTTCTTCGTTAGCAATTGTGACGTTTCTGGTGCTTTCTAGAGCTCCTCC
TCCGATTGCTCTGTTAAGTGTCGCATCGTTCAAAGGGTGTTGTTGCTGTCTCTTCCTTCT
ATTCCCGTTCCTAGCGTTGCTGGTGCTAGCGGTGGTGCTGGTAATAATCGTAGCGGTGA
AGCCAAAGAAGCTGCAGTTTGATCTGGAGCAACTGGGGGTGCAGTACATGGGTATCAC
ACACAATCCCCCCTCAATCGCTTCCCTCTCTCTCACCATTCACCTCCTCTTCGCTGCCAC
CAACCCCAACAAGGTTGGGATCAAGTACGGCCAGTCCAGCTTCACTTTCATGTACCGC
GGCATCCCATTGGGAAAAGCCACCATTTTCGACTTCTTCCAACAGCCTTACAGCACCC
GACAACTCATCGCCGTTGATCGCGTCAATTTGCTTGAGGTCGATGCCGCTGATTTGATT
CGAGACGCCTCCCTTAGATTCTTGGGCGACGTTGCTGCCAAGATCCGCTTTAACCTGA
GGACTGCATCTGGAGACGTTGTCCCTATCAACTTAGAAATTGAAGATACTTGTCGGCGT
AGCAACGCTGCAAGAAGAAGAAGGGAACAAGACACAGAAGGAAGCAGTTACACCTC
GCCTCCTCTTTCTCCGCATCACGCTAAAATGGACGGGGAACCGGCACGAAGAGTCACC
CTTGAGGACTTCTCCAATACCGCCACTCCTCATTTCTTCACGAGCATTGCAAAACCGGA
GGTACAAGCCGCCAATATTTGCTACCCACACTCCCTTATTCAGCTAATCCAAGGGAACC
TCTTCCATGGTCTTCCAAGCGAAGATCCATATGCTCACCTCGCCTCGTACATAGAAATAT
GCAACACCGTAAAAATCGCTGGAGTCCCAGAAGATGCGGAAGAAGTAGTGGAAAAGT
TCTTAAAGAAGTACTTTCCAGAGTCAAAGACTGCTGAAGGGAAAATGGAGATTTCTTC
TTTCCATCAATTCCCGGATGAGTCCCTTAGCGAAGCACTAGACCGTTTTCACGGGCTGT
TACGAAAGACACTGACACACGGATACAGCGAGCCGGTACAACTGAACATATTCATCGA
TGGCTTGCGGCCTCAATCGAAACAGCTACTAGATGCATCTGCAGGGGGAAAAATCAAA
CTGAAGACTCCCAAAGAAGCGATGGAGCTCATCGAAAACATGGCAGCCAGTGATCAA
GCAATCCTTCATGATCGTAGCTATATGCCCACAAAAAGAAGCCTTTTGGAACTTGGCAC
ACAGGACGCGACATTGGCACATAACAAGCTGTTGACGAGGCAGATAGAAGCCCTTACC
GAAACCCTCAGCAAATTACCTCAGCAATTACAAGCGGTAAGTTCTTCCCACTCTTCTGT
TTTGCAGGTAGAAGGATGCCCCACATGCGGAGGAACCCATGAGCCTGGACAATGTGTA
AGCCAACAGGATACCTCTCAAGAAGTAAACTATATGGGCATACCAAATCGCAGATTCCA
GGGTTACAACCAGGGGAACTCATCTGGATTCCACCAAGGGGGAGCAGGATTCAATCAC
GGACCACCGGGATTCAATCAAGGAAGAAACTTCATGCAAGGT
TCAGGGTGGAGGAATCAGGGAAACCAGTACAAGGAGCAAAGGAACCAACAACCATA
CCAACCGCCATGCCCGCATCCCAGCCAGGGCCCCAATCAGCAAGAAAAGCCCACCAAT
ATAGAAGAACTGTTGCTGTAA
>GmLEA2-110
ATGTCTCTTCTCTTCTTGATCATTGTTGCTATTGTGATTGTTGCATTGTTTTTTACCATCTT
TAGACCCAAGGATCCTAACATCATTGTCCACCCCGTTGGCTTCGAAAACTTTAACCCTT
CCCTCTTAACCAATGTGACCGCGAATGTGAGCCTCGGCATGCTTATCACTATCGAGAAT

TCAAACTATGAAAACTTTGAGTACCCAAATGCCACTGGTTATATCAAATTCCATGACAC
CGTTGTGGGCCAAGTTCCAATTGTGGGAGAGTTGGTCCCACCGCGAAGCCAGATTAAT
GTGAACACTTCGGCAAATTTCATGGTAGCAAAGTTAATCAATGACCCCAATTTTTTGTC
AGATTTTCTAAGTGGAATTGTGAATTTCACATCAACAGCTTCACTGCCTGGGAAAGCGC
ATATGCTAAAGATTATCAAATTCAAGGCCACAGTTTATAGCTCGTGTGACATCTCTCTTA
ATATAACCTCTAGGAATGTTGATTCCAAATGCATATCCAAGATCAAGCTTTGA
>GmLEA2-111
ATGGTGGCATGTTGCAGTATGAGGTGTTCCAAAGGGCTTAAAATCTGCTGTGGTGTCAC
TGCCCTTCTTCTAATTATATTAATAGTAGTACTGGTGGTCTTGTTCTTCACTATCTTCAAG
CCTAAAGACCCCGACATTGTTCTGCAGTCAGTTAAGCTTGACGGTTTCAAACTTGAAG
TCTTCCCAACTCTGAAACTAAACGTATCACTAGGCATAGTGGTGACGGTTGAAAACCC
AAACCATGGAAGCTTTACCTACCAGAACAGCACAGCTTTTCTTTACTATCGAGGAAATC
TTGTTGCTGAGGCTCCACTACACCAGGACACAATTCCAGCTCGTAACGATCATAATATA
AGCACATCTTTGACTATTTTTGTAGATCTCACAAAATTTAAGGATTTACCCAGTGATTAT
TCCGGGGGCGTTATAAATTTTACCTCAACAACCACCTTACTGGGGAAAGTCAAGGTATT
GGACCTTTTCAAGATCAAGGCCACTAGTTACTCCACCTGTGATCTCTCTCTATTCGTAAA
TGACCAAACCATCAATTCTACGTGCAACTCTGAAATCAAATTCTGA
>GmLEA2-112
ATGTTTTTTTTTGCTGAATCCCCTAATGATATATTGAAACCGCGCCGCAACATAGAGCCC
TCTCTCTCTCTCTCCCTCCCTCTCTCTTCCCTCTCTCTCTGGGAAGAACAAAGTAGAAG
AAGCAACAGAAAACAGAACCACCGAACCCACTCAGAACCACCAGTGCTTCGAAGTAG
CGGCGCAAAAAACCGCTCTGCAACCCGCACCGCTCCGCCGCGACCCAAAACTGCGCC
GCTACACATGTGCCCGTCGCGGATGGGTGCCACTCTGCTCCGCCCTGCTGCTATAGCGG
CCGCAACGGCTGCTATTGACAACTATGTTAACACCACTTATTCTGATTGTTCAGACTTC
AGTGACTATTCCCTTGCATTGAATTTAGTTCTTCGGTTCTGTCAAGCAAAAAAGCAATA
CCATCACTCCGCTTTTAATGGCAGTTCCGTTGAACAGCCTTTAAAGTGTAATCCTACGC
ATCCCCGTTCAACAGTTTCTCTTCTCAATTTTCGTGACTGCCGACTATCATTTGATAACG
GATCCTTTGAAGGAACAAGCAGAATAAAGGTAGGATCTGGTAAAGGGAGAAAAGTGT
GCCTGACGGTGACAGGTGTTGTGATTGCAATTGTATTGCTAATTGTGATACTAGCGTTG
ACAGTGTTCAAAGCCAAGCATCCTGTTACCATAGTGGACTCAACGAAGCTAGAGGACT
TTCACGTGAGCTTGGATCCAGTAAAACTAAGGGTAGATTTGAATGTGACCTTGGGAGT
GGATGTCTCAGTGAAGAACCCGAACAAGGTGGGATTCCAGTATTCAGACAGCGCTGCC
CACCTCAATTACAGAGGGCAGCTGATAGGTGAAGTCCCGATCTCTGCCGGAGAGATTT
CATCCGGTGAGACCAAAGGATTCAATCTCACCCACACCATTATGGCCGACCGTTTGCTC
TCCAATTCTCAGCTTTTATCTGATGTCACATCTGGTACATTGCCCCTAAGCACTTTCGTG
AGGATGTCTGGGAAAGTCAGCATCTTAGGCTTTATCAAAGTCCATGTGGTTTCCTCCAC
TTCTTGTGATGTTGCAATTAATCTTTCTAATGGAACTGTTGGGAACCAAGAGTGCCAAT
ACAAGACAAAACTTTGA
>GmLEA2-113
ATGCTTATCACTATAGAGAATCCAAACTATGAAAACTTTGAGTACCCAAACGCCACTGG
TTATGTCAAATTTCTTGACACCGTTGTGGGCCAAGTTCCAATTGTGGGAGAGTTGGTCC
CACCACGTAGCCAGATTAATGTGAACACTTCGGCAAATTTTATGGTATCAAAGTTAATC
AATGATCCCAATTTTCTGTCAGATTTTCTTAGTGGAATTGTGAATTTCACATCAACAGCT
TCACTGCCCGGGAAAGCGCATATGCTAAAGATTATCAAATTCAAGGCCACAGTTTATAG

CTTATGTGACATCTCTATTAATATAACCTCTCGCAATGTTGATTCCAATTGCATATCCAAG
ATCAAGATTTGA

The amino acid sequences:

>GmLEA2-1
MPPHRDRDHDHDDDHVAEHHHHHHHHRPIRRKLVNTHHSGKTHPLIWLAAILCTIIAIGVVI
AGIVVFVGYMVIHPRIPVISITNAHLDLLSNDYTGLLQTQLTIIVVAQNGNAKAHATFSDIR
FNLSYQGQGIAVMLAPPFDVAKNSSKPLNYVVRSASIPLTPEQMEEVDESWKRDVIGFDLK
GSARTRWRVGPLGSVKFWCNLECQLRFRPSNGSYIHHSRCTSESK

>GmLEA2-2
MAKSIQQERRSGKCFVYLLAAFVILCALVLVFASLLRVKNPYLKLRSATSNKISYSTSPSFN
ATLIIFLGIKNPNFGAFSYNNNRVSVLYAGVKIADRQINGGRVRFRETKEINVTVKLMSAKA
PISENLSIDISSGSLNLTSNVKFSGTVHMLKIINIRKTIEMACAMKLNLTSHTIQGIQCQ

>GmLEA2-3
MCPWAWVMFHFATSIHPSSHSNTERAMAKPENRTNLASCFVATVFLIFLLIVVFIVYFTVFK
PQYPKIAVSAIQIPSFSATNGTVNFTFSQYASVRNPNRGTFSHYDSSLQLLYYGRQVGFMFV
PAGKIAAGRTQYMAATFTVQSFPLGLGPTSVDGPSSVGPTMEMESRIEMAGRVRVLHLFS
HHVEAKAQCRVAIAINDGSVLGFRC

>GmLEA2-4
MPAISPAYYAPNYNNLLHHSPLYNEINALSQISNIFVKSLCIWLLQVIGLLSLIVLCLWLALR
PKSPSYSIVFISIGQPSNSNENNTIFYNLDIENPNKDSSIYYDDTILSFLYGEQEDDVGETTIG
SFHQGTGNTRDVSDTVNAKPRPFKPLLNAISNATRVKSCFDHKISIQDMGN

>GmLEA2-5
MSECEHHKGKKRKIFRQVFWCIVVFLFLVLLTILLIWAILRPTKPTFTLQDVTVYAFNATIPN
FLTSNFQVTLISRNPNDNIGVYYDRLEIYVIYRSQQITYRTAIPPTYQGHNEINVWSPFVYGT
NIPVAPFNFLRLSQDQSDGNVLVTIRADGRVRWKVGAFISGRYHFYVRCPAFISFGPRSNGI
VVGENAIKFQIIQRCSVSV

>GmLEA2-6
MSHLNGAYYGPSVPPPKSYHRPSRGGRDGCCCGCLSCFCGCIFDCILGLICKILTTILIIVAIL
GFLFWFIVRPNVLKFHVTDASLTRFDYTTNNTLHYDLALNVSIRNPNRRVGVYYDHIEAH
ALYQDVLFGNQTLGPFFQHHKNTTFVNPLFKGQRVTPLAGNQVEVFDKEKGSGVYTIDL
KLFMVVRFKFLLFKSASVKPKIRCALHVPLKSRNATTTISPDAAFQPTECGWDYGKKWWI
H

>GmLEA2-7
MADPQKIHPVHHDVEAQNHPSAPLVPRSMSKSDAGDPQRVVVQQQQQNIPLKQTKPPPK
KRRSCCCRFFCWLISILLILIVAIGITVGILYLVFRPKLPKYSVDELRVTHFDLADNNSLSVTF
NLTITARNPNKKIGIDYRGGSHISAWYMDTKLCEGSLPKFYQGHRNTTVLSIPLTGKTQDA
TGLQSTLQNQLQESGNVPLNLKVKQPVRIKLGKLKLFKIKFRVRCKIVVDSLSANSSIRIQS
SSCKFRFRL

>GmLEA2-8
MADRVHPSHSPSVSADSQPASPQDSSVVPKPPLPPSSEKPVPPPGTYVIKIPKDQVYRVPPP
ENARRYDQYTRRKHRRSRCCCCFCWLIGILFILVVFLAIAAGVLYLVFRPEEPKYSIENIAV
RGINLTSPSSTAAMSPVFNVTVKADNPNDKIGIRYLKDSSAEVFYKDARLCNGALPAFYQP
SNNVTVFGTALRGDGIELRSEVRRALLEAQTKRRVPLTVRIRAPVKIKVGSVKTWKITVKV
NCHMTVNELTARAKIVSKRCNYDVDLW

>GmLEA2-9
MSTSDKPEEVERGSKDEKHKEDDQEEGKGGFIEKVKDFIHDIGEKIEEAIGFGKPSADVTAI
HIPSINLHKADLVVDVLIKNPNPVPIPLIDIDYLVDSDGRKLVSGLIPDAGTIHAHGEQTVKI
PVTLIYDDIKQTYADIKPGSIIPYRVKVSLIFDVPILGRLTLPLEKTGEIPIPYKPDIDLEKIHFE
RFSFEETIATLHLKLENKNDFDLGLNALDYEVWLGDVSIGGAELTKSAKIEKSGISYIDIPIT
FRPKDFGSALWDMIRGRGTAYTIKGHIDVDTPFGAMKLPISKEGGTTRLKKKKEDRDYDD
DDDDEQE
>GmLEA2-10
MPTESGPNVVANEELGTPKQPRNQHSQQSGAPNSLANTRAIFGQPRLQRTKPIIWCAAIMC
FIFSLVLIFFGIATLILYLSMKPRNPTFDIPNASLNVVYFDSPQYLNGEFTLLANFSNPNRRIG
VRFESLNIELFYSDRLVSSQTIKPFTQRPRENRLQSVNLISSLVFLPQDVGVKLQRQVENNR
VNYNARGTFKVRFNVGLIHLSYSLYSTCQIEMTSPPAGILVARQCITNR
>GmLEA2-11
MKVGSGKGRKVCLTVTGVVIAIVLLIVILALTVFKAKHPVTTVDSTKLEDFHVSLDPVKLR
VDLNVTLGVDVSVKNPNKVGFQYSDSTAHLNYRGQLIGEVPISAGEISSGETKGFNLTLTI
MADRLLSNSQLLSDVTSGTLPLNTFVRMSGKVSILGFIKVHVVSSTSCDVAINLSNGTVGN
QECQYKTKL
>GmLEA2-12
MSQLNGAYYGPSIPPPKTSYHRPGRGGGLGCCCGCLFSLIFKLILTVIIIIGIAVFLFWLIVRP
NVVKFHVTEATLTQFNYTPNNTLHYDLALNITVRNPNKRLGIYYDRIEARAMFHDARFDS
QFPEPFYQGHKSTNVLNPVFKGQQLVPLNADQSAELKKENATGVYEIDVKMYLRVRFKL
GVFKTKTLKPKVSCDLRVPLKGSAGAGVFQTTKCDWDR
>GmLEA2-13
MADKQPHLNGAYYGPAIPPAEQPHYRPSRERSCCCCLFGILWKILVALIVLVGLAVLIFWLV
VQPRSFKFHVTKANLTQFDYYTNNNTLHYNMVLNFTARNPNKKLSIYYDKVEALAFYED
VRFANYSVITHMNSFRQYKKTTSHMSAVFSGQQVLPLDNDLVSELNQDKSGGVYEIDVKL
YFRIRFRLGDVKTRRFKPEVKCDIRVPLRTNGSVTLFQTTKCDVDY
>GmLEA2-14
MADYKQHHGRSCGCCCLFGILWKIVVALIVLVGLVFLIFWLVVQPRYFKFHVTEADLTQFE
YYPNNNTLHYNMVLNFTARNPNKKLSIYYDKVEALAFYEDARFANYDVITHMNSFRQYK
KSTSPMSAVFSGQKVLMLNNEQVSQFNQDKSVGAYDIYVKLNFRIRFRLGDSISRHLKPK
VKCDLKVPLNKSGTFTLFQTTKCHVNV
>GmLEA2-15
MADKHGRSCCCCLFGILWKILVALIVLVGLVFLIFWLVVQPRSFKFQVTEADLTQFDYYTN
NHTLHYNMVLNFTARNPNKKLSIYYDKVEALAFYEDARFANYDVITHMNSFRQYKKSTS
PMSAVFSGKKVLMLNSEQVSKLNQDKSDGVYDIYVKLNFRIRFRLGDSISGNLKPKVKCH
LKVPFSKSGTFTLFETTKSAVAASLESCGRFWLHSSSSLASYSSYSGWWFNPAPSSSRSRKR
T
>GmLEA2-16
MADKQPHLNGAYYGPAIPPAEQPRYRPHSHRGRSCCCCLFGILWKILVALIVLVGLAVLIFW
LVVQPRSFKFHVTEADLTQFDYYTNNNTLHYNMVLNFTARNPNKKLNIYYDKVEALAFY
EDVRFASYDVITHMNSFRQYKKSSSPMSAVFTGQQLLMLNNDQVSEFNQDRNTGVYDIY
VKLYFRMRFRLGDFISNDYKPKVKCHLKVPFSKNGTFTLFQTTKCDVDF
>GmLEA2-17

MILPYHFKTLKYQTSNSSLFSLHSSLLSSFPHKTSVRNISTLTMSVKECHHHKGKKHKLWR
RIFWGIVIFAFIVLLTVLIIWAILKPSKPTFILQDVTVYGFNATIPNFLTSSFQVTLSSRNPNDKI
GVYYDRLDTYVTYRNQQVTYRTSIPPSYQGHKEEDVWSPFVFGTNVPVAPFNFVGLSQD
QTNGNVLVLVKIDGKVRWKVGTFVSGHYNLYVRCPAFITFGPQSTGIALGKNAVKYQLVQ
RCTVGV
>GmLEA2-18
MSKDKVSGDPRRAVCTGITIFLLLAGVTLLVLWLVYRPHKPRFTVIGAAVYDLNTTTPPLM
STTVQFSVLIKNPNRRVSIYYDRFSAFVSYRNQAITPQVLLPPLHQEKRSSVSVSPVMGGTA
LPVSVEVSDGLAVDEAYGLVGLRLIFEGRVRWKAGAIKTAHYGLYVKCDVLMGLKKGLV
GQVPLLGVTPCHVHL
>GmLEA2-19
MDVSTQNPVFSSKKCCFFLLIICMVVGLATFFMILIIRPHKPVFSVREVKINFYKIDNDSSNL
TLLVSSVIYLTLNAENHNKFGIGFSSSRFLVYHEGLHIGTIRIPWFFQPPHSENVSVPSRVLL
HSANLTKIIANTSLQEISKQNMAQMRIIGDSRAHVWVLHIKLFEIMIFAFSCDCESHLS
>GmLEA2-20
MSDPASKPNGNAAAATNGNPGPVKSQLYNPNRQVYRPQSHYHRRGQRSHRNFCCCCCF
WTILTLLAVALLAAIVGAALYVLYRPHRPEFSVTNLRIAKMNLTTSADSPSHLTTLFNLTLIA
KNPNNHLVFFYDPFSMTVFSNSVPVGNGSVTAFTSDKNNQTSLRAVLSGSQDLDTDSLTSL
RSGLKMKRGFPVEIQMDTKVKMKMDWLKSKKVGIRVTCDGIRGTVPSGKSPAVASVVDS
ECKVDLRIKIWKFSF
>GmLEA2-21
MEERVLLPSPPPPPPPLEKKHSTNKLELPDFNPGTYVVQVPKDQVYRVPPPENARIAESHK
KAPPKAAKTSRCCLFCVLFFIIFFVLLILLGAVLGGLFSMLLTPKDPQFSITRFKVVETKPHP
KYDVTLEVHNLNSDVGVSYKNKGHVSLSLRRQEVASGAYPSFNQDAHDRTTFGVTLTSS
KVGLPKEVEESVTNDKKKVNVTFSLAIHALARMKMGLLRSGTMKFDVTCNVKLDTLAK
TTQVLSQQCETKRH
>GmLEA2-22
MSKITITSPKHCAGKEGLRIKKNYKKIYFTFSAFFTTILLLILLIWLILHPAKPQFSLKEVDIY
QLNLSGPNLNSSIQLTLLSKNPNQKVGIYYDEIQFYATYKGQQITGDTPVPPFYQGQEESNL
ITASLVGNALPVAPSLGYELGRDQIVGRLVLNLKANGKLRWKVGTWVSGRYRFNVNCVAI
NAFGPSIPAGPLTSKQGAQCSTTL
>GmLEA2-23
MKPNDNMTMLAKTDSEVSSLTQSSPARSPRRDVYYVQSPSRDSSHDGEKTTNSFHSSPLQ
SPLGSPPHSHSNSSLGRHSRESASTRFSGSRKSSSSGNNRKGPWRPWKDQFHAIEEEGLLD
PNDNAHHGFPRRCYFPAFVVGFVVLFSAFSLILWGASRPQKPAISLKSITFDQFVIQAGAD
MSGVATSLVSMNSSVKMTFRNTATFFGVHVTSTPVDLNYYQLTVATGTMPKFYQSRKSQR
SVRVMVIGSHIPLYGGGANLNSVNGKPVEPVPLTLSVMVRSRAYVLGKLVKPKFYKKIECS
IVMDPKKMGKAISLVKKCTYQ
>GmLEA2-24
MPMEGHADVSATFPPPPGHHGADTYIVQLPKDQVYRVPPRENALIVEQYRNPATAKKRRG
GCCCCCSRRVFLTVALIVVAIVAVVGITLATLYFIFSPSGPKFTVSHVAVNRNKSSRAAPQYE
VSLRARNPNEKLAIEYQDGDVSLLFDDTKVAEGKFPTLEQGRGEASEVTVELTGSSGALP
RRMNGGDAAVDLKLEMKLAIRIRTAGLETWAMRSDVACEFKVSALGNDTRVLSQQCDTK
FKQY

>GmLEA2-25
MAQSPDQQMKPLAPFISSTQFSRQEDQFQERTSEQKIIRIRKFVLCCGCFTALVVILVVILIV
LSFTVYNVKEPEVRMNSVTLLSGTFANGGATNNVTLVADIFVKNTNAFTLRFGSTSTIVYY
DGVRIGEGTSPPGKAKARRTIRVNSTLEIMSKKLLEIPTLNIDLRDQFLNISSYTRIDGKVKI
LNIFPRKVVVEMNCTIGYNITTGSVTNGDNCLGAVDI
>GmLEA2-26
MAAGVSAPPPAPQAPRPKRPRPPSGRTNLASCVVATIFLIFIIIVILIVYYTVFKPQDPKIAVN
AVQLPSFSVANGTVNFTFSQYASVRNPNRAAFTHYDSSLQLIYSGSQVGFMFIPAGEIDAGR
TQYMAATFSVQSFPLSVPPRMGPTLANGDGVGFNYGLRVEPTMEIESKLEMAGRVKVLH
FFTHHVYAKAGCRVAIAVTDGSVLGFHC
>GmLEA2-27
MHAKTDSEVTSLDASSSTRSPRRAVYYVQSPSHDGEKTTTSLHSTPVLSPMGSPPHSHSSS
SRFSASRHRNNHNNKSWKGIDVIEEEGLLQSELDRQHSLSRRYYFLAFLLGFFLLFSLFSLI
LWCASRPMKPKILIKSIKFDHLRVQAGSDSSGVATDMITMNSTVKFTYRNTGTFFGVHVTS
TPFDLSYSDIVIATGNLKKFYQSRKSQRLVSVAVMGNKIPLYGGGASLSSSTGVPTLPVPLN
LTFVIRSRAYVLGRLVKPKYYKRVQCSINLDPKKINVPISLKHSCTYD
>GmLEA2-28
MSDPASKPSGNGAAATNGNPGPVKSQLYNPNRQVYRPQSHYHRRGQRSHRNLCCCCCF
WTILTLLAVALLAAIVGAALYVLYRPHRPEFSVTNLRIAKMNLTTSADSPSHLTTLFNLTLIA
KNPNNHLVFFYDPFSVTVLSNSVPVGNGSVTAFTSDKNNQTSLRAVLSGSQDLDTDSLTSL
RSGLKMKRGFPVEIQMDTKVKMKMDWLKSKKVGIRVTCDGIRGTVPSGKTPAVASVVDS
ECKVDLRIKIWKFSF
>GmLEA2-29
MEERVSSSSLPSPSPPPLEKNHNTDKLKLPDLDPGTYVVQVPKDQVYRVPPPENAQIAESH
KKSPPNKEAKTSRCCLCCVLFFIIFLVLAILLGAVLGGLFSMLLTPEDPKFSITSFKVVETKP
HPKYDVTLEVHNPNSDVGILYNGKGHVSLSLRRQENIASGAYPTFRQDSHDTTTFGLTLTS
SSKAGLPKEVEESVRNDKKKVSVTFSLAIHALAHMKMGLLRSGTMKFDVTCKVKLDTLA
KTTHVLSQQCETKRH
>GmLEA2-30
MSKITITSPKHCADKEGLKIKNYKKIYFTFSAFFITILLLILVIWLILHPAKPQFSLKEVDIFQL
NLSGPNLNSSIQLTLLSKNPNQKVGIYYDEIQLYATYKGQQITGDTPVPPFYQGQEESNLIT
ASLVGNALPVAPSLGYELGRDQIVGRLVLNLKANGKLRWKVGTWVSGRYSILSRIDYQEL
LFHLCVYASIFGIHCYMHILKLPVEYFVLNHLSHEY
>GmLEA2-31
MSRHNINPIGSQFFLRTPALFFPHKQVCKGNNLYYPHYPKSEKMATRGLKICLAVSSLFLIIL
AIVIVTLILTIFKPKNPDIFLHPVDLENFQLLSPNTTSAPLGIVITIVNPNYGNFKYVNSSGYL
KYRDTIIAEVPLGIRSFPARSTTNVSTTVGIMTDKLIQDPKFLSDIEGGVFNLTAEATLPGKV
TMIKILRLKAKIYISCGVSFNIIAVDASSSCMSKIKL
>GmLEA2-32
MTDRVHPSAKTTANAGPKPTFPATKSQLSGANRPTYRPQPQHHRRRRSRGCASTLCCWLL
LILLFLLLLVGAAGTVLYFLYRPQRPTFSVTSLKLSSFNLTTPSTINAKFDLTLSTTNPNDKII
FSYDPTSVSLLYGDTAVASTTIPSFLHRQRNTTVLQAYVTSTEEVVDSDAAMELKRSMKRK
SQLVALKVELETKVEAQMGVFQTPRVGIKVLCDGVAVSLPDDEKPATASAENTACQVDVR
FKVWKWTVG

>GmLEA2-33
MQIQHQEDHNHNPNHILRPNPNPNPNPTQYTHYRFPGKVPHHQHQVHNMDKGAASRFK
PNAPKREHCICITVFLLLLGIILLVLWLAYHPTKPRFTVASAAVYGLNATSPPLMSIAMQFN
MVIRNPNRRVSISFDRLSAYVSYRNQPVTPHVMLPPLFIEKHSAVSLSPEIGGVPVPVSEDV
SNGLAMDENYGVVGVKLVLFGRLRWRAGDINSAHYGLYVKCDVLMGLRKGFVGQVPL
LGAPVCDVNT
>GmLEA2-34
MAHAKTDSDVTSMDTSSSPKRAVYYVQSPSRDSHDGDKSSTATHATPACNSPVDSPSHHS
YGHHSRASSSSRVSGGSYNIASWGRKVTRKNKLGWPHECKVIEEEEEGYYGERQGFSRK
TQIFVGVLGFAFIFTLFCFIIAAAARPYNVRISVKSFTVHNFLFGEGSDMTGVPTKMLTVNC
SARMTVHNPATFFGIHVSSKAVNLMYSEMTVATGELKKHYLSRKSTRTVSVNLQGSKVSL
YGADASLTGLVDNGKIPMTLVFEVGSLGNIVGRLVRSKHRRRVSCSVAIDSHNIEPIKLKEN
ACTYN
>GmLEA2-35
MTGKDEFIRLTRIRLNRIGIRTNPRPILDLSFSITVKVHNRDFFSLTYDTLAVSVGYRGRQLG
FVTGGGGGRIRARGSSYVDATLTIDGFEVIYDAFYLLEDIAKGVIPFDTDTRVEGKLGLFFF
TVPLKATVSCEVYVNINQQTIVRQDCYPKIFCSLTIMQ
>GmLEA2-36
MHAKTDSEVTSLAASSPTRSPPRRPLYYVQSPSRDSHDGEKTATTSFHSTPVLSPSASPPHS
RHSSSTRFSKKDHSHSLKPWKQIDVIEEEGLLQGDDHHNGLPRRCYFLAFVVGFLVLFSFF
SLILWGASRPMKPKINIKSIKFDHVRVQAGSDATGVATDMITLNSTLKFAYRNTGTFFGVH
VTSTPVELSYSDIVIASGNMKKFYQSRRSQRLVSVAVMGNKIPLYGSGASLSSTTGVPTVP
VLLNLNFVLRSRAYVLGKLVKPKYYKTIQCSITLDPKK
LNAAISLKKSCTYD
>GmLEA2-37
MCCRNRFIACTCCIIITLFFMFIISIIVCISPSSVKFHVTDASLTQFNLTSNNTLYYNLKVNVT
VRNPNKHTIVYYRRITVISWYKDNAFGWVSLTPFDQGHKNTTFLQAVFEGQRVIKLKSKQ
LGEYKDETSVGIYKDLAVDFDLRIRAKYGKFKSSRFNTPIVQCRRLRVPLISNGKSVPPFSV
TRCKSASFFSDRDAPDVDG
>GmLEA2-38
MCCRNRFIACTCCIIITLFFMFIISIILFWIIISPSNVKFHVTDASLTEFNLTSNNTLYYNLKVN
VTVRNPNNKIVVYYRRIKAIAWYKDNDFGWVSLTPFDQGHKNTTFLQAVFEGQRVIKLKP
KQLGEYKDETSVGIYEDLAVDFDLRIKANFGRFKSSRFDPPVVQCRRLKVPLISNGKTAPP
FSVTKCKIDGSFFADRDSQAEAR
>GmLEA2-39
MCFRSRCSFCYIFFTIYTLMLMFILSIILFWIIISPSSVKFHVTDASLTQFNLTSNNTLYYNFK
VNVTMRNPNNNIIVYYRRITAISWYKDNAFGWVSLTPFDQGHKNTTFLQAVFEGQRVIKL
KSKQLGEYKDETSVGIYKDLAVDFDLRIRAKYAKFKSSRFNTPIVQCRRLRVPLISNGKSVP
PFSVTRCKSAYFFSDRDAADG
>GmLEA2-40
MAQSPDQQMKPLAPFVSSTSHFSRQEDHDQFQDTSEQKIIRIRKFVLCCGCFTALVVILVV
VLIVLGFTVYNVKEPEVSMNSVTLVSGTFANSGATNNVTLLADISVKNTNAFTLRFGKTTT
IVYYGGMGIGEGTSPPGKAKARRTIRVNSTLEIMAKKLLDIPTLNIDLRDQSLNISSYTRIDG
KVKILNMFPRKVVVELNCTIGYNITTGLVTNGDNCLGTVDI

>GmLEA2-41
MAAGAAPVPAPRPKRPRPPSGRTNLASCVVATIFLIFIVIVILIVYYTIFKPQDPKIAVNAVQL
PSFSVANGTVNFTFSQYASVRNPNRAAFSHYDSSLQLIYSGSQVGFMFIPAGEIDAGRTQY
MAATFSVQSFPLSAPPRMGPTLANGDGVGFNYGLRVEPTLEIESKLEMAGRVKVLHFFTH
HVYAKAGCRVAIAVTDGSVLGFHC
>GmLEA2-42
MNTRAGANSESQPQPSRRQQHLHYASSPTTSSHSSCCCCLFLLFSFFALLVLAALLVIVLAI
KPKSPHLDLRQVGLQYMALVPNPTRPGPSTAKLYLVIRLVLAVVNPNEVGIRCGESRVTVV
YRDTPLGRTSLPAFCQRAHTVKEVVATMAVDDVNLSNADGADFARDALLNDRVELRVLA
HVATKIRLFNLPSPPLQVSVNCVIVISPRKQSLTYKQCGFEGLN
>GmLEA2-43
MEGHADHNSAVLPPPPGRHAPAAGTYIVQFPKDQVYRVPPRENALIVEQYRNPATAKKRR
GGCCCCCNRRVLITFALVVTTIVAVVGITLATLYFIFSPAGPKFTVSHVAVNRNNKNSQGAA
AQYEVSLRARNPNEKLAIQYQEGDVSLLLFDESKVAEGKFPTLEQGGGEASEVKLELTGS
SGAFPRGMHGGDAAVDLKLEIKLAIRIRTAGLETWGMSSNVACQFKVSGLGNDTRILSQQ
CDTKFKQY
>GmLEA2-44
MTTPRGNHHPQPNGERRRQWPPPQPPAAAPEAAAAPTAVAANYNGYRQYHPTTPARSSSS
ASFKGCCCCLFLLFSFLALLVLAVVLVIILAVKPKKPQFDLEQVGVQYMGITPNSPSTASLSL
TIRLLFAATNPNKVGIRYGQSSFTVMYRGIPLGKATVPGFFQQPHSTRQVIATIAVDRVNLL
QADAADLIRDASLNDRVDLRVLGDVAAKIRVINFDSPAVQVSVDCAIVISPRKQSLTYKQC
GFDGLTV
>GmLEA2-45
MKKGFGCKGSIMCLVVALCVAIALVLLGVILALTVFKPRHPITNVDSVRLQNMSLAMDIFS
MSVNVNLTLEVDVSVNNPNKLGFNYYNSFAQLNYKGQLIGEAPIPNGDILAEEIKGLNLTL
IVMADRLVSNSNVTKDVALGSLPLNTLVRIFCQVNILGFMKFYVASTSSPKIPHFLDFKNLY
KGAKLSPFTSI
>GmLEA2-46
MHAKTDSEVTSLAASSPTRSPPRRPLYYVQSPSRDSHDGEKTATTSFHSTPVLSPSASPPHS
RHSSSTRFSKKDHSHSLKPWKQIDVIEEEGLLQGDDRRNGLPRRCYFLAFVVGFLVLFSFF
SLILWGASRPMKPKITIRSITFDHVRVQAGSDATGVATDMITLNSTLKFTYRNTGTFFGVHV
TSTPVELSYSDIVIAAGNLKKFYQSRRSQRLLSVSVMGNKIPLYGSGASLSSTTGVPTLPVP
LNLSFVLRSRAYVLGKLVKPKYYKTIKCSITLDPKKLNAAISLKKSCTYD
>GmLEA2-47
MIGKDEFVSYSPLPSNPNPNPNPNPNPNPNPYPNPNPYYDPNPHPYPQNVVVLLPSYRPHF
QRRRRRCIIYSAALFLFLLVAGAAFLLYPSDPEIRLARIRLDRIGIRTNPRPILDLSFSLTVKVR
NRDFFSLSYDSLTVSVGYRGRQLGFVTAGGGSIRARGSSYVDATLTIDGFEVIYDAFYLLE
DIAKGVIPFDTDTRVEGKLGLFFFTVPLKATVSCEVDVNINQQTIVRQDCYPKSLGDPLDES
AYIEAGDT
>GmLEA2-48
MAHAKTDSDVTSMDTSSSPKRAVYYVQSPSRDSHDGDKSSTATHATPACNSPVDSPSHHS
YVHHSRASSSSRVSAGSYNNIASYWGRNNKGTRKNKLGSWTHDQCKVIQEEEGYYGERE
GFSRRKTQIFVGILAFAFIFTLFCFIIAGVARPHKVRISVKSFTVHNFLFGEGLDLTGVPTKM
LTVNCSVRMTVHNPATFFGIHVSSKAVNLMYSEMTVATGELNKHYLPRKSTRIVSLNLQGS

KVSLYGAGASLIGLVDNGKIPMTLVFDVRSRGNIVGKLVMSKHRRRVSCSVAIDSHNIKPIK
LKENACTYN
>GmLEA2-49
MPSHDHVAERQHHRPIRRRLVNTHHSGKTHPLIWLAAILCTIIAIGVVIAGIVVFVGYMVIH
PRIPVMSITNAHLDLLSNDYAGLLQTQLTIIVVAQNGNAKAHATFSDIRFNLSYQGQRIALM
RAPPFDVPKNSSKSLNYVVRAASIPLTPQQMEEVDESWKRDVIGFDLKGSARTRWRVGPL
GSVKFWCNLECQLKFHPSNGSYIHHSRCTSKSK
>GmLEA2-50
MSHLNGAYYGPAVPPPKSYHRPSHGGGGRDGCCCGCLSCFCGCIFDCILGLICKILTTIIIILA
VLAFLFWFIVRPNVIKFHVTDASLTRFDYTTNNTLHYDLTLNVSIRNPNRRVGVYYDQIEA
LALYEDVLFGNLTLGSFYQHHKNTTFVSPIFKGQRVTPLAKVQVSEFDKEKGSGVYTIDLK
LFMTVRFKFLLFKSGSLKPKIRCALHVPLKSRNATTSPDAAFQPTECDWDYGKKWWIH
>GmLEA2-51
MADKQSQLNGAYYGPAIPPSELPRHHHRGRRCCCCLFSFFWKLLLAIVVFLVVVFLVFWA
VVQPRTFKLHVTDAKLTQFNLTTNNNNNINMLRYNLVLNLTARNPNKKLNIYYDQVEGH
VSYEGMRFASADLITWRNSFRQYTKSTNNMSGVFTGQHGLVFDGDHVKDFEKDERGGV
FDIDVKLYFNIRFRLGDFIGSSSRVRAKCELQVPLVSRGVFLPTRCHVKF
>GmLEA2-52
MSVKECEHHKGKKRKIFRQVFWCLVVFLFIVLVTILLIWAILRPTKPTFTLQDVTVYAFNAT
VANFLTSNFQVTLISRNPNDRIGVYYDRLETFVTYRSQQVTYRTAIPPTYQGHKEINVWSPF
VYGTNIPVAPFNFLGLSQDQSNGNVLVTIRAEGRVRWKVGTFISGRYHLYVRCPAFISFGPR
SNGIVVGENAIKFQIIQRCSVSV
>GmLEA2-53
MADHQRLRIHPMEGEAPPPPTTPLVPPGSSRSEKGVPLHHPPPLPRAMPAAYPTPHKRSSSC
CCKCICWTIILVVLLLIIFAASVGILYLVFQPKLPDYSVDTLRISDLRLNFDMSLYAKFDVKIT
ATNPNKKIGIYYEKGGKLSVWYTNTRLCEGSLPQFYQGHENKTVLNVSLSGQVQSGSTL
MNALQQQQQTGRIPLDLKVHAPIAIKLGRLKLMKVRVLGECLLVVDSLSSNNLISIKASNC
KFRLKL
>GmLEA2-54
MHNNDHIPVHHVQGSNPKPVKLNRHHTMRYYVHRVHESLTTRVSKMICATFLGLLFIVGL
ITFILWLSLRPHRPRFHIQEFNLPGLTQNSGFENAVITFKVSARNSNQNIGVYYESMDGAVY
YRDQKIGSKPLLYPFYQQPKNTTEVDGDLSGATLTVSSQRWSEFQSDRADGSVVFRLELTS
VIRFKISTWDSKRHTMHANCNVGVGPDGSLLTLYKDKRCPVYFS
>GmLEA2-55
MCEGKSLYIWPLKVIGLLGLIVLCLWLALRPKNPSYSIMFISIQHPSNSSENCTIFYSLQIENP
NKDSSIYYDKTILSFLYGEPEDEVGETTIVPFHQGTGNTRDVSDTVNAKPRPFKPLFSAISN
ATTELKVALITRYRYKTWGIKSKFHGLQLKGILPIDSDGKLSRKKKKYPLSRNSNKLGRFKI
RH
>GmLEA2-56
MTTEEVITMFPSRKEEKQSSSKCLVYALVVLVAILFIWLVFASIVLRVVDPQIQLKSARLMH
NTKNHSFSSTSSLNVTMIARVSLTNPNLFGRFYYGNSRVSVLYGASIVGAWELEGARLEGR
ETKEIDFMVHMRFSTKLLVIMRNLTNDTAHSDSAGMLKLRSYAKLSGTVHVLNMVNKKK
TLGMACIMNLNLTSYSTQHFQC
>GmLEA2-57

MAKSIEQEGRSGKCFVYFLGAFVILCALVLVFASILRLKNPYLKLRSATFNQIRYSASPSPSF
NATLIIFLALNNPTFGAFTYENNSLSVLYAGLKIAHSQINGGRVSFRQTKEIHVTVKFMSAID
ITSGSLNLTTNVFFSGKVHLFKIINIRKTIEMPCSMNLNFTSHATQAIQCQ
>GmLEA2-58
MGGRMHTKSDSEVTSNSMEQSSPSRSPPRRPLYYVQSPSNHDVEKMSYGSSPMGSPHHHF
HYYLSSPIHHSRESSTSRFSASLKNPRSNFSSSSSWKKLHPHPNPDAGLDDDDDDDDGDD
DLNHFPRNLRLYFCFFLLFLLFFTLFSLILWGTSKSYKPRIIVKSIVFENLNVQSGNDGTGVP
TDMLSLNSTVRILYRNPATFFGVHVTSTPLHLSYYQLAIASGQMQKFYQSRKSQRKLAVV
VLGHQIPLYGGVSVLGNTKEHLENVALPLKLTFVVRSRAFILGRLVKSKFYRRITCSVTLH
GNKLGKHLNLTDSCVYK
>GmLEA2-59
MSVKECGHHSSQRRNLLRLILGATAAFVLLILLTIFLIWVILRPTKPRFTLQDATLYAFNLST
PTPNTLTLTMQVTLSSHNPNARVGVYYHALHVYASYRSQQISLATALPDTYQGHRDFAVW
SPFLFGNVVPVSPFVLSSLQQDQSAAGAVLVNVKVNGRVKWKVGSWVSGRYHIYVNCPA
YISFAGDRSIAAGGLVASPVKFRLLQSCSVDV
>GmLEA2-60
MDNKKSKWSWSSAIIGAASAVAASALISAKPKDPTFHLISINFTSLKLNFPLLDAEVLLTVH
VTNPNIAPIHYSSTSMSIFYQGSLLGSAQVQAGSQPPRSCQLLRLPARLHALELAHHATRFL
HDVARREMFLDAAVDIAGTARVMWWDHNFKVHVDSHVTVDPVFLDVIDQENTSELEVF
TAAALGSEQ
>GmLEA2-61
MEEGKSKSTLAKNPSRSRNPSRRVALDVPSPRHMGSTLSGIHSSIDHLDKGRYHPCCFACC
AWSCLIVFILIIAILFLGITYLAFLKSGMPKINVRAFNITKFQVDDGSQKMNSVIGLGLIFSN
KNDKLKLLYGPLDVDVTSEDVLLGKKKQGGFSQKPLNVTNLDMTMTLENADVDKYAAE
ELKSDIKAYEMVFDLYVGGHIGFQVGKLQMNNVPFLASCNQIKREDVDFGRKPECEVKLF
AARPSTN
>GmLEA2-62
MMAAEHQRIHPVHDVEAQHRPLVPQNIAKSDDKGIPHRTFPVMHSKPPKRRRSCCCRFM
CWTLSILLILIIAIAITIGILYLVFRPKLPKYSVDQLRISQFNVSDNNTLYATFNVAITARNPNK
KIGIYYEGGSHISAWYMETQLCEGSLPKFYQGHRNTTVLDLPLTGQAHDANGLVNRIQEQ
LQQTNNVPLNLKVNQPVRVKLGKLKLFKVKFRVRCKLEVDNILGASNDIRISRSSCKFRLR
L
>GmLEA2-63
MGGRMHTKSDSEVTSNSMEQSSPARSPPRRPLYYVQSPSNHDVEKMSYGSSPMGSPHHH
FHYYLSSPIHHSRESSTSRFSASLKNPRNFSSSSSWKKLHPHPNRDVDPDDHDDADHHLKQ
SSRNLRLYLWFFLLFLLLLTLFSFILWAASKTYKPRIIVKVTCPNGIVIYCSLCDLIFGFCYGG
GQSIVLENLYVQSGNDGTGVPTDMLSLNSTVRILYRNPATFFGVHVTSTPLLISYYQLAIAS
GQMQKFYQSRKSRRSLAVVVSGHQIPLYGGVSVLGNTKEHLESVALPLNLTFVVRSRAFIL
GRLVKSKFFRRIRCSVTLHGNKLGKHLNLTDSCVYK
>GmLEA2-64
MSVKECGHHSSQRRNLLRLILGAIAAFVVLILLVIFLIWVILRPTKPHFTLQDATLYAFNLST
PTPNTLTLTMQVTLSSHNPNARIGVYYHALRVYASYRSQQISLATALPDTYQGHRDFAVWS
PFLFGNVVPVSPFVLTSLQQDQSAAGAVVVNVKVNGRVKWKVGSWVSGRYHIYVNCPAY
ISFAGDRSNVAGVVASPVKFRLLQSCSVDV

>GmLEA2-65
MSTKECCHHDEERRQLPRRVFAAILGFILLILLVIFLIWIILRPTKPRFILQDATVYAFNLSSG
DTPSPITPTPNTLTLTMQVTLAAFNPNHRIGVYYTKLDAYAAYRGQQVSVATSLPATYQGH
RDTSVWSPYLYATAVPVSPFTLQILQQDKTSGGILVNVKVNGRVKWKVGTWVSGIYHINV
NCPAYLRLAGDRDDAIGFAGPAVKFQLFQTCIVDV
>GmLEA2-66
MANRGLKICLAVSLLFLVIVTIMIVTLFMTIFKPKNPEITVHPVGLEDFQFSLSPNLTINVTL
GMIITIRNPNYGSFEYKNSTGYVNFHDTVVAEVPIEAELVPARGQINVNTSADFMVEKLIN
DPNFLSDVLGGTLNFTSTTALPGKARMFNIIKLKATSYSSCDISVNISSRKVDTNCNYKIKL
>GmLEA2-67
MTSEAYVPKRYESLENPFAHKPRKRSHQGSGLCGCLRKENKVYSDVVIVVKADNQNEDI
GLDYLDNEVGIMYLGSQLSSGQIPPFLQPGKNTTKVNVELKGENEFGEKGKIPLLITVKLPI
RLVIKDLIHLRKVVVNVNCSVVIDKLEANKSPKILDKVFTYGIDF
>GmLEA2-68
MRPTPTPDNLNCIPRIKVGSGKGRKVCLTVTGVVIAIVLLIVILALTVFKAKHPVTIVDSTK
LEDFHVSLDPVKLRVDLNVTLGVDVSVKNPNKVGFQYSDSAAHLNYRGQLIGEVPISAGE
ISSGETKGFNLTHTIMADRLLSNSQLLSDVTSGTLPLSTFVRMSGKVSILGFIKVHVVSSTS
CDVAINLSNGTVGNQECQYKTKL
>GmLEA2-69
MAHPPTQSQSRAANKPKRSNLLRCIAIFILALIILVGIAVIIIWLVLKPKRLEYTVENAAIHNF
NLTDANHLYANFDFTIRSYNPNSRVSIYYDTVEVSVRYEDQTLATNAVQPFFQSHKNVTRL
HVGLTAQTVALYDSVPKDLRLERSSGDIELDVWMRARIRFKVGVWKSKHRVLKIFCSPVL
VHFSKGKSFERAPCDVEL
>GmLEA2-70
MTSEAYVPKRYESLENPFAHKPPRKRSRRGSGFCGCLRCCCCCFSFCRCCICTIFIIIILLVGI
GLALFYFIKPKVPSYDIEHINVKSFDIRKESKVYTDVVIVVKADNPNEEIGLDYLENEVGIM
YVGSQLSSGQIPPFLQPGKNTTTVTVELKGENEFSVEMQNHFMEDQEKGNIPLLITVKLPI
RIVINDLIHLRKVVVNLNCSVVIDKLEVNKSPKILDKVFSYGIEF
>GmLEA2-71
MANKGLKFCLIVSLLFLIIVTIVIVTLFFTVFKPKDPNITVHPIGLEHFDFSLLPNITANVSLG
MVITIENPNYGSFEFTNSIGYINFHDTVVGEVPIGAELVPAHGQINVNTWANFMVAKLISVP
KFWSDVLSGTLNFTSTSSLPGIARMFKIFKLKATAYSSCNISLRIVPRNVDTKCISKIKL
>GmLEA2-72
MASRGLKICLAVSLLLLIIFTIAIVTLFMTIFKPKNPEITVHPVGLEDFQSSFSPNLTLNVTLG
MIITIRNPNYGSFQYINSTSYVKFHDTVVAEVPIEAELVPARSQINVNTSADFMVAKLINDP
NFLSDVLGGTLNFTSTIALPGKARMLNIIKLKATSYSTCDISVNISSRNVDTNCKSKIKL
>GmLEA2-73
MINPTKAMPVSHSFLSLADSTDTHRFTIMQDPSRPAPPPFYHQNGGQPPPPAASAAIATGYP
YAAQQQYYNTTYPPPRSYASRSFFRAFFATMICLAVVFGVVLIITWLVLRPSLPHFTLHSLS
VSNLSSTSQSLSATWHLSFLVRNGNKKMTVSYNALRSSIFYRQNYISESQLAPFRQDTRSQ
TTLNATLTAAGTYLEPKLIDNLNAERNASSVLFDVQVVAATSFRSGSWRFRTRVLKVLCRK
VPVGVSSKSSSGDLVGGDRECQVWT
>GmLEA2-74
MSTKDCGNHDEKNRQFLRCLFAAILGSILLLIFLIWIILRPTKPLFILQDATVYAFNLSSSGPT

PSPINPTPNTLTLTLQVTLASFNPNHRIGVIYTKLDTYSAYRGQQLSIATSLPATYQGHRETA
VWSPYLYASAVPVSSFTLQILQQDRTSGGILVNVKVSGRVKWKVGTWVSGNYHINVNCPA
YLRVASDRDDAVGFAGPAIKFQLSQSCIVDV
>GmLEA2-75
MTDRVYPSAKPAAVNGAAANPSFPATKAQLYGATRPTYRPQPHHRRRSKRRCCCTFFFWL
ILTVLILLLLIGVGGTVFYLLYRPHHPTFTVTSLKLSYLNLTSSSNTLNSRFDITVSATNPNKK
ILFAYDPTSITILSADIDLGDGTVPGFQHPKKNTTLIKGSILSSGQALQSDEASRLKSSMKSK
NGLPLKVNLETKVKAKMGNLKTPKVGIRVSCDGIRVSLPSGKKPATASTSNAKCDVDVRF
KIWKWTV
>GmLEA2-76
MTLSAKSESDITSLAPSSPSRSPKRPVYYVQSPSRDSHDGDKSSSMQATPISNSPMESPSHPS
FGRHSRNSSASRFSGIFRSSSGRKGSRKRNDKGWPECDVILEEGSYHEFQDKGFMKALPG
LDYLCGCLRCVLFDHLGPYKAQIAVKSLTVHNFYVGEGSDFTSVPTKMLTVNGTLRMSIY
NPATLFGIHVHSTPINLVFSDITVATGELKKHYQPRKSHRIISVNLEGTKVPLYGAGSTITVS
QTGVEVGLTLNFEIRSHGNVVGKLVKTRHRKEITCPL
VLNSSRSKPYQIQKEFMHL
>GmLEA2-77
MRFSKASFYTLLLRKPHNHHHQNYVVLSRRRVVTLAVGVLILLSALYIFWPSGPDLKIVGL
KLRRIKVHPVPPITIDISMLLTLRVHNVDVYFMDFGAVNVAVAYRGKMLGHVTSRRMHVR
ARGSSYVDADVEFAGISVLPELVLLLEDVARGIVPFDTISHAKGQLGLLFFHFPIKAKLSCE
VVVSIINQTIVRQHCLHE
>GmLEA2-78
MLASVEEELGTPKQPRNQHSQQSEAPNSLANTRAIFGQPRLQRTKPIIWCAAILCFIFSLVLI
FFGIATLILYLSMKPRNPTFDIPNASLNVVYFDSPQYLNGEFTLLANFSNPNRRIGLRFESLN
IELFYSDRLVSSQTIKPFTQRPRETRLQSVNLISSLVFLPQDVGVKLQRQVENNRVNYNARG
TFKVRFNIGLVHLSYSLYSTCQIEMTSPPAGILVARQCITNR
>GmLEA2-79
MSTSDKPEVVERGSKDEKHKEDDQEEGKGGFIEKVKDFIHDIGEKIEEAIGFGKPTADVTA
IHIPSINLHKADLVVDVLIKNPNPVPIPLIDIDYLVDSDERKLVSGLIPDAGTIGAHGEQTVKI
PVTLIYDDIKQTYADIKPGSIIPYRVKVSLIFDVPILGRLTLPLEKTGEIPIPYKPDIDLEKIHFE
RFSFEETIATLHLKLENKNDFDLGLNALDYEVWLGDVSIGGAELTKSAKIEKSGISYIDIPIT
FRPKDFGSALWDMIRGRGTGYTMKGHIDVDTPFGAMKLPISKEGGTTRLKKKKEDRDYD
DDDDDED
>GmLEA2-80
MADRVHPSHSPSVSADSQPPSPQDSSVVPKPPSPPSPEKPVPPPGTYVIKIPKDQVYRVPPPE
NARRYDQYARRKHRRSRCCCCFCWLIGILFILVVLLAIAAGVLYLVFRPEAPKYSIENITVR
GINLTSPSSVAAISPEFNVTVKADNPNDKIGIRYLKDSSAEVFYKDARLCNGALPAFYQPSN
NVTVFGTALRGDGIELRSEDRRALLEAQTKRRVPLTVRIRAPVKIKVGSIRTWKITVKVNC
DVTVNELTAQAKIVSKRCSYDVDLW
>GmLEA2-81
MVALCVAIALVLLRVILALTVFKPRHPITNVDSIRLQNMSLGMDMFSMSVNVNFTLEVDVL
VNNPNKLGFNYYNSSAQLNYRTQLIGEAPIPNGDILVEEIKGLNLTLTVMADRLVSNSKVT
KDVALGSLPLNTLVRIFCQVNVLGFMKFYVASTSYQKFPISSSSRICTKEQSCPHSLQYSKRI
TK

>GmLEA2-82
MADPQKIHPVHHDVEAQNHPSAPLVPRSMSKSDAGDPQRVVVVQQQQQQQQHIPVKH
TKPPTKKRRSCCCRFFCWLISILLILIVAIGITIGILYLVFRPKLPKYSVDELKVTNFDLADNN
SLSVTFNLTITARNPNKKIGIDYRGGSHISAWYMDTKLCEGSLPKFYQGHRNTTILSIPLTG
KTQDATGLQNTLQNQLQETGNVPLNLRVKQPVRIKLGKLKLFKIKFRVRCRIVVDSLSAN
SSIRIQSSSCKFRFRL
>GmLEA2-83
MMLSAKSESDITSLAPSSPSRSPKRPVYYVQSPSRDSHDGDKSSSMQATPISNSPMESPSHP
SFGRHSRNSSASRFSGIFRSSSGRKGSRKRNDKGWPECDVILEEGSYHEFQDKGFTRRFQA
LIAVLTFVVVFTVFCLIIWGASRPYKAEIAVKSLTVHNFYVGEGSDFTGVLTKMLTVNGTLR
MSIYNPATFFGIHVHSTPINLVFSEITVATGELKKHYQPRKSHRIVSVNLEGTKVPLYGAGST
ITVSQTGVEVPLTLNFEIRSRGNVVGKLVKTRHRKEITCPLVLNSSRSKPIKFKKNSCTYD
>GmLEA2-84
MTDRVYPSAKPAVVNGGAANPSFPATKAQLYGATRPTYRPQPHHRRRSRRRCCCTFFFWL
ILTVLILLLLLIGVAGTVFYLLYRPHHPTFTVTSLKLSYLNLTSSSNTLNSRFDITVSATNPNKK
ILFAYDPTSITILSGDIDVGDGTVPGFQHPKKNTTLIKASILSSGHALQSDEASRLKSSMKSK
NGLPLKVNLETKVKAKMGNLKTPKVGIRVSCDGIRVTLPSGKKPATASTSNAKCDVDVRF
KIWKWTI
>GmLEA2-85
MTTPRGNHHPQPNGERRRQWPPAHPAAAAAAAPEAAETATATAAGGHSYNGYRQYHPRT
PGRSSSSSASFKGCCCCLFLLFSFLALLVLAVVLVIILAVKPKKPQFDLEQVGVQYMGITPN
PPSTASLSLTIRLLFAATNPNKVGIRYGQSSFTVMYRGIPLGKATVPGFFQQPHSTRQVIATIA
VDRVNLLQADAADLIRDASLSDRVDLRVLGDVAAKIRVINFDSPGVQVSVDCAIVISPRKQ
SLTYKQCGFDGLTV
>GmLEA2-86
MAHPPSQSNSTAANKPKRSNLLHYIAMFIVALIILVGIAVIIIWLVLKPKRLEYSVENAAIHN
FNLTDANHLYANFDFTIRSYNPNSRISIYYDTVEVSVRYEDQTLATNAVQPFFQSHKNVTRL
HVALTAQSVALYESVPKDLRLERSSGDIELDVWVRARIRFKVGAWKSRHRVLRIFCSPVLV
HFSKGKSFERAPCEVEL
>GmLEA2-87
MLETEQARPLAPSIERQSSDEDNTTPHPQTQGHKKLIKRCACPLISLLLIAIVIIVLIFTVFRV
KDPVITMNSIKITKLQLVNTMSQQPGANMSLVADVSVKNPNVASFRYSNTTTSLYYHGVIV
GEARGPPGRAKARRTLRMNVTIDVITARVISSPDFVTDLGSGLLTMSSFSRVPGQVKILNLI
KRHVVVKMNCTTTFNISTQAIKEQSCKRKVKL
>GmLEA2-88
MSQLLDKAKNFVSEKVNDMAKPEASVTDVDFKRVSKDNVEYLAKVSVRNPYSTSIPICEI
NYSFKSASREIASGKIPDPGSLKAKDTTMVDVPVKVPYSILMSLAKDIGADWDIDYQLDL
GLVIDVPVIGIFTIPLSQKGEIKLPTLSTMFA
>GmLEA2-89
MKVGSGKGRKVCLSLTGVVIAIVLLIVILALTVFKAKHPVTTVDSTKLEDFHMGLDTPKLR
VDLNVTLHVDVSVKNPNKVGFKYSDSTAHLNYRGQLIGEVPIPAGEISSGETKGFNLTLTI
MADRLLSNSQLLSDVASGTLPLNTFVMMSGKVSILGFIKVHVVSSTSCNVPINLSNGTVGN
QECQYKTKL
>GmLEA2-90

MKPNDNMAMLAKTDSEVSSLTQSSPTRSPRRAVYYVQSPSRDSSHDGEKTTNSFHSSPLQ
SPLGSPPHSHSNSSLGHHSRESASTRFSGSRKSSSSGNNRKGPWRPWKDQFHAIEEEGLID
AHDNARGFPRCCYFPAFVIGFVLLFSAFSLILWGASRPQKPAISLKSITFDQFVIQAGADMS
GVATSLVSMNSSVKMTFRNTATFFGVHVTSTPVDLNYYQLTLATGTMPKFYQSRKSQRSV
RVMVIGSHIPLYGGGANLNSVNGKPVEPVPLTLSVMVRSRAYVLGKLVKPKFYKKIECSIV
MDPKKMGKAISLVKKCTYQ
>GmLEA2-91
MMAADQQRIHPVHDVEAPHRPLVPENYAKSDKGIPQRTFPVMHSKPPKRRRSCCCRFMC
WTLSILLILIIAIAITIGILYLVFRPKLPKYSVDQLRISQFNVSDNNTLYATFNVAITARNPNKKI
GIYYEGGSHISAWYMDTKLCEGSLPKFYQGHRNTTVLDLPLTGQAPDASGLVNRIQEQLQ
QTNNVPLNLKVNQPVRVKFGKLKLFKVKFRVRCRLEVDNFGASNDIRISSSSCKFKLRL
>GmLEA2-92
MLTLPPPPPPRLLTTRQTKQVSPDQIVISKLPIKQHSQESDAPNYVTTKSIRPPPPPPPPPPILR
QPPFQRTNPIIWFAAVLCLIFSLLLIFFGVVTLIIFLGIKPRNPYFDIPNANLNAVYFDSPEYFN
GDFTLVANITNPNKKIDVRFESFDVELFFSDRIISTQSIEPFTQRRRESRLESLHFISSLVFLPK
DLGVNLKGQVQGNRVKYNVRGTFKVRVSMGFFHLSYWLHSRCQIEMTGPPTGVLVARK
CITKR
>GmLEA2-93
MSQLAPKQFMTESQSNQDHEQVVVISQKKLKRRRVCVMVTGAVLLLLIVLVIVAIILAFTL
FKTKEPRTQLVSATLEGIAPRLTLPAIDLQINVTLDLKVRVENRNRASLKHEGGKSVLLYKG
KEVGDAYINPGLIPSRGSTILPCRLTLQVEKLASNLTSLVGDLMGGEISMDTVTRIPGKVTF
LGFIKKHIVAESNCQFTISVSELKITNQTCKSKAKL
>GmLEA2-94
MKKGSGCKGSIVCLMVTLCVVIALVLLGVILAMTVFKPRHPITNVDSVRLQNMSLDMDIF
SMSVNVNLTLEVDVSVNNPNKLGFNYYNSYAQLNYRGQLIGEAPIPNGHILAEEIKGLNST
LTVMADRLVSNSEVTKDVALGLLPLNSLVRIFGQVNVLGFIKFYVASTSSCDFTLNLSNRTI
VDNKCQEKTKISG
>GmLEA2-95
MRVGLGVKVSLGVIGVSLIFAVLKMSQLLDKAKNYVAEKVTNMPKPEASVTDVDFKRVS
RDSVEYLAKVSVSNPYSTPIPICEIKYSLKSAGKEIASGTIPDPGSLKASDTTMLDVPVKVP
HSILLSLAKDIGADWDIDYQLDLGLVIDLPVIGNFTIPLSQKGEIKLPTLSDMFA
>GmLEA2-96
MSQLNGAYYGPSIPPPKTSYHRPGRGGGCCCGCLFSLIFKLILTVIIIVGIAGFVFWLIVRPN
VVKFHVTDATLTQFNYTANNTLHYDLALNITVRNPNKRLGIYYDRIEARAMFHDARFDSQ
FPEPFYQGHKSTKVLNPVFKGQQVVPLNAEQSAELKKENATGVYEIDVKMYLRVRFKLG
VLKTKTLKPKVSCDLRVPFKGSAAFETTKCHWDR
>GmLEA2-97
MADKQPHLNGAYYGPAIPPAEQPRYRPHRERSCCCCLFGILWKILVALIVLVGLAVLIFWLV
VQPRYFKFYVTEADLTQFDYYSNNNTLHYNMVLNFTARNPNKKLSIYYDKVEALAFYED
VRFANYSVITPMNSFRQYKKSSSTMSAVLSGQQVLPLDNDLVSELNQDKIGGVYEIYVKL
YFRIRFRLGDVKTRRFKPKVKCDAKVPLRTMGNVTLFQTTKCDVDY
>GmLEA2-98
MADKQPHLNGAYYGPAIPPAEQPRYRPHRGRSCCCCLFGILWKILVALIVLVGLAILIFWLV
VQPRYFKFHVTKADLTQFDYYSNNNTLHYNMVLNFTARNPNKKLSIYYDKVEALAFYED

VRFANYDVITHMNSFRQYKKSSSPMSAVFTGQQVLMLNNEQVSELNQDKNAGVYDIYV
KLYFRIRFRLGDVISNDYKPKVKCHLKVPFSKNGTFTLFPTTKCDVDF
>GmLEA2-99
MSVKECHHHHKGKKHKLWRRIFWGIVIFAFIVLLTVLIIWAILKPSKPTFILQDVTVYGFNA
TIPNFLTSSFQVTLSSRNPNDKIGIYYDRLNTYVTYRNQQVTYRTSIPPSYQGHKEEDVWSP
FVYGTNVPVAPYNFVGLSQDQTNGNVLVLVKIDGKVRWKVGSFVSAHYNLNVRCPAFITF
GPQSNGIALGNNAVKYQLVQRCTVGV
>GmLEA2-100
MSKDKVSGDPRRAVCTGITIFLLLAGVTLLVLWLVYRPHKPRFTVIGAAIYGLNTSTPPLM
STTMQFSVLIKNPNRRVSIYYDRFSAFVSYRNQAITPQVLLPPLYQEKRSSVSVSPVIGGTPL
PVSVEVSNGLAMDEAYGVVGLRLIFQGRVRWKAGAIKTAHYGLYVKCDVLMGLKKGLV
GQVPLLGVTPCDVDL
>GmLEA2-101
MSTSDKSEVVERGAKDDEKPKEEHEKGGFLDTVKDFIQDIGEKIEGAVGFGKPTADVTGI
HIPSINLEKAELVVDVLIKNPNPVPIPLIDINYLVESDGRKLVSGLIPDSGTIHAHGEETVKIPL
TLIFDDIRNTYDDIKPGSIIPYRVKVDLIVDVPVFGRLTLPLEKTGEIPIPYKPDIDLDKIQFER
FSFEETVAILHLKLDNKNDFDLGLNALDYEVWLGDVSIGGAELAKSAKLVKSGISDIDVPI
TFRPKDFGSALWDMIRGRGTGYTFKGHIDVDTPFGAMKLPITKEGGTTRLKKNEDSGDDE
E
>GmLEA2-102
MHNNDHIPVHHVQGPNPKPVKLNRHHTMRYYAHRVHESLTTRVSKMICATFLGLLFIVGL
ITFILWLSLRPHRPRFHIHEFNIPGLTQDSGFENAVITFKVSARNSNQNIGVYYESMDGAVY
YRDTKIGYTPLLYPFYQQPKNTTEVDGDLSGATLTVSSQRWSEFQSDRADGSVVFRLELTS
VIRFKISTWDSKRHTMHANCNVGVGPDGSLLTIYKDKRCPVYFS
>GmLEA2-103
MADHQRLRIHPMEGEAPPPPPTTPLVPPGSSKSEKRIPLHHPPQLRAMPAAYPTAHKRSCC
CKCICWIITLLVLLLIILAASVGILYLVFKPKLPDYSVDTLRISDLRLNFDMSLYARFDVKITA
TNPNKKIGIYYKKGGRLSVWYTNTRLCEGSLPQFYQGHENKTMLNVSLSGQVQSGSTLM
NALQQQQQTGRIPLDLKVHAPIAIKLGRLKLMKVRVLGECLLVVDSLSSNNLISIKASNCK
FRLKL
>GmLEA2-104
MATTMPKFKRKLVMGANGRTNPLVWLVAIICTIIAVAVVVVGIVVFIGYIVIHPRVPVISVTN
AHLDLLRNDYAGLLQTQLTIVVTAKNGNAKAHASFSAITFNISYQGQDIAVLVADPFEVPK
NSSKDLSYVVQSSSIPLTPDQMEEVNDAWKRNEIEFDFKGAARTQWRVGPLGSVKFLCHL
DCDLKFRPVNGTYIPSRCTSKSH
>GmLEA2-105
MADKQPHLNGTYYGPAIPPAEQPRNRHHHGRSCCCCLFGILWKILVALIVLVGLVFLIFWL
VVQPRSFKFQVTEADLTQFDYYTNNLTLHYNMVLNFTARNPNKKLSIYYDKVEALAFYE
DARFANYDVITHMNSFRQYKKSTSPMSAVFSGKKVLMLNSEQVSKLNQDKSDGVYDIYV
KLNFRIRFRLGDSISGNLKPKVKCHLKVPFSKSGTFTLFETTKCSVHF
>GmLEA2-106
MAEMQIQHQEDHNYNPNHIPRPSPSPNPTQYTQYRFPGKVPHHQHQVHNMDKATPSRFK
PNAPKRQHCICITVFLLLLGIILLVLWLAYHPNKPRFTVASASVYSLNATSPPLMSIAMQFN
VVIKNPNRRVSISFDRLSAYVSYRNQPVTPHVMLPPLFIEKNSAVSLSPEIGGVAVPVSEDLT

NGMAMDENYGVVGVKLVLSGRLRWRAGDINSAHYGFYVKCDVLMGLRKGFVGQVPL
LGAPVCDVNT
>GmLEA2-107
MPRENPDYELFFCRMAATCIAFLIIILIFVLCIIFSTYTPSFTVTSITVSKFNVTTHAEELTATF
NVEGILENPNLAYSIRYQSLDLALWFDNFTIASTTIKQPPFSTQGQTDTPVRAQFAMARRW
LPSGLASEIVAQRNYHGSVDFGATLVARFRYKFGVLHSKKVHHFKLYCHPLHVALHSNSTT
GKLVALVDCKSV
>GmLEA2-108
MPCKPRCIYCTIYIFIFIFMLCFFLFLIIINPSSVKFYVTEATLTQFNLTSNNTLYYNFKVNITV
TNPNKHMIVYYRTIKAIAWYKDNEFDRVSLTPFDQGYKNTTFLRAVFVGQSVIMLKPKQL
GEYKDETSIGIYNDLAVDFDLRIRAKFGRIKSRRFKFDFLKYLCGENCRSFGN
>GmLEA2-109
MVHNCSSTASLIGHNLCHVIQGLGKKSFSLISRSHYSLCHSLKRSSLAIVTFLVLSRAPPPIA
LLSVASFKGCCCCLFLLFPFLALLVLAVVLVIIVAVKPKKLQFDLEQLGVQYMGITHNPPSIA
SLSLTIHLLFAATNPNKVGIKYGQSSFTFMYRGIPLGKATIFDFFQQPYSTRQLIAVDRVNLL
EVDAADLIRDASLRFLGDVAAKIRFNLRTASGDVVPINLEIEDTCRRSNAARRRREQDTEG
SSYTSPPLSPHHAKMDGEPARRVTLEDFSNTATPHFFTSIAKPEVQAANICYPHSLIQLIQGN
LFHGLPSEDPYAHLASYIEICNTVKIAGVPEDAEEVVEKFLKKYFPESKTAEGKMEISSFHQ
FPDESLSEALDRFHGLLRKTLTHGYSEPVQLNIFIDGLRPQSKQLLDASAGGKIKLKTPKEA
MELIENMAASDQAILHDRSYMPTKRSLLELGTQDATLAHNKLLTRQIEALTETLSKLPQQL
QAVSSSHSSVLQVEGCPTCGGTHEPGQCVSQQDTSQEVNYMGIPNRRFQGYNQGNSSGF
HQGGAGFNHGPPGFNQGRNFMQGSGWRNQGNQYKEQRNQQPYQPPCPHPSQGPNQQE
KPTNIEELLL
>GmLEA2-110
MSLLFLIIVAIVIVALFFTIFRPKDPNIIVHPVGFENFNPSLLTNVTANVSLGMLITIENSNYEN
FEYPNATGYIKFHDTVVGQVPIVGELVPPRSQINVNTSANFMVAKLINDPNFLSDFLSGIVN
FTSTASLPGKAHMLKIIKFKATVYSSCDISLNITSRNVDSKCISKIKL
>GmLEA2-111
MVACCSMRCSKGLKICCGVTALLLIILIVVLVVLFFTIFKPKDPDIVLQSVKLDGFKLEVFPT
LKLNVSLGIVVTVENPNHGSFTYQNSTAFLYYRGNLVAEAPLHQDTIPARNDHNISTSLTIF
VDLTKFKDLPSDYSGGVINFTSTTTLLGKVKVLDLFKIKATSYSTCDLSLFVNDQTINSTCN
SEIKF
>GmLEA2-112
MFFFAESPNDILKPRRNIEPSLSLSLPLSSLSLWEEQSRRSNRKQNHRTHSEPPVLRSSGAKN
RSATRTAPPRPKTAPLHMCPSRMGATLLRPAAIAAATAAIDNYVNTTYSDCSDFSDYSLAL
NLVLRFCQAKKQYHHSAFNGSSVEQPLKCNPTHPRSTVSLLNFRDCRLSFDNGSFEGTSRI
KVGSGKGRKVCLTVTGVVIAIVLLIVILALTVFKAKHPVTIVDSTKLEDFHVSLDPVKLRV
DLNVTLGVDVSVKNPNKVGFQYSDSAAHLNYRGQLIGEVPISAGEISSGETKGFNLTHTIM
ADRLLSNSQLLSDVTSGTLPLSTFVRMSGKVSILGFIKVHVVSSTSCDVAINLSNGTVGNQ
ECQYKTKL
>GmLEA2-113
MLITIENPNYENFEYPNATGYVKFLDTVVGQVPIVGELVPPRSQINVNTSANFMVSKLIND
PNFLSDFLSGIVNFTSTASLPGKAHMLKIIKFKATVYSLCDISINITSRNVDSNCISKIKI

Table S3. Pairwise identities between homologous pairs of soybean LEA2 proteins.

Gene name	Score	Expect	Identities	Positives	Gaps	Duplication pattern
GmLEA2-5/52	397bits (1019)	5.00E-146	189/207(91%)	198/207 (95%)	0/207 (0%)	
GmLEA2-17/99	384 bits (985)	5.00E-140	188/197(95%)	193/197 (97%)	0/197 (0%)	segmental
GmLEA2-59/64	387 bits (994)	4.00E-142	202/214(94%)	207/214 (96%)	1/214 (0%)	
GmLEA2-65/74	329 bits (844)	5.00E-119	178/221(81%)	202/221 (91%)	3/221 (1%)	segmental
GmLEA2-22/30	344 bits (883)	4.00E-125	170/177(96%)	173/177 (97%)	1/177 (0%)	
GmLEA2-18/100	376 bits (966)	2.00E-138	184/197(93%)	193/197 (97%)	0/197 (0%)	segmental
GmLEA2-33/106	424 bits (1091)	2.00E-155	226/249(91%)	237/249 (95%)	2/249 (0%)	segmental
GmLEA2-54/102	460 bits (1183)	3E-170	218/227(96%)	221/227 (97%)	0/227 (0%)	segmental
GmLEA2-34/48	497 bits (1279)	0	271/314(86%)	280/314 (89%)	6/314 (1%)	
GmLEA2-76/83	525 bits (1351)	0	264/305(87%)	274/305 (89%)	10/305 (3%)	segmental
GmLEA2-58/63	494bits (1272)	3.00E-180	286/347(82%)	292/347 (84%)	30/347 (8%)	
GmLEA2-23/90	594 bits (1531)	0	311/324(96%)	316/324 (97%)	1/324 (0%)	
GmLEA2-36/46	559 bits (1441)	0	283/297(95%)	291/297 (97%)	0/297 (0%)	
GmLEA2-4/55	212 bits (540)	3.00E-74	105/124(85%)	112/124 (90%)	0/124 (0%)	segmental
GmLEA2-69/86	391bits (1004)	5.00E-144	186/203(92%)	197/203 (97%)	0/203 (0%)	segmental
GmLEA2-37/39	353 bits (905)	1.00E-128	179/209(86%)	185/209 (88%)	10/209 (4%)	tandem
GmLEA2-6/50	390 bits (1001)	5.00E-142	212/246(86%)	222/246 (90%)	4/246 (1%)	
GmLEA2-12/96	395 bits (1015)	5E-145	202/221(91%)	208/221 (94%)	5/221 (2%)	segmental
GmLEA2-13/97	434 bits (1116)	4E-160	208/227(92%)	217/227 (95%)	0/227 (0%)	segmental
GmLEA2-16/98	448 bits (1153)	1E-165	213/229(93%)	222/229 (96%)	2/229 (0%)	segmental
GmLEA2-15/105	401 bits	6E-147	194/197(98%)	195/197	0/197	tandem

	(1031)			(98%)	(0%)	
GmLEA2-21/29	413 bits (1061)	9E-151	206/241(85%)	220/241 (91%)	3/241 (1%)	segmental
GmLEA2-24/43	389 bits (998)	2E-141	208/250(83%)	222/250 (88%)	6/250 (2%)	segmental
GmLEA2-67/70	249 bits (635)	4E-88	143/232(62%)	154/232 (66%)	66/232 (28%)	segmental
GmLEA2-8/80	443 bits (1139)	4E-162	256/273(94%)	262/273 (95%)	0/273 (0%)	segmental
GmLEA2-53/103	406 bits (1043)	3E-148	228/253(90%)	237/253 (93%)	4/253 (1%)	segmental
GmLEA2-7/82	448 bits (1153)	1E-164	242/260(93%)	252/260 (96%)	5/260 (1%)	
GmLEA2-62/91	431 bits (1107)	4E-158	229/246(93%)	236/246 (95%)	2/246 (0%)	
GmLEA2-20/28	522 bits (1345)	0	250/256(98%)	253/256 (98%)	0/256 (0%)	segmental
GmLEA2-75/84	504 bits (1299)	0	244/254(96%)	248/254 (97%)	0/254 (0%)	segmental
GmLEA2-44/85	373 bits (957)	7E-135	232/260(89%)	236/260 (90%)	7/260 (2%)	segmental
GmLEA2-1/49	407 bits (1045)	2E-149	198/216(92%)	205/216 (94%)	3/216 (1%)	
GmLEA2-10/78	449 bits (1154)	1E-165	218/224(97%)	222/224 (99%)	0/224 (0%)	segmental
GmLEA2-73/107						
GmLEA2-60/61						
GmLEA2-26/41	356 bits (914)	7E-130	185/190(97%)	189/190 (99%)	0/190 (0%)	segmental
GmLEA2-2/57	271 bits (694)	1E-97	137/186(74%)	151/186 (81%)	11/186 (5%)	
GmLEA2-110/113	232 bits (592)	2E-83	114/122(93%)	118/122 (96%)	0/122 (0%)	segmental
GmLEA2-66/72	336 bits (862)	7E-123	166/186(89%)	171/186 (91%)	0/186 (0%)	segmental
GmLEA2-25/40	376 bits (965)	3E-137	201/227(89%)	212/227 (93%)	4/227 (1%)	segmental
GmLEA2-45/81	288 bits (736)	1E-103	144/161(89%)	149/161 (92%)	0/161 (0%)	
GmLEA2-11/112	374 bits (959)	2E-134	187/192(97%)	189/192 (98%)	0/192 (0%)	
GmLEA2-35/47	272 bits (695)	6E-97	137/151(91%)	141/151 (93%)	1/151 (0%)	

GmLEA2-88/95	266 bits (680)	5E-96	131/152(86%)	141/152(92%)	0/152(0%)	
GmLEA2-9/79	617bits (1590)	0	313/319(98%)	315/319(98%)	0/319(0%)	segmental

Table S4. The amino acid sequences of Arabidopsis proteins of LEA2 domain.
>AT1G01470.1
MASLLDKAKDFVADKLTAIPKPEGSVTDVDLKDVNRDSVEYLAKVSVTNPYSHSIPICEISFTFHSAGREIGKGKIP
DPGSLKAKDMTALDIPVVVPYSILFNLARDVGVDWDIDYELQIGLTIDLPVVGEFTIPISSKGEIKLPTFKDFF
>AT1G08160.1
MVPPNPAHQPARRTQPQLQPQSQPRAQPLPGRRMNPVLCIIVALVLLGLLVGLAILITYLTLRPKRLIYTVEAASV
QEFAIGNNDDHINAKFSYVIKSYNPEKHVSVRYHSMRISTAHHNQSVAHKNISPFKQRPKNETRIETQLVSHNVA
LSKFNARDLRAEKSKGTIEMEVYITARVSYKTWIFRSRRRTLKAVCTPVMINVTSSSLDGFQRVLCKTRL
>AT1G17620.1
MTDDRVYPASKPPAIVGGGAPTTNPTFPANKAQLYNANRPAYRPPAGRRRTSHTRGCCCRCCCWTIFVIILLLLI
VAAASAVVYLIYRPQRPSFTVSELKISTLNFTSAVRLTTAISLSVIARNPNKNVGFIYDVTDITLYKASTGGDDDVVI
GKGTIAAFSHGKKNTTTLRSTIGSPPDELDEISAGKLKGDLKAKKAVAIKIVLNSKVKVKMGALKTPKSGIRVTCEG
IKVVAPTGKKATTATTSAAKCKVDPRFKIWKITF
>AT1G32270.1
MVRSNDVKFQVYDAELTHFDLESNNNLQYSLSLNLSIRNSKSSIGIHYDRFEATVYYMNQRLGAVPMPLFYLGSK
NTMLLRALFEGQTLVLLKGNERKKFEDDQKTGVYRIDVKLSINFRVMVLHLVTWPMKPVVRCHLKIPLALGSSN
STGGHKKMLLIGQLVKDTSANLREASETDHRRDVAQSKKIADAKLAKDFEAALKEFQKAQHITVERETSYIPFDP
KGSFSSSEVDIGYDRSQEQRVLMESRRQEIVLLDNEISLNEARIEAREQGIQEVKHQISEVMEMFKDLAVMVDH
QGTIDDIDEKIDNLRSAAAQGKSHLVKASNTQGSNSSLLFSCSLLLFFFLSGDLCRCVCVGSENPRLNPTRRKAW
CEEEDEEQRKKQQKKKTMSEKRRREEKKVNKPNGFVFCVLGHK
>AT1G52330.1
MTVEEDPKATPSLPDYKPLPSSSSHELNDAVLISSHPSPPSRRRFIISIFLISFASILIYIFWPSDPRIKIIRVKISHVHVH
RRPVPSIDMTLLVTLKVSNADVYSFDFTDLDVTIDYRGKTLGHVSSDGGHVTAFGSSYLDAEAELDGVMVFPDVI
HLIHDLAKGSVEFDTVTETNGKLGVLFFRFPLKAKVACGILVDTVNQTISRQSCSPL
>AT1G54540.1
MGDQQKIHPVLQMEANKTKTTTPAPGKTVLLPVQRPIPPPVIPSKNRNMCCKIFCWVLSLLVIALIALAIAVAVV
YFVFHPKLPSYEVNSLRVTNLGINLDLSLSAEFKVEITARNPNEKIGIYYEKGGHIGVWYDKTKLCEGPIPRFYQGH
RNVTKLNVALTGRAQYGNTVLAALQQQQQTGRVPLDLKVNAPVAIKLGNLKMKKIRILGSCKLVVDSLSTNNNI
NIKASDCSFKAKL
>AT1G61760.1
MHNKVDSLPVRSNPSTRPISRHHSASNIVHRVKESLTTRVSKLICAIFLSLLLCLGIITFILWISLQPHRPRVHIRGFSI
SGLSRPDGFETSHISFKITAHNPNQNVGIYYDSMEGSVYYKEKRIGSTKLTNPFYQDPKNTSSIDGALSRPAMAV
NKDRWMEMERDRNQGKIMFRLKVRSMIRFKVYTWHSKSHKMYASCYIEIGWDGMLLSATKDKRCPVYFT
>AT1G64065.1
MVDEDRITLAPTEIYGRSDEEQSGPRIWRRKTEEPPGKCLVYSLTIIVIIFALCLILSSIFLRISKPEIETRSISTRDLRSG
GNSTNPYFNATLVSDISIRNSNFGAFEFEDSTLRVVYADHGVVGETKIEGRRVEAHKTVRITGVVVEIGSFRLLDT
KDLDKDLRLGFLELRSVAEVRGRIKVLGRKRWKVSVMSCTMRLNLTGRFIQNLLCE
>AT1G65690.1
MSQHQKIYPVQDPEAATARPTAPLVPRGSSRSEHGDPSKVPLNQRPQRFVPLAPPKKRRSCCCRCFCYTFCFLLL
LVVAVGASIGILYLVFKPKLPDYSIDRLQLTRFALNQDSSLTTAFNVTITAKNPNEKIGIYYEDGSKITVWYMEHQLS
NGSLPKFYQGHENTTVIYVEMTGQTQNASGLRTTLEEQQQRTGNIPLRIRVNQPVRVKFGKLKLFEVRFLVRCG
VFVDSLATNNVIKIQSSSCKFRLRL
>AT2G01080.1
MPPPPSSSRAGLNGDPIAAQNQQPYYRSYSSSSSASLKGCCCCLFLLFAFLALLVLAVVLIVILAVKPKKPQFDLQQ

VAVVYMGISNPSAVLDPTTASLSLTIRMLFTAVNPNKVGIRYGESSFTVMYKGMPLGRATVPGFYQDAHSTKNV
EATISVDRVNLMQAHAADLVRDASLNDRVELTVRGDVGAKIRVMNFDSPGVQVSVNCGIGISPRKQALIYKQC
GFDGLSV
>AT2G27080.1
MAERVYPADSPPQSGQFSGNFSSGEFPKKPAPPPSTYVIQVPKDQIYRIPPPENAHRFEQLSRKKTNRSNCRCCF
CSFLAAVFILIVLAGISFAVLYLIYRPEAPKYSIEGFSVSGINLNSTSPISPSFNVTVRSRNGNGKIGVYYEKESSVDVY
YNDVDISNGVMPVFYQPAKNVTVVKLVLSGSKIQLTSGMRKEMRNEVSKKTVPFKLKIKAPVKIKFGSVKTWT
MIVNVDCDVTVDKLTAPSRIVSRKCSHDVDLW
>AT2G27260.1
MQDPSRPATGYPYPYPYPYPNPQQQQPPTNGYPNPAAGTAYPYQNHNPYYAPQPNPRAVIIRRLFIVFTTFLLLLGL
ILFIFFLIVRPQLPDVNLNSLSVSNFNVSNNQVSGKWDLQLQFRNPNSKMSLHYETALCAMYYNRVSLSETRLQ
PFDQGKKDQTVVNATLSVSGTYVDGRLVDSIGKERSVKGNVEFDLRMISYVTFRYGAFRRRRYVTVYCDDVAVG
VPVSSGEGKMVGSSKRCKTY
>AT2G30505.1
MSELLPPPPPPPPPPPPPRRDSGFGDPALVFPEFAAKQCGLEKVIEEEGEGEDSSYGKGSFDLSSRFSSPSISRNRF
DDVENPKKASSFKRPPSASRLSGFREEEEADRSRKSGSFVDHIGQEDKRICASGCFRKCCACTCMFVSVVLII
VLLVGLSANSSIKSILPQVLVTNLKFSRLDIAKSSTDLLMNANLNTVLQLSNNNDKTVLYYSPMKADISSENINLGK
KTLSGFKQDPGNVTSLKILTRLRKSKVYDVDATLLTNKEKTLEALVDVFLRGKLSVDWLGFKVHIPIVIACESVKQS
DVINGLKPACDVRIFSE
>AT2G35460.1
MANGLNGASYGPPIKPPVKTYYSHGRRGSDVGCGICGCFSSCLLCCGGCLVNIICNILIGVLVCLGVVALILWFILR
PNVVKFQVTEADLTRFEFDPRSHNLHYNISLNFSIRNPNQRLGIHYDQLEVRGYYGDQRFSAANMTSFYQGHK
NTTVVGTELNGQKLVLLGAGGRRDFREDRRSGVYRIDVKLRFKLRFKFGFLNSWAVRPKIKCHLKVPLSTSSSDE
RFQFHPTKCHVDL
>AT2G35960.1
MTTKDCGNHGGGGGGGTASRICGVIIGFIIIVLITIFLVWIILQPTKPRFILQDATVYAFNLSQPNLLTSNFQITIASR
NRNSRIGIYYDRLHVYATYRNQQITLRTAIPPTYQGHKEDNVWSPFVYGNSVPIAPFNAVALGDEQNRGFVTLII
RADGRVRWKVGTLITGKYHLHVRCQAFINLADKAAGVHVGENAVKYMLINKCSVNV
>AT2G35970.1
MTTKECGNHGGGGGGGGTACRICGAIIGFIIIVLMTIFLVSIILQPKKPEFILQDTTVYAFNLSQPNLLTSKFQITIAS
RNRNSNIGIYYDHLHAYASYRNQQITLASDLPPTYQRHKENSVWSPLLYGNQVPIAPFNAVALGDEQNSGVFTL
TICVDGRVRWKVGTLTIGNYHLHVRCQAFINQADKAAGVHVGENTVKYTLINKCSVNF
>AT2G35980.1
MAAEQPLNGAFYGPSVPPPAPKGYYRRGHGRGCGCCLLSLFVKVIISLIVILGVAALIFWLIVRPRAIKFHVTDASL
TRFDHTSPDNILRYNLALTVPVRNPNKRIGLYYDRIEAHAYYEGKRFSTITLTPFYQGHKNTTVLTPTFQGQNLVIF
NAGQSRTLNAERISGVYNIEIKFRLRVRFKLGDLKFRRIKPKVDCDDLRLPLSTSNGTTTTSTVFPIKCDFDF
>AT2G41990.1
MHAKTDSEATSIDAAALSPPRSAIRPLYYVQSPSNHDVEKMSFGSGCSLMGSPTHPHYYHCSPIHHSRESSTSRF
SDRALLSYKSIRERRRYINDGDDKTDGGDDDDPFRNVRLYVWLLLSVIFLFTVFSLILWGASKSYPPKVTVKGMLV
RDLNLQAGNDLSGVPTDMLSLNSTVRIYYRNPSTFFAVHVTASPLLLHYSNLLLSSGEMNKFTVGRNGETNVVT
VVQGHQIPLYGGVSFHLDTLSLPLNLTIVLHSKAYILGRLVTSKFYTRIICSFTLDANHLPKSISLLRSCIP
>AT2G44000.1
MSVYAETAYGRRSKDVDEARRIYQPKQSDITGKIIIFILAGLCMLLFIFIVIGFFFIAKPLTASLTSVEVRNLRYNNNTS
SSSSLYFNATLAMEIRIENPNLGFFEFPTSRGEILYNGDVVGEMRINGQRVASFGDLRTEVSAEVAYRGN

QKSPVWLKNDIERRLIILETRAKLRGEVHLKDLNKRTVNLKCLMQLNLSDDVIHRLWCK
>AT2G44060.1
MSTSEDKPEIISRVVHQEGDVEIVDRSQKDKDEEKEEGKGGFLDKVKDFIHDIGEKLEGTIGFGKPTADVSAIHIP
KINLERADIVVDVLVKNPNPVPIPLIDVNYLVESDGRKLVSGLIPDAGTLKAHGEETVKIPLTLIYDDIKSTYNDINP
GMIIPYRIKVDLIVDVPVLGRLTLPLEKCGEIPIPKKPDVDIEKIKFQKFSLEETVAILHVRLQNMNDFDLGLNDLDC
EVWLCDVSIGKAEIADSIKLDKNGSGLINVPMTFRPKDFGSALWDMIRGKGTGYTIKGNIDVDTPFGAMKLPIIK
EGGETRLKKEDDDDDDEE
>AT2G46140.1
MASADEKVVEEKASVISSLLDKAKGFFAEKLANIPTPEATVDDVDFKGVTRDGVDYHAKVSVKNPYSQSIPICQIS
YILKSATRTIASGTIPDPGSLVGSGTTVLDVPVKVAYSIAVSLMKDMCTDWDIDYQLDIGLTFDIPVVGDITIPVST
QGEIKLPSLRDFF
>AT2G46150.1
MADSEHVRPLAPATILPVSDESASNIKNTHRSRNRIKCSICVTATSLILTTIVLTLVFTVFRVKDPIIKMNGVMVNGL
DSVTGTNQVQLLGTNISMIVDVSVKNPNTASFKYSNTTTDIYYKGTLVGEAHGLPGKARPHRTSRMNVTVDIM
LDRILSDPGLGREISRSGLVNVWSYTRVGGKVKIMGIVKKHVTVKMNCTMAVNITGQAIQDVDCKKKIDL
>AT2G46300.1
MADYQMNPVLQKPPGYRDPNMSSPPPPPPPIQQQPMRKAVPMPTSYRPKKKRRSCCRFCCCCICITLVLFIFLL
LVGTAVFYLWFDPKLPTFSLASFRLDGFKLADDPDGASLSATAVARVEMKNPNSKLVFYYGNTAVDLSVGSGND
ETGMGETTMNGFRQGPKNSTSVKVETTVKNQLVERGLAKRLAAKFQSKDLVINVVAKTKVGLGVGGIKIGMLA
VNLRCGGVSLNKLDTDSPKCILNTLKWVTIQ
>AT3G05975.1
MSKRRICCIVSGIIFVLFVIFMTALILAQVFKPKHPILQTVSSTVDGISTNISLPYEVQLNFTLTLEMLLKNPNVADFE
YKTVENLVYYRDTLVGNLTLPSSTLPAKGSVLLPCPLFLQLDKFVANLGDIVQDVLHGKIVMETRAKMPGKITLLGI
FKIPLDSISHCNLVLGFPSMVVEDQVCDLKTKL
>AT3G11650.1
MGSKQPYLNGAYYGPSIPPPPKAHRSYNSPGFGCCCFSCLGSCLRCCGCCILSLICNILIAVAVILGVAALILWLIFR
PNAVKFYVADANLNRFSFDPNNNLHYSLDLNFTIRNPNQRVGVYYDEFSVSGYYGDQRFGSANVSSFYQGHKN
TTVILTKIEGQNLVVLGDGARTDLKDDEKSGIYRINAKLRLSVRFKFWFIKSWKLKPKIKCDDLKIPLGSSNSTGGF
KFQPVQCDFDLS
>AT3G11660.1
MKDCENHGHSRRKLIRRIFWSIIFVLFIIFLTILLIWAILQPSKPRFILQDATVYAFNVSGNPPNLLTSNFQITLSSRNP
NNKIGIYYDRLDVYATYRSQQITFPTSIPPTYQGHKDVDIWSPFVYGTSVPIAPFNGVSLDTDKDNGVVLLIIRAD
GRVRWKVGTFITGKYHLHVKCPAYINFGNKANGVIVGDNAVKYTFTTSCSVSV
>AT3G20590.1
MTKIDPEEELGRKCCTCFFKFIFTTRLGALILWLSLRAKKPKCSIQNFYIPALSKNLSSRDNTTLNFMVRCDNPNKD
KGIYYDDVHLTFSTINTTTTNSSDLVLVANYTVPKFYQGHKKKAKKWGQVWPLNNQTVLRAVLPNGSAVFRLDL
KTHVRFKIVFWKTKWYRRIKVGADVEVNGDGVKANEKEIKMEKSNFWKTHGYWSEFGFDDDVELTGDGAQK
KGSKTKKSDSSLPLRSSFPIFVLMNLLVFFAIR
>AT3G20600.1
MNNQNEDTEGGRNCCTCCLSFIFTAGLTSLFLWLSLRADKPKCSIQNFFIPALGKDPNSRDNTTLNFMVRCDNP
NKDKGIYYDDVHLNFSTINTTKINSSALVLVGNYTVPKFYQGHKKKAKKWGQVKPLNNQTVLRAVLPNGSAVFR
LDLKTQVRFKIVFWKTKRYGVEVGADVEVNGDGVKAQKKGIKMKKSDSSFPLRSSFPISVLMNLLVFFAIR
>AT3G24600.1
MKMYPKSDSDVTSLDLSSPKRPTYYVQSPSRDSDKSSSVALTTHQTTPTESPSHPSIASRVSNGGGGGFRWKGR

RKYHGGIWWPADKEEGGDGRYEDLYEDNRGVSIVTCRLILGVVATLSIFFLLCSVLFGASQSSPPIVYIKGVNVRS
FYYGEGSDNTGVPTKIMNVKCSVVITTHNPSTLFGIHVSSTAVSLIYSRQFTLANARLKSYHQPKQSNHTSRINLIG
SKVPLYGAGAELVASDNSGGVPVHTPNYTILSESRLSSSSRTSNGTSGMGFRWKGSSRRSNMYWPEKPYTINE
DEVYDDNRGLSVGQCRAVLVILGTVVVFSVFCSVLWGASHPFSPIVSVKSVDIHSFYYGEGIDRTGVATKILSFNS
SVKVTIDSPAPYFGIHVSSSTFKLTFSALTLATGQLKSYYQPRKSKHISIVKLTGAEVPLYGAGPHLAASDKKGKVPV
KLEFEIRSRGNLLGKLVKSKHENHVSCSFFISSSKTSKPIEFTHKTCKLVTK
>AT3G26350.1
MSHHHHHETNPHFARIPSQNPHLKSGGASTSQTSSNQPHIPPIPHPKKSHHKTTQPHPVAPPGILIKTRGRHRE
NPIQEPKHSVIPVPLSPEERLPPRKTQNSSKRPLLLSPEDNQQQRPPPPQAPQRNGGGYGSTLPPIPKPSPWRT
APTPSPHHRRGPRLPPPSRETNAMTWSAAFCCAIFWVILILGGLIILIVYLVYRPRSPYVDISAANLNAAYLDMGF
LLNGDLTILANVTNPSKKSSVEFSYVTFELYYYNTLIATQYIEPFKVPKKTSMFANVHLVSSQVQLQATQSRELQRQ
IETGPVLLNLRGMFHARSHIGPLFRYSYKLHTHCSVSLNGPPLGAMRARRCNTKR
>AT3G44220.1
MTEKECEHHHDEDEKMRKRIGALVLGFLAAVLFVVFLVWAILHPHGPRFVLQDATIYAFNVSQPNYLTSNLQVT
LSSRNPNDKIGIFYDRLDIYASYRNQQVTLATLLPATYQGHLDVTIWSPFLYGTTVPVAPYFSPALSQDLTAGMVLL
NIKIDGWVRWKVGTWVSGRYRLHVNCPAYITLAGHFSGDGPAVKYQLVQRCAVDV
>AT3G44380.1
MGSRDQKVKWSWSSALIGAASATAAASLLSAKPKDPTFHLISIDLTSLKLNLPVLDAELMLTVHVTNPNIAAIHYS
STKMTILYDGTVLGSAEVKAGSQPARSCQLLRLPARLDGMELAQHARQFFSDVANREMKLEAKLTIEGAAKVL
WWDHSFRVHVDSFVTVDPVFLDVIGQENKSQMDLFLT
>AT3G52470.1
MSKDCGNHGGGKEVVVRKLCAAIIAFIVIVLITIFLVWVILRPTKPRFVLQDATVYAFNLSQPNLLTSNFQVTIASR
NPNSKIGIYYDRLHVYATYMNQQITLRTAIPPTYQGHKEVNVWSPFVYGTAVPIAPYNSVALGEEKDRGFVGLMI
RADGTVRWKVRTLITGKYHIHVRCQAFINLGNKAAGVLVGDNAVKYTLANKCSVNV
>AT3G54200.1
MSDFSIKPDDKKEEEKPATAMLPPPKPNASSMETQSANTGTAKKLRRKRNCKICICFTILLILLIAIVIVILAFTLFKP
KRPTTTIDSVTVDRLQASVNPLLLKVLLNLTLNVDLSLKNPNRIGFSYDSSSALLNYRGQVIGEAPLPANRIAARKT
VPLNITLTLMADRLLSETQLLSDVMAGVIPLNTFVKVTGKVTVLKIFKIKVQSSSSCDLSISVSDRNVTSQHCKYST
KL
>AT4G01410.1
MGEGEAKAEHAAKADHKNAPSASSTPESYSKEGGGGGGDARRAICGAIFTILVILGIIALILWLVYRPHKPRLTVV
GAAIYDLNFTAPPLISTSVQFSVLARNPNRRVSIHYDKLSMYVTYKDQIITPPLPLPPLRLGHKSTVVIAPVMGGN
GIPVSPEVANGLKNDEAYGVVLMRVVIFGRLRWKAGAIKTGRYGFYARCDVWLRFNPSSNGQVPLLAPSTCKV
DV
>AT4G05220.1
MTPDRTTIPIRTSPVPRAQPMKRHHSASYYAHRVRESLSTRISKFICAMFLLVLFFVGVIAFILWLSLRPHRPRFHI
QDFVVQGLDQPTGVENARIAFNVTILNPNQHMGVYFDSMEGSIYYKDQRVGLIPLLNPFFQQPTNTTIVTGTL
TGASLTVNSNRWTEFSNDRAQGTVGFRLDIVSTIRFKLHRWISKHHRMHANCNIVVGRDGLILPKFNHKRCPV
YFT
>AT4G09590.1
MTTKECGNHGGGGGGGGTACRICGAIIGFIIIVLMTIFLVWIILQPKNPEFILQDTTVYAFNLSQPNLLTSKFQITIA
SRNRNSNIGIYYDHLHAYASYRNQQITLASDLPPTYQRHKEDSVWSPLLYGNQVPIAPFNAVALGDEQNSGVFTL
TICVDGQVRWKVGTLTIGNYHLHVRCQAFINQADKAAGVHVGENTVKYTLINKCSVNF
>AT4G13270.1

MASSKHEDYGIPYTPLPSSQPSQSVILLTPYRRHRRPSLLRNLRCSLLFTAVILLLSAAVYLLYPSDPDITVSRINLNHI
SVVDSHKIALDLSFSLTIKVRNRDFFSLDYDSLVVSIGYRGRELGLVKSKGGHLKARDSSYIDATLELDGLEVVHDVI
YLIGDLAKGVIPFDTIAQVQGDLGVLLFNIPIQGKVSCEVYVNVNNQKISHQDCHRK
>AT4G23610.1
MELAMSKINEDQAKPLAPLFLTTRSDQPDEEDQYHHDRTKYVHSQTKLILCCGFIASLTMLIAVTFIVLSLTVFHL
HSPNLTVDSISFNQRFDFVNGKVNTNQNTTVSVEISLHNPNPALFIVKNVNVSFYHGELVVVGESIRRSETIPAKR
TVKMNLTAEIVKTKLLASLPGLMEDLNGRGVDLKSSVEVRGRVKKMKIFRKTVHLQTDCFMKMTTNNFLTPTF
QCF
>AT4G23930.1
MSKSCSNLASCAVATLFIVFLIIAALTVYLTVFRPRDPEISVTSVKVPSFSVANSSVSFTFSQFSAVRNPNRAAFSHY
NNVIQLFYYGNRIGYTFVPAGEIESGRTKRMLATFSVQSFPLAAASSSQISAAQFQNSDRSGSTVEIESKLEMAGR
VRVLGLFTHRIAAKCNCRIAISSSDGSIVAVRC
>AT4G26490.1
MPNLPHLPPPPQLQPPETPSWETPSSVWYTPRTTPWRTPQSTQSTPVGQMVLTKPATVRFNGLDAEPRKDRV
ILRQPRSSRTSLWIWCVAGFCFVFSLLLIFFAIATLIVFLAIRPRIPVFDIPNANLHTIYFDTPEFFNGDLSMLVNFT
NPNKKIEVKFEKLRIELFFFNRLIAAQVVQPFLQKKHETRLEPIRLISSLVGLPVNHAVELRRQLENNKIEYEIRGTFK
VKAHFGMIHYSYQLHGRCQLQMTGPPTGILISRNCTTKK
>AT4G35170.1
MMTWPARSSPQNTRKPVYVVHSPPNTDVDKISTGSGFSPFGSPLNDQGQVSNFQHHSVAESSSYPRSSGPLR
NEYSSVQVHDLDRRTHEDEDYDEMDGPDEKRRRITRFYSCLLFTLVLAFTLFCLILWGVSKSFAPIATLKEMVLEN
LNVQSGNDQSGVLTDMLTLNSTVRILYRNPATFFTVHVTSAPLQLSYSQLILASGQMGEFSQRRKSERIIETKVFG
DQIPLYGGVPALFGQRAEPDQVVLPLNLTFTLRARAYVLGRLVKTTFHSNIKCSITFYGDKLGKTLDLSKSCSDH
>AT5G22200.1
MTGRYCDQHNGYEERRMRMMMRRIAWACLGLIVAVAFVVFLVWAILHPHGPRFVLQDVTINDFNVSQPNFL
SSNLQVTVSSRNPNDKIGIFYDRLDIYVTYRNQEVTLARLLPSTYQGHLEVTVWSPFLIGSAVPVAPYLSSALNED
LFAGLVLLNIKIDGWVRWKVGSWVSGSYRLHVNCPAFITVTGKLTGTGPAIKYQLVQRCAVDV
>AT5G21130.1
MTVEKPQEMTGDTNSDGFLTNKDVHRIKHPSLDTNDSSSSRYSVDSQKSRIGPPPGTYVIKLPKDQIYRVPPPE
NAHRYEYLSRRKTNKSCCRRCLCYSLSALLIIIVLAAIAFGFFYLVYQPHKPQFSVSGVSVTGINLTSSSPFSPVIRIKL
RSQNVKGKLGLIYEKGNEADVFFNGTKLGNGEFTAFKQPAGNVTVIVTVLKGSSVKLKSSSRKELTESQKKGKVPF
GLRIKAPVKFKVGSVTTWTMTITVDCKITVDKLTASATVKTENCETGLSLL
>AT5G06330.1
MTSKDCGSHDSHSSCNRKIVIWTISIILLLILVVILLVWAILQPSKPRFVLQDATVFNFNVSGNPPNLLTSNFQFTLS
SRNPNDKIGIYYDRLDVYASYRSQQITLPSPMLTTYQGHKEVNVWSPFVGGYSVPVAPYNAFYLDQDHSSGAI
MLMLHLDGRVRWKVGSFITGKYHLHVRCHALINFGSSAAGVIVGKYMLTETCSVSV
>AT5G06320.1
MADLNGAYYGPSIPPPKKVSHSHGRRGGGCGCLGDCLGCCGCCILSVIFNILITIAVLLGIAALIIWLIFRPNAIKFH
VTDAKLTEFTLDPTNNLRYNLDLNFTIRNPNRRIGVYYDEIEVRGYYGDQRFGMSNNISKFYQGHKNTTVVGTK
LVGQQLVLLDGGERKDLNEDVNSQIYRIDAKLRLKIRFKFGLIKSWRFKPKIKCDLKVPLTSNSTSGFVFQPTKCDV
DF
>AT5G22870.1
MCHKPKLELMPMETSPAQPLRRPSLICYIFLVILTLIFMAAVGFLITWLETKPKKLRYTVENASVQNFNLTNDNH
MSATFQFTIQSHNPNHRISVYYSSVEIFVKFKDQTLAFDTVEPFHQPRMNVKQIDETLIAENVAVSKSNGKDLRS
QNSLGKIGFEVFVKARVRFKVGIWKSSHRTAKIKCSHVTVSLSQPNKSQNSSCDADI

>AT5G36970.1
MSDHQKIHPVSDPEAPPHPTAPLVPRGSSRSEHGDPTKTQQAAPLDPPREKKGSRSCWCRCVCYTLLVLFLLIVI
VGAIVGILYLVFRPKFPDYNIDRLQLTRFQLNQDLSLSTAFNVTITAKNPNEKIGIYYEDGSKISVLYMQTRISNGSL
PKFYQGHENTTIILVEMTGFTQNATSLMTTLQEQQRLTGSIPLRIRVTQPVRIKLGKLKLMKVRFLVRCGVSVDSL
AANSVIRVRSSNCKYRFRL
>AT5G45320.1
MPRLTSRHGTSPFIWCAAIICAIISIVVIVGGIIVFVGYLVIHPRVPIISVADAHLDFLKYDIVGVLQTQLTIVIRVEND
NAKAHALFDETEFKLSYEGKPIAILKAPEFEVVKEKSMFLPYLVQSYPIPLNPTMMQAVDYAVKKDVITFELKGGS
RTRWRVGPLGSVKFECNLSCQLRFRPSDHSYIPSPCTSAHKH
>AT5G53730.1
MSQISITSPKHCAKKGGININNRHKKLFFTFSTFFSGLLLIIFLVWLILHPERPEFSLTEADIYSLNLTTSSTHLLNSSV
QLTLFSKNPNKKVGIYYDKLLVYAAYRGQQITSEASLPPFYQSHEEINLLTAFLQGTELPVAQSFGYQISRERSTGKII
IGMKMDGKLRWKIGTWVSGAYRFNVNCLAIVAFGMNMTTPPLASLQGTRCSTTI
>glycine-rich uncharacterized protein
MAKPHDRRRSSGRTNLASCAVATVFLLILLVVLLVVYFTVFKPKDPKISVNAVQLPSFAVSNNTANFSFSQYVAVR
NPNRAVFSHYDSSIQLLYSGNQVGFMFIPAGKIDSGRIQYMAATFTVHSFPISPPSSSAISTVSAAVIPDSPIIPGPP
DFTVTPRNPDSPYFPGYPESPDLPGNPGSPDFSGNPGPPSFPRNPGSPEFPGNPGAPIIPRNPGSPEFPINPPRN
PGAPVIPRNPNPPVFPGNPRSMGPPGFPGIGGPPGFPGTPFGGGGTGPTLGDGYANPGFGYGNRVGPTM
EIESKMELAGRVKVLHVFTHHVVAKSDCRVTVSIADGSVLGFHC

Table S5. The mutants selection of four homologous genes of *GmSYP24*.

gene name	mutant name	insertion type	Amplification primers	expression level
AT2G35460	SALK_064143	exon insertion	LP AGGGTGGTGGCTTAGATATCG; RP CGTGCCAATCTACATCTCTCC	downregulated 6.4 times
	SALK_069015	promoter insertion	LP ACAAACCAAGGCACTTCTACG; RP CTGAGTCAGACCCAGCAAAAG	upregulated 16.4 times
	SALK_073333	exon insertion	LP GAACCAAGACCAACCTATGCC; RP TTCACGTCGATCCTGTAAACC	downregulated 2.4 times
	SALK_106063	exon insertion	LP TGATATTCTCCTTGGGTTCCC; RP CGTGCCAATCTACATCTCTCC	upregulated 24.5 times
AT3G11650	SALK_008804	promoter insertion	LPTTTAGTGTAGTTCCATGAAACTAGGG; RPATGAGGATGTTGCAGATCAGG	upregulated 3.2 times
	SALK_129820	promoter insertion	LPTGAGAAACCTAACTTTGGATTTCC; RP TATAGCTGCGGTGAGCCTTAG	upregulated 11.5 times
AT5G06320	SALK_150318	exon insertion	LPGAGACGTCAGAAAACTCCGTG; RPAACTCACCGAATTCACACTCG	downregueated 2.6 times
	SALK_035427	exon insertion	LPTCTCTGATGGACATCCTGACC; RP:AACTCACCGAATTCACACTCG	downregueated 1.4 times
AT2G35980	CS423587	exon insertion	LPCATCGTCCTGATCTGCTTAGG; RPCTCCCAAAGGCTACTACCGAC	No homozygous transgenic lines
	SALK_132320	unknown insertion site	LPAGTCTACGACGCTGAGCTGAC; RPCTGAAACTCTTTCAACGCTGC	No homozygous transgenic lines
LBPrimer: ATTTTGCCGATTTCGGAAC				

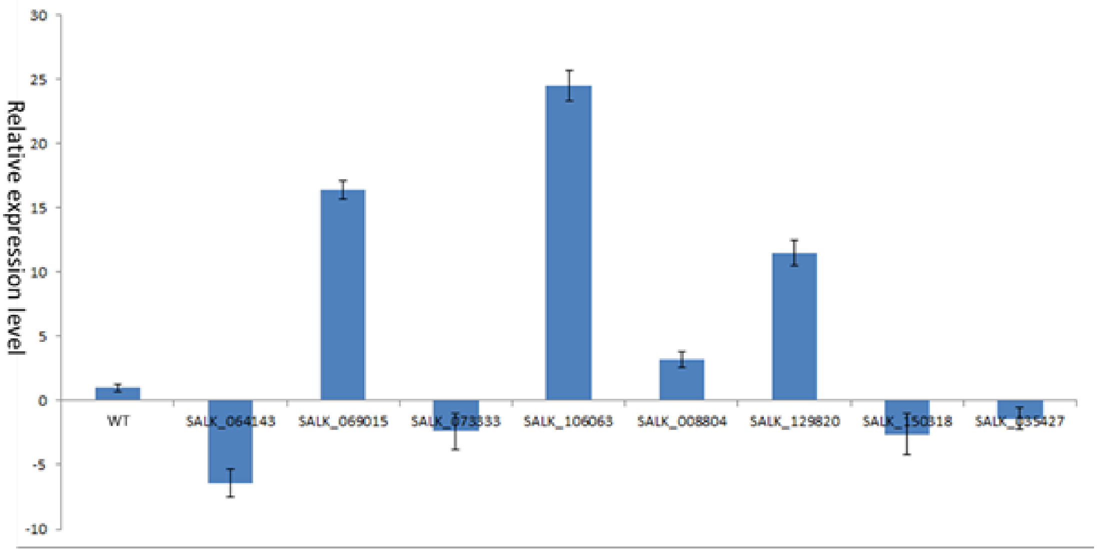
Relative expression level
30
25
20
15
10
5
0
-5
-10
WT
SALK_064143
SALK_069015
SALK_073333
SALK_106063
SALK_008804
SALK_129820
SALK_150318
SALK_035427

Table S6. Genes that were up-regulated in the GmSYP24ox transgenic plants under drought.

Locus	log2.Fold_change.	P-value	Q-value	Blast swissprot
Glyma.01G015400.Wm82.a2.v1	2.2027	1.4076E-07	7.7824E-06	unknown
Glyma.01G023300.Wm82.a2.v1	1.7189	9.05E-07	2.08E-05	sp|Q8W486|Y1491_ARATH Uncharacterized protein At1g04910 OS=Arabidopsis thaliana GN=At1g04910 PE=2 SV=1//2.97641e-83
Glyma.01G126600.Wm82.a2.v1	3.5778	1.55E-31	2.54E-29	sp|Q9C523|DIR19_ARATH Dirigent protein 19 OS=Arabidopsis thaliana GN=DIR19 PE=2 SV=1//5.46253e-61
Glyma.01G127200.Wm82.a2.v1	2.444	4.15E-64	1.67E-61	sp|Q9FI66|DIR3_ARATH Dirigent protein 3 OS=Arabidopsis thaliana GN=DIR3 PE=3 SV=1//1.68406e-49
Glyma.01G185800.Wm82.a2.v1	1.3681	0.00011231	0.0034201	sp|P22335|HSF24_SOLPE Heat shock factor protein HSF24 OS=Solanum peruvianum GN=HSF24 PE=2 SV=1//2.27036e-80
Glyma.01G228700.Wm82.a2.v1	1.5952	5.47E-54	1.88E-51	sp|P30081|CHS7_SOYBN Chalcone synthase 7 OS=Glycine max GN=CHS7 PE=3 SV=1//0
Glyma.01G232400.Wm82.a2.v1	1.7658	1.66E-11	7.50E-10	sp|P31687|4CL2_SOYBN 4-coumarate--CoA ligase 2 OS=Glycine max PE=2 SV=2//0
Glyma.02G208400.Wm82.a2.v1	1.4087	1.39E-19	1.22E-17	sp|P27608|AROF_TOBAC Phospho-2-dehydro-3-deoxyheptonate aldolase 1, chloroplastic OS=Nicotianatabacum GN=DHAPS-1 PE=2 SV=1//0
Glyma.03G174300.Wm82.a2.v1	1.8271	6.00E-08	3.47E-06	sp|Q9LZD3|E70A1_ARATH Exocyst complex component EXO70A1 OS=Arabidopsis thaliana GN=EXO70A1 PE=1 SV=1//3.9145e-68

Glyma.03G213300.Wm82.a2.v1	6.3721	8.55E-11	7.37E-09	sp|O82245|TSPO_ARATH Translocator protein homolog OS=Arabidopsis thaliana GN=TSPO PE=1 SV= 1//2.54929e-44
Glyma.04G050300.Wm82.a2.v1	1.5345	4.81E-06	0.00020339	sp|Q9FU27|C3H2_ORYSJ Zinc finger CCCH domain-containing protein 2 OS=Oryza sativa subsp. japonica GN=Os01g0192000 PE=2 SV=1//1.22277e-61
Glyma.04G089700.Wm82.a2.v1	1.7043	8.67E-11	7.43E-09	sp|Q9T074|PCKA_ARATH Phosphoenolpyruvate carboxykinase [ATP] OS=Arabidopsis thaliana GN= PCKA PE=1 SV=1//0
Glyma.04G104200.Wm82.a2.v1	1.9169	6.64E-06	0.00026914	sp|Q9Y7K5|YGI3_SCHPO Uncharacterized WD repeat-containing protein C2A9.03 OS= Schizosac -charomycespombe (strain 972/ ATCC 24843) GN=SPBC2A9.03 PE=3 SV=2//2.58827e-38
Glyma.05G044200.Wm82.a2.v1	1.2521	8.28E-06	0.00033017	sp|Q53EL6|PDCD4_HUMAN Programmed cell death protein 4 OS=Homo sapiens GN=PDCD4 PE=1 SV =2//4.99051e-26
Glyma.05G056300.Wm82.a2.v1	2.8705	5.76E-59	3.99E-56	sp|P29001|INVA_VIGRR Acid beta-fructofuranosidase OS=Vignaradiata var. radiata GN=INVA PE=1 SV=1//0
Glyma.05G180100.Wm82.a2.v1	1.8907	4.48E-17	6.31E-15	sp|O65201|ACOX2_ARATH Acyl-coenzyme A oxidase 2, peroxisomal OS=Arabidopsis thaliana GN=ACX2 PE=1 SV=2//0
Glyma.06G021200.Wm82.a2.v1	5.2677	1.13E-115	2.16E-112	sp|Q0JIL1|NRX2_ORYSJ Probable nucleoredoxin 2 OS=Oryza sativa subsp. japonica GN=Os01g0794400 PE=2 SV=1//1.57959e-114
Glyma.06G059600.Wm82.a2.v1	1.2725	1.36E-25	3.43E-23	sp|Q940T9|COL4_ARATH Zinc finger protein CONSTANS-LIKE 4 OS=Arabidopsis thaliana

				GN=COL4 PE=2 SV=2//5.89374e-93
Glyma.06G105200.Wm82.a2.v1	3.4919	5.25E-09	3.50E-07	sp|Q9Y7K5|YGI3_SCHPO Uncharacterized WD repeat-containing protein C2A9.03 OS=Schizosac -charomycespombe (strain 972 / ATCC 24843) GN=SPBC2A9.03 PE=3 SV=2//1.32105e-38
Glyma.06G216700.Wm82.a2.v1	1.6107	2.45E-09	1.75E-07	sp|Q94A82|NUD19_ARATH Nudix hydrolase 19, chloroplastic OS=Arabidopsis thaliana GN=NUDT19 PE=1 SV=1//0
Glyma.06G239900.Wm82.a2.v1	1.9392	6.81E-47	3.70E-44	sp|Q9T076|ENL2_ARATH Early nodulin-like protein 2 OS=Arabidopsis thaliana GN=At4g27520 PE=1 SV=1//1.22676e-40
Glyma.07G126800.Wm82.a2.v1	1.7026	5.75E-06	0.00023712	sp|O82199|C3H20_ARATH Zinc finger CCCH domain-containing protein 20 OS=Arabidopsis thaliana GN=At2g19810 PE=2 SV=1//1.47867e-130
Glyma.07G261500.Wm82.a2.v1	4.8253	2.20E-47	6.18E-45	sp|P92980|APR3_ARATH 5'-adenylylsulfate reductase 3, chloroplastic OS=Arabidopsis thaliana GN= APR3 PE=2 SV=2//0
Glyma.08G056600.Wm82.a2.v1	6.1673	3.13E-09	2.17E-07	sp|Q9C942|CSE_ARATH Caffeoylshikimate esterase OS=Arabidopsis thaliana GN=CSE PE=1 SV= 1//8.87112e-34
Glyma.09G010500.Wm82.a2.v1	2.291	1.91E-14	2.25E-12	unknown
Glyma.09G069800.Wm82.a2.v1	1.2887	4.74E-37	1.93E-34	sp|Q8RWQ9|ALEUL_ARATH Thiol protease aleurain-like OS=Arabidopsis thaliana GN=At3g45310 PE=2 SV=1//7.6746e-178
Glyma.09G173300.Wm82.a2.v1	2.3094	6.58E-05	0.002114	unknown
Glyma.09G190600.Wm82.a2.v1	2.1601	2.60E-20	4.51E-18	sp|Q9LV52|HSFC1_ARATH Heat stress transcription

				factor C-1 OS=Arabidopsis thaliana GN=HSFC1 PE=2 SV=1//1.19541e-70
Glyma.09G199000.Wm82.a2.v1	1.5502	1.39E-17	2.05E-15	sp|Q8W486|Y1491_ARATH Uncharacterized protein At1g04910 OS=Arabidopsis thaliana GN=At1g04910 PE=2 SV=1//2.47287e-83
Glyma.09G205700.Wm82.a2.v1	2.2597	4.26E-08	1.22E-06	sp|Q500U8|TKPR1_ARATH Tetraketide alpha-pyrone reductase 1 OS=Arabidopsis thaliana GN=TKPR1 PE=1 SV=1//1.00335e-34
Glyma.09G269600.Wm82.a2.v1	1.8934	2.60E-06	5.49E-05	sp|Q40316|VESTR_MEDSA Vestitone reductase OS=Medicago sativa PE=1 SV=1//4.14286e-173
Glyma.10G001700.Wm82.a2.v1	2.8948	3.19E-89	4.14E-86	sp|Q9XIW0|CIPK7_ARATH CBL-interacting serine/threonine-protein kinase 7 OS=Arabidopsis thaliana GN=CIPK7 PE=1 SV=1//4.03366e-150
Glyma.10G291400.Wm82.a2.v1	1.1681	1.53E-06	3.37E-05	sp|Q9C942|CSE_ARATH Caffeoylshikimate esterase OS=Arabidopsis thaliana GN=CSE PE=1 SV=1//4.32716e-49
Glyma.11G010500.Wm82.a2.v1	1.4475	1.45E-05	0.00025842	sp|P31687|4CL2_SOYBN 4-coumarate--CoA ligase 2 OS=Glycine max PE=2 SV=2//0
Glyma.11G011500.Wm82.a2.v1	1.5424	2.81E-46	7.52E-44	sp|P23569|CHSY_PUEML Chalcone synthase OS=Puerariamontana var. lobata GN=CHS PE=2 SV=1//0
Glyma.11G063600.Wm82.a2.v1	3.3452	1.78E-08	1.11E-06	sp|Q9SYY4|ELO3L_ARATH Elongation of fatty acids protein 3-like OS=Arabidopsis thaliana GN=HOS3 PE=2 SV=1//6.67054e-118
Glyma.11G070500.Wm82.a2.v1	3.4429	8.37E-13	4.30E-11	sp|P52575|IFR_MEDSA Isoflavone reductase OS=Medicago sativa GN=IFR PE=1 SV=1//0

Glyma.11G119500.Wm82.a2.v1	1.4381	3.36E-07	8.35E-06	sp|Q8VZ80|PLT5_ARATH Polyol transporter 5 OS=Arabidopsis thaliana GN=PLT5 PE=1 SV=2//0
Glyma.11G208700.Wm82.a2.v1	1.1571	5.10E-05	0.0016717	unknown
Glyma.12G117000.Wm82.a2.v1	2.1039	1.20E-10	1.02E-08	sp|Q9FE67|ERF80_ARATH Ethylene-responsive transcription factor 9 OS=Arabidopsis thaliana GN=ERF9 PE=2 SV=1//7.38365e-29
Glyma.12G134300.Wm82.a2.v1	1.9234	0.00015715	0.0045974	unknown
Glyma.13G145000.Wm82.a2.v1	1.2321	8.23E-17	5.84E-15	sp|P19142|PAL2_PHAVU Phenylalanine ammonia-lyase class 2 OS=Phaseolus vulgaris PE=3 SV=1//0
Glyma.13G360600.Wm82.a2.v1	1.2766	1.34E-05	0.00050859	sp|Q9M8L2|FBK30_ARATH F-box/kelch-repeat protein At1g80440 OS=Arabidopsis thaliana GN=At1g80440 PE=2 SV=1//6.28671e-123
Glyma.14G010500.Wm82.a2.v1	2.5757	2.96E-17	4.27E-15	sp|Q84VX0|RFS1_ARATH Probable galactinol--sucrose galactosyltransferase 1 OS=Arabidopsis thaliana GN=RFS1 PE=2 SV=1//0
Glyma.14G038200.Wm82.a2.v1	1.3152	8.78E-07	4.23E-05	sp|Q06445|CYTI_VIGUN Cysteine proteinase inhibitor OS=Vignaunguiculata PE=3 SV=1//6.14795e-42
Glyma.14G087600.Wm82.a2.v1	2.3909	2.12E-06	9.62E-05	sp|Q9STX1|ACBP3_ARATH Acyl-CoA-binding domain-containing protein 3 OS=Arabidopsis thaliana GN=ACBP3 PE=1 SV=1//3.08907e-32
Glyma.15G072400.Wm82.a2.v1	2.9506	7.98E-19	1.27E-16	sp|P24805|TSJT1_TOBAC Stem-specific protein TSJT1 OS=Nicotianatabacum GN=TSJT1 PE=2 SV=1//2.56932e-32
Glyma.16G100400.Wm82.a2.v1	2.5454	6.16E-30	1.92E-27	sp|A3FPF2|DEF_NELNU Defensin-like protein OS=Nelumbonucifera PE=3 SV=1//3.46437e-14
Glyma.17G034900.Wm82.a2.v1	4.633	5.36E-103	4.84E-100	sp|Q9NZT1|CALL5_HUMAN Calmodulin-like protein 5

				OS=Homo sapiens GN=CALML5 PE=1 SV=2//1.53851e-13
Glyma.17G096700.Wm82.a2.v1	1.4622	1.27E-22	2.61E-20	sp|Q02283|HAT5_ARATH Homeobox-leucine zipper protein HAT5 OS=Arabidopsis thaliana GN=HAT5 PE=1 SV=1//9.02554e-41
Glyma.17G235300.Wm82.a2.v1	1.6879	1.59E-16	2.11E-14	sp|J9U5U9|DAD2_PETHY Probable strigolactone esterase DAD2 OS=Petunia hybrida GN=DAD2 PE=1 SV=1//1.42839e-159
Glyma.18G009700.Wm82.a2.v1	1.02	1.68E-28	2.33E-26	sp|Q7FAH2|G3PC2_ORYSJ Glyceraldehyde-3-phosphate dehydrogenase 2, cytosolic OS=Oryza sativa subsp. japonica GN=GAPC2 PE=1 SV=1//0
Glyma.18G061100.Wm82.a2.v1	3.9037	0	0	sp|P49093|ASNS2_LOTJA Asparagine synthetase [glutamine-hydrolyzing] 2 OS=Lotus japonicus GN=AS2 PE=2 SV=2//0
Glyma.18G193400.Wm82.a2.v1	1.8893	4.58E-08	1.31E-06	sp|Q9SR40|LAC7_ARATH Laccase-7 OS=Arabidopsis thaliana GN=LAC7 PE=2 SV=1//0
Glyma.19G076500.Wm82.a2.v1	3.5398	8.84E-06	0.00034992	unknown
Glyma.19G089800.Wm82.a2.v1	2.2837	3.22E-38	1.39E-35	sp|P92941|CLCA_ARATH Chloride channel protein CLC-a OS=Arabidopsis thaliana GN=CLC-A PE=1 SV=2//0
Glyma.19G099400.Wm82.a2.v1	1.1542	4.22E-05	0.0014111	sp|Q9LW26|Y3684_ARATH Acyltransferase-like protein At3g26840, chloroplastic OS=Arabidopsis thaliana GN=At3g26840 PE=2 SV=1//0
Glyma.19G125300.Wm82.a2.v1	1.2203	1.84E-05	0.00067755	sp|O80683|CAB25_ARATH Calmodulin-binding protein 25 OS=Arabidopsis thaliana GN=CAMBP25 PE=1

				SV=1//2.72625e-22
Glyma.19G198600.Wm82.a2.v1	2.8398	3.65E-213	1.08E-209	sp|Q9SJ52|YLS9_ARATH Protein YLS9 OS= Arabidopsis thaliana GN=YLS9 PE=2 SV= 1// 2.36052e-62
Glyma.19G217700.Wm82.a2.v1	4.7525	1.38E-06	6.44E-05	sp|Q93XK2|STSYN_PEA Stachyose synthase OS= Pisumsativum GN=STS1 PE=1 SV=1//0
Glyma.19G245400.Wm82.a2.v1	3.3694	9.67E-08	2.64E-06	sp|P02877|HEVE_HEVBR Pro-hevein OS= Hevea-brasiliensis GN=HEV1 PE=1 SV= 2//3.75062e-99
Glyma.20G135200.Wm82.a2.v1	1.5041	1.37E-10	5.49E-09	unknown
Glyma.20G158400.Wm82.a2.v1	1.9212	0.00016959	0.0048902	unknown

Table S8. Genes that were up-regulated in the GmSYP24ox transgenic plants under ABA.

Locus	log2.Fold_change	P-value	Q-value	Blast swissprot
Glyma.01G006000.Wm82.a2.v1	1.6235	0.000451	0.0045645	unknown
Glyma.01G115900.Wm82.a2.v1	1.5826	1.76E-124	1.37E-121	sp|Q9XF88|CB4B_ARATH Chlorophyll a-b binding protein CP29.2, chloroplastic OS=Arabidopsis thaliana GN=LHCB4.2 PE=1 SV=1//3.49966e-165
Glyma.02G213700.Wm82.a2.v1	3.3283	4.45E-19	3.04E-17	sp|Q94CE3|BCA5_ARATH Beta carbonic anhydrase 5, chloroplastic OS=Arabidopsis thaliana GN=BCA5 PE=2 SV=1//2.9914e-97
Glyma.04G020300.Wm82.a2.v1	1.3446	6.09E-104	3.42E-101	sp|P17340|PLAS_SOLLC Plastocyanin, chloroplastic OS=Solanum lycopersicum GN=PETE PE=2 SV=1//1.86003e-69
Glyma.04G118700.Wm82.a2.v1	2.5952	1.86E-15	9.58E-14	sp|P17407|21KD_DAUCA 21 kDa protein OS=Daucuscarota PE=2 SV=1//7.00889e-71
Glyma.04G179500.Wm82.a2.v1	3.6466	2.41E-18	1.56E-16	sp|Q93X17|SNAK2_SOLTU Snakin-2 OS=Solanum tuberosum GN=SN2 PE=1 SV=1//3.43079e-22
Glyma.05G168400.Wm82.a2.v1	2.1553	1.10E-79	4.37E-77	sp|Q9ZTS2|FER_CAPAN Ferredoxin, chloroplastic OS=Capsicum annuum GN=AP1 PE=1 SV=1//3.53401e-71
Glyma.05G218400.Wm82.a2.v1	1.6683	5.64E-28	6.29E-26	sp|Q9FLE8|Y5986_ARATH Uncharacterized protein At5g39865 OS=Arabidopsis thaliana GN=At5g39865 PE=2 SV=1//1.12827e-26
Glyma.05G224500.Wm82.a2.v1	4.1785	3.41E-07	6.84E-06	sp|Q8H1S0|MIOX4_ARATH Inositol oxygenase 4 OS=Arabidopsis thaliana GN=MIOX4 PE=2 SV=1//7.88763e-179
Glyma.06G050100.Wm82.a2.v1	4.3766	8.92E-05	0.001091	sp|Q9M439|BCAT2_ARATH

				Branched-chain-amino-acid aminotransferase 2, chloroplastic OS=Arabidopsis thaliana GN=BCAT2 PE=1 SV=1//6.11164e-166
Glyma.06G102100.Wm82.a2.v1	3.2377	2.67E-50	5.85E-48	sp|Q9ZPE7|EXO_ARATH Protein EXORDIUM OS=Arabidopsis thaliana GN=EXO PE=2 SV=1//3.98167e-140
Glyma.06G117200.Wm82.a2.v1	3.7351	3.97E-28	4.47E-26	sp|Q0WPW4|ACCO5_ARATH 1-aminocyclopropane-1-carboxylate oxidase 5 OS=Arabidopsis thaliana GN=At1g77330 PE=2 SV=1//8.60588e-159
Glyma.06G121100.Wm82.a2.v1	2.6421	3.17E-06	5.33E-05	sp|P00303|BABL_CUCSA Basic blue protein OS=Cucumissativus PE=1 SV=1//6.00089e-48
Glyma.06G185300.Wm82.a2.v1	2.6502	8.36E-50	1.77E-47	sp|Q93X17|SNAK2_SOLTU Snakin-2 OS=Solanum tuberosum GN=SN2 PE=1 SV=1//1.98791e-22
Glyma.07G125600.Wm82.a2.v1	2.1205	1.39E-07	2.97E-06	unknown
Glyma.07G150400.Wm82.a2.v1	3.7216	4.92E-144	4.56E-141	sp|Q9SRM5|CPR49_ARATH GDSL esterase/lipase CPRD49 OS=Arabidopsis thaliana GN=CPRD49 PE=2 SV=1//1.65434e-91
Glyma.08G152500.Wm82.a2.v1	1.0563	7.67E-08	6.03E-06	sp|Q66GR3|BH130_ARATH Transcription factor bHLH130 OS=Arabidopsis thaliana GN=BHLH130 PE=1 SV=1//5.36096e-38
Glyma.08G171000.Wm82.a2.v1	3.8824	6.56E-23	5.55E-21	unknown
Glyma.08G200100.Wm82.a2.v1	2.9297	9.84E-202	1.45E-198	sp|P10743|VSPB_SOYBN Stem 31 kDa glycoprotein OS=Glycine max GN=VSPB PE=2 SV=1//0
Glyma.09G005700.Wm82.a2.v1	1.6272	5.01E-11	5.90E-09	sp|Q9C519|WRKY6_ARATH WRKY transcription factor 6 OS=Arabidopsis thaliana GN=WRKY6 PE=1

				SV=1//5.28188e-122
Glyma.09G089700.Wm82.a2.v1	1.3209	1.15E-12	1.66E-10	sp|Q93VD3|CIPKN_ARATH CBL-interacting serine/threonine-protein kinase 23 OS=Arabidopsis thaliana GN=CIPK23 PE=1 SV=1//0
Glyma.10G200700.Wm82.a2.v1	1.5059	4.85E-08	3.90E-06	sp|Q84WB7|GT645_ARATH Glycosyltransferase family protein 64 protein C5 OS=Arabidopsis thaliana GN=At5g04500 PE=2 SV=1//0
Glyma.11G088600.Wm82.a2.v1	2.058	4.28E-09	1.17E-07	sp|O14197|YDQ4_SCHPO Uncharacterized transporter C5D6.04 OS=Schizosaccharomycespombe (strain 972 / ATCC 24843) GN=SPAC5D6.04 PE=3 SV=1//2.83664e-06
Glyma.11G207500.Wm82.a2.v1	1.7387	1.94E-06	0.00011766	sp|Q9CAL3|CRK2_ARATH Cysteine-rich receptor-like protein kinase 2 OS=Arabidopsis thaliana GN=CRK2 PE=2 SV=1//2.1279e-108
Glyma.13G212600.Wm82.a2.v1	2.593	5.59E-14	2.59E-12	sp|Q8RXC8|RBK2_ARATH Receptor-like cytosolic serine/threonine-protein kinase RBK2 OS=Arabidopsis thaliana GN=RBK2 PE=1 SV=1//3.78835e-156
Glyma.14G086300.Wm82.a2.v1	2.3803	1.30E-286	3.99E-283	sp|Q8GXG1|ASPGB_ARATH Probable isoaspartyl peptidase/L-asparaginase 2 OS=Arabidopsis thaliana GN=At3g16150 PE=2 SV=2//0
Glyma.14G087200.Wm82.a2.v1	2.8531	7.43E-43	1.33E-40	sp|Q948Z4|SNAK1_SOLTU Snakin-1 OS=Solanum tuberosum GN=SN1 PE=1 SV=1//5.29715e-31
Glyma.14G093100.Wm82.a2.v1	1.1126	3.75E-15	6.99E-13	sp|P43399|MT1_TRIRP Metallothionein-like protein 1 OS=Trifoliumrepens GN=MT1B PE=3 SV=1//1.07989e-13
Glyma.14G220000.Wm82.a2.v1	1.6715	3.33E-10	1.06E-08	sp|B6ETT4|SYT2_ARATH Synaptotagmin-2

				OS=Arabidopsis thaliana GN=SYT2 PE=2 SV=1//0
Glyma.15G072400.Wm82.a2.v1	2.9329	1.03E-17	6.42E-16	sp|P24805|TSJT1_TOBAC Stem-specific protein TSJT1 OS=Nicotianatabacum GN=TSJT1 PE=2 SV=1//2.56932e-32
Glyma.15G110300.Wm82.a2.v1	1.2815	2.83E-06	0.00016449	sp|Q9C519|WRKY6_ARATH WRKY transcription factor 6 OS=Arabidopsis thaliana GN=WRKY6 PE=1 SV=1//2.29689e-126
Glyma.16G173000.Wm82.a2.v1	2.8834	5.88E-09	5.28E-07	sp|P36908|CHIA_CICAR Acidic endochitinase OS=Cicer arietinum PE=2 SV=1//2.03516e-138
Glyma.16G205200.Wm82.a2.v1	1.542	4.37E-74	1.53E-71	sp|Q9XF89|CB5_ARATH Chlorophyll a-b binding protein CP26, chloroplastic OS=Arabidopsis thaliana GN=LHCB5 PE=1 SV=1//5.02959e-141
Glyma.17G237100.Wm82.a2.v1	3.0164	1.24E-62	3.50E-60	sp|Q948Z4|SNAK1_SOLTU Snakin-1 OS=Solanum tuberosum GN=SN1 PE=1 SV=1//1.19277e-25
Glyma.18G259400.Wm82.a2.v1	2.2789	1.13E-07	2.45E-06	sp|P46690|GASA4_ARATH Gibberellin-regulated protein 4 OS=Arabidopsis thaliana GN=GASA4 PE=1 SV=2//1.72057e-37
Glyma.19G147400.Wm82.a2.v1	2.0514	6.18E-44	1.17E-41	sp|P48631|FD6E2_SOYBN Omega-6 fatty acid desaturase, endoplasmic reticulum isozyme 2 OS=Glycine max GN=FAD2-2 PE=2 SV=1//0
Glyma.20G027200.Wm82.a2.v1	2.1882	1.62E-53	3.83E-51	unknown
Glyma.20G167500.Wm82.a2.v1	3.1219	2.25E-21	1.76E-19	unknown

Table S9 The expression level of aquaporins in the GmSYP24ox transgenic line under osmotic/drought, salt or ABA stress.

S24N1L vs S24L				
Locus	log2.Fold_change.	pvalue	qvalue	Blast swiss prot
Glyma.19G181300.Wm82.a2.v1	-1.8936	3.12E-31	1.09E-29	sp|P43286|PIP21_ARATH Aquaporin PIP2-1 OS=Arabidopsis thaliana GN=PIP2-1 PE=1 SV=1//1.21882e-159
Glyma.03G078700.Wm82.a2.v1	-1.5052	2.96E-05	0.0001612	sp|Q39196|PIP14_ARATH Probable aquaporin PIP1-4 OS=Arabidopsis thaliana GN=PIP1.4 PE=1 SV=1//0
Glyma.03G180900.Wm82.a2.v1	5.659	7.52E-06	4.52E-05	sp|Q9ATM4|PIP27_MAIZE Aquaporin PIP2-7 OS=Zea mays GN=PIP2-7 PE=2 SV=1//7.13148e-158
Glyma.12G075400.Wm82.a2.v1	-3.3361	6.17E-17	1.14E-15	sp|Q9ATM4|PIP27_MAIZE Aquaporin PIP2-7 OS=Zea mays GN=PIP2-7 PE=2 SV=1//7.87722e-176
Glyma.12G172500.Wm82.a2.v1	2.2324	1.60E-35	6.52E-34	sp|Q9ATM4|PIP27_MAIZE Aquaporin PIP2-7 OS=Zea mays GN=PIP2-7 PE=2 SV=1//0
Glyma.01G208200.Wm82.a2.v1	-2.2134	0.0010242	0.0039515	sp|P21653|TIP1_TOBAC Probable aquaporin TIP-type RB7-5A OS=Nicotiana tabacum PE=2 SV=1//8.56123e-108
Glyma.04G083200.Wm82.a2.v1	-3.1633	1.23E-07	9.65E-07	sp|O82316|TIP41_ARATH Aquaporin TIP4-1 OS=Arabidopsis thaliana GN=TIP4-1 PE=2 SV=1//2.71385e-104
Glyma.06G084600.Wm82.a2.v1	-6.4231	5.57E-09	5.22E-08	sp|O82316|TIP41_ARATH Aquaporin TIP4-1 OS=Arabidopsis thaliana GN=TIP4-1 PE=2 SV=1//5.03132e-112
Glyma.11G034000.Wm82.a2.v1	-4.7633	1.79E-61	1.30E-59	sp|Q41975|TIP22_ARATH Probable aquaporin TIP2-2 OS=Arabidopsis thaliana GN=TIP2-2 PE=1 SV=2//1.28753e-108
Glyma.12G023600.Wm82.a2.v1	2.612	0.0008194	0.0032401	sp|Q9ATL5|TIP42_MAIZE Aquaporin TIP4-2 OS=Zea mays GN=TIP4-2 PE=2 SV=1//1.41957e-16
Glyma.13G146300.Wm82.a2.v1	-1.9586	1.21E-07	9.50E-07	sp|P25818|TIP11_ARATH Aquaporin TIP1-1 OS=Arabidopsis thaliana

				GN=TIP1-1 PE=1 SV=1//3.98186e-100
Glyma.13G224900.Wm82.a2.v1	-3.8681	3.57E-07	2.63E-06	sp|Q8LFP7|NIP12_ARATH Aquaporin NIP1-2 OS=Arabidopsis thaliana GN=NIP1-2 PE=2 SV=2//2.31568e-131
Glyma.16G210000.Wm82.a2.v1	6.1432	9.63E-09	8.73E-08	sp|P23958|TIPA_PHAVU Probable aquaporin TIP-type alpha OS=Phaseolus vulgaris PE=1 SV=1//1.5688e-132
Glyma.19G186100.Wm82.a2.v1	-1.4319	8.73E-28	2.68E-26	sp|Q9FY14|TIP1_MEDTR Probable aquaporin TIP-type OS=Medicago truncatula GN=AQP1 PE=1 SV=1//1.79911e-139
**S24N2L vs S24L**				
Locus	log2.Fold_change.	pvalue	qvalue	Blast swiss prot
Glyma.03G078700.Wm82.a2.v1	-2.7425	1.31E-09	5.11E-09	sp|Q39196|PIP14_ARATH Probable aquaporin PIP1-4 OS=Arabidopsis thaliana GN=PIP1.4 PE=1 SV=1//0
Glyma.03G180900.Wm82.a2.v1	7.1414	6.07E-10	2.46E-09	sp|Q9ATM4|PIP27_MAIZE Aquaporin PIP2-7 OS=Zea mays GN=PIP2-7 PE=2 SV=1//7.13148e-158
Glyma.10G211000.Wm82.a2.v1	-2.0288	2.78E-06	6.99E-06	sp|P43287|PIP22_ARATH Aquaporin PIP2-2 OS=Arabidopsis thaliana GN=PIP2-2 PE=1 SV=2//3.46181e-175
Glyma.11G228000.Wm82.a2.v1	3.6206	0.0020952	0.0029389	sp|P25794|PIP2_PEA Probable aquaporin PIP-type 7a OS=Pisum sativum GN=TRG-31 PE=2 SV=2//0
Glyma.12G075400.Wm82.a2.v1	-6.0915	1.45E-19	1.20E-18	sp|Q9ATM4|PIP27_MAIZE Aquaporin PIP2-7 OS=Zea mays GN=PIP2-7 PE=2 SV=1//7.87722e-176
Glyma.12G172500.Wm82.a2.v1	2.7414	1.10E-30	1.47E-29	sp|Q9ATM4|PIP27_MAIZE Aquaporin PIP2-7 OS=Zea mays GN=PIP2-7 PE=2 SV=1//0
Glyma.14G061500.Wm82.a2.v1	-3.4944	3.14E-256	5.83E-254	sp|P25794|PIP2_PEA Probable aquaporin PIP-type 7a OS=Pisum sativum GN=TRG-31 PE=2 SV=2//0
Glyma.19G181300.Wm82.a2.v1	-1.8146	2.39E-41	4.34E-40	sp|P43286|PIP21_ARATH Aquaporin PIP2-1 OS=Arabidopsis thaliana GN=PIP2-1 PE=1 SV=1//1.21882e-159
Glyma.20G179700.Wm82.a2.v1	-1.2547	1.07E-09	4.23E-09	sp|Q9SV31|PIP25_ARATH Probable aquaporin PIP2-5 OS=Arabidopsis

				thaliana GN=PIP2-5 PE=1 SV=1//2.42474e-174
Glyma.01G208200.Wm82.a2.v1	-3.2561	3.87E-05	7.96E-05	sp|P21653|TIP1_TOBAC Probable aquaporin TIP-type RB7-5A OS=Nicotiana tabacum PE=2 SV=1//8.56123e-108
Glyma.03G185900.Wm82.a2.v1	-3.9833	3.58E-49	7.75E-48	sp|Q9FY14|TIP1_MEDTR Probable aquaporin TIP-type OS=Medicago truncatula GN=AQP1 PE=1 SV=1//4.13105e-138
Glyma.04G083200.Wm82.a2.v1	-2.4955	5.31E-08	1.71E-07	sp|O82316|TIP41_ARATH Aquaporin TIP4-1 OS=Arabidopsis thaliana GN=TIP4-1 PE=2 SV=1//2.71385e-104
Glyma.06G084600.Wm82.a2.v1	-7.8808	5.20E-08	1.68E-07	sp|O82316|TIP41_ARATH Aquaporin TIP4-1 OS=Arabidopsis thaliana GN=TIP4-1 PE=2 SV=1//5.03132e-112
Glyma.10G221100.Wm82.a2.v1	4.4345	1.72E-08	5.90E-08	sp|Q9SV84|NIP51_ARATH Probable aquaporin NIP5-1 OS=Arabidopsis thaliana GN=NIP5-1 PE=2 SV=1//9.09463e-136
Glyma.11G034000.Wm82.a2.v1	-7.0479	1.24E-57	3.10E-56	sp|Q41975|TIP22_ARATH Probable aquaporin TIP2-2 OS=Arabidopsis thaliana GN=TIP2-2 PE=1 SV=2//1.28753e-108
Glyma.13G146300.Wm82.a2.v1	-3.9189	6.20E-13	3.32E-12	sp|P25818|TIP11_ARATH Aquaporin TIP1-1 OS=Arabidopsis thaliana GN=TIP1-1 PE=1 SV=1//3.98186e-100
Glyma.13G224900.Wm82.a2.v1	-4.0888	6.06E-08	1.93E-07	sp|Q8LFP7|NIP12_ARATH Aquaporin NIP1-2 OS=Arabidopsis thaliana GN=NIP1-2 PE=2 SV=2//2.31568e-131
Glyma.16G210000.Wm82.a2.v1	8.4148	1.80E-29	2.32E-28	sp|P23958|TIPA_PHAVU Probable aquaporin TIP-type alpha OS=Phaseolus vulgaris PE=1 SV=1//1.5688e-132
Glyma.19G186100.Wm82.a2.v1	-2.6303	2.21E-58	5.61E-57	sp|Q9FY14|TIP1_MEDTR Probable aquaporin TIP-type OS=Medicago truncatula GN=AQP1 PE=1 SV=1//1.79911e-139
**S24P4L vs S24L**				
Locus	log2.Fold_change.	pvalue	qvalue	Blast swiss prot
Glyma.02G255000.Wm82.a2.v1	1.9736	3.38E-84	3.88E-81	sp|P25794|PIP2_PEA Probable aquaporin PIP-type 7a OS=Pisum sativum GN=TRG-31 PE=2 SV=2//0
Glyma.03G078700.Wm82.a2.v1	1.4195	4.37E-06	0.000186	sp|Q39196|PIP14_ARATH Probable aquaporin PIP1-4 OS=Arabidopsis

			6	thaliana GN=PIP1.4 PE=1 SV=1//0
Glyma.10G211000.Wm82.a2.v1	4.0029	4.72E-76	4.87E-73	sp|P43287|PIP22_ARATH Aquaporin PIP2-2 OS=Arabidopsis thaliana GN=PIP2-2 PE=1 SV=2//3.46181e-175
Glyma.13G325900.Wm82.a2.v1	1.4467	3.06E-25	7.46E-23	sp|Q9ATM4|PIP27_MAIZE Aquaporin PIP2-7 OS=Zea mays GN=PIP2-7 PE=2 SV=1//0
Glyma.14G061500.Wm82.a2.v1	1.2008	1.02E-85	1.20E-82	sp|P25794|PIP2_PEA Probable aquaporin PIP-type 7a OS=Pisum sativum GN=TRG-31 PE=2 SV=2//0
Glyma.16G155100.Wm82.a2.v1	3.5931	2.60E-06	0.000115 1	sp|P43287|PIP22_ARATH Aquaporin PIP2-2 OS=Arabidopsis thaliana GN=PIP2-2 PE=1 SV=2//3.48282e-165
Glyma.20G179700.Wm82.a2.v1	1.7429	1.13E-14	1.36E-12	sp|Q9SV31|PIP25_ARATH Probable aquaporin PIP2-5 OS=Arabidopsis thaliana GN=PIP2-5 PE=1 SV=1//2.42474e-174
Glyma.03G185900.Wm82.a2.v1	1.1109	7.58E-14	8.41E-12	sp|Q9FY14|TIP1_MEDTR Probable aquaporin TIP-type OS=Medicago truncatula GN=AQP1 PE=1 SV=1//4.13105e-138
Glyma.11G143100.Wm82.a2.v1	4.8672	1.73E-14	2.06E-12	sp|O82598|TIP13_ARATH Aquaporin TIP1-3 OS=Arabidopsis thaliana GN=TIP1-3 PE=1 SV=1//6.95573e-136
Glyma.13G333100.Wm82.a2.v1	2.8634	3.84E-16	4.97E-14	sp|O82598|TIP13_ARATH Aquaporin TIP1-3 OS=Arabidopsis thaliana GN=TIP1-3 PE=1 SV=1//1.6409e-139
**S24P4R vs S24R**				
Locus	log2.Fold_change.	pvalue	qvalue	Blast swiss prot
Glyma.03G078700.Wm82.a2.v1	1.5271	0.000176 7	0.002433	sp|Q39196|PIP14_ARATH Probable aquaporin PIP1-4 OS=Arabidopsis thaliana GN=PIP1.4 PE=1 SV=1//0
Glyma.11G023200.Wm82.a2.v1	-1.6612	9.01E-24	1.01E-21	sp|Q7XSQ9|PIP12_ORYSJ Probable aquaporin PIP1-2 OS=Oryza sativa subsp. japonica GN=PIP1-2 PE=2 SV=3//1.77595e-180
Glyma.12G172500.Wm82.a2.v1	-1.0375	4.25E-09	1.43E-07	sp|Q9ATM4|PIP27_MAIZE Aquaporin PIP2-7 OS=Zea mays GN=PIP2-7 PE=2 SV=1//0
Glyma.13G325900.Wm82.a2.v1	-1.5726	1.18E-124	1.58E-121	sp|Q9ATM4|PIP27_MAIZE Aquaporin PIP2-7 OS=Zea mays GN=PIP2-7

				PE=2 SV=1//0
Glyma.16G155000.Wm82.a2.v1	1.8013	2.20E-07	5.70E-06	sp|P43287|PIP22_ARATH Aquaporin PIP2-2 OS=Arabidopsis thaliana GN=PIP2-2 PE=1 SV=2//2.43539e-164
Glyma.16G155100.Wm82.a2.v1	2.4703	1.20E-05	0.0002187	sp|P43287|PIP22_ARATH Aquaporin PIP2-2 OS=Arabidopsis thaliana GN=PIP2-2 PE=1 SV=2//3.48282e-165
Glyma.01G208200.Wm82.a2.v1	-1.5375	5.57E-84	3.40E-81	sp|P21653|TIP1_TOBAC Probable aquaporin TIP-type RB7-5A OS=Nicotiana tabacum PE=2 SV=1//8.56123e-108
Glyma.11G034000.Wm82.a2.v1	-1.494	1.88E-75	9.91E-73	sp|Q41975|TIP22_ARATH Probable aquaporin TIP2-2 OS=Arabidopsis thaliana GN=TIP2-2 PE=1 SV=2//1.28753e-108
Glyma.11G143100.Wm82.a2.v1	1.5806	0.0001159	0.0016699	sp|O82598|TIP13_ARATH Aquaporin TIP1-3 OS=Arabidopsis thaliana GN=TIP1-3 PE=1 SV=1//6.95573e-136
Glyma.13G333100.Wm82.a2.v1	5.1343	4.70E-22	4.78E-20	sp|O82598|TIP13_ARATH Aquaporin TIP1-3 OS=Arabidopsis thaliana GN=TIP1-3 PE=1 SV=1//1.6409e-139
Glyma.19G186100.Wm82.a2.v1	-1.1421	7.19E-119	8.07E-116	sp|Q9FY14|TIP1_MEDTR Probable aquaporin TIP-type OS=Medicago truncatula GN=AQP1 PE=1 SV=1//1.79911e-139
Glyma.09G238200.Wm82.a2.v1	-5.6833	1.15E-78	6.44E-76	sp|Q6Z2T3|NIP21_ORYSJ Aquaporin NIP2-1 OS=Oryza sativa subsp. japonica GN=NIP2-1 PE=1 SV=1//3.71203e-92
Glyma.18G259500.Wm82.a2.v1	-2.9387	1.15E-10	4.72E-09	sp|Q6Z2T3|NIP21_ORYSJ Aquaporin NIP2-1 OS=Oryza sativa subsp. japonica GN=NIP2-1 PE=1 SV=1//1.04006e-92
Glyma.19G108400.Wm82.a2.v1	-1.2588	1.13E-13	6.26E-12	sp|Q9ATM2|SIP12_MAIZE Aquaporin SIP1-2 OS=Zea mays GN=SIP1-2 PE=2 SV=1//6.2894e-74
**S24P10L vs S24L**				
Locus	log2.Fold_change.	pvalue	qvalue	Blast swiss prot
Glyma.02G255000.Wm82.a2.v1	1.4353	1.97E-30	2.14E-28	sp|P25794|PIP2_PEA Probable aquaporin PIP-type 7a OS=Pisum sativum GN=TRG-31 PE=2 SV=2//0
Glyma.03G180900.Wm82.a2.v1	7.1822	7.94E-16	4.08E-14	sp|Q9ATM4|PIP27_MAIZE Aquaporin PIP2-7 OS=Zea mays GN=PIP2-7

				PE=2 SV=1//7.13148e-158
Glyma.10G211000.Wm82.a2.v1	2.814	3.89E-23	3.19E-21	sp|P43287|PIP22_ARATH Aquaporin PIP2-2 OS=Arabidopsis thaliana GN=PIP2-2 PE=1 SV=2//3.46181e-175
Glyma.16G155100.Wm82.a2.v1	3.2682	0.0001118	0.0014843	sp|P43287|PIP22_ARATH Aquaporin PIP2-2 OS=Arabidopsis thaliana GN=PIP2-2 PE=1 SV=2//3.48282e-165
Glyma.02G255000.Wm82.a2.v1	1.4353	1.97E-30	2.14E-28	sp|P25794|PIP2_PEA Probable aquaporin PIP-type 7a OS=Pisum sativum GN=TRG-31 PE=2 SV=2//0
Glyma.19G181300.Wm82.a2.v1	1.6089	1.07E-36	1.53E-34	sp|P43286|PIP21_ARATH Aquaporin PIP2-1 OS=Arabidopsis thaliana GN=PIP2-1 PE=1 SV=1//1.21882e-159
Glyma.04G083200.Wm82.a2.v1	-3.9144	3.74E-08	9.09E-07	sp|O82316|TIP41_ARATH Aquaporin TIP4-1 OS=Arabidopsis thaliana GN=TIP4-1 PE=2 SV=1//2.71385e-104
Glyma.06G084600.Wm82.a2.v1	-3.3701	7.72E-08	1.80E-06	sp|O82316|TIP41_ARATH Aquaporin TIP4-1 OS=Arabidopsis thaliana GN=TIP4-1 PE=2 SV=1//5.03132e-112
Glyma.11G034000.Wm82.a2.v1	-2.1254	1.34E-34	1.72E-32	sp|Q41975|TIP22_ARATH Probable aquaporin TIP2-2 OS=Arabidopsis thaliana GN=TIP2-2 PE=1 SV=2//1.28753e-108
Glyma.11G143100.Wm82.a2.v1	3.2988	0.0001835	0.0023031	sp|O82598|TIP13_ARATH Aquaporin TIP1-3 OS=Arabidopsis thaliana GN=TIP1-3 PE=1 SV=1//6.95573e-136
Glyma.13G333100.Wm82.a2.v1	1.9577	1.36E-05	0.0002197	sp|O82598|TIP13_ARATH Aquaporin TIP1-3 OS=Arabidopsis thaliana GN=TIP1-3 PE=1 SV=1//1.6409e-139
Glyma.13G224900.Wm82.a2.v1	-1.929	0.0002146	0.0026527	sp|Q8LFP7|NIP12_ARATH Aquaporin NIP1-2 OS=Arabidopsis thaliana GN=NIP1-2 PE=2 SV=2//2.31568e-131
**S24P10R vs S24R**				
Locus	log2.Fold_change.	pvalue	qvalue	Blast swiss prot
Glyma.01G220600.Wm82.a2.v1	-1.0456	5.90E-07	1.34E-05	sp|Q39196|PIP14_ARATH Probable aquaporin PIP1-4 OS=Arabidopsis thaliana GN=PIP1.4 PE=1 SV=1//0
Glyma.05G208700.Wm82.a2.v1	-1.5477	1.53E-09	5.24E-08	sp|Q7XSQ9|PIP12_ORYSJ Probable aquaporin PIP1-2 OS=Oryza sativa

				subsp. japonica GN=PIP1-2 PE=2 SV=3//0
Glyma.11G023200.Wm82.a2.v1	-1.1189	1.33E-15	8.09E-14	sp|Q7XSQ9|PIP12_ORYSJ Probable aquaporin PIP1-2 OS=Oryza sativa subsp. japonica GN=PIP1-2 PE=2 SV=3//1.77595e-180
Glyma.11G146500.Wm82.a2.v1	-1.2914	1.01E-06	2.22E-05	sp|Q9ATM4|PIP27_MAIZE Aquaporin PIP2-7 OS=Zea mays GN=PIP2-7 PE=2 SV=1//0
Glyma.13G325900.Wm82.a2.v1	-1.4212	2.08E-116	1.61E-113	sp|Q9ATM4|PIP27_MAIZE Aquaporin PIP2-7 OS=Zea mays GN=PIP2-7 PE=2 SV=1//0
Glyma.01G208200.Wm82.a2.v1	-2.1452	7.68E-132	7.48E-129	sp|P21653|TIP1_TOBAC Probable aquaporin TIP-type RB7-5A OS=Nicotiana tabacum PE=2 SV=1//8.56123e-108
Glyma.04G083200.Wm82.a2.v1	-1.474	2.51E-35	4.18E-33	sp|O82316|TIP41_ARATH Aquaporin TIP4-1 OS=Arabidopsis thaliana GN=TIP4-1 PE=2 SV=1//2.71385e-104
Glyma.08G203000.Wm82.a2.v1	-1.985	4.72E-06	9.12E-05	sp|Q41951|TIP21_ARATH Aquaporin TIP2-1 OS=Arabidopsis thaliana GN=TIP2-1 PE=1 SV=2//7.86985e-112
Glyma.11G034000.Wm82.a2.v1	-1.8039	5.70E-101	3.48E-98	sp|Q41975|TIP22_ARATH Probable aquaporin TIP2-2 OS=Arabidopsis thaliana GN=TIP2-2 PE=1 SV=2//1.28753e-108
Glyma.19G186100.Wm82.a2.v1	-1.0718	4.28E-119	3.43E-116	sp|Q9FY14|TIP1_MEDTR Probable aquaporin TIP-type OS=Medicago truncatula GN=AQP1 PE=1 SV=1//1.79911e-139
Glyma.09G238200.Wm82.a2.v1	-7.2272	9.14E-74	3.40E-71	sp|Q6Z2T3|NIP21_ORYSJ Aquaporin NIP2-1 OS=Oryza sativa subsp. japonica GN=NIP2-1 PE=1 SV=1//3.71203e-92
Glyma.16G043800.Wm82.a2.v1	1.1158	2.22E-05	0.000368 9	sp|Q9ATM2|SIP12_MAIZE Aquaporin SIP1-2 OS=Zea mays GN=SIP1-2 PE=2 SV=1//1.074e-64
Glyma.18G259500.Wm82.a2.v1	-3.0228	4.15E-11	1.70E-09	sp|Q6Z2T3|NIP21_ORYSJ Aquaporin NIP2-1 OS=Oryza sativa subsp. japonica GN=NIP2-1 PE=1 SV=1//1.04006e-92
**S24A1L vs S24L**				
Locus	log2.Fold_change.	pvalue	qvalue	Blast swiss prot
Glyma.01G220600.Wm82.a2.v1	2.85	6.43E-07	1.23E-05	sp|Q39196|PIP14_ARATH Probable aquaporin PIP1-4 OS=Arabidopsis

				thaliana GN=PIP1.4 PE=1 SV=1//0
Glyma.02G255000.Wm82.a2.v1	1.7729	2.59E-58	6.95E-56	sp|P25794|PIP2_PEA Probable aquaporin PIP-type 7a OS=Pisum sativum GN=TRG-31 PE=2 SV=2//0
Glyma.03G180900.Wm82.a2.v1	6.4452	1.44E-10	4.81E-09	sp|Q9ATM4|PIP27_MAIZE Aquaporin PIP2-7 OS=Zea mays GN=PIP2-7 PE=2 SV=1//7.13148e-158
Glyma.08G015300.Wm82.a2.v1	5.6265	1.71E-15	8.82E-14	sp|Q7XSQ9|PIP12_ORYSJ Probable aquaporin PIP1-2 OS=Oryza sativa subsp. japonica GN=PIP1-2 PE=2 SV=3//0
Glyma.05G208700.Wm82.a2.v1	3.5809	2.30E-16	1.28E-14	sp|Q7XSQ9|PIP12_ORYSJ Probable aquaporin PIP1-2 OS=Oryza sativa subsp. japonica GN=PIP1-2 PE=2 SV=3//0
Glyma.10G211000.Wm82.a2.v1	2.0977	9.87E-11	3.34E-09	sp|P43287|PIP22_ARATH Aquaporin PIP2-2 OS=Arabidopsis thaliana GN=PIP2-2 PE=1 SV=2//3.46181e-175
Glyma.11G146500.Wm82.a2.v1	3.1783	6.24E-07	1.20E-05	sp|Q9ATM4|PIP27_MAIZE Aquaporin PIP2-7 OS=Zea mays GN=PIP2-7 PE=2 SV=1//0
Glyma.12G075400.Wm82.a2.v1	2.3425	1.19E-38	1.89E-36	sp|Q9ATM4|PIP27_MAIZE Aquaporin PIP2-7 OS=Zea mays GN=PIP2-7 PE=2 SV=1//7.87722e-176
Glyma.12G172500.Wm82.a2.v1	1.0966	1.02E-07	2.25E-06	sp|Q9ATM4|PIP27_MAIZE Aquaporin PIP2-7 OS=Zea mays GN=PIP2-7 PE=2 SV=1//0
Glyma.13G325900.Wm82.a2.v1	1.2707	1.42E-16	8.00E-15	sp|Q9ATM4|PIP27_MAIZE Aquaporin PIP2-7 OS=Zea mays GN=PIP2-7 PE=2 SV=1//0
Glyma.14G061500.Wm82.a2.v1	1.0535	8.51E-54	2.04E-51	sp|P25794|PIP2_PEA Probable aquaporin PIP-type 7a OS=Pisum sativum GN=TRG-31 PE=2 SV=2//0
Glyma.19G181300.Wm82.a2.v1	2.5944	1.80E-154	1.79E-151	sp|P43286|PIP21_ARATH Aquaporin PIP2-1 OS=Arabidopsis thaliana GN=PIP2-1 PE=1 SV=1//1.21882e-159
Glyma.20G179700.Wm82.a2.v1	1.5905	6.58E-11	2.26E-09	sp|Q9SV31|PIP25_ARATH Probable aquaporin PIP2-5 OS=Arabidopsis thaliana GN=PIP2-5 PE=1 SV=1//2.42474e-174
Glyma.03G185900.Wm82.a2.v1	3.0357	1.05E-211	1.82E-208	sp|Q9FY14|TIP1_MEDTR Probable aquaporin TIP-type OS=Medicago

				truncatula GN=AQP1 PE=1 SV=1//4.13105e-138
Glyma.07G018000.Wm82.a2.v1	4.7891	2.60E-13	1.13E-11	sp|Q41951|TIP21_ARATH Aquaporin TIP2-1 OS=Arabidopsis thaliana GN=TIP2-1 PE=1 SV=2//9.26677e-112
Glyma.08G203000.Wm82.a2.v1	2.5759	9.56E-05	0.001159	sp|Q41951|TIP21_ARATH Aquaporin TIP2-1 OS=Arabidopsis thaliana GN=TIP2-1 PE=1 SV=2//7.86985e-112
Glyma.11G034000.Wm82.a2.v1	1.0229	4.45E-13	1.90E-11	sp|Q41975|TIP22_ARATH Probable aquaporin TIP2-2 OS=Arabidopsis thaliana GN=TIP2-2 PE=1 SV=2//1.28753e-108
Glyma.15G018100.Wm82.a2.v1	5.3221	2.62E-23	2.29E-21	sp|Q41951|TIP21_ARATH Aquaporin TIP2-1 OS=Arabidopsis thaliana GN=TIP2-1 PE=1 SV=2//3.77304e-115
Glyma.19G186100.Wm82.a2.v1	3.1099	0	0	sp|Q9FY14|TIP1_MEDTR Probable aquaporin TIP-type OS=Medicago truncatula GN=AQP1 PE=1 SV=1//1.79911e-139
Glyma.06G307000.Wm82.a2.v1	2.6283	4.46E-05	0.0005873	sp|Q9ATM2|SIP12_MAIZE Aquaporin SIP1-2 OS=Zea mays GN=SIP1-2 PE=2 SV=1//7.29961e-66
**S24A1R vs S24R**				
Locus	log2.Fold_change.	pvalue	qvalue	Blast swiss prot
Glyma.02G255000.Wm82.a2.v1	1.2037	3.02E-111	7.42E-108	sp|P25794|PIP2_PEA Probable aquaporin PIP-type 7a OS=Pisum sativum GN=TRG-31 PE=2 SV=2//0
Glyma.16G155000.Wm82.a2.v1	1.4911	8.41E-06	0.0004411	sp|P43287|PIP22_ARATH Aquaporin PIP2-2 OS=Arabidopsis thaliana GN=PIP2-2 PE=1 SV=2//2.43539e-164
Glyma.09G238200.Wm82.a2.v1	-1.9697	1.01E-34	5.32E-32	sp|Q6Z2T3|NIP21_ORYSJ Aquaporin NIP2-1 OS=Oryza sativa subsp. japonica GN=NIP2-1 PE=1 SV=1//3.71203e-92
Glyma.19G108400.Wm82.a2.v1	-1.0846	9.63E-10	9.73E-08	sp|Q9ATM2|SIP12_MAIZE Aquaporin SIP1-2 OS=Zea mays GN=SIP1-2 PE=2 SV=1//6.2894e-74
**S24A3L vs S24L**				
Locus	log2.Fold_change.	pvalue	qvalue	Blast swiss prot
Glyma.03G180900.Wm82.a2.v1	6.7068	1.76E-13	1.44E-11	sp|Q9ATM4|PIP27_MAIZE Aquaporin PIP2-7 OS=Zea mays GN=PIP2-7

				PE=2 SV=1//7.13148e-158
Glyma.05G208700.Wm82.a2.v1	3.0156	1.63E-11	1.11E-09	sp|Q7XSQ9|PIP12_ORYSJ Probable aquaporin PIP1-2 OS=Oryza sativa subsp. japonica GN=PIP1-2 PE=2 SV=3//0
Glyma.08G015300.Wm82.a2.v1	4.6566	3.94E-09	2.04E-07	sp|Q7XSQ9|PIP12_ORYSJ Probable aquaporin PIP1-2 OS=Oryza sativa subsp. japonica GN=PIP1-2 PE=2 SV=3//0
Glyma.12G075400.Wm82.a2.v1	1.5905	2.01E-17	2.31E-15	sp|Q9ATM4|PIP27_MAIZE Aquaporin PIP2-7 OS=Zea mays GN=PIP2-7 PE=2 SV=1//7.87722e-176
Glyma.19G181300.Wm82.a2.v1	1.9465	1.08E-83	8.12E-81	sp|P43286|PIP21_ARATH Aquaporin PIP2-1 OS=Arabidopsis thaliana GN=PIP2-1 PE=1 SV=1//1.21882e-159
Glyma.03G185900.Wm82.a2.v1	1.6317	1.03E-44	3.33E-42	sp|Q9FY14|TIP1_MEDTR Probable aquaporin TIP-type OS=Medicago truncatula GN=AQP1 PE=1 SV=1//4.13105e-138
Glyma.07G018000.Wm82.a2.v1	3.7763	2.79E-07	1.07E-05	sp|Q41951|TIP21_ARATH Aquaporin TIP2-1 OS=Arabidopsis thaliana GN=TIP2-1 PE=1 SV=2//9.26677e-112
Glyma.15G018100.Wm82.a2.v1	4.2759	1.73E-12	1.29E-10	sp|Q41951|TIP21_ARATH Aquaporin TIP2-1 OS=Arabidopsis thaliana GN=TIP2-1 PE=1 SV=2//3.77304e-115
Glyma.19G186100.Wm82.a2.v1	1.3203	3.59E-36	8.72E-34	sp|Q9FY14|TIP1_MEDTR Probable aquaporin TIP-type OS=Medicago truncatula GN=AQP1 PE=1 SV=1//1.79911e-139
**S24A3R vs S24R**				
Locus	log2.Fold_change.	pvalue	qvalue	Blast swiss prot
Glyma.10G211000.Wm82.a2.v1	-1.2424	2.93E-15	2.64E-13	sp|P43287|PIP22_ARATH Aquaporin PIP2-2 OS=Arabidopsis thaliana GN=PIP2-2 PE=1 SV=2//3.46181e-175
Glyma.01G208200.Wm82.a2.v1	-1.8079	8.46E-97	8.56E-94	sp|P21653|TIP1_TOBAC Probable aquaporin TIP-type RB7-5A OS=Nicotiana tabacum PE=2 SV=1//8.56123e-108
Glyma.03G185900.Wm82.a2.v1	-1.1135	4.80E-98	5.24E-95	sp|Q9FY14|TIP1_MEDTR Probable aquaporin TIP-type OS=Medicago truncatula GN=AQP1 PE=1 SV=1//4.13105e-138
Glyma.04G083200.Wm82.a2.v1	-2.1545	3.31E-50	1.45E-47	sp|O82316|TIP41_ARATH Aquaporin TIP4-1 OS=Arabidopsis thaliana

				GN=TIP4-1 PE=2 SV=1//2.71385e-104
Glyma.06G084600.Wm82.a2.v1	-1.6221	1.78E-11	1.13E-09	sp|O82316|TIP41_ARATH Aquaporin TIP4-1 OS=Arabidopsis thaliana GN=TIP4-1 PE=2 SV=1//5.03132e-112
Glyma.07G018000.Wm82.a2.v1	1.4453	6.43E-07	2.33E-05	sp|Q41951|TIP21_ARATH Aquaporin TIP2-1 OS=Arabidopsis thaliana GN=TIP2-1 PE=1 SV=2//9.26677e-112
Glyma.11G034000.Wm82.a2.v1	-1.3318	1.20E-58	6.71E-56	sp|Q41975|TIP22_ARATH Probable aquaporin TIP2-2 OS=Arabidopsis thaliana GN=TIP2-2 PE=1 SV=2//1.28753e-108
Glyma.09G238200.Wm82.a2.v1	-5.1685	9.41E-78	7.36E-75	sp|Q6Z2T3|NIP21_ORYSJ Aquaporin NIP2-1 OS=Oryza sativa subsp. japonica GN=NIP2-1 PE=1 SV=1//3.71203e-92
Glyma.15G003900.Wm82.a2.v1	1.9064	0.0001142	0.0025937	sp|Q9SAI4|NIP61_ARATH Aquaporin NIP6-1 OS=Arabidopsis thaliana GN=NIP6-1 PE=1 SV=1//2.87757e-112
Glyma.18G259500.Wm82.a2.v1	-2.1677	8.39E-08	3.47E-06	sp|Q6Z2T3|NIP21_ORYSJ Aquaporin NIP2-1 OS=Oryza sativa subsp. japonica GN=NIP2-1 PE=1 SV=1//1.04006e-92

Table S10. The information of all primers in the manuscript.

SYP24-F1	ACATCATTTCCCACTCCCG
SYP24-R1	GGACCTATGTCCAAAAGTAACCA
SYP24-F2	TCTGGCTCATAGTCCGTCCG
SYP24-R2	GCCTCTTGTTGGGGTTTCG
SYP24-F3	cgacgacaagaccgtgACATCATTTCCCACTCCCG
SYP24-R3	gaggagaagagccgtGGACCTATGTCCAAAAGTAACCA
Atactin-F	GAAGTTCAATGTTTCGTTTCATGT
Atactin-R	GGATTATACAAGGCCCCAAAA
Gmactin-F	ATCTTGACTGAGCGTGGTTATTCC
Gmactin-R	GCTGGTCCTGGCTGTCTCC
RD29AF	GGCGTAACAGGTAAACCTAGAG
RD29AR	TCCGATGTAAACGTCGTCC
RD22F	GGTTCGGAAGAAGCGGAG
RD22R	GAAACAGCCCTGACGTGATAT
COR47F	GGAGTACAAGAACAACGTTCCCGA
COR47R	TGTCGTCGCTGGTGATTCCTCT
NCED3F	CAGCTTGTAGCTTTTGGGCTGTA
NCED3R	TAACAGAAACCAGCTGAGCTCGA
COR15AF	GGCCACAAAGAAAGCTTCAG
COR15AR	CTTGTTTGCGGCTTCTTTTC
KIN1F	AACAAGAATGCCTTCCAAGC
KIN1R	CGCATCCGATACACTCTTTCC
P5CSF	GCGCATAGTTTCTGATGCAA
P5CSR	TGCAACTTCGTGATCCTCTG
ABI1F	AGAGTGTGCCTTTGTATGGTTTTA
ABI1R	CATCCTCTCTCTACAATAGTTCGCT
ABI2F	GATGGAAGATTCTGTCTCAACGATT
ABI2R	GTTTCTCCTTCACTATCTCCTCCG
ABI4F	ACTCCAAGTTCCGTTACCGTG
ABI4R	GGGGTTAAGTTGAGCTGAGCA
ABI5F	CAATAAGAGAGGGATAGCGAACGAG
ABI5R	CGTCCATTGCTGTCTCCTCCA
ABF4F	AACAACTTAGGAGGTGGTGGTC
ABF4R	CTTCAGGAGTTCATCCATGTTC
ABA3F	TGCACACAACGTCACTTTCCATATA
ABA3R	GACCGTAGCTCAGAATGGGATAACT
CIPK3F	AGAATCTTAGATCCGAATCCGATGAC
CIPK3R	CCTCCTCTCAATGTTATCCTTGTTTC
RGALF	CCCGGTTCCATCGATAAGTTTT
RGALR	TCGCATGCCACAAGGTTTAGTAT
IAA28F	ACCAATTCACTTCCAACAACAAC
IAA28R	CTTGCCATGTTTTCTAGGTGAG

Cu/Zn SODF	ATGGCGAAAGGAGTTGCAGT
Cu/Zn SODR	TTAGCCCTGGAGACCAATGA
